# Osteoporosis in Europe: a compendium of country-specific reports

**DOI:** 10.1007/s11657-021-00969-8

**Published:** 2022-01-26

**Authors:** Carl Willers, Nicholas Norton, Nicholas C Harvey, Trolle Jacobson, Helena Johansson, Mattias Lorentzon, Eugene V McCloskey, Fredrik Borgström, John A Kanis, the SCOPE review panel of the IOF

**Affiliations:** 1 Quantify Research, Stockholm, Sweden; 2 Department of Neurobiology, Care Sciences and Society, Karolinska Institutet, Stockholm, Sweden; 3 MRC Lifecourse Epidemiology Unit, University of Southampton, Southampton, UK; 4 NIHR Southampton Biomedical Research Centre, University of Southampton and University Hospital Southampton NHS Foundation Trust, Southampton, UK; 5 Mary McKillop Institute for Health Research, Australian Catholic University, Melbourne, Australia; 6 Centre for Metabolic Bone Diseases, University of Sheffield Medical School, Beech Hill Road, Sheffield, S10 2RX UK; 7 Geriatric Medicine, Institute of Medicine, Sahlgrenska Academy, University of Gothenburg, Gothenburg, Sweden; 8 Department of Learning, Informatics, Management and Ethics (LIME), Karolinska Institutet, Stockholm, Sweden; 9 MRC Versus Arthritis Centre for Integrated Research in Musculoskeletal Ageing, Mellanby Centre for Bone Research, University of Sheffield, Sheffield, UK

**Keywords:** Epidemiology, Fracture, Economic burden, European Union, Treatment, Health technology assessment

## Abstract

**Summary:**

This report describes epidemiology, burden, and treatment of osteoporosis in each of the 27 countries of the European Union plus Switzerland and the UK (EU 27+2).

**Introduction:**

The aim of this report was to characterize the burden of osteoporosis in each of the countries of the European Union plus Switzerland and the UK in 2019 and beyond.

**Methods:**

The data on fracture incidence and costs of fractures in the EU27+2 was taken from a concurrent publication in this journal (SCOPE 2021: a new scorecard for osteoporosis in Europe) and country-specific information extracted. The information extracted covered four domains: burden of osteoporosis and fractures; policy framework; service provision; and service uptake.

**Results:**

The clinical and economic burden of osteoporotic fractures in 2019 is given for each of the 27 countries of the EU plus Switzerland and the UK. Each domain was ranked and the country performance set against the scorecard for all nations studied. Data were also compared with the first SCOPE undertaken in 2010. Fifteen of the 16 score card metrics on healthcare provision were used in the two surveys. Scores had improved or markedly improved in 15 countries, remained constant in 8 countries and worsened in 3 countries. The average treatment gap increased from 55% in 2010 to 71% in 2019. Overall, 10.6 million women who were eligible for treatment were untreated in 2010. In 2019, this number had risen to 14.0 million.

**Conclusions:**

In spite of the high cost of osteoporosis, a substantial treatment gap and projected increase of the economic burden driven by aging populations, the use of pharmacological prevention of osteoporosis has decreased in recent years, suggesting that a change in healthcare policy concerning the disease is warranted.

*SCOPE review panel of the IOF

**Table:** 

Country	Name	Affiliation	Contact
Austria	Hans Peter Dimai	Division of Endocrinology and Diabetology, Department of Medicine, Medical University of Graz, Graz, Austria	hans.dimai@medunigraz.at
	Christian Muschitz	Medical Department II, St. Vincent Hospital, Vienna, Austria	christian.muschitz@meduniwien.ac.at
Belgium	Jean-Francois Kaux	Department of Physical and Rehabilitation Medicine, University Hospital and University of Liège, Belgium	jfkaux@chuliege.be
	Jean-Yves Reginster	Division of Public Health, Epidemiology and Health Economics, University of Liège, World Health Organization Collaborating Centre for Public Health aspects of musculo-skeletal health and ageing, Liège, Belgium	jyr.ch@bluewin.ch
		Biochemistry Department, College of Science, King Saud University, Riyadh, Saudi Arabia.	
	Olivier Bruyère	Division of Public Health, Epidemiology and Health Economics, University of Liège, World Health Organization Collaborating Centre for Public Health aspects of musculo-skeletal health and ageing, Liège, Belgium	olivier.bruyere@uliege.be
	Etienne Cavalier	Department of Clinical Chemistry, CHU de Liege, University of Liege, Liège, Belgium.	Etienne.Cavalier@uliege.be
	Marie-Paule Lecart	University of Liège, Bone and Cartilage Metabolism Research Unit, Department of Physical and Rehabilitation Medicine, Department of Geriatrics, CHU Centre Ville, Liège, Belgium	mplecart@chuliege.be
Bulgaria	Anna-Maria Borissova	University Hospital Sofiamed, Faculty of Medicine, Sofia University “Saint Kliment Ohridski”, Sofia-, Bulgaria; Bulgarian League for the Prevention of Osteoporosis	anmarbor@abv.bg
	Mihail Boyanov	University Hospital Alexandrovska, Sofia, Bulgaria; Bulgarian Society for Clinical Densitometry	mihailboyanov@yahoo.com
	Zlatimir Kolarov	University Hospital Sv. Ivan Rilski, Department of Rheumatology, Faculty of Medicine, Medical University Sofia, Sofia, Bulgaria; Bulgarian Association for Osteoporosis and Osteoarthrosis	zkolarov@abv.bg
Croatia	Simeon Grazio	Department of Rheumatology, Physical and Rehabilitation Medicine, University Clinical Centre Sisters of Mercy, Zagreb, Croatia	simeon.grazio@zg.t-com.hr
	Velimir Altabas	Department of Endocrinology, Diabetes and Metabolic Diseases, University Clinical Centre Sisters of Mercy, Zagreb, Croatia	velimir.altabas@gmail.com
	Zlatko Giljević	Department of Endocrinology, University Clinical Centre Zagreb, Zagreb, Croatia	zlatko.giljevic@kbc-zagreb.hr
Cyprus	George L Georgiades	Deputy President of the Cyprus Association against Osteoporosis	geoendo@cytanet.com.cy
Czechia	Vladimir Palicka	Osteology Centre, University Hospital and School of Medicine, Charles University, Hradec Kralove, Czech Republic	Palicka@lfhk.cuni.cz
	Richard Pikner	Department of Clinical Biochemistry and Bone Metabolism, Klatovy Hospital, Klatovy, Czech Republic	richard.pikner@klatovy.nemocnicepk.cz
		Department of Clinical Biochemistry and Haematology, Faculty of Medicine Pilsen, Charles University Prague, Pilsen, Czech Republic	
		Faculty of Health Care Studies, University of West Bohemia, Pilsen, Czech Republic	
	Jan Rosa	Osteology Centre, Affidea Praha, Prague, Czech Republic	rosaj@affidea-praha.cz
	Petr Kasalicky	Osteology Centre, Affidea Praha, Prague, Czech Republic	kasalickyp@affidea-praha.cz
Denmark	Pernille Hermann	Department of Endocrinology, Odense University Hospital, Denmark	Pernille.Hermann@rsyd.dk
		Department of Clinical Research, University of Southern Denmark, Odense, Denmark	
	Bo Abrahamsen	Department of Medicine, Holbæk Hospital, DK-4300, Holbæk, Denmark.	b.abrahamsen@physician.dk.
Estonia	Katre Maasalu	Tartu University Hospital, Clinic of Traumatology and Orthopaedics, Estonia	katre.maasalu@kliinikum.ee
		University of Tartu, Department of Traumatology and Orthopaedics, Estonia	
	Eiki Strauss	Tartu University Hospital, Clinic of Traumatology and Orthopaedics, Estonia	eiki.strauss@kliinikum.ee
Finland	Ansa Holm	Suomen Luustoliitto ry, Köydenpunojankatu 8 G, 00180 Helsinki, Finland	ansa.holm@luustoliitto.fi
France	Bernard Cortet	Department of Rheumatology and EA 4490, University-Hospital of Lille, Lille, France	Bernard.CORTET@CHRU-LILLE.FR
	Thierry Thomas	Department of Rheumatology, Hôpital Nord, CHU Saint-Etienne, and INSERM U1059, Lyon University, Saint-Etienne, France	thierry.thomas@chuse.fr
	Laurent Grange	Department of Rheumatology, AFLAR, Grenoble Alpes University Hospital, Grenoble, France	LGrange@chu-grenoble.fr
	Francoise Alliot Launois	AFLAR - Association Française de Lutte Anti-Rhumatismale, Paris, France.	francoisealliotlaunois@gmail.com
Germany	Gisela Klatt	Bundesselbsthilfeverband für Osteoporose e.V. (BfO) Federal Self-Help Association for Osteoporosis, Düsseldorf, Germany	gisela-klatt@t-online.de
	Stephan Scharla	Salinenstr. 8, 83435 Bad Reichenhall, Germany.	sscharla@gmx.de
	Andreas Kurth	Department of Orthopaedic and Trauma Surgery, Campus Kemperhof, Community Clinics Middle Rhine, Koblenz - Germany	kurth@dv-osteologie.de
Greece	Polyzois Makras	Department of Endocrinology and Diabetes, 251 Hellenic Air Force General Hospital, Athens, Greece,	pmakras@gmail.com
	Tatiana Drakopoulou	Butterfly Bone Health Society, Athens, Greece	tatiana@osteocare.gr
	George Trovas	Laboratory of musculoskeletal diseases, University of Athens, Athens, Greece	trovas1@otenet.gr
	George P Lyritis,	Hellenic Osteoporosis Foundation, Athens, Greece	glyritis@heliost.gr
	Stavroula Rizou	Hellenic Osteoporosis Foundation, Athens, Greece	st.rizou@heliost.gr
Hungary	Istvan Takacs	Semmelweis University, Department of Internal Medicine and Oncology	takacs.istvan@med.semmelweis-univ.hu
	Judit Donáth	National Institute of Rheumatology and Physiotherapy, Budapest, Hungary	donjudit@gmail.com
	László Szekeres	National Institute of Rheumatology and Physiotherapy, Budapest, Hungary	szekeres.laszlo@mail.orfi.hu
Ireland	Moira O'Brien	Irish Osteoporosis Society, Clonskeagh, Dublin, Ireland	info@irishosteoporosis.ie
	Michelle O’Brien	Irish Osteoporosis Society, Clonskeagh, Dublin, Ireland	info@irishosteoporosis.ie
Italy	Ferdinando Silveri	Department of Rheumatology, Università Politecnica delle Marche, Ancona, Italy	ferdinando.silveri@sanita.marche.it
		Italian Federation of Osteoporosis and Diseases of the Skeleton (FEDIOS), Falconara Marittima, Italy	
	Maurizio Rossini	Rheumatology Unit, University of Verona, Policlinico Borgo Roma, Verona, Italy	maurizio.rossini@univr.it
		Italian Society for Osteoporosis, Mineral Metabolism and Bone Diseases (SIOMMMS), Verona, Italy	
	Maria Luisa Brandi	Fondazione Italiana sulla Ricerca per le Malattie dell'Osso (F.I.R.M.O.), Florence, Italy	info@fondazionefirmo.com
Latvia	Ingvars Rasa	Latvian Osteoporosis and Bone Metabolic Diseases Association (LOKMSA), Riga East Clinical University Hospital Rīga Stradiņš University; Riga, Latvia	dr.irasa@inbox.lv
Lithuania	Alekna Vidmantas	Faculty of Medicine, Vilnius University, Vilnius, Lithuania	vidmantas.alekna@osteo.lt
	Marija Tamulaitiene	Faculty of Medicine, Vilnius University, Vilnius, Lithuania	marija.tamulaitiene@mf.vu.lt
Malta	Raymond Galea	Department of Obstetrics and Gynaecology, University of Malta, Mater Dei Hospital, Malta	raymond.galea@um.edu.mt
		Malta Osteoporosis Society, c/o Department of Obstetrics & Gynaecology, Sptar Mater Dei, Malta	
	Neville Calleja	Health Information & Research, Ministry for Health, Malta	neville.calleja@gov.mt
Netherlands	Harry van den Broek	Osteoporose Vereniging, PO Box 418,2000 AK Haarlem, Netherlands	hvdbroek@osteoporosevereniging.nl
	Geraldine EMP Willemsen-De Mey	National Association ReumaZorg Nederland, Nijmegen, Netherlands	voorzitter@reumazorgnederland.nl
	Hendrien Witte	Osteoporose Vereniging, PO Box 418,2000 AK Haarlem, Netherlands	hwitte@osteoporosevereniging.nl
Poland	Edward Czerwiński	Jagiellonian University, Faculty of Health Sciences, Institute of Physiotherapy, Rehabilitation Clinics, Krakow, Poland	czerwinski@kcm.pl
	Janusz E. Badurski	The Polish Foundation of Osteoporosis Research Team, Białystok, Poland.	badurski@pfo.com.pl
Portugal	José António P. Da Silva	Faculty of Medicine, University of Coimbra, Portugal	jdasilva@ci.uc.pt
	António Tirado	Portuguese Society of Osteoporosis and Metabolic Bone Diseases (SPODOM), Lisbon, Portugal.	tirado.antonio2@icloud.com
	Ana Paula Barbosa	Portuguese Society of Osteoporosis and Metabolic Bone Diseases (SPODOM), Lisbon, Portugal.	apgsb1@gmail.com
	Ana Rodrigues	Portuguese Society of Osteoporosis and Metabolic Bone Diseases (SPODOM), Lisbon, Portugal.	anamfrodrigues@gmail.com
	Ana Pires Gonçalves	Portuguese Society of Osteoporosis and Metabolic Bone Diseases (SPODOM), Lisbon, Portugal.	aa.pgoncalves@gmail.com
Romania	Andrea Ildiko Gasparik	Department of Public Health and Health Management, University of Medicine and Pharmacy of Tirgu Mures, Tirgu Mures, Romania.	ildikogasparik@gmail.com
	Ionela Pascanu	Department of Endocrinology, University of Medicine and Pharmacy, Science and Technology (UMFST) G.E. Palade of Tg. Mures, Romania	iopascanu@gmail.com
	Daniel Grigorie	National Institute of Endocrinology, Carol Davila University of Medicine, Bucharest, Romania.	grigorie_d@yahoo.com
Slovakia	Juraj Payer	Comenius University Faculty of Medicine in Bratislava, 5. Department of Internal Medicine, University Hospital Bratislava, Bratislava, Slovakia	payer@ruzinov.fnspba.sk
	Pavol Masarky	National Institute of Rheumatology, Piešťany, Slovakia	pavol.masaryk@nurch.sk
	Peter Jackuliak	Comenius University Faculty of Medicine in Bratislava, 5. Department of Internal Medicine, University Hospital Bratislava, Bratislava, Slovakia	peter.jackuliak@fmed.uniba.sk
Slovenia	Tomaz Kocjan	Department of Endocrinology, Diabetes, and Metabolic Diseases, University Medical Centre Ljubljana, Ljubljana, Slovenia	tomaz.kocjan@kclj.si
		Faculty of Medicine, University of Ljubljana, Ljubljana, Slovenia	
Spain	Santiago Palacios	Palacios Institute of Women's Health, Madrid, Spain.	spalacios@institutopalacios.com
	Manuel Naves-Díaz	Bone and Mineral Research Unit, Hospital Universitario Central de Asturias, Instituto de Investigación Sanitaria del Principado de Asturias (ISPA), Retic REDinREN-ISCIII, Oviedo, Spain.	mnaves.huca@gmail.com
	Adolfo Diez-Perez	Department of Internal Medicine, Hospital del Mar/IMIM and CIBERFES, Autonomous University of Barcelona, , Barcelona, Spain.	ADiez@parcdesalutmar.cat.
Sweden	Kristina E Åkesson	Department of Clinical Sciences, Clinical and Molecular Osteoporosis Research Unit Malmö, Lund University, Lund, Sweden.	kristina.akesson@med.lu.se
		Department of Orthopaedics, Skåne University Hospital, Malmö, Sweden.	
	Bo Freyschuss	Department of Medicine, Karolinska Institutet, Stockholm, Sweden	bo.freyschuss@ki.se
Switzerland	Serge Ferrari	Service and Laboratory of Bone Diseases, Geneva University Hospital and Faculty of Medicine, Geneva, Switzerland.	Serge.Ferrari@unige.ch
	Rene Rizzoli	University Hospitals and Faculty of Medicine of Geneva, Geneva, Switzerland	Rene.Rizzoli@unige.ch
United Kingdom	M Kassim Javaid	Nuffield Department of Orthopaedics, Rheumatology and Musculoskeletal Sciences, University of Oxford, Oxford, UK.	kassim.javaid@ndorms.ox.ac.uk
	Craig Jones	Royal Osteoporosis Society, Bath, UK	Craig.Jones@theros.org.uk
	Cyrus Cooper	MRC Lifecourse Epidemiology Unit, Southampton General Hospital, University of Southampton, Southampton, UK.	cc@mrc.soton.ac.uk
IOF	Philippe Halbout	International Osteoporosis Foundation, Nyon, Switzerland	phalbout@iofbonehealth.org

**Table of Contents**

**Table:** 

		Page
Introduction		5
Epidemiology and economic burden of osteoporosis in		
1.	Austria	6
2.	Belgium	11
3.	Bulgaria	15
4.	Croatia	19
5.	Cyprus	23
6.	Czech Republic	27
7.	Denmark	32
8.	Estonia	37
9.	Finland	42
10.	France	46
11.	Germany	50
12.	Greece	54
13.	Hungary	58
14.	Ireland	62
15.	Italy	66
16.	Latvia	71
17.	Lithuania	75
18.	Luxembourg	79
19.	Malta	83
20.	Netherlands	87
21.	Poland	91
22.	Portugal	95
23.	Romania	99
24.	Slovakia	104
25.	Slovenia	108
26.	Spain	112
27.	Sweden	116
28.	Switzerland	120
29.	United Kingdom	125

## Introduction

Osteoporosis, literally “porous bone,” is a disease characterized by weak bone. It is a major public health problem, affecting hundreds of millions of people worldwide, predominantly postmenopausal women. The main clinical consequence of the disease is bone fractures. It is estimated that one in three women and one in five men over the age of fifty worldwide will sustain an osteoporotic fracture. Hip and spine fractures are the two most serious fracture types, associated with substantial pain and suffering, disability, and even death. As a result, osteoporosis imposes a significant burden on both the individual and society. Over the past three decades, a range of medications has become available for the treatment and prevention of osteoporosis. The primary aim of pharmacological therapy is to reduce the risk of osteoporotic fractures.

A recent report “SCOPE 2021: a new scorecard for osteoporosis in Europe” describes the current burden of osteoporosis in the EU in 2019 [1]. In 2019, 25.5 million women and 6.5 million men were estimated to have osteoporosis in the European Union plus Switzerland and the United Kingdom; and 4.3 million new fragility fractures were sustained, comprising 827,000 hip fractures, 663,000 vertebral fractures, 637,000 forearm fractures and 2,150,000 other fractures (i.e., fractures of the pelvis, rib, humerus, tibia, fibula, clavicle, scapula, sternum, and other femoral fractures). The economic burden of incident and prior fragility fractures in 2019 was estimated at € 57 billion. In the EU27+2, there were estimated to be 248,487 causally related deaths in 2019. The number of fracture-related deaths are comparable to or exceed some of the most common causes of death such as lung cancer, diabetes, chronic lower respiratory diseases. The population age 50 years or more is projected to increase by 11.4% in men and women between 2019 and 2034 and the annual number of osteoporotic fractures in the EU27+2 will increase by 25%. The majority of individuals who have sustained an osteoporosis-related fracture or who are at high risk of fracture are untreated and the proportion of high risk patients on treatment is declining.

The objective of this report is to review and describe the current burden of osteoporosis in each of the EU member states plus Switzerland and the UK. Epidemiological and health economic aspects of osteoporosis and osteoporotic fractures are summarised for 2019 with projections of the future prevalence of osteoporosis, the number of incident fractures, the direct and total cost of the disease including the value of QALYs lost. The report also provides information on the policy framework together with service provision and service uptake within each country. The report may serve as a basis for the formulation of healthcare policy concerning osteoporosis in general and the treatment and prevention of osteoporosis in particular. It may also provide guidance regarding the overall healthcare priority of the disease in each member state.

## References

1. Kanis JA, Norton N, Harvey NC, Jacobson T, Johansson H Lorentzon M, McCloskey EV, Willers C, Borgström F (2021) SCOPE 2021: a new scorecard for osteoporosis in Europe. Arch Osteoporos 16: 82. 10.1007/s11657-020-00871-9

## Epidemiology and economic burden of osteoporosis in Austria

HP Dimai ∙ C Muschitz ∙ C Willers ∙ N Norton ∙ NC Harvey ∙ T Jacobson ∙ H Johansson ∙ M Lorentzon ∙ EV McCloskey ∙ F Borgström ∙ JA Kanis

**Introduction**

The scorecard summarises key indicators of the burden of osteoporosis and its management in the 27 member states of the European Union, as well as the UK and Switzerland (termed EU27+2) [1]. This country-specific report summarises the principal results for Austria.

**Methods**

The information obtained covers four domains: burden of osteoporosis and fractures; policy framework; service provision; and service uptake. Data were collected from numerous sources including previous research and IOF reports, and available registers which were used for additional analysis of resource utilization, costing and HRQoL data. Furthermore, country-specific information on osteoporosis management was obtained from each IOF member state via a questionnaire.

**Burden of disease**

The direct cost of incident fractures in Austria in 2019 was €833.5 million. Added to this was the ongoing cost in 2019 from fractures that occurred before 2019, which amounted to €468.1 million (long-term disability). The cost of pharmacological intervention (assessment and treatment) was €41.7 million. Thus, the total direct cost (excluding the value of QALYs lost) amounted to €1.3 billion in 2019. Key metrics are presented in Table 1.

In 2019, the average direct cost of osteoporotic fractures in Austria was €151.8 per individual in the population, while in 2010 the average was €104.8 (after adjusting for inflation) representing a relative increase of 45% (€151.8 versus €104.8). The 2019 numbers put Austria in the 6th place in terms of highest cost of osteoporotic fractures per capita in the EU27+2.

The cost of osteoporotic fractures in Austria accounted for approximately 3.4% of healthcare spending (i.e. €1.3 billion out of €38.7 billion in 2019), close to the EU27+2 average of 3.5% placing Austria at 13th in the ranked order across the EU27+2 countries. These numbers indicate a substantial impact of fragility fractures on the healthcare budget.

Using World Health Organization diagnostic criteria for osteoporosis based on the measurement of bone mineral density (BMD) [2], there were approximately 552,000 individuals with osteoporosis in Austria in 2019, of whom almost 80% were women. The prevalence of osteoporosis in the total Austrian population amounted to 5.5%, on par with the EU27+2 average (5.6%).

**Table 1** Key measures of burden of disease for Austria

**Table:** 

**Category**	**Measure**	**Estimate**	**Rank (EU27+2)**
Burden of disease	Direct cost of incident fracture (€m)	833.5	
	Long-term disability cost (€m)	468.1	
	Intervention cost (€m)	41.7	
	Total cost (€m)	1 343	
	QALYs lost (€m)	4 111	
	Cost per capita (€)	151.8	6
	Proportion of healthcare spending	3.4%	13
	Prevalence of osteoporosis	5.5%	12

There were estimated to be 110,000 new fragility fractures in Austria in 2019, equivalent to 300 fractures/day (or 12 per hour). This was a slight increase compared to 2010, equivalent to an increment of 1.1 fractures/1000 individuals, totalling 29.6 fractures/ 1000 individuals in 2019.

Some osteoporotic fractures are associated with premature mortality [3]. In Austria, the annual number of deaths associated with a fracture event was estimated to be 165 per 100,000 individuals of the population aged 50 years or more, compared to the EU27+2 average of 116/100,000. The number of fracture-related deaths is comparable to or exceeds that for some of the most common causes of death such as lung cancer, diabetes, chronic lower respiratory diseases.

The remaining lifetime probability of hip fracture (%) at the ages of 50 years in men and women was 8.3% and 19.7% respectively, placing Austria in the upper tertile of risk for both men and women.

The population of men and women age 50 years or more is projected to increase by 11.8% in men and women between 2019 and 2034, close to the EU27+2 average of 11.4%. The increases in men and women aged 75 years or more are even more marked and amount to 38.0% and 22.0%, respectively. The annual number of osteoporotic fractures in Austria is expected to increase by 30,000 to 140,000 in 2034.

**Policy framework** (Table 2)

Documentation of the burden of disease is an essential prerequisite to determine the resources that should be allocated to the diagnosis and treatment of the disorder. High quality national data on hip fracture rates have been identified in 18 of 29 countries, of which Austria is one. Data are collected on a national basis and include more than only hip fracture data.

Given that osteoporosis and fragility fractures are common and that effective treatments are widely available, the vast majority of patients with osteoporosis are preferably managed at the primary health care level by general practitioners (GPs), with specialist referral reserved for difficult complex cases. Primary care was the principal provider of the medical care for osteoporosis in Austria, as for 13 of the 28 countries where data were available.

Osteoporosis and metabolic bone disease is not a recognised specialty in most countries including Austria. Specialty care of osteoporosis in Austria is via other specialties including endocrinology, gynaecology, orthopaedic surgery and rheumatology. Osteoporosis is however recognized as a component of specialty training. Although it is possible that these specialties educate their trainees adequately, the wide variation may reflect inconsistencies in patient care, training of primary care physicians and a suboptimal voice to “defend” the interests of those who work within the field of osteoporosis.

**Table 2** Policy framework for osteoporosis in Austria

**Table:** 

**Category**	**Measure**	**Estimate**
Policy framework	National fracture data availability	Yes
	OP recognized as a specialty	No
	OP primarily managed in primary care	Yes
	Other specialties involved	Endocrinology, Rheumatology, Gynaecology, Orthopaedics
	Advocacy areas covered by patient organisation	Policy, capacity, peer support, research and development

The role of national patient organisations is to improve the care of patients and increase awareness and prevention of osteoporosis and related fractures among the general public. Advocacy by patient organisations can fall into four categories: policy, capacity building and education, peer support, research and development. For Austria, all four of the advocacy areas were covered by a patient organisation, which was the case for only 10 out of the 26 countries with at least one patient organisation.

**Service provision** (Table 3)

A wide variety of approved drug treatments is available for the management of osteoporosis [4]. Potential limitations of their use in member states relate to reimbursement policies which may impair the delivery of health care. Austria is one of the 12 (out of 27) countries that offer full reimbursement.

The assessment of bone mineral density forms a key component for the general management of osteoporosis, being used for diagnosis, risk prediction, selection of patients for treatment and monitoring of patients on treatment. In Austria, the number of DXA units expressed per million of the general population amounted to 29.7 which puts the country in the 3rd place among the EU27+2. Furthermore, the availability of TBS was highest in Austria.

The average waiting time for DXA ranged from 0 to 180 days across countries, and there was no clear relation between waiting times and the availability of DXA. In Austria, the estimated average waiting time for DXA amounted to 14 days. Nine countries reported shorter average waiting times.

**Table 3** Service provision for osteoporosis in Austria

**Table:** 

**Category**	**Measure**	**Estimate**	**Rank (EU27+2)**
Service provision	Reimbursement of OP medications	100%	
	DXA units/million inhabitants	29.7	3
	DXA cost (€)	50	12
	FRAX risk assessment model available	Yes	
	Fracture liaison service density	1-10%	

Reimbursement for DXA scans varied between member states both in terms of the criteria required and level of reimbursement awarded. In Austria, the reimbursement was conditional and varied depending on public versus private delivery of the service.

The effective targeting of treatment to those at highest risk of fracture requires an assessment of fracture risk. Risk assessment models for fractures, most usually based on FRAX, were available in 24 out of 29 countries, of which Austria was one. An additional risk assessment model, DVO, was also used in Austria. For Austria, guidance on the use of risk assessment within national guidelines was available, as in only 14 of the other countries.

Guidelines for the management of osteoporosis were available in Austria (as in 27 out of 29 countries). The guidelines in Austria included postmenopausal women specifically, as well as for osteoporosis in men and for secondary osteoporosis including glucocorticoid-induced osteoporosis.

Fracture liaison services (FLS), also known as osteoporosis coordinator programmes and care manager programmes, provide a system for the routine assessment and management of postmenopausal women and older men who have sustained a low trauma fracture. Fracture liaison services were reported for 1–10% of the hospitals in Austria.

The use of indicators to systematically measure the quality of care provided to people with osteoporosis or associated fractures has expanded as a discipline within the past decade [5]. No use of national quality indicators was reported for Austria.

**Service uptake** (Table 4)

The web-based usage of FRAX showed considerable heterogeneity in uptake between the countries. The average uptake for the EU27+2 was 1,555 sessions/million/year of the general population with an enormous range of 49 to 41,874 sessions/million. The usage for Austria amounted to 2,439 sessions/million in 2019, with a 59% increase since 2011.

Many studies have demonstrated that a significant proportion of men and women at high fracture risk do not receive therapy for osteoporosis (the treatment gap) [6]. In the EU27+2 the average treatment gap was 71% but ranged from 32 to 87%. For Austria, the treatment gap amongst women amounted to 52% or 168,000 out of 325,000 characterised at risk. The Austrian treatment gap did not change significantly compared to 2010, whilst the average treatment gap among EU27+2 increased from 55% in 2010 to 71% in 2019. In recent nationwide study, the treatment gap 4, 12 and 18 months after the first hip fracture was 82 %, 84 % and 85 % in women, and 92 %, 88 % and 90 % in men, respectively [7].

**Table 4** Service uptake for osteoporosis in Austria

**Table:** 

**Category**	**Measure**	**Estimate**	**Rank (EU27+2)**
Service uptake	Number of FRAX sessions/million people/year	2439	10
	Treatment gap for women eligible for treatment (%)	52	4
	Proportion surgically managed hip fractures	>90%	

About 5% of people with a hip fracture die within 1 month of their fracture [8]. A determinant of peri-operative morbidity and mortality is the time a patient takes to get to surgery [9]. For Austria, the average waiting time for hip fracture surgery after hospital admission was reported to be less than 24 h, implying a reduction in waiting time compared to 2010 (waiting time of 1–2 days). The proportion of surgically managed hip fractures was reported to be over 90%.

**Scores and scorecard**

Scores were developed for Burden of disease and the healthcare provision (Policy framework, Service provision and Service uptake) in the EU27+2 countries. Austria scores resulted in a 4th place regarding Burden of disease after only Denmark, Sweden and Switzerland. The combined healthcare provision scorecard resulted in a 7th place for Austria. Thus, Austria presents as one of the eight high-burden high-provision countries among the EU27+2.

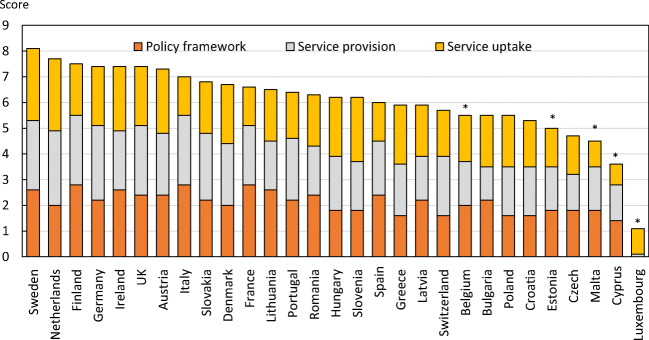



**Fig. 1** Scores by country for metrics related to policy framework, service provision and service uptake. The mean score for each of the 3 domains is given. An asterisk denotes that there was one or more missing metric which decreases the overall score

The first SCOPE was undertaken in 2010, almost 10 years previously. Fifteen of the 16 score card metrics on healthcare provision were used in the two surveys. Scores had improved or markedly improved in 15 countries, remained constant in 8 countries and worsened in 3 countries. For Austria the scores were somewhat improved.

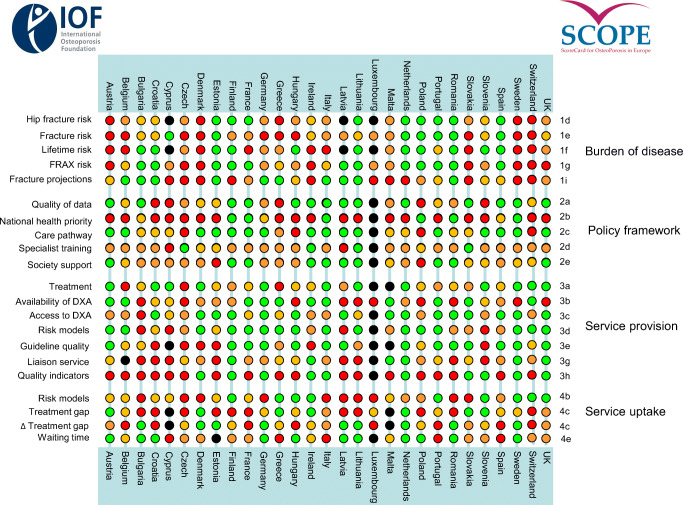



**Fig. 2** The scorecard for all the EU27+2 countries illustrating the scores across the four domains. The elements of each domain in each country were scored and coded using a traffic light system (red, orange, green). Black dots signify missing information

The second edition of the Scorecard for Osteoporosis in Europe (SCOPE 2021) allows health and policy professionals to assess key indicators on the healthcare provision for osteoporosis within countries and between counties within the EU 27+2. The scorecard is not intended as a prescriptive template. Thus, it does not set performance targets but may serve as a guide to the performance targets at which to aim in order to deliver the outcomes required.

**Acknowledgements**

SCOPE was supported by an unrestricted grant from Amgen to the International Osteoporosis Foundation (IOF). Amgen was neither involved in the design nor writing of the report. We are grateful to Anastasia Soulié Mlotek and Dominique Pierroz of the IOF for their help in the administration of SCOPE. The report has been reviewed by the members of the SCOPE Consultation Panel and the relevant IOF National societies, and we are grateful for their local insights on the management of osteoporosis in each country. The source document has been reviewed and endorsed by the Committee of Scientific Advisors of the IOF and benefitted from their feedback.

**References**

1. Kanis JA, Norton N, Harvey NC, Jacobson T, Johansson H, Lorentzon M, McCloskey EV, Willers C, Borgström F (2021) SCOPE 2021: a new scorecard for osteoporosis in Europe. Arch Osteoporos  16:82. 10.1007/s11657-020-00871-9

2. World Health Organisation (1994) Assessment of fracture risk and its application to screening for postmenopausal osteoporosis. Report of a WHO Study Group. World Health Organ Tech Rep Ser, 1994/01/01 edn, pp 1-129

3. Johnell O, Kanis JA, Oden A, Sernbo I, Redlund-Johnell I, Petterson C, De Laet C, Jonsson B (2004) Mortality after osteoporotic fractures. Osteoporos Int 15:38-42

4. Hernlund E, Svedbom A, Ivergard M, Compston J, Cooper C, Stenmark J, McCloskey EV, Jonsson B, Kanis JA (2013) Osteoporosis in the European Union: medical management, epidemiology and economic burden. A report prepared in collaboration with the International Osteoporosis Foundation (IOF) and the European Federation of Pharmaceutical Industry Associations (EFPIA). Arch Osteoporos 8:136

5. Allen P, Pilar M, Walsh-Bailey C, Hooley C, Mazzucca S, Lewis CC, Mettert KD, Dorsey CN, Purtle J, Kepper MM, Baumann AA, Brownson RC (2020) Quantitative measures of health policy implementation determinants and outcomes: a systematic review. Implement Sci 15:47

6. Borgstrom F, Karlsson L, Ortsater G, Norton N, Halbout P, Cooper C, Lorentzon M, McCloskey EV, Harvey NC, Javaid MK, Kanis JA (2020) Fragility fractures in Europe: burden, management and opportunities. Arch Osteoporos 15:59

7. Malle O, Borgstrom F, Fahrleitner-Pammer A, Svedbom A, Dimai SV, Dimai HP (2021) Mind the gap: Incidence of osteoporosis treatment after an osteoporotic fracture - results of the Austrian branch of the International Costs and Utilities Related to Osteoporotic Fractures Study (ICUROS). Bone 142:115071. doi: 10.1016/j.bone.2019.115071.

8. Kanis JA, Oden A, Johnell O, De Laet C, Jonsson B, Oglesby AK (2003) The components of excess mortality after hip fracture. Bone 32:468-473

9. National Clinical Guideline Centre (2011) The Management of Hip Fracture in Adults. In Centre NCG (ed)London

## Epidemiology and economic burden of osteoporosis in Belgium

JF Kaux ∙ J-Y Reginster ∙ O Bruyère ∙ E Cavalier ∙ M-P Lecart ∙ C Willers ∙ N Norton ∙ NC Harvey ∙ T Jacobson ∙ H Johansson ∙ M Lorentzon ∙ EV McCloskey ∙ F Borgström ∙ JA Kanis

**Introduction**

The scorecard summarises key indicators of the burden of osteoporosis and its management in the 27 member states of the European Union, as well as the UK and Switzerland (termed EU27+2) [1]. This country-specific report summarises the principal results for Belgium.

**Methods**

The information obtained covers four domains: burden of osteoporosis and fractures; policy framework; service provision; and service uptake. Data were collected from numerous sources including previous research and IOF reports, and available registers which were used for additional analysis of resource utilization, costing and HRQoL data. Furthermore, country-specific information on osteoporosis management was obtained from each IOF member state via a questionnaire.

**Burden of disease**

The direct cost of incident fractures in Belgium in 2019 was €766.4 million. Added to this was the ongoing cost in 2019 from fractures that occurred before 2019, which amounted to €321.9 million (long-term disability). The cost of pharmacological intervention (assessment and treatment) was €34.0 million. Thus, the total direct cost (excluding the value of QALYs lost) amounted to €1.1 billion in 2019. Key metrics are presented in Table 1.

In 2019, the average direct cost of osteoporotic fractures in Belgium was €98.3 per individual in the population, while in 2010 the average was €62.9 (after adjusting for inflation), representing a relative increase of 56% (€98.3 versus €62.9). The 2019 numbers put Belgium in 9th place in terms of highest cost of osteoporotic fractures per capita in the EU27+2.

The cost of osteoporotic fractures in Belgium accounted for approximately 2.4% of healthcare spending (i.e. €1.1 billion out of €45.7 billion in 2019), lower than the EU27+2 average of 3.5% and placed Belgium at 23rd in the ranked order across the EU27+2 countries.

Using World Health Organization diagnostic criteria for osteoporosis based on the measurement of bone mineral density (BMD) [2], there were approximately 681,000 individuals with osteoporosis in Belgium in 2019, of whom almost 80% were women. The prevalence of osteoporosis in the total Belgian population amounted to 5.6%, on par with the EU27+2 average (5.6%).

**Table 1** Key measures of burden of disease for Belgium

**Table:** 

**Category**	**Measure**	**Estimate**	**Rank**
Burden of disease	Direct cost of incident fracture (€m)	766.36	
	Long-term disability cost (€m)	321.85	
	Intervention cost (€m)	33.97	
	Total cost (€m)	1122.18	
	QALYs lost (€m)	3 079	
	Cost per capita (€)	98.25	9
	Proportion of healthcare spending	2.4%	23
	Prevalence of osteoporosis	5.6%	8

There were estimated to be 100,000 new fragility fractures in Belgium in 2019, equivalent to 274 fractures/day (or 11 per hour). This was a slight increase compared to 2010, equivalent to an increment of 1.8 fractures/1000 individuals, totalling 22 fractures/ 1000 individuals in 2019.

Some osteoporotic fractures are associated with premature mortality [3]. In Belgium, the annual number of deaths associated with a fracture event was estimated to be 119 per 100,000 individuals of the population aged 50 years or more, compared to the EU27+2 average of 116/100,000. The number of fracture-related deaths is comparable to or exceeds that for some of the most common causes of death such as lung cancer, diabetes, chronic lower respiratory diseases.

The remaining lifetime probability of hip fracture (%) at the ages of 50 years in men and women was 7.8% and 18.2%, respectively, placing Belgium in the upper tertile of risk for both men and women.

The population of men and women age 50 years or more is projected to increase by 12.6% between 2019 and 2034, close to the EU27+2 average of 11.4%. The increases in men and women aged 75 years or more are even more marked and amount to 55.5% and 32.8%, respectively. The annual number of osteoporotic fractures in Belgium is expected to increase by 23,000 to 123,000 in 2034.

**Policy framework** (Table 2)

Documentation of the burden of disease is an essential prerequisite to determine the resources that should be allocated to the diagnosis and treatment of the disorder. High quality national data on hip fracture rates have been identified in 18 of 29 countries, of which Belgium is one. Data are collected on a national basis and include more than only hip fracture data.

Given that osteoporosis and fragility fractures are common and that effective treatments are widely available, the vast majority of patients with osteoporosis are preferably managed at the primary health care level by general practitioners (GPs), with specialist referral reserved for difficult complex cases. Primary care was the principal provider of the medical care for osteoporosis in Belgium, as for 13 of the 28 countries where data were available.

Osteoporosis and metabolic bone disease is not a recognised specialty in most countries including Belgium. Specialty care of osteoporosis in Belgium is via other specialties including rehabilitation medicine. Osteoporosis is however recognized as a component of specialty training. Although it is possible that trainees are educated adequately, the wide variation may reflect inconsistencies in patient care, training of primary care physicians and a suboptimal voice to “defend” the interests of those who work within the field of osteoporosis.

**Table 2** Policy framework for osteoporosis in Belgium

**Table:** 

**Category**	**Measure**	**Estimate**
Policy framework	National fracture data availability	No
	OP recognized as a specialty	No
	OP primarily managed in primary care	Yes
	Other specialties involved	Rehabilitation medicine
	Advocacy areas covered by patient organisation	None

The role of national patient organisations is to improve the care of patients and increase awareness and prevention of osteoporosis and related fractures among the general public. Advocacy by patient organisations can fall into four categories: policy, capacity building and education, peer support, research and development. For Belgium, none of the advocacy areas were covered by a patient organisation. For 10 out of the 26 countries with at least one patient organisation, all advocacy areas were covered.

**Service provision** (Table 3)

A wide variety of approved drug treatments is available for the management of osteoporosis [4]. Potential limitations of their use in member states relate to reimbursement policies which may impair the delivery of health care. 12 (out of 27) countries offer full reimbursement, Belgium is not one of them.

The assessment of bone mineral density forms a key component for the general management of osteoporosis, being used for diagnosis, risk prediction, selection of patients for treatment and monitoring of patients on treatment. In Belgium, the number of DXA units expressed per million of the general population amounted to 28.9 which puts the country in the 4th place among the EU27+2.

The average waiting time for DXA ranged from 0 to 180 days across countries, and there was no clear relation between waiting times and the availability of DXA. In Belgium, the estimated average waiting time for DXA amounted to 7 days. Only four countries reported shorter average waiting times.

**Table 3** Service provision for osteoporosis in Belgium

**Table:** 

**Category**	**Measure**	**Estimate**	**Rank**
Service provision	Reimbursement of OP medications	61-98%	
	DXA units/million inhabitants	28,9	4
	DXA cost (€)	93	5
	FRAX risk assessment model available	Yes	
	Fracture liaison service density	N/A	

Reimbursement for DXA scans varied between member states both in terms of the criteria required and level of reimbursement awarded. In Belgium, the reimbursement was conditional.

The effective targeting of treatment to those at highest risk of fracture requires an assessment of fracture risk. Risk assessment models for fractures, most usually based on FRAX, were available in 24 out of 29 countries, of which Belgium was one. For Belgium, guidance on the use of risk assessment within national guidelines was available, as in only 14 of the other countries.

Guidelines for the management of osteoporosis were available in Belgium (as in 27 out of 29 countries). The guidelines in Belgium included postmenopausal women specifically, as well as for osteoporosis in men and for secondary osteoporosis including glucocorticoid-induced osteoporosis.

Fracture liaison services (FLS), also known as osteoporosis coordinator programmes and care manager programmes, provide a system for the routine assessment and management of postmenopausal women and older men who have sustained a low trauma fracture. No information on fracture liaison services was reported for Belgium.

The use of indicators to systematically measure the quality of care provided to people with osteoporosis or associated fractures has expanded as a discipline within the past decade [5]. No use of national quality indicators was reported for Belgium.

**Service uptake** (Table 4)

The web-based usage of FRAX showed considerable heterogeneity in uptake between the countries. The average uptake for the EU27+2 was 1,555 sessions/million/year of the general population with an enormous range of 49 to 41,874 sessions/million. The usage for Belgium amounted to 2,144 sessions/million in 2019, with a 57 percent decrease since 2011.

Many studies have demonstrated that a significant proportion of men and women at high fracture risk do not receive therapy for osteoporosis (the treatment gap) [6]. In the EU27+2 the average treatment gap was 71% but ranged from 32 to 87%. For Belgium, the treatment gap amongst women amounted to 66% or 291,000 out of 441,000 characterised at risk. The Belgian treatment gap grew significantly compared to 2010, as did the treatment gap among EU27+2 which increased from 55% in 2010 to 71% in 2019.

**Table 4** Service uptake for osteoporosis in Belgium

**Table:** 

**Category**	**Measure**	**Estimate**	**Rank**
Service uptake	Number of FRAX sessions/million people/year	2144	11
	Treatment gap for women eligible for treatment (%)	66	10
	Proportion surgically managed hip fractures	>90%	

About 5% of people with a hip fracture die within 1 month of their fracture [7]. A determinant of peri-operative morbidity and mortality is the time a patient takes to get to surgery [8]. For Belgium, the average waiting time for hip fracture surgery after hospital admission was reported to be 1–2 days, implying an increase in waiting time compared to 2010 (waiting time of <24 h). The proportion of surgically managed hip fractures was reported to be over 90%.

**Scores and scorecard**

Scores were developed for Burden of disease and the healthcare provision (Policy framework, Service provision and Service uptake) in the EU27+2 countries. Belgium scores resulted in an 8th place regarding Burden of disease. The combined healthcare provision scorecard resulted in a 21st place for Belgium. Thus, Belgium presents as one of the eight high-burden low-provision countries among the EU27+2.

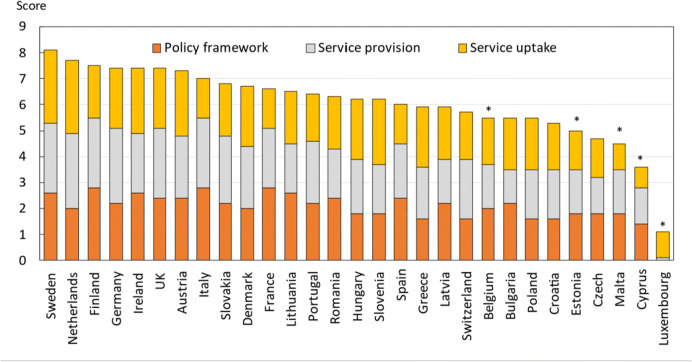



**Fig. 1** Scores by country for metrics related to policy framework, service provision and service uptake. The mean score for each of the 3 domains is given. An asterisk denotes that there was one or more missing metric which decreases the overall score

The first SCOPE was undertaken in 2010, almost 10 years previously. Fifteen of the 16 score card metrics on healthcare provision were used in the two surveys. Scores had improved or markedly improved in 15 countries, remained constant in 8 countries and worsened in 3 countries. For Belgium, the scores were worse in 2019 compared to 2010.

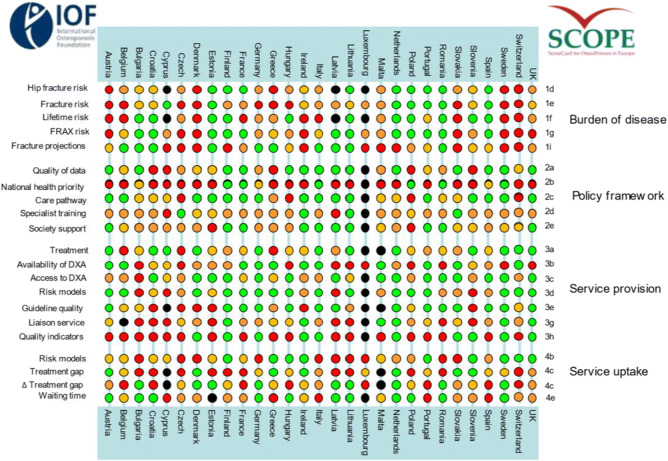



**Fig. 2** The scorecard for all the EU27+2 countries illustrating the scores across the four domains. The elements of each domain in each country were scored and coded using a traffic light system (red, orange, green). Black dots signify missing information

The second edition of the Scorecard for Osteoporosis in Europe (SCOPE 2021) allows health and policy professionals to assess key indicators on the healthcare provision for osteoporosis within countries and between countries within the EU 27+2. The scorecard is not intended as a prescriptive template. Thus, it does not set performance targets but may serve as a guide to the performance targets at which to aim in order to deliver the outcomes required.

**Acknowledgements**

SCOPE was supported by an unrestricted grant from Amgen to the International Osteoporosis Foundation (IOF). Amgen was neither involved in the design nor writing of the report. We are grateful to Anastasia Soulié Mlotek and Dominique Pierroz of the IOF for their help in the administration of SCOPE. We acknowledge the assistance of the Royal Belgian Society of Physical and Rehabilitation Medicine and the Belgian Bone Club. We thank Stefan Goemaere, Ghent University Hospital, Belgium for his helpful input.The report has been reviewed by the members of the SCOPE Consultation Panel and the relevant IOF National societies, and we are grateful for their local insights on the management of osteoporosis in each country. The source document has been reviewed and endorsed by the Committee of Scientific Advisors of the IOF and benefitted from their feedback.

**References**

1. Kanis JA, Norton N, Harvey NC, Jacobson T, Johansson H, Lorentzon M, McCloskey EV, Willers C, Borgström F (2021) SCOPE 2021: a new scorecard for osteoporosis in Europe. Arch Osteoporos  16:82. 10.1007/s11657-020-00871-9

2. World Health Organisation (1994) Assessment of fracture risk and its application to screening for postmenopausal osteoporosis. Report of a WHO Study Group. World Health Organ Tech Rep Ser, 1994/01/01 edn, pp 1-129

3. Johnell O, Kanis JA, Oden A, Sernbo I, Redlund-Johnell I, Petterson C, De Laet C, Jonsson B (2004) Mortality after osteoporotic fractures. Osteoporos Int 15:38-42

4. Hernlund E, Svedbom A, Ivergard M, Compston J, Cooper C, Stenmark J, McCloskey EV, Jonsson B, Kanis JA (2013) Osteoporosis in the European Union: medical management, epidemiology and economic burden. A report prepared in collaboration with the International Osteoporosis Foundation (IOF) and the European Federation of Pharmaceutical Industry Associations (EFPIA). Arch Osteoporos 8:136

5. Allen P, Pilar M, Walsh-Bailey C, Hooley C, Mazzucca S, Lewis CC, Mettert KD, Dorsey CN, Purtle J, Kepper MM, Baumann AA, Brownson RC (2020) Quantitative measures of health policy implementation determinants and outcomes: a systematic review. Implement Sci 15:47

6. Borgstrom F, Karlsson L, Ortsater G, Norton N, Halbout P, Cooper C, Lorentzon M, McCloskey EV, Harvey NC, Javaid MK, Kanis JA (2020) Fragility fractures in Europe: burden, management and opportunities. Arch Osteoporos 15:59

7. Kanis JA, Oden A, Johnell O, De Laet C, Jonsson B, Oglesby AK (2003) The components of excess mortality after hip fracture. Bone 32:468-473

8. National Clinical Guideline Centre (2011) The Management of Hip Fracture in Adults. In Centre NCG (ed)London

## Epidemiology and economic burden of osteoporosis in Bulgaria

A-M Borissova ∙ M Boyanov ∙ Z Kolarov ∙ C Willers ∙ N Norton ∙ NC Harvey ∙ T Jacobson ∙ H Johansson ∙ M Lorentzon ∙ EV McCloskey ∙ F Borgström ∙ JA Kanis

**Introduction**

The scorecard summarises key indicators of the burden of osteoporosis and its management in the 27 member states of the European Union, as well as the UK and Switzerland (termed EU27+2) [1]. This country-specific report summarises the principal results for Bulgaria.

**Methods**

The information obtained covers four domains: burden of osteoporosis and fractures; policy framework; service provision; and service uptake. Data were collected from numerous sources including previous research and IOF reports, and available registers which were used for additional analysis of resource utilization, costing and HRQoL data. Furthermore, country-specific information on osteoporosis management was obtained from each IOF member state via a questionnaire.

**Burden of disease**

The direct cost of incident fractures in Bulgaria in 2019 was €135.1 million. Added to this was the ongoing cost in 2019 from fractures that occurred before 2019, which amounted to €41.3 million (long-term disability). The cost of pharmacological intervention (assessment and treatment) was €9.2 million. Thus, the total direct cost (excluding the value of QALYs lost) amounted to €186 million in 2019. Key metrics are presented in Table 1.

In 2019, the average direct cost of osteoporotic fractures in Bulgaria was €26.4 per individual in the population, while in 2010 the average was €6.6 (after adjusting for inflation), representing a relative increase of 299% (€26.4 versus €6.6) The 2019 numbers put Bulgaria in the 25th place in terms of cost of osteoporotic fractures per capita in the EU27+2.

The cost of osteoporotic fractures in Bulgaria accounted for approximately 4.2% of healthcare spending (i.e. €186 million out of €4.2 billion in 2019), somewhat higher than the EU27+2 average of 3.5% and placed Bulgaria at the 9th place in the rank order of the EU27+2 countries. These numbers indicate a substantial impact of fragility fractures on the healthcare budget.

Using World Health Organization diagnostic criteria for osteoporosis based on the measurement of bone mineral density (BMD) [2], there were approximately 420,000 individuals with osteoporosis in Bulgaria in 2019, of whom approximately 80% were women. The prevalence of osteoporosis in the total Bulgarian population amounted to 5.6%, on par with the EU27+2 average (5.6%).

**Table 1** Key measures of burden of disease for Bulgaria

**Table:** 

**Category**	**Measure**	**Estimate**	**Rank**
Burden of disease	Direct cost of incident fracture (€m)	135.09	
	Long-term disability cost (€m)	41.30	
	Intervention cost (€m)	9.19	
	Total cost (€m)	185.58	
	QALYs lost (€m)	327	
	Cost per capita (€)	26.42	25
	Proportion of healthcare spending	4.2%	9
	Prevalence of osteoporosis	5.6%	9

There were estimated to be 56,000 new fragility fractures in Bulgaria in 2019, equivalent to 150 fractures/day (or 6.4 per hour). This was a significant increase compared to 2010, equivalent to an increment of 6.0 fractures/1000 individuals, totalling 19.3 fractures/ 1000 individuals in 2019.

Some osteoporotic fractures are associated with premature mortality [3]. In Bulgaria, the annual number of deaths associated with a fracture event was estimated to be 184 per 100,000 individuals of the population aged 50 years or more, compared to the EU27+2 average of 116/100,000. The number of fracture-related deaths is comparable to or exceeds that for some of the most common causes of death such as lung cancer, diabetes, chronic lower respiratory diseases.

The remaining lifetime probability of hip fracture (%) at the ages of 50 years in men and women was 4.4% and 11.2%, respectively [4], placing Bulgaria in the lower tertile of risk for both men and women.

The Bulgarian population of men and women age 50 years or more is projected to decrease by 0.1% between 2019 and 2034, compared to the EU27+2 average of an increase with 11.4%. The number of men and women aged 75 years in Bulgaria are however projected to increase with 20.1% and 19.7%, respectively. The annual number of osteoporotic fractures in Bulgaria is expected to increase by 5,000 to 61,000 in 2034.

**Policy framework** (Table 2)

Documentation of the burden of disease is an essential prerequisite to determine the resources that should be allocated to the diagnosis and treatment of the disorder. High quality national data on hip fracture rates have been identified in 18 of 29 countries, of which Bulgaria is one. Data are collected on a national basis and include more than only hip fracture data.

Given that osteoporosis and fragility fractures are common and that effective treatments are widely available, the vast majority of patients with osteoporosis are preferably managed at the primary health care level by general practitioners (GPs), with specialist referral reserved for difficult complex cases. Primary care was the principal provider of the medical care for osteoporosis in 13 of the 28 countries where data were available. In Bulgaria, the lead specialty for osteoporosis was reported to be rheumatology.

Osteoporosis and metabolic bone disease is not a recognised specialty in most countries including Bulgaria. Specialty care of osteoporosis in Bulgaria is managed via specialties including rheumatology, endocrinology, internal medicine and orthopaedics. Osteoporosis is however recognized as a component of specialty training. Although it is possible that these specialties educate their trainees adequately, the wide variation may reflect inconsistencies in patient care, training of primary care physicians and a suboptimal voice to “defend” the interests of those who work within the field of osteoporosis.

**Table 2** Policy framework for osteoporosis in Bulgaria

**Table:** 

**Category**	**Measure**	**Estimate**
Policy framework	National fracture data availability	Yes
	OP recognized as a specialty	No
	OP primarily managed in primary care	No
	Other specialties involved	Rheumatology, Endocrinology. Internal medicine, Orthopaedics
	Advocacy areas covered by patient organisation	None

The role of national patient organisations is to improve the care of patients and increase awareness and prevention of osteoporosis and related fractures among the general public. Advocacy by patient organisations can fall into four categories: policy, capacity building and education, peer support, research and development. For Bulgaria, none of the advocacy areas were covered by a patient organisation, whilst 10 out of the 26 countries with at least one patient organisation had all four areas covered.

**Service provision** (Table 3)

A wide variety of approved drug treatments is available for the management of osteoporosis [5]. Potential limitations of their use in member states relate to reimbursement policies which may impair the delivery of health care. Twelve out of 27 countries offered full reimbursement, and Bulgaria belonged to the remaining 15 countries offering partial reimbursement.

The assessment of bone mineral density forms a key component for the general management of osteoporosis, being used for diagnosis, risk prediction, selection of patients for treatment and monitoring of patients on treatment. In Bulgaria, the number of DXA units expressed per million of the general population amounted to 3.6 which puts the country in the 28th place among the EU27+2.

The average waiting time for DXA ranged from 0 to 180 days across countries, and there was no clear relation between waiting times and the availability of DXA. In Bulgaria, the estimated average waiting time for DXA amounted to five days. Only three countries reported shorter average waiting times. Reimbursement for DXA scans varied between member states both in terms of the criteria required and level of reimbursement awarded.

**Table 3** Service provision for osteoporosis in Bulgaria

**Table:** 

**Category**	**Measure**	**Estimate**	**Rank**
Service provision	Reimbursement of OP medications	50%	
	DXA units/million inhabitants	3.6	28
	DXA cost (€)	50	14
	FRAX risk assessment model available	From 2020	
	Fracture liaison service density	No FLS	

The effective targeting of treatment to those at highest risk of fracture requires an assessment of fracture risk. Risk assessment models for fractures, most usually based on FRAX, were available in 24 out of 29 countries. Since this survey, Bulgaria has become the 25th country with a risk assessment model. For Bulgaria, guidance on the use of risk assessment within national guidelines was not yet available, as it was in 14 of the other countries.

Guidelines for the management of osteoporosis were available in Bulgaria (as in 27 out of 29 countries). The guidelines in Bulgaria included postmenopausal women specifically, as well as osteoporosis in men and secondary osteoporosis including glucocorticoid-induced osteoporosis.

Fracture liaison services (FLS), also known as osteoporosis coordinator programmes and care manager programmes, provide a system for the routine assessment and management of postmenopausal women and older men who have sustained a low trauma fracture. No fracture liaison services were reported from Bulgaria (together with seven other countries).

The use of indicators to systematically measure the quality of care provided to people with osteoporosis or associated fractures has expanded as a discipline within the past decade [6]. No use of national quality indicators was reported for Bulgaria.

**Service uptake** (Table 4)

The web-based usage of FRAX showed considerable heterogeneity in uptake between the countries. The average uptake for the EU27+2 was 1,555 sessions/million/year of the general population with an enormous range of 49 to 41,874 sessions/million. The usage for Bulgaria amounted to 49 sessions/million in 2019 (placing the country last amongst EU 27+2), with a 56 percent decrease since 2011.

Many studies have demonstrated that a significant proportion of men and women at high fracture risk do not receive therapy for osteoporosis (the treatment gap) [7]. In the EU27+2 the average treatment gap was 71% but ranged from 32 to 87%. For Bulgaria, the treatment gap amongst women amounted to 87% or 239,000 out of 273,000 characterised at risk. The Bulgarian treatment gap decreased by more than 5% compared to 2010, whilst the average treatment gap among EU27+2 increased from 55% in 2010 to 71% in 2019

**Table 4** Service uptake for osteoporosis in Bulgaria

**Table:** 

**Category**	**Measure**	**Estimate**	**Rank**
Service uptake	Number of FRAX sessions/million people/year	49	29
	Treatment gap for women eligible for treatment (%)	87	27
	Proportion surgically managed hip fractures	75–90%	

About 5% of people with a hip fracture die within 1 month of their fracture [8]. A determinant of peri-operative morbidity and mortality is the time a patient takes to get to surgery [9]. For Bulgaria, the average waiting time for hip fracture surgery after hospital admission was reported to be less than 24 h, implying a reduction in waiting time compared to 2010 (waiting time of 1–2 days). The proportion of surgically managed hip fractures was reported to be 75–90%.

**Scores and scorecard**

Scores were developed for Burden of disease and the healthcare provision (Policy framework, Service provision and Service uptake) in the EU27+2 countries. Bulgaria scores resulted in an 18th place regarding Burden of disease. The combined healthcare provision scorecard resulted in a 22nd place for Bulgaria. Thus, Bulgaria presents as one of the five low-burden low-provision countries among the EU27+2.

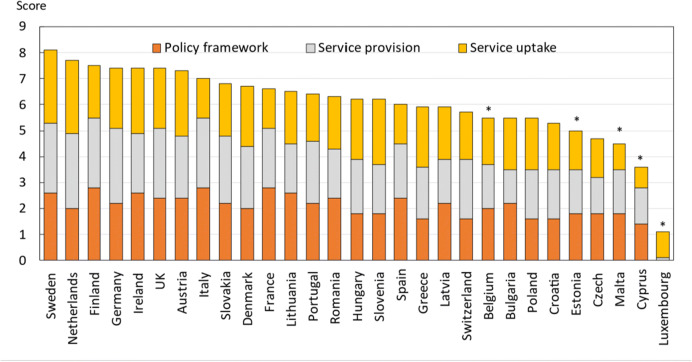



**Fig. 1** Scores by country for metrics related to policy framework, service provision and service uptake. The mean score for each of the 3 domains is given. An asterisk denotes that there was one or more missing metric which decreases the overall score

The first SCOPE was undertaken in 2010, almost 10 years previously. Fifteen of the 16 score card metrics on healthcare provision were used in the two surveys. Scores had improved or markedly improved in 15 countries, remained constant in 8 countries and worsened in 3 countries. For Bulgaria, the scores were somewhat improved.

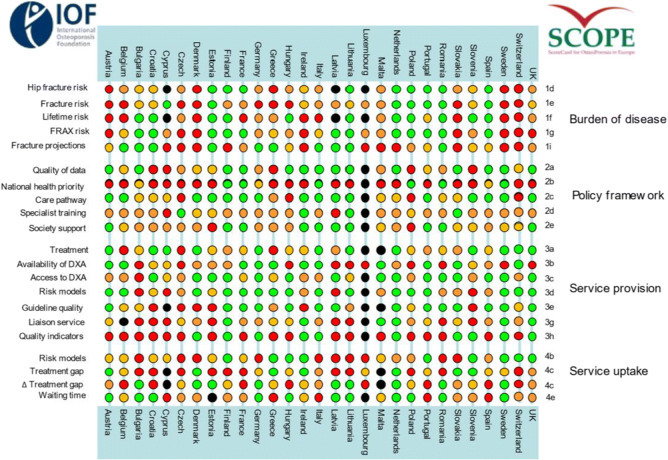



**Fig. 2** The scorecard for all the EU27+2 countries illustrating the scores across the four domains. The elements of each domain in each country were scored and coded using a traffic light system (red, orange, green). Black dots signify missing information

The second edition of the Scorecard for Osteoporosis in Europe (SCOPE 2021) allows health and policy professionals to assess key indicators on the healthcare provision for osteoporosis within countries and between countries within the EU 27+2. The scorecard is not intended as a prescriptive template. Thus, it does not set performance targets but may serve as a guide to the performance targets at which to aim in order to deliver the outcomes required.

**Acknowledgements**

SCOPE was supported by an unrestricted grant from Amgen to the International Osteoporosis Foundation (IOF). Amgen was neither involved in the design nor writing of the report. We are grateful to Anastasia Soulié Mlotek and Dominique Pierroz of the IOF for their help in the administration of SCOPE. The report has been reviewed by the members of the SCOPE Consultation Panel and the relevant IOF National societies, and we are grateful for their local insights on the management of osteoporosis in each country. The source document has been reviewed and endorsed by the Committee of Scientific Advisors of the IOF and benefitted from their feedback.

**References**

1. Kanis JA, Norton N, Harvey NC, Jacobson T, Johansson H, Lorentzon M, McCloskey EV, Willers C, Borgström F (2021) SCOPE 2021: a new scorecard for osteoporosis in Europe. Arch Osteoporos  16:82. 10.1007/s11657-020-00871-9

2. World Health Organisation (1994) Assessment of fracture risk and its application to screening for postmenopausal osteoporosis. Report of a WHO Study Group. World Health Organ Tech Rep Ser, 1994/01/01 edn, pp 1-129

3. Johnell O, Kanis JA, Oden A, Sernbo I, Redlund-Johnell I, Petterson C, De Laet C, Jonsson B (2004) Mortality after osteoporotic fractures. Osteoporos Int 15:38-42

4. Kirilova E, Johansson H, Kirilov N, Vladeva S, Petranova T, Kolarov Z, Liu E, Lorentzon M, Vandenput L, Harvey NC, McCloskey E, Kanis JA (2020) Epidemiology of hip fractures in Bulgaria: development of a country-specific FRAX model. Arch Osteoporos 27;15(1):28.

5. Hernlund E, Svedbom A, Ivergard M, Compston J, Cooper C, Stenmark J, McCloskey EV, Jonsson B, Kanis JA (2013) Osteoporosis in the European Union: medical management, epidemiology and economic burden. A report prepared in collaboration with the International Osteoporosis Foundation (IOF) and the European Federation of Pharmaceutical Industry Associations (EFPIA). Arch Osteoporos 8:136

6. Allen P, Pilar M, Walsh-Bailey C, Hooley C, Mazzucca S, Lewis CC, Mettert KD, Dorsey CN, Purtle J, Kepper MM, Baumann AA, Brownson RC (2020) Quantitative measures of health policy implementation determinants and outcomes: a systematic review. Implement Sci 15:47

7. Borgstrom F, Karlsson L, Ortsater G, Norton N, Halbout P, Cooper C, Lorentzon M, McCloskey EV, Harvey NC, Javaid MK, Kanis JA (2020) Fragility fractures in Europe: burden, management and opportunities. Arch Osteoporos 15:59

8. Kanis JA, Oden A, Johnell O, De Laet C, Jonsson B, Oglesby AK (2003) The components of excess mortality after hip fracture. Bone 32:468-473

9. National Clinical Guideline Centre (2011) The Management of Hip Fracture in Adults. In Centre NCG (ed) London

## **Epidemiology and economic burden of osteoporosis in Croatia**

S Grazio ∙ V Altabas ∙ Z Giljević∙ C Willers ∙ N Norton ∙ NC Harvey ∙ T Jacobson ∙ H Johansson ∙ M Lorentzon ∙ EV McCloskey ∙ F Borgström ∙ JA Kanis

**Introduction**

The scorecard summarises key indicators of the burden of osteoporosis and its management in the 27 member states of the European Union, as well as the UK and Switzerland (termed EU27+2) [1]. This country-specific report summarises the principal results for Croatia.

**Methods**

The information obtained covers four domains: burden of osteoporosis and fractures; policy framework; service provision; and service uptake. Data were collected from numerous sources including previous research and IOF reports, and available registers which were used for additional analysis of resource utilization, costing and HRQoL data. Furthermore, country-specific information on osteoporosis management was obtained from each IOF member state via a questionnaire.

**Burden of disease**

The direct cost of incident fractures in Croatia in 2019 was €71.3 million. Added to this was the ongoing cost in 2019 from fractures that occurred before 2019, which amounted to €58.6 million (long-term disability). The cost of pharmacological intervention (assessment and treatment) was €6.1 million. Thus, the total direct cost (excluding the value of QALYs lost) amounted to €136 million in 2019. Key metrics are presented in Table 1.

In 2019, the average direct cost of osteoporotic fractures in Croatia was €31.8 per individual. Data for 2010 were not available to assess the development, but the 2019 numbers put Croatia in 24^th^ place in terms of highest cost of osteoporotic fractures per capita in the EU27+2.

The cost of osteoporotic fractures in Croatia accounted for approximately 3.9% of healthcare spending (i.e. €136 million out of €3.3 billion in 2019), slightly higher than the EU27+2 average of 3.5% and placed Croatia in 10^th^ place in the rank order of the EU27+2 countries These numbers indicate a substantial impact of fragility fractures on the healthcare budget.

Using World Health Organization diagnostic criteria for osteoporosis based on the measurement of bone mineral density (BMD) [2], there were approximately 252,000 individuals with osteoporosis in Croatia in 2019, of whom approximately 80% were women. The prevalence of osteoporosis in the total Croatian population amounted to 5.5%, on par with the EU27+2 average (5.6%).

**Table 1** Key measures of burden of disease for Croatia

**Table:** 

**Category**	**Measure**	**Estimate**	**Rank**
Burden of disease	Direct cost of incident fracture (€m)	71.30	
	Long-term disability cost (€m)	58.55	
	Intervention cost (€m)	6.08	
	Total cost (€m)	135.93	
	QALYs lost (€m)	373	
	Cost per capita (€)	31.75	24
	Proportion of healthcare spending	3.9%	10
	Prevalence of osteoporosis	5.5%	13

There were estimated to be 35,000 new fragility fractures in Croatia in 2019, equivalent to 96 fractures/day (or 4 per hour). The remaining lifetime probability of hip fracture (%) at the ages of 50 years in men and women was 5.1% and 11.4%, respectively, placing Croatia in the middle tertile of risk for men and the lower tertile of risk for women.

Some osteoporotic fractures are associated with premature mortality [3]. In Croatia, the annual number of deaths associated with a fracture event was estimated to be 172 per 100,000 individuals of the population aged 50 years or more, compared to the EU27+2 average of 116/100,000. The number of fracture-related deaths is comparable to or exceeds that for some of the most common causes of death such as lung cancer, diabetes, chronic lower respiratory diseases.

The population of men and women age 50 years or more is projected to increase by 2.8% between 2019 and 2034, a significantly smaller increase than the EU27+2 average of 11.4%. The increases in men and women aged 75 years or more in Croatia are more marked and amount to 41.0% and 17.3%, respectively. The annual number of osteoporotic fractures in Croatia is expected to increase by 4,000 to 39,000 in 2034.

**Policy framework** (Table 2)

Documentation of the burden of disease is an essential prerequisite to determine the resources that should be allocated to the diagnosis and treatment of the disorder. High quality national data on hip fracture rates have been identified in 18 of 29 countries, of which Croatia is one. Data are collected on a national basis and include more than only hip fracture data.

Given that osteoporosis and fragility fractures are common and that effective treatments are widely available, the vast majority of patients with osteoporosis are preferably managed at the primary health care level by general practitioners (GPs), with specialist referral reserved for difficult complex cases. Primary care was the principal provider of the medical care for osteoporosis in 13 of the 28 countries where data were available. For Croatia, this was not the case, and the lead specialty for osteoporosis was reported to be endocrinology.

Osteoporosis and metabolic bone disease is not a recognised specialty in most countries including Croatia. Specialty care of osteoporosis in Croatia is managed via other specialties including endocrinology, rehabilitation medicine, orthopaedics and gynaecology. Osteoporosis is however recognized as a component of specialty training. Although it is possible that these specialties educate their trainees adequately, the wide variation may reflect inconsistencies in patient care, training of primary care physicians and a suboptimal voice to “defend” the interests of those who work within the field of osteoporosis.

**Table 2** Policy framework for osteoporosis in Croatia

**Table:** 

**Category**	**Measure**	**Estimate**
Policy framework	National fracture data availability	No
	OP recognized as a specialty	No
	OP primarily managed in primary care	No
	Other specialties involved	Endocrinology, Rehabilitation medicine, Orthopaedics, Gynaecology
	Advocacy areas covered by patient organisation	Policy, capacity, peer support

The role of national patient organisations is to improve the care of patients and increase awareness and prevention of osteoporosis and related fractures among the general public. Advocacy by patient organisations can fall into four categories: policy, capacity building and education, peer support, research and development. For Croatia, three of these four advocacy areas were covered by a patient organisation. All advocacy areas were covered for only 10 out of the 26 countries with at least one patient organisation.

**Service provision** (Table 3)

A wide variety of approved drug treatments is available for the management of osteoporosis [4]. Potential limitations of their use in member states relate to reimbursement policies which may impair the delivery of health care. Croatia is one of the 12 (out of 27) countries that offer full reimbursement.

The assessment of bone mineral density forms a key component for the general management of osteoporosis, being used for diagnosis, risk prediction, selection of patients for treatment and monitoring of patients on treatment. In Croatia, the number of DXA units expressed per million of the general population amounted to 10.8 which puts the country in the 19th place among the EU27+2.

The average waiting time for DXA ranged from 0 to 180 days across countries, and there was no clear relation between waiting times and the availability of DXA. In Croatia, the estimated average waiting time for DXA amounted to 21 days. 16 countries reported shorter average waiting times. Reimbursement for DXA scans varied between member states both in terms of the criteria required and level of reimbursement awarded.

**Table 3** Service provision for osteoporosis in Croatia

**Table:** 

**Category**	**Measure**	**Estimate**	**Rank**
Service provision	Reimbursement of OP medications	100%	
	DXA units/million inhabitants	10.8	19
	DXA cost (€)	25	22
	FRAX risk assessment model available	Yes	
	Fracture liaison service density	No FLS	

The effective targeting of treatment to those at highest risk of fracture requires an assessment of fracture risk. Risk assessment models for fractures, most usually based on FRAX, were available in 24 out of 29 countries, of which Croatia was one. For Croatia, guidance on the use of risk assessment within national guidelines was not available, as was the case in 14 of the EU27+2 countries.

Guidelines for the management of osteoporosis were available in Croatia (as in 27 out of 29 countries). The guidelines in Croatia included postmenopausal women specifically, as well as osteoporosis in men and secondary osteoporosis including glucocorticoid-induced osteoporosis.

Fracture liaison services (FLS), also known as osteoporosis coordinator programmes and care manager programmes, provide a system for the routine assessment and management of postmenopausal women and older men who have sustained a low trauma fracture. No fracture liaison services were reported from Croatia (together with 7 other countries).

The use of indicators to systematically measure the quality of care provided to people with osteoporosis or associated fractures has expanded as a discipline within the past decade [5]. No use of national quality indicators was reported for Croatia.

**Service uptake** (Table 4)

The web-based usage of FRAX showed considerable heterogeneity in uptake between the countries. The average uptake for the EU27+2 was 1,555 sessions/million/year of the general population with an enormous range of 49 to 41,874 sessions/million. The usage for Croatia amounted to 629 sessions/million.

Many studies have demonstrated that a significant proportion of men and women at high fracture risk do not receive therapy for osteoporosis (the treatment gap) [6]. In the EU27+2 the average treatment gap was 71% but ranged from 32 to 87%. For Croatia, the treatment gap amongst women amounted to 82% or 138,000 out of 169,000 characterised at risk. The Croatian treatment gap increased with approximately 15% compared to 2010. The average treatment gap among EU27+2 increased from 55% in 2010 to 71% in 2019.

**Table 4** Service uptake for osteoporosis in Croatia

**Table:** 

**Category**	**Measure**	**Estimate**	**Rank**
Service uptake	Number of FRAX sessions/million people/year	629	17
	Treatment gap for women eligible for treatment (%)	82	22
	Proportion surgically managed hip fractures	>90%	

About 5% of people with a hip fracture die within 1 month of their fracture [7]. A determinant of peri-operative morbidity and mortality is the time a patient takes to get to surgery [8]. For Croatia, the average waiting time for hip fracture surgery after hospital admission was reported to be less than 24 h. The proportion of surgically managed hip fractures was reported to be over 90%.

**Scores and scorecard**

Scores were developed for Burden of disease and the healthcare provision (Policy framework, Service provision and Service uptake) in the EU27+2 countries. Croatia scores resulted in a 19th place regarding Burden of disease. The combined healthcare provision scorecard resulted in a 24th place for Croatia. Thus, Croatia presents as one of the low-burden low-provision countries among the EU27+2.

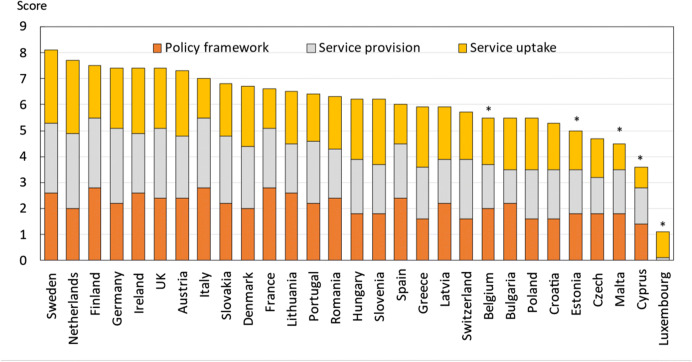



**Fig. 1** Scores by country for metrics related to policy framework, service provision and service uptake. The mean score for each of the 3 domains is given. An asterisk denotes that there was one or more missing metric which decreases the overall score

The first SCOPE was undertaken in 2010, almost 10 years previously. Fifteen of the 16 score card metrics on healthcare provision were used in the two surveys. Scores had improved or markedly improved in 15 countries, remained constant in 8 countries and worsened in 3 countries. For Croatia data for comparison to 2010 were not available.

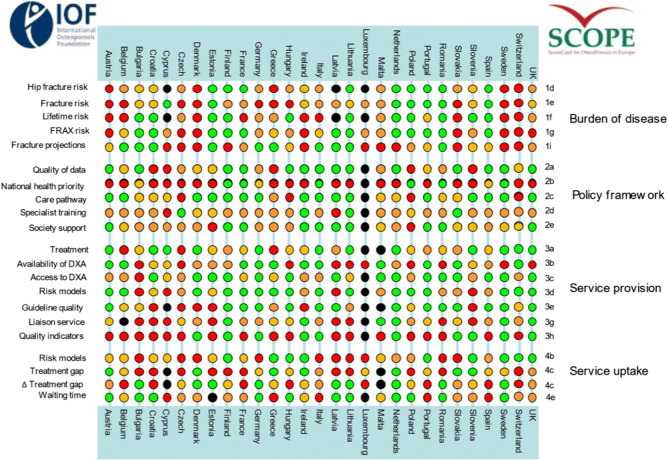



**Fig. 2** The scorecard for all the EU27+2 countries illustrating the scores across the four domains. The elements of each domain in each country were scored and coded using a traffic light system (red, orange, green). Black dots signify missing information

The second edition of the Scorecard for Osteoporosis in Europe (SCOPE 2021) allows health and policy professionals to assess key indicators on the healthcare provision for osteoporosis within countries and between countries within the EU 27+2. The scorecard is not intended as a prescriptive template. Thus, it does not set performance targets but may serve as a guide to the performance targets at which to aim in order to deliver the outcomes required.

**Acknowledgements**

SCOPE was supported by an unrestricted grant from Amgen to the International Osteoporosis Foundation (IOF). Amgen was neither involved in the design nor writing of the report. We are grateful to Anastasia Soulié Mlotek and Dominique Pierroz of the IOF for their help in the administration of SCOPE. We thank the Croatian League Against Rheumatism and the Croatian Society for Osteoporosis for their invaluable help. The report has been reviewed by the members of the SCOPE Consultation Panel and the relevant IOF National societies, and we are grateful for their local insights on the management of osteoporosis in each country. The source document has been reviewed and endorsed by the Committee of Scientific Advisors of the IOF and benefitted from their feedback.

**References**

1. Kanis JA, Norton N, Harvey NC, Jacobson T, Johansson H, Lorentzon M, McCloskey EV, Willers C, Borgström F (2021) SCOPE 2021: a new scorecard for osteoporosis in Europe. Arch Osteoporos 16:82. 10.1007/s11657-020-00871-9

2. World Health Organisation (1994) Assessment of fracture risk and its application to screening for postmenopausal osteoporosis. Report of a WHO Study Group. World Health Organ Tech Rep Ser, 1994/01/01 edn, pp 1-129

3. Johnell O, Kanis JA, Oden A, Sernbo I, Redlund-Johnell I, Petterson C, De Laet C, Jonsson B (2004) Mortality after osteoporotic fractures. Osteoporos Int 15:38-42

4. Hernlund E, Svedbom A, Ivergard M, Compston J, Cooper C, Stenmark J, McCloskey EV, Jonsson B, Kanis JA (2013) Osteoporosis in the European Union: medical management, epidemiology and economic burden. A report prepared in collaboration with the International Osteoporosis Foundation (IOF) and the European Federation of Pharmaceutical Industry Associations (EFPIA). Arch Osteoporos 8:136

5. Allen P, Pilar M, Walsh-Bailey C, Hooley C, Mazzucca S, Lewis CC, Mettert KD, Dorsey CN, Purtle J, Kepper MM, Baumann AA, Brownson RC (2020) Quantitative measures of health policy implementation determinants and outcomes: a systematic review. Implement Sci 15:47

6. Borgstrom F, Karlsson L, Ortsater G, Norton N, Halbout P, Cooper C, Lorentzon M, McCloskey EV, Harvey NC, Javaid MK, Kanis JA (2020) Fragility fractures in Europe: burden, management and opportunities. Arch Osteoporos 15:59

7. Kanis JA, Oden A, Johnell O, De Laet C, Jonsson B, Oglesby AK (2003) The components of excess mortality after hip fracture. Bone 32:468-473

8. National Clinical Guideline Centre (2011) The Management of Hip Fracture in Adults. In Centre NCG (ed)London

## Epidemiology and economic burden of osteoporosis in Cyprus

GL Georgiades ∙ C Willers ∙ N Norton ∙ NC Harvey ∙ T Jacobson ∙ H Johansson ∙ M Lorentzon ∙ EV McCloskey ∙ F Borgström ∙ JA Kanis

**Introduction**

The scorecard summarises key indicators of the burden of osteoporosis and its management in the 27 member states of the European Union, as well as the UK and Switzerland (termed EU27+2) [1]. This country-specific report summarises the principal results for Cyprus.

**Methods**

The information obtained covers four domains: burden of osteoporosis and fractures; policy framework; service provision; and service uptake. Data were collected from numerous sources including previous research and IOF reports, and available registers which were used for additional analysis of resource utilization, costing and HRQoL data. Furthermore, country-specific information on osteoporosis management was obtained from each IOF member state via a questionnaire.

**Burden of disease**

The direct cost of incident fractures in Cyprus in 2019 was €64.1 million. Added to this was the ongoing cost in 2019 from fractures that occurred before 2019, which amounted to €12.7 million (long-term disability). The cost of pharmacological intervention (assessment and treatment) was €8.9 million. Thus, the total direct cost (excluding the value of QALYs lost) amounted to €86 million in 2019. Key metrics are presented in Table 1.

In 2019, the average direct cost of osteoporotic fractures in Cyprus was €72.1 per individual in the population, while in 2010 the average was €51.9 (after adjusting for inflation), representing a relative increase of 39% (€72.1 versus €51.9). The 2019 numbers put Cyprus in 16^th^ place in terms of highest cost of osteoporotic fractures per capita in the EU27+2.

The cost of osteoporotic fractures in Cyprus accounted for approximately 5.8% of healthcare spending (i.e. 86 million out of €1.3 billion in 2019), significantly higher than the EU27+2 average of 3.5% and placed Cyprus 3^rd^ in the rank order of the EU27+2 countries. These numbers indicate a substantial impact of fragility fractures on the healthcare budget.

Using World Health Organization diagnostic criteria for osteoporosis based on the measurement of bone mineral density (BMD) [2], there were approximately 50,000 individuals with osteoporosis in Cyprus in 2019, of whom almost 80% were women. The prevalence of osteoporosis in the total population of Cyprus amounted to 3.7%, which is significantly lower than the EU27+2 average (5.6%).

**Table 1** Key measures of burden of disease for Cyprus

**Table:** 

**Category**	**Measure**	**Estimate**	**Rank**
Burden of disease	Direct cost of incident fracture (€m)	64.09	
	Long-term disability cost (€m)	12.71	
	Intervention cost (€m)	8.92	
	Total cost (€m)	85.73	
	QALYs lost (€m)	95	
	Cost per capita (€)	72.08	16
	Proportion of healthcare spending	5.8%	3
	Prevalence of osteoporosis	3.7%	28

There were estimated to be 6,600 new fragility fractures in Cyprus in 2019, equivalent to 18 fractures/day (or almost 1 per hour). This was a slight increase compared to 2010, equivalent to an increment of 0.6 fractures/1000 individuals, totalling 17.1 fractures/ 1000 individuals in 2019.

Some osteoporotic fractures are associated with premature mortality [3]. In Cyprus, the annual number of deaths associated with a fracture event was estimated to be 84 per 100,000 individuals of the population aged 50 years or more, compared to the EU27+2 average of 116/100,000. The number of fracture-related deaths is comparable to or exceeds that for some of the most common causes of death such as lung cancer, diabetes, chronic lower respiratory diseases.

The population in men and women age 50 years or more is projected to increase by 33.8% between 2019 and 2034, significantly above to the EU27+2 average of 11.4%. The increases in men and women aged 75 years or more are even more marked and amount to 71.0% and 59.4%, respectively. The annual number of osteoporotic fractures in Cyprus is expected to increase by 3,200 to almost 10,000 in 2034.

**Policy framework** (Table 2)

Documentation of the burden of disease is an essential prerequisite to determine the resources that should be allocated to the diagnosis and treatment of the disorder. High quality national data on hip fracture rates have been identified in 18 of 29 countries, Cyprus belonging to the remaining 11 countries.

Given that osteoporosis and fragility fractures are common and that effective treatments are widely available, the vast majority of patients with osteoporosis are preferably managed at the primary health care level by general practitioners (GPs), with specialist referral reserved for difficult complex cases. Primary care was the principal provider of the medical care for osteoporosis in 13 of the 28 countries where data were available. For Cyprus, endocrinology was reported to be the lead specialty for osteoporosis.

Osteoporosis and metabolic bone disease is not a recognised specialty in most countries including Cyprus. Furthermore, osteoporosis is not even recognized as a component of specialty training. This implies possible inconsistencies in patient care and a suboptimal voice to “defend” the interests of those who work within the field of osteoporosis.

**Table 2** Policy framework for osteoporosis in Cyprus

**Table:** 

**Category**	**Measure**	**Estimate**
Policy framework	National fracture data availability	No
	OP recognized as a specialty	No
	OP primarily managed in primary care	No
	Other specialties involved	Endocrinology
	Advocacy areas covered by patient organisation	Policy, capacity

The role of national patient organisations is to improve the care of patients and increase awareness and prevention of osteoporosis and related fractures among the general public. Advocacy by patient organisations can fall into four categories: policy, capacity building and education, peer support, research and development. For Cyprus, two of the four advocacy areas were covered by a patient organisation. All four advocacy areas were covered in only 10 out of the 26 countries with at least one patient organisation.

**Service provision** (Table 3)

A wide variety of approved drug treatments is available for the management of osteoporosis [4]. Potential limitations of their use in member states relate to reimbursement policies which may impair the delivery of health care. Cyprus is one of the 12 (out of 27) countries that offer full reimbursement.

The assessment of bone mineral density forms a key component for the general management of osteoporosis, being used for diagnosis, risk prediction, selection of patients for treatment and monitoring of patients on treatment. In Cyprus, the number of DXA units expressed per million of the general population amounted to 19.7 which puts the country in 12^th^ place among the EU27+2.

The average waiting time for DXA ranged from 0 to 180 days across countries, and there was no clear relation between waiting times and the availability of DXA. In Cyprus, the estimated average waiting time for DXA amounted to 120 days. Only one country (Spain) reported longer average waiting times.

**Table 3** Service provision for osteoporosis in Cyprus

**Table:** 

**Category**	**Measure**	**Estimate**	**Rank**
Service provision	Reimbursement of OP medications	100%	
	DXA units/million inhabitants	19.7	12
	DXA cost (€)	70	9
	FRAX risk assessment model available	No	
	Fracture liaison service density	No FLS	

Reimbursement for DXA scans varied between member states both in terms of the criteria required and level of reimbursement awarded. In Cyprus, the reimbursement was conditional and varied depending on patient income.

The effective targeting of treatment to those at highest risk of fracture requires an assessment of fracture risk. Risk assessment models for fractures, most usually based on FRAX, were available in 24 out of 29 countries, where Cyprus belonged to the remaining five countries. Guidelines for the management of osteoporosis were not available in Cyprus (as in only one other of the 29 countries).

Fracture liaison services (FLS), also known as osteoporosis coordinator programmes and care manager programmes, provide a system for the routine assessment and management of postmenopausal women and older men who have sustained a low trauma fracture. No fracture liaison services were reported from Cyprus (together with 7 other countries).

The use of indicators to systematically measure the quality of care provided to people with osteoporosis or associated fractures has expanded as a discipline within the past decade [5]. No use of national quality indicators was reported for Cyprus.

**Service uptake** (Table 4)

The web-based usage of FRAX showed considerable heterogeneity in uptake between the countries. The average uptake for the EU27+2 was 1,555 sessions/million/year of the general population with an enormous range of 49 to 41,874 sessions/million. The usage for Cyprus amounted to 1,058 sessions/million in 2019, an increase with almost 300% since 2011.

Many studies have demonstrated that a significant proportion of men and women at high fracture risk do not receive therapy for osteoporosis (the treatment gap) [6]. In the EU27+2 the average treatment gap was 71% but ranged from 32 to 87%. For Cyprus, there was no information available regarding the treatment gap. The average treatment gap among EU27+2 increased from 55% in 2010 to 71% in 2019.

**Table 4** Service uptake for osteoporosis in Cyprus

**Table:** 

**Category**	**Measure**	**Estimate**	**Rank**
Service uptake	Number of FRAX sessions/million people/year	1058	14
	Treatment gap for women eligible for treatment (%)	N/A	
	Proportion surgically managed hip fractures	75-90%	

About 5% of people with a hip fracture die within 1 month of their fracture [7]. A determinant of peri-operative morbidity and mortality is the time a patient takes to get to surgery [8]. For Cyprus, the average waiting time for hip fracture surgery after hospital admission was reported to be 2–3 days, implying similar levels as reported for 2010. The proportion of surgically managed hip fractures was reported to be 75–90%.

**Scores and scorecard**

Scores were developed for Burden of disease and the healthcare provision (Policy framework, Service provision and Service uptake) in the EU27+2 countries. Cyprus scores resulted in a 23rd place regarding Burden of disease. The combined healthcare provision scorecard resulted in a 28th place for Cyprus.

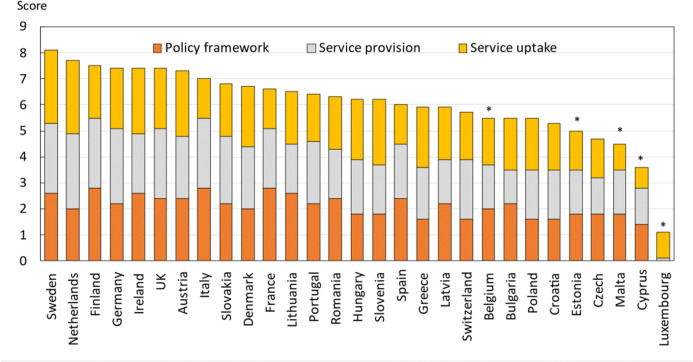



**Fig. 1** Scores by country for metrics related to policy framework, service provision and service uptake. The mean score for each of the 3 domains is given. An asterisk denotes that there was one or more missing metric which decreases the overall score

The first SCOPE was undertaken in 2010, almost 10 years previously. Fifteen of the 16 score card metrics on healthcare provision were used in the two surveys. Scores had improved or markedly improved in 15 countries, remained constant in 8 countries and worsened in 3 countries. For Cyprus, the scores were somewhat improved.

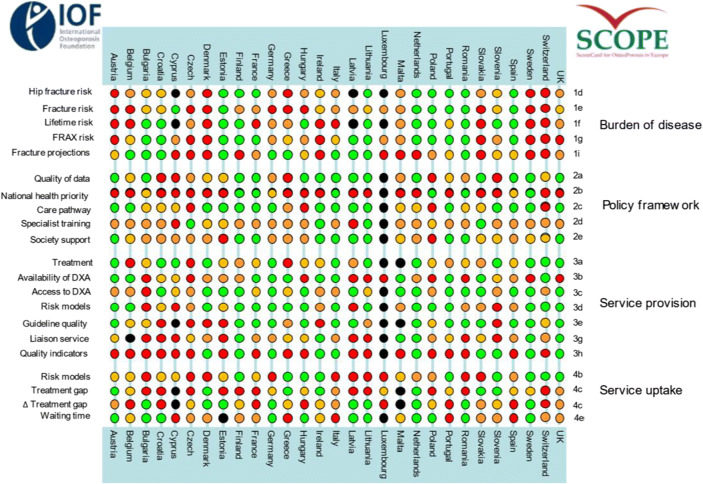



**Fig. 2** The scorecard for all the EU27+2 countries illustrating the scores across the four domains. The elements of each domain in each country were scored and coded using a traffic light system (red, orange, green). Black dots signify missing information

The second edition of the Scorecard for Osteoporosis in Europe (SCOPE 2021) allows health and policy professionals to assess key indicators on the healthcare provision for osteoporosis within countries and between countries within the EU 27+2. The scorecard is not intended as a prescriptive template. Thus, it does not set performance targets but may serve as a guide to the performance targets at which to aim in order to deliver the outcomes required.

**Acknowledgements**

SCOPE was supported by an unrestricted grant from Amgen to the International Osteoporosis Foundation (IOF). Amgen was neither involved in the design nor writing of the report. We are grateful to Anastasia Soulié Mlotek and Dominique Pierroz of the IOF for their help in the administration of SCOPE. We thank Dr Myrto Azina MD, Medical Officer at the Ministry of Health of the Republic of Cyprus, for her valuable contribution and information that she provided. The report has been reviewed by the members of the SCOPE Consultation Panel and the relevant IOF National societies, and we are grateful for their local insights on the management of osteoporosis in each country. The source document has been reviewed and endorsed by the Committee of Scientific Advisors of the IOF and benefitted from their feedback.

**References**

1. Kanis JA, Norton N, Harvey NC, Jacobson T, Johansson H, Lorentzon M, McCloskey EV, Willers C, Borgström F (2021) SCOPE 2021: a new scorecard for osteoporosis in Europe. Arch Osteoporos 16:82. 10.1007/s11657-020-00871-9

2. World Health Organisation (1994) Assessment of fracture risk and its application to screening for postmenopausal osteoporosis. Report of a WHO Study Group. World Health Organ Tech Rep Ser, 1994/01/01 edn, pp 1-129

3. Johnell O, Kanis JA, Oden A, Sernbo I, Redlund-Johnell I, Petterson C, De Laet C, Jonsson B (2004) Mortality after osteoporotic fractures. Osteoporos Int 15:38-42

4. Hernlund E, Svedbom A, Ivergard M, Compston J, Cooper C, Stenmark J, McCloskey EV, Jonsson B, Kanis JA (2013) Osteoporosis in the European Union: medical management, epidemiology and economic burden. A report prepared in collaboration with the International Osteoporosis Foundation (IOF) and the European Federation of Pharmaceutical Industry Associations (EFPIA). Arch Osteoporos 8:136

5. Allen P, Pilar M, Walsh-Bailey C, Hooley C, Mazzucca S, Lewis CC, Mettert KD, Dorsey CN, Purtle J, Kepper MM, Baumann AA, Brownson RC (2020) Quantitative measures of health policy implementation determinants and outcomes: a systematic review. Implement Sci 15:47

6. Borgstrom F, Karlsson L, Ortsater G, Norton N, Halbout P, Cooper C, Lorentzon M, McCloskey EV, Harvey NC, Javaid MK, Kanis JA (2020) Fragility fractures in Europe: burden, management and opportunities. Arch Osteoporos 15:59

7. Kanis JA, Oden A, Johnell O, De Laet C, Jonsson B, Oglesby AK (2003) The components of excess mortality after hip fracture. Bone 32:468-473

8. National Clinical Guideline Centre (2011) The Management of Hip Fracture in Adults. In Centre NCG (ed)London

## Epidemiology and economic burden of osteoporosis in the Czech Republic

R Pikner ∙ J Rosa ∙ P Kasalicky ∙ V Palicka ∙ C Willers ∙ N Norton ∙ NC Harvey ∙ T Jacobson ∙ H Johansson ∙ M Lorentzon ∙ EV McCloskey ∙ F Borgström ∙ JA Kanis

**Introduction**

The scorecard summarises key indicators of the burden of osteoporosis and its management in the 27 member states of the European Union, as well as the UK and Switzerland (termed EU27+2) [1]. This country-specific report summarises the principal results for Czech Republic.

**Methods**

The information obtained covers four domains: burden of osteoporosis and fractures; policy framework; service provision; and service uptake. Data were collected from numerous sources including previous research and IOF reports, and available registers which were used for additional analysis of resource utilization, costing and HRQoL data. Furthermore, country-specific information on osteoporosis management was obtained from each IOF member state via a questionnaire.

**Burden of disease**

The direct cost of incident fractures in Czech Republic in 2019 was €260.1 million. Added to this was the ongoing cost in 2019 from fractures that occurred before 2019, which amounted to €121.3 million (long-term disability). The cost of pharmacological intervention (assessment and treatment) was €14.1 million. Thus, the total direct cost (excluding the value of QALYs lost) amounted to €396 million in 2019. Key metrics are presented in Table 1.

In 2019, the average direct cost of osteoporotic fractures in Czech Republic was €37.3 per individual in the population, while in 2010 the average was €28.7 (after adjusting for inflation), representing an increase of 30% (€37.3 versus €28.7). The 2019 data put the Czech Republic in 21st place in terms of highest cost of osteoporotic fractures per capita in the EU27+2.

The cost of osteoporotic fractures in Czech Republic accounted for approximately 2.7% of healthcare spending (i.e. €396 million out of €14.0 billion in 2019), somewhat lower than the EU27+2 average of 3.5%. Nonetheless, these numbers indicate a substantial impact of fragility fractures on the healthcare budget.

Using World Health Organization diagnostic criteria for osteoporosis based on the measurement of bone mineral density (BMD) [2], there were approximately 572,000 individuals with osteoporosis in Czech Republic in 2019, of whom approximately 80% were women. The prevalence of osteoporosis in the total population of Czech Republic amounted to 5.0%, on par with the EU27+2 average (5.6%).

**Table 1** Key measures of burden of disease for Czech Republic

**Table:** 

**Category**	**Measure**	**Estimate**	**Rank**
Burden of disease	Direct cost of incident fracture (€m)	260.88	
	Long-term disability cost (€m)	121.34	
	Intervention cost (€m)	14.05	
	Total cost (€m)	396.27	
	QALYs lost (€m)	1350	
	Cost per capita (€)	37.29	21
	Proportion of healthcare spending	2.7%	18
	Prevalence of osteoporosis	5.0%	21

There were estimated to be 91,000 new fragility fractures in Czech Republic in 2019, equivalent to 250 fractures/day (or more than 10 per hour). This was an increase compared to 2010, equivalent to 3.0 fractures/1000 individuals, totalling 22.0 fractures/ 1000 individuals in 2019.

Some osteoporotic fractures are associated with premature mortality [3]. In Czech Republic, the annual number of deaths associated with a fracture event was estimated to be 159 per 100,000 individuals of the population aged 50 years or more, compared to the EU27+2 average of 116/100,000. The number of fracture-related deaths is comparable to or exceeds that for some of the most common causes of death such as lung cancer, diabetes, chronic lower respiratory diseases.

The remaining lifetime probability of hip fracture (%) at the ages of 50 years in men and women was 6.9% and 14.8%, respectively, placing Czech Republic in the mid tertile of risk for both men and women.

The population in men and women age 50 years or more is projected to increase by 18.5% between 2019 and 2034, significantly above the EU27+2 average of 11.4%. The increases in men and women aged 75 years or more are even more marked and amount to 60.9% and 41.1%, respectively. The annual number of osteoporotic fractures in Czech Republic is expected to increase by 32,000 to 123,000 in 2034.

**Policy framework** (Table 2)

Documentation of the burden of disease is an essential prerequisite to determine the resources that should be allocated to the diagnosis and treatment of the disorder. High quality national data on hip fracture rates have been identified in 18 of 29 countries, of which Czech Republic is one. Data are collected on a national basis and include more than only hip fracture data.

Given that osteoporosis and fragility fractures are common and that effective treatments are widely available, the vast majority of patients with osteoporosis are preferably managed at the primary health care level by general practitioners (GPs), with specialist referral reserved for difficult complex cases in most countries. Primary care was the principal provider of the medical care for osteoporosis in 13 of the 28 countries where data were available.

Osteoporosis and metabolic bone disease is a recognised specialty in Czech Republic whilst it is not in most other countries. Other specialties involved in osteoporosis management were reported to be osteology, internal medicine, rheumatology, endocrinology, orthopaedics and gynaecology.

**Table 2** Policy framework for osteoporosis in the Czech Republic

**Table:** 

**Category**	**Measure**	**Estimate**
Policy framework	National fracture data availability	No
	OP recognized as a specialty	Yes
	OP primarily managed in primary care	No
	Other specialties involved	Osteology, Rheumatology, Internal medicine, Orthopaedics, Gynaecology, Endocrinology
	Advocacy areas covered by patient organisation	Peer support

The role of national patient organisations is to improve the care of patients and increase awareness and prevention of osteoporosis and related fractures among the general public. Advocacy by patient organisations can fall into four categories: policy, capacity building and education, peer support, research and development. For Czech Republic, one of the four of the advocacy areas, peer support, were covered by a patient organisation. All four advocacy areas were covered in only 10 out of the 26 countries with at least one patient organisation.

**Service provision** (Table 3)

A wide variety of approved drug treatments is available for the management of osteoporosis [4]. Potential limitations of their use in member states relate to reimbursement policies which may impair the delivery of health care. Czech Republic offered 90–100% reimbursement, i.e. close to full. 12 out of 27 countries offered full reimbursement.

The assessment of bone mineral density forms a key component for the general management of osteoporosis, being used for diagnosis, risk prediction, selection of patients for treatment and monitoring of patients on treatment. In Czech Republic, the number of DXA units expressed per million of the general population amounted to 8.1 which puts the country in 21st place among the EU27+2. The proportion of DXA units providing TBS was relatively high in Czech Republic compared to other countries.

The average waiting time for DXA ranged from 0 to 180 days across countries, and there was no clear relation between waiting times and the availability of DXA. In Czech Republic, the estimated average waiting time for DXA amounted to 30 days. 18 countries reported shorter average waiting times.

**Table 3** Service provision for osteoporosis in the Czech Republic

**Table:** 

**Category**	**Measure**	**Estimate**	**Rank**
Service provision	Reimbursement of OP medications	90-100%	
	DXA units/million inhabitants	8.1	21
	DXA cost (€)	30	20
	FRAX risk assessment model available	Yes	
	Fracture liaison service density	1-10%	

Reimbursement for DXA scans varied between member states both in terms of the criteria required and level of reimbursement awarded. In Czech Republic, the reimbursement was conditional and varied depending on the patient’s condition.

The effective targeting of treatment to those at highest risk of fracture requires an assessment of fracture risk. Risk assessment models for fractures, most usually based on FRAX, were available in 24 out of 29 countries, of which Czech Republic was one. For Czech Republic, guidance on the use of risk assessment within national guidelines was not available, as there was in 14 of the other countries.

Guidelines for the management of osteoporosis were available in Czech Republic (as in 27 out of 29 countries). The guidelines in Czech Republic included postmenopausal women specifically, as well as osteoporosis in men and secondary osteoporosis including glucocorticoid-induced osteoporosis.

Fracture liaison services (FLS), also known as osteoporosis coordinator programmes and care manager programmes, provide a system for the routine assessment and management of postmenopausal women and older men who have sustained a low trauma fracture. Fracture liaison services were reported for 1-10% of the hospitals in Czech Republic.

The use of indicators to systematically measure the quality of care provided to people with osteoporosis or associated fractures has expanded as a discipline within the past decade [5]. No use of national quality indicators was reported for Czech Republic.

**Service uptake** (Table 4)

The web-based usage of FRAX showed considerable heterogeneity in uptake between the countries. The average uptake for the EU27+2 was 1,555 sessions/million/year of the general population with an enormous range of 49 to 41,874 sessions/million. The usage for Czech Republic amounted to 344 sessions/million in 2019, with an almost 100 percent increase since 2011.

Many studies have demonstrated that a significant proportion of men and women at high fracture risk do not receive therapy for osteoporosis (the treatment gap) [6]. In the EU27+2 the average treatment gap was 71% but ranged from 32 to 87%. For Czech Republic, the treatment gap amongst women amounted to 79% or 285,000 out of 360,000 characterised at risk. The treatment gap did not change significantly compared to 2010, whilst the average treatment gap among EU27+2 increased from 55% in 2010 to 71% in 2019.

**Table 4** Service uptake for osteoporosis in the Czech Republic

**Table:** 

**Category**	**Measure**	**Estimate**	**Rank**
Service uptake	Number of FRAX sessions/million people/year	344	24
	Treatment gap for women eligible for treatment (%)	79	19
	Proportion surgically managed hip fractures	67%	

About 5% of people with a hip fracture die within 1 month of their fracture [7]. A determinant of peri-operative morbidity and mortality is the time a patient takes to get to surgery [8]. For Czech Republic, the average waiting time for hip fracture surgery after hospital admission was reported to be 1–2 days. The proportion of surgically managed hip fractures was reported to be 67%.

**Scores and scorecard**

Scores were developed for Burden of disease and the healthcare provision (Policy framework, Service provision and Service uptake) in the EU27+2 countries. Czech Republic scores resulted in a 6th place regarding Burden of disease. The combined healthcare provision scorecard resulted in a 26th place for Czech Republic. Thus, Czech Republic presents as one of the eight high-burden low-provision countries among the EU27+2.

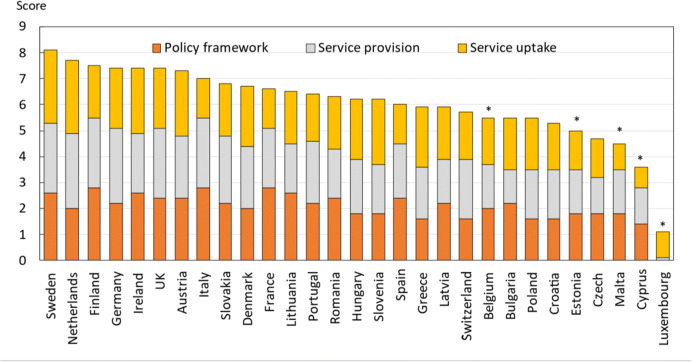



**Fig. 1** Scores by country for metrics related to policy framework, service provision and service uptake. The mean score for each of the 3 domains is given. An asterisk denotes that there was one or more missing metric which decreases the overall score

The first SCOPE was undertaken in 2010, almost 10 years previously. Fifteen of the 16 score card metrics on healthcare provision were used in the two surveys. Scores had improved or markedly improved in 15 countries, remained constant in 8 countries and worsened in 3 countries. For Czech Republic, the scores were somewhat worsened. 
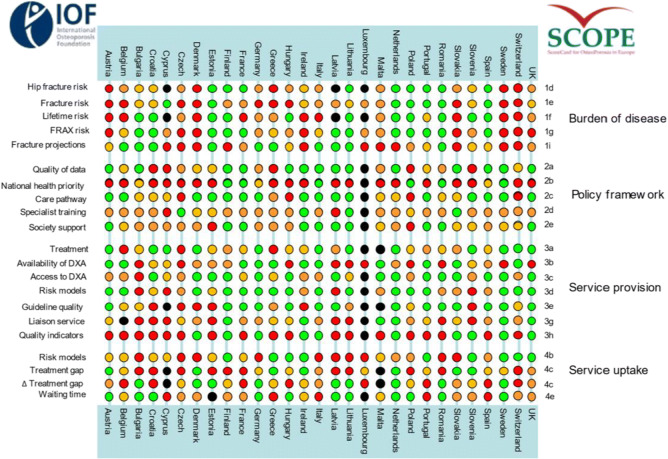


**Fig. 2** The scorecard for all the EU27+2 countries illustrating the scores across the four domains. The elements of each domain in each country were scored and coded using a traffic light system (red, orange, green). Black dots signify missing information

The second edition of the Scorecard for Osteoporosis in Europe (SCOPE 2021) allows health and policy professionals to assess key indicators on the healthcare provision for osteoporosis within countries and between countries within the EU 27+2. The scorecard is not intended as a prescriptive template. Thus, it does not set performance targets but may serve as a guide to the performance targets at which to aim in order to deliver the outcomes required.

**Acknowledgements**

SCOPE was supported by an unrestricted grant from Amgen to the International Osteoporosis Foundation (IOF). Amgen was neither involved in the design nor writing of the report. We are grateful to Anastasia Soulié Mlotek and Dominique Pierroz of the IOF for their help in the administration of SCOPE. The report has been reviewed by the members of the SCOPE Consultation Panel and the relevant IOF National societies, and we are grateful for their local insights on the management of osteoporosis in each country. The source document has been reviewed and endorsed by the Committee of Scientific Advisors of the IOF and benefitted from their feedback. The country report was supported by the Czech Society for Metabolic Bone Diseases, Czech Medical Association of JE Purkyne.

**References**

1. Kanis JA, Norton N, Harvey NC, Jacobson T, Johansson H, Lorentzon M, McCloskey EV, Willers C, Borgström F (2021) SCOPE 2021: a new scorecard for osteoporosis in Europe. Arch Osteoporos 16:82. 10.1007/s11657-020-00871-9

2. World Health Organisation (1994) Assessment of fracture risk and its application to screening for postmenopausal osteoporosis. Report of a WHO Study Group. World Health Organ Tech Rep Ser, 1994/01/01 edn, pp 1-129

3. Johnell O, Kanis JA, Oden A, Sernbo I, Redlund-Johnell I, Petterson C, De Laet C, Jonsson B (2004) Mortality after osteoporotic fractures. Osteoporos Int 15:38-42

4. Hernlund E, Svedbom A, Ivergard M, Compston J, Cooper C, Stenmark J, McCloskey EV, Jonsson B, Kanis JA (2013) Osteoporosis in the European Union: medical management, epidemiology and economic burden. A report prepared in collaboration with the International Osteoporosis Foundation (IOF) and the European Federation of Pharmaceutical Industry Associations (EFPIA). Arch Osteoporos 8:136

5. Allen P, Pilar M, Walsh-Bailey C, Hooley C, Mazzucca S, Lewis CC, Mettert KD, Dorsey CN, Purtle J, Kepper MM, Baumann AA, Brownson RC (2020) Quantitative measures of health policy implementation determinants and outcomes: a systematic review. Implement Sci 15:47

6. Borgstrom F, Karlsson L, Ortsater G, Norton N, Halbout P, Cooper C, Lorentzon M, McCloskey EV, Harvey NC, Javaid MK, Kanis JA (2020) Fragility fractures in Europe: burden, management and opportunities. Arch Osteoporos 15:59

7. Kanis JA, Oden A, Johnell O, De Laet C, Jonsson B, Oglesby AK (2003) The components of excess mortality after hip fracture. Bone 32:468-473

8. National Clinical Guideline Centre (2011) The Management of Hip Fracture in Adults. In Centre NCG (ed)London

## Epidemiology and economic burden of osteoporosis in Denmark

B Abrahamsen ∙ P Hermann ∙ C Willers ∙ N Norton ∙ NC Harvey ∙ T Jacobson ∙ H Johansson ∙ M Lorentzon ∙ EV McCloskey ∙ F Borgström ∙ JA Kanis

**Introduction**

The scorecard summarises key indicators of the burden of osteoporosis and its management in the 27 member states of the European Union, as well as the UK and Switzerland (termed EU27+2) [1]. This country-specific report summarises the principal results for Denmark.

**Methods**

The information obtained covers four domains: burden of osteoporosis and fractures; policy framework; service provision; and service uptake. Data were collected from numerous sources including previous research and IOF reports, and available registers which were used for additional analysis of resource utilization, costing and HRQoL data. Furthermore, country-specific information on osteoporosis management was obtained from each IOF member state via a questionnaire.

**Burden of disease**

The direct cost of incident fractures in Denmark in 2019 was €852.8 million. Added to this was the ongoing cost in 2019 from fractures that occurred before 2019, which amounted to €548.4 million (long-term disability). The cost of pharmacological intervention (assessment and treatment) was €51.2 million. Thus, the total direct cost (excluding the value of QALYs lost) amounted to €1.45 billion in 2019. Key metrics are presented in Table 1.

In 2019, the average direct cost of osteoporotic fractures in Denmark was €250.5 per individual in the population, while in 2010 the average was €209.7 (after adjusting for inflation), representing an increase of 19% (€250.5 versus €209.7. The 2019 data rank Denmark in the 2nd place in terms of highest cost of osteoporotic fractures per capita in the EU27+2.

The cost of osteoporotic fractures in Denmark accounted for approximately 4.7% of healthcare spending (i.e. €1.45 billion out of €29.8 billion in 2019), significantly higher than the EU27+2 average of 3.5%. These numbers indicate a substantial impact of fragility fractures on the healthcare budget.

Using World Health Organization diagnostic criteria for osteoporosis based on the measurement of bone mineral density (BMD) [2], there were approximately 328,000 individuals with osteoporosis in Denmark in 2019, of whom almost 80% were women. The prevalence of osteoporosis in the total Danish population amounted to 5.1%, on par with the EU27+2 average (5.6%).

**Table 1** Key measures of burden of disease for Denmark

**Table:** 

**Category**	**Measure**	**Estimate**	**Rank**
Burden of disease	Direct cost of incident fracture (€m)	852.75	
	Long-term disability cost (€m)	548.37	
	Intervention cost (€m)	51.15	
	Total cost (€m)	1452.27	
	QALYs lost (€m)	3096	
	Cost per capita (€)	250.5	2
	Proportion of healthcare spending	4.7%	6
	Prevalence of osteoporosis	5.1%	20

There were estimated to be 86,000 new fragility fractures in Denmark in 2019, equivalent to 236 fractures/day (or 10 per hour). This was a slight increase compared to 2010, equivalent to an increment of 3.9 fractures/1000 individuals, totalling 37.0 fractures/ 1000 individuals in 2019.

Some osteoporotic fractures are associated with premature mortality [3]. In Denmark, the annual number of deaths associated with a fracture event was estimated to be 211 per 100,000 individuals of the population aged 50 years or more, compared to the EU27+2 average of 116/100,000. The number of fracture-related deaths is comparable to or exceeds that for some of the most common causes of death such as lung cancer, diabetes, chronic lower respiratory diseases.

The remaining lifetime probability of hip fracture (%) at the ages of 50 years in men and women was 10.6% and 22.1%, respectively, placing Denmark in the upper tertile of risk for both men and women.

The Danish population of men and women age 50 years or more is projected to increase by 7.0% between 2019 and 2034, somewhat less than the EU27+2 average of 11.4%. The increases in men and women aged 75 years or more are more marked and amount to 48.2% and 38.8%, respectively. The annual number of osteoporotic fractures in Denmark is expected to increase by 28,000 to 114,000 in 2034.

**Policy framework** (Table 2)

Documentation of the burden of disease is an essential prerequisite to determine the resources that should be allocated to the diagnosis and treatment of the disorder. High quality national data on hip fracture rates have been identified in 18 of 29 countries, of which Denmark is one. Data are collected on a national basis and include more than only hip fracture data.

Given that osteoporosis and fragility fractures are common and that effective treatments are widely available, the vast majority of patients with osteoporosis are preferably managed at the primary health care level by general practitioners (GPs), with specialist referral reserved for difficult complex cases. Primary care was the principal provider of the medical care for osteoporosis in Denmark, as for 13 of the 28 countries where data were available.

Osteoporosis and metabolic bone disease is not a recognised specialty in most countries including Denmark. Specialty care of osteoporosis in Denmark is managed via other specialties including endocrinology and rheumatology. Osteoporosis is however recognized as a component of specialty training. Although it is possible that these specialties educate their trainees adequately, the wide variation may reflect inconsistencies in patient care, training of primary care physicians and a suboptimal voice to “defend” the interests of those who work within the field of osteoporosis.

**Table 2** Policy framework for osteoporosis in Denmark

**Table:** 

**Category**	**Measure**	**Estimate**
Policy framework	National fracture data availability	No
	OP recognized as a specialty	No
	OP primarily managed in primary care	Yes
	Other specialties involved	Endocrinology, Rheumatology
	Advocacy areas covered by patient organisation	Policy, capacity, research and development

The role of national patient organisations is to improve the care of patients and increase awareness and prevention of osteoporosis and related fractures among the general public. Advocacy by patient organisations can fall into four categories: policy, capacity building and education, peer support, research and development. For Denmark, three of the four advocacy areas were covered by a patient organisation. All four advocacy areas were covered in only 10 out of the 26 countries with at least one patient organisation.

**Service provision** (Table 3)

A wide variety of approved drug treatments is available for the management of osteoporosis [4]. Potential limitations of their use in member states relate to reimbursement policies which may impair the delivery of health care. Denmark is one of the 12 (out of 27) countries that offer full reimbursement.

The assessment of bone mineral density forms a key component for the general management of osteoporosis, being used for diagnosis, risk prediction, selection of patients for treatment and monitoring of patients on treatment. In Denmark, the number of DXA units expressed per million of the general population amounted to 17.4 which puts the country in 14th place among the EU27+2. Furthermore, the availability of TBS was high, putting Denmark in third place.

The average waiting time for DXA ranged from 0 to 180 days across countries, and there was no clear relation between waiting times and the availability of DXA. In Denmark, the estimated average waiting time for DXA amounted to 90 days. 23 countries reported shorter average waiting times.

**Table 3** Service provision for osteoporosis in Denmark

**Table:** 

**Category**	**Measure**	**Estimate**	**Rank**
Service provision	Reimbursement of OP medications	100%	
	DXA units/million inhabitants	17.4	14
	DXA cost (€)	100	3
	FRAX risk assessment model available	Yes	
	Fracture liaison service density	10-25%	

Reimbursement for DXA scans varied between member states both in terms of the criteria required and level of reimbursement awarded. In Denmark, the reimbursement was unconditional for those patients that fulfil criteria (based on BMD and risk factors).

The effective targeting of treatment to those at highest risk of fracture requires an assessment of fracture risk. Risk assessment models for fractures, most usually based on FRAX, were available in 24 out of 29 countries, of which Denmark was one. For Denmark, guidance on the use of risk assessment within national guidelines was available, as in only 14 of the other countries.

Guidelines for the management of osteoporosis were available in Denmark (as in 27 out of 29 countries). The guidelines in Denmark included postmenopausal women specifically, as well as for osteoporosis in men and for secondary osteoporosis including glucocorticoid-induced osteoporosis.

Fracture liaison services (FLS), also known as osteoporosis coordinator programmes and care manager programmes, provide a system for the routine assessment and management of postmenopausal women and older men who have sustained a low trauma fracture. Fracture liaison services were reported for 10–25% of the hospitals in Denmark.

The use of indicators to systematically measure the quality of care provided to people with osteoporosis or associated fractures has expanded as a discipline within the past decade [5]. Systematic use of national quality indicators was reported for Denmark regarding hip fractures.

**Service uptake** (Table 4)

The web-based usage of FRAX showed considerable heterogeneity in uptake between the countries. The average uptake for the EU27+2 was 1,555 sessions/million/year of the general population with an enormous range of 49 to 41,874 sessions/million. The use of FRAX in Denmark amounted to 319 sessions/million in 2019, with a 66 percent decrease since 2011.

Many studies have demonstrated that a significant proportion of men and women at high fracture risk do not receive therapy for osteoporosis (the treatment gap) [6]. In the EU27+2 the average treatment gap was 71% but ranged from 32 to 87%. For Denmark, the treatment gap amongst women amounted to 43% or 93,000 out of 218,000 characterised at risk. The Danish treatment gap decreased somewhat compared to 2010, whilst the average treatment gap among EU27+2 increased from 55% in 2010 to 71% in 2019.

**Table 4** Service uptake for osteoporosis in Denmark

**Table:** 

**Category**	**Measure**	**Estimate**	**Rank**
Service uptake	Number of FRAX sessions/million people/year	319	25
	Treatment gap for women eligible for treatment (%)	43	2
	Proportion surgically managed hip fractures	>90%	

About 5% of people with a hip fracture die within 1 month of their fracture [7]. A determinant of peri-operative morbidity and mortality is the time a patient takes to get to surgery [8]. For Denmark, the average waiting time for hip fracture surgery after hospital admission was reported to be 1–2 days. The proportion of surgically managed hip fractures was reported to be over 90%.

**Scores and scorecard**

Scores were developed for Burden of disease and the healthcare provision (Policy framework, Service provision and Service uptake) in the EU27+2 countries. Denmark scores resulted in a 1^st^ place regarding Burden of disease. The combined healthcare provision scorecard resulted in a 10th place for Denmark. Thus, Denmark presents as one of the high-burden high-provision countries among the EU27+2.

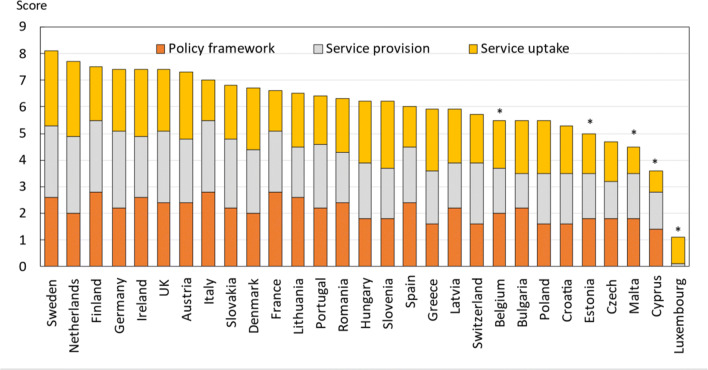



**Fig. 1** Scores by country for metrics related to policy framework, service provision and service uptake. The mean score for each of the 3 domains is given. An asterisk denotes that there was one or more missing metric which decreases the overall score

The first SCOPE was undertaken in 2010, almost 10 years previously. Fifteen of the 16 score card metrics on healthcare provision were used in the two surveys. Scores had improved or markedly improved in 15 countries, remained constant in 8 countries and worsened in 3 countries. For Denmark, the scores were somewhat improved.

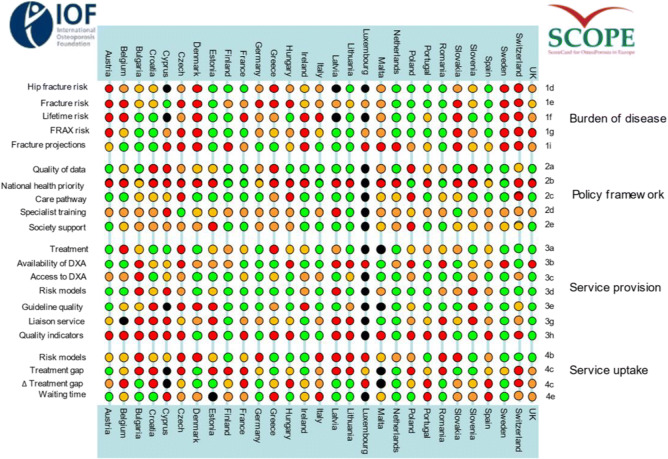



**Fig. 2** The scorecard for all the EU27+2 countries illustrating the scores across the four domains. The elements of each domain in each country were scored and coded using a traffic light system (red, orange, green). Black dots signify missing information

The second edition of the Scorecard for Osteoporosis in Europe (SCOPE 2021) allows health and policy professionals to assess key indicators on the healthcare provision for osteoporosis within countries and between countries within the EU 27+2. The scorecard is not intended as a prescriptive template. Thus, it does not set performance targets but may serve as a guide to the performance targets at which to aim in order to deliver the outcomes required.

**Acknowledgements**

SCOPE was supported by an unrestricted grant from Amgen to the International Osteoporosis Foundation (IOF). Amgen was neither involved in the design nor writing of the report. We are grateful to Anastasia Soulié Mlotek and Dominique Pierroz of the IOF for their help in the administration of SCOPE. We acknowledge the assistance of the Danish Bone Society. The report has been reviewed by the members of the SCOPE Consultation Panel and the relevant IOF National societies, and we are grateful for their local insights on the management of osteoporosis in each country. The source document has been reviewed and endorsed by the Committee of Scientific Advisors of the IOF and benefitted from their feedback.

**References**

1. Kanis JA, Norton N, Harvey NC, Jacobson T, Johansson H, Lorentzon M, McCloskey EV, Willers C, Borgström F (2021) SCOPE 2021: a new scorecard for osteoporosis in Europe. Arch Osteoporos 16:82. 10.1007/s11657-020-00871-9

2. World Health Organisation (1994) Assessment of fracture risk and its application to screening for postmenopausal osteoporosis. Report of a WHO Study Group. World Health Organ Tech Rep Ser, 1994/01/01 edn, pp 1-129

3. Johnell O, Kanis JA, Oden A, Sernbo I, Redlund-Johnell I, Petterson C, De Laet C, Jonsson B (2004) Mortality after osteoporotic fractures. Osteoporos Int 15:38-42

4. Hernlund E, Svedbom A, Ivergard M, Compston J, Cooper C, Stenmark J, McCloskey EV, Jonsson B, Kanis JA (2013) Osteoporosis in the European Union: medical management, epidemiology and economic burden. A report prepared in collaboration with the International Osteoporosis Foundation (IOF) and the European Federation of Pharmaceutical Industry Associations (EFPIA). Arch Osteoporos 8:136

5. Allen P, Pilar M, Walsh-Bailey C, Hooley C, Mazzucca S, Lewis CC, Mettert KD, Dorsey CN, Purtle J, Kepper MM, Baumann AA, Brownson RC (2020) Quantitative measures of health policy implementation determinants and outcomes: a systematic review. Implement Sci 15:47

6. Borgstrom F, Karlsson L, Ortsater G, Norton N, Halbout P, Cooper C, Lorentzon M, McCloskey EV, Harvey NC, Javaid MK, Kanis JA (2020) Fragility fractures in Europe: burden, management and opportunities. Arch Osteoporos 15:59

7. Kanis JA, Oden A, Johnell O, De Laet C, Jonsson B, Oglesby AK (2003) The components of excess mortality after hip fracture. Bone 32:468-473

8. National Clinical Guideline Centre (2011) The Management of Hip Fracture in Adults. In Centre NCG (ed)London

## Epidemiology and economic burden of osteoporosis in Estonia

K Maasalu ∙ E Strauss ∙ C Willers ∙ N Norton ∙ NC Harvey ∙ T Jacobson ∙ H Johansson ∙ M Lorentzon ∙ EV McCloskey ∙ F Borgström ∙ JA Kanis

**Introduction**

The scorecard summarises key indicators of the burden of osteoporosis and its management in the 27 member states of the European Union, as well as the UK and Switzerland (termed EU27+2) [1]. This country-specific report summarises the principal results for Estonia.

**Methods**

The information obtained covers four domains: burden of osteoporosis and fractures; policy framework; service provision; and service uptake. Data were collected from numerous sources including previous research and IOF reports, and available registers which were used for additional analysis of resource utilization, costing and HRQoL data. Furthermore, country-specific information on osteoporosis management was obtained from each IOF member state via a questionnaire.

**Burden of disease**

The direct cost of incident fractures in Estonia in 2019 was €18.1 million. Added to this was the ongoing cost in 2019 from fractures that occurred before 2019, which amounted to €11.9 million (long-term disability). The cost of pharmacological intervention (assessment and treatment) was €1.7 million. Thus, the total direct cost (excluding the value of QALYs lost) amounted to €31.6 million in 2019. Key metrics are presented in Table 1.

In 2019, the average direct cost of osteoporotic fractures in Estonia was €23.9 per individual in the population, while in 2010 the average was €24.3 (after adjusting for inflation). This relative decrease of 1% (€23.9 versus €24.3), makes Estonia one of only a few countries with decreasing costs per capita for osteoporosis. The 2019 numbers put Estonia in 27th place in terms of highest cost of osteoporotic fractures per capita in the EU27+2.

The cost of osteoporotic fractures in Estonia accounted for approximately 2.0% of healthcare spending (i.e. €31.6 million out of €1.5 billion in 2019), somewhat lower than the EU27+2 average of 3.5%. Nonetheless, these numbers indicate a substantial impact of fragility fractures on the healthcare budget.

Using World Health Organization diagnostic criteria for osteoporosis based on the measurement of bone mineral density (BMD) [2], there were approximately 82,000 individuals with osteoporosis in Estonia in 2019, of whom approximately 84% were women. The prevalence of osteoporosis in the total Estonian population amounted to 5.8%, on par with the EU27+2 average (5.6%).

**Table 1** Key measures of burden of disease for Estonia

**Table:** 

**Category**	**Measure**	**Estimate**	**Rank**
Burden of disease	Direct cost of incident fracture (€m)	18.05	
	Long-term disability cost (€m)	11.89	
	Intervention cost (€m)	1.68	
	Total cost (€m)	31.62	
	QALYs lost (€m)	106	
	Cost per capita (€)	23.94	27
	Proportion of healthcare spending	2.0%	26
	Prevalence of osteoporosis	5.8%	4

There were estimated to be 7,900 new fragility fractures in Estonia in 2019, equivalent to 22 fractures/day (or 0.9 per hour). This was a decrease compared to 2010, equivalent to a decrement of 2.8 fractures per 1000 individuals, totalling 15.1 fractures/1000 individuals in 2019.

Some osteoporotic fractures are associated with premature mortality [3]. In Estonia, the annual number of deaths associated with a fracture event was estimated to be 121 per 100,000 individuals of the population aged 50 years or more, compared to the EU27+2 average of 116/100,000. The number of fracture-related deaths is comparable to or exceeds that for some of the most common causes of death such as lung cancer, diabetes, chronic lower respiratory diseases.

The remaining lifetime probability of hip fracture (%) at the ages of 50 years in men and women was 4.4% and 9.1%, respectively, placing Estonia in the lower tertile of risk for both men and women.

The population in men and women age 50 years or more is projected to increase by 7.3% between 2019 and 2034, somewhat lower than the EU27+2 average of 11.4%. The increases in men and women aged 75 years or more are more marked and amount to 41.0% and 11.6%, respectively. The annual number of osteoporotic fractures in Estonia is expected to increase by 1,600 to 9,500 in 2034.

**Policy framework** (Table 2)

Documentation of the burden of disease is an essential prerequisite to determine the resources that should be allocated to the diagnosis and treatment of the disorder. High quality national data on hip fracture rates have been identified in 18 of 29 countries, of which Estonia is one, although there is no established national fracture registry. National data can be extracted, however, from the sole Health Insurance Fund

Given that osteoporosis and fragility fractures are common and that effective treatments are widely available, the vast majority of patients with osteoporosis are preferably managed at the primary health care level by general practitioners (GPs), with specialist referral reserved for difficult complex cases. Primary care was the principal provider of the medical care for osteoporosis in Estonia, as for 13 of the 28 countries where data were available.

Osteoporosis and metabolic bone disease is not a recognised specialty in most countries including Estonia. Specialty care of osteoporosis in Estonia is via other specialties including orthopaedics, gynaecology, endocrinology and rheumatology. Osteoporosis is however recognized as a component of specialty training. As a small country there is only one medical university and all medical education and training is conducted by the same teaching staff including residents and postgraduate training. Thus, training programs are harmonised for all involved specialities trainees.

**Table 2** Policy framework for osteoporosis in Estonia

**Table:** 

**Category**	**Measure**	**Estimate**
Policy framework	National fracture data availability	Can be extracted from Health Fund database.
	OP recognized as a specialty	No
	OP primarily managed in primary care	Yes
	Other specialties involved	Orthopaedics, Gynaecology, Endocrinology, Rheumatology
	Advocacy areas covered by national osteoporosis organisation	None

The role of national patient organisations is to improve the care of patients and increase awareness and prevention of osteoporosis and related fractures among the general public. Advocacy by patient organisations can fall into four categories: policy, capacity building and education, peer support, research and development. For Estonia, none of the advocacy areas were covered. All four advocacy areas were covered in only 10 out of the 26 countries with at least one patient organisation. Osteoporosis related issues are covered by specialty societies.

**Service provision** (Table 3)

A wide variety of approved drug treatments is available for the management of osteoporosis [4]. Potential limitations of their use in member states relate to reimbursement policies which may impair the delivery of health care. 12 out of 27 countries offered full reimbursement, and Estonia was reported to offer partial reimbursement.

The assessment of bone mineral density forms a key component for the general management of osteoporosis, being used for diagnosis, risk prediction, selection of patients for treatment and monitoring of patients on treatment. In Estonia, the number of DXA units expressed per million of the general population amounted to 12.7 which puts the country in the 16th place among the EU27+2.

The average waiting time for DXA ranged from 0 to 180 days across countries, and there was no clear relation between waiting times and the availability of DXA. In Estonia, the estimated average waiting time for DXA amounted to 14 days. Nine countries reported shorter average waiting times.

**Table 3** Service provision for osteoporosis in Estonia

**Table:** 

**Category**	**Measure**	**Estimate**	**Rank**
Service provision	Reimbursement of OP medications	50-100%	
	DXA units/million inhabitants	12.7	16
	DXA cost (€)	25	23
	FRAX risk assessment model available	Yes	
	Fracture liaison service density	No FLS	

Reimbursement for DXA scans varied between member states both in terms of the criteria required and level of reimbursement awarded. In Estonia, the reimbursement was unconditional.

The effective targeting of treatment to those at highest risk of fracture requires an assessment of fracture risk. Risk assessment models for fractures, most usually based on FRAX, were available in 24 out of 29 countries, of which Estonia was one. For Estonia, guidance on the use of risk assessment within national guidelines was not available, as there was in 14 of the other countries.

Guidelines for the management of osteoporosis were available in Estonia (as in 27 out of 29 countries). The guidelines in Estonia included postmenopausal women specifically, as well as osteoporosis in men.

Fracture liaison services (FLS), also known as osteoporosis coordinator programmes and care manager programmes, provide a system for the routine assessment and management of postmenopausal women and older men who have sustained a low trauma fracture. No fracture liaison services were reported from Estonia (together with 7 other countries). Osteoporosis management following a fracture is part of orthopaedic management. Assessment includes access to DXA, bone turnover markers and treatment plans.

The use of indicators to systematically measure the quality of care provided to people with osteoporosis or associated fractures has expanded as a discipline within the past decade [5]. No use of national quality indicators was reported for Estonia.

**Service uptake** (Table 4)

The web-based usage of FRAX showed considerable heterogeneity in uptake between the countries. The average uptake for the EU27+2 was 1,555 sessions/million/year of the general population with an enormous range of 49 to 41,874 sessions/million. The usage for Estonia amounted to 916 sessions/million in 2019, with a 343 percent increase since 2011.

Many studies have demonstrated that a significant proportion of men and women at high fracture risk do not receive therapy for osteoporosis (the treatment gap) [6]. In the EU27+2 the average treatment gap was 71% but ranged from 32% to 87%. For Estonia, the treatment gap amongst women amounted to 84% or 35,000 out of 42,000 characterised at risk. The Estonian treatment gap did not change significantly compared to 2010, whilst the average treatment gap among EU27+2 increased from 55% in 2010 to 71% in 2019.

**Table 4** Service uptake for osteoporosis in Estonia

**Table:** 

**Category**	**Measure**	**Estimate**	**Rank**
Service uptake	Number of FRAX sessions/million people/year	916	15
	Treatment gap for women eligible for treatment (%)	84	26
	Proportion surgically managed hip fractures	>90%	

About 5% of people with a hip fracture die within 1 month of their fracture [7]. A determinant of peri-operative morbidity and mortality is the time a patient takes to get to surgery [8]. For Estonia, the average waiting time for hip fracture surgery after hospital admission was reported to be less than 24 hours. The proportion of surgically managed hip fracture cases was reported to be over 90%, of which 65% underwent osteosynthesis and 35% hip replacement surgery.

**Scores and scorecard**

Scores were developed for Burden of disease and the healthcare provision (Policy framework, Service provision and Service uptake) in the EU27+2 countries. Estonia scores resulted in a 28th place regarding Burden of disease. The combined healthcare provision scorecard resulted in a 25th place for Estonia. Thus, Estonia presents as one of the five low-burden low-provision countries among the EU27+2.

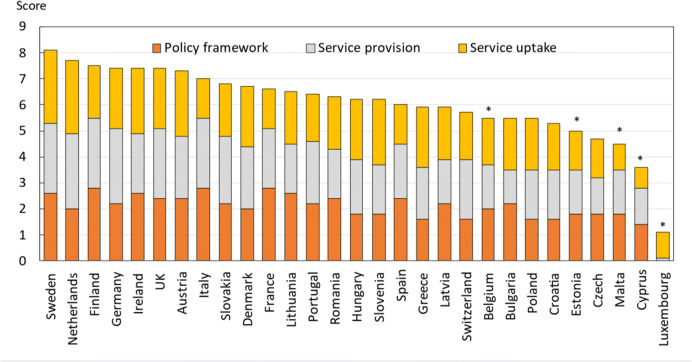



**Fig. 1** Scores by country for metrics related to policy framework, service provision and service uptake. The mean score for each of the 3 domains is given. An asterisk denotes that there was one or more missing metric which decreases the overall score

The first SCOPE was undertaken in 2010, almost 10 years previously. Fifteen of the 16 score card metrics on healthcare provision were used in the two surveys. Scores had improved or markedly improved in 15 countries, remained constant in 8 countries and worsened in 3 countries. For Estonia, the scores were unchanged.

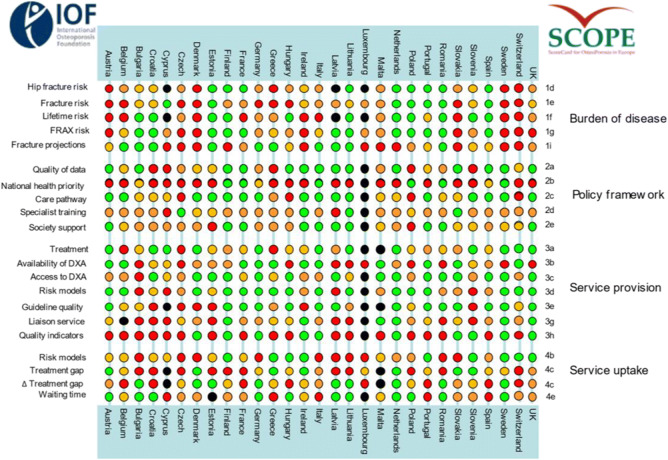



**Fig. 2** The scorecard for all the EU27+2 countries illustrating the scores across the four domains. The elements of each domain in each country were scored and coded using a traffic light system (red, orange, green). Black dots signify missing information

The second edition of the Scorecard for Osteoporosis in Europe (SCOPE 2021) allows health and policy professionals to assess key indicators on the healthcare provision for osteoporosis within countries and between countries within the EU 27+2. The scorecard is not intended as a prescriptive template. Thus, it does not set performance targets but may serve as a guide to the performance targets at which to aim in order to deliver the outcomes required.

**Acknowledgements**

SCOPE was supported by an unrestricted grant from Amgen to the International Osteoporosis Foundation (IOF). Amgen was neither involved in the design nor writing of the report. We are grateful to Anastasia Soulié Mlotek and Dominique Pierroz of the IOF for their help in the administration of SCOPE. The report has been reviewed by the members of the SCOPE Consultation Panel and the relevant IOF National societies, and we are grateful for their local insights on the management of osteoporosis in each country. The source document has been reviewed and endorsed by the Committee of Scientific Advisors of the IOF and benefitted from their feedback.

**References**

1. Kanis JA, Norton N, Harvey NC, Jacobson T, Johansson H, Lorentzon M, McCloskey EV, Willers C, Borgström F (2021) SCOPE 2021: a new scorecard for osteoporosis in Europe. Arch Osteoporos 16:82. 10.1007/s11657-020-00871-9

2. World Health Organisation (1994) Assessment of fracture risk and its application to screening for postmenopausal osteoporosis. Report of a WHO Study Group. World Health Organ Tech Rep Ser, 1994/01/01 edn, pp 1-129

3. Johnell O, Kanis JA, Oden A, Sernbo I, Redlund-Johnell I, Petterson C, De Laet C, Jonsson B (2004) Mortality after osteoporotic fractures. Osteoporos Int 15:38-42

4. Hernlund E, Svedbom A, Ivergard M, Compston J, Cooper C, Stenmark J, McCloskey EV, Jonsson B, Kanis JA (2013) Osteoporosis in the European Union: medical management, epidemiology and economic burden. A report prepared in collaboration with the International Osteoporosis Foundation (IOF) and the European Federation of Pharmaceutical Industry Associations (EFPIA). Arch Osteoporos 8:136

5. Allen P, Pilar M, Walsh-Bailey C, Hooley C, Mazzucca S, Lewis CC, Mettert KD, Dorsey CN, Purtle J, Kepper MM, Baumann AA, Brownson RC (2020) Quantitative measures of health policy implementation determinants and outcomes: a systematic review. Implement Sci 15:47

6. Borgstrom F, Karlsson L, Ortsater G, Norton N, Halbout P, Cooper C, Lorentzon M, McCloskey EV, Harvey NC, Javaid MK, Kanis JA (2020) Fragility fractures in Europe: burden, management and opportunities. Arch Osteoporos 15:59

7. Kanis JA, Oden A, Johnell O, De Laet C, Jonsson B, Oglesby AK (2003) The components of excess mortality after hip fracture. Bone 32:468-473

8. National Clinical Guideline Centre (2011) The Management of Hip Fracture in Adults. In Centre NCG (ed)London

## Epidemiology and economic burden of osteoporosis in Finland

A Holm ∙ C Willers ∙ N Norton ∙ NC Harvey ∙ T Jacobson ∙ H Johansson ∙ M Lorentzon ∙ EV McCloskey ∙ F Borgström ∙ JA Kanis

**Introduction**

The scorecard summarises key indicators of the burden of osteoporosis and its management in the 27 member states of the European Union, as well as the UK and Switzerland (termed EU27+2) [1]. This country-specific report summarises the principal results for Finland.

**Methods**

The information obtained covers four domains: burden of osteoporosis and fractures; policy framework; service provision; and service uptake. Data were collected from numerous sources including previous research and IOF reports, and available registers which were used for additional analysis of resource utilization, costing and HRQoL data. Furthermore, country-specific information on osteoporosis management was obtained from each IOF member state via a questionnaire.

**Burden of disease**

The direct cost of incident fractures in Finland in 2019 was €406.6 million. Added to this was the ongoing cost in 2019 from fractures that occurred before 2019, which amounted to €190.9 million (long-term disability). The cost of pharmacological intervention (assessment and treatment) was €13.6 million. Thus, the total direct cost (excluding the value of QALYs lost) amounted to €611 million in 2019. Key metrics are presented in Table 1.

In 2019, the average direct cost of osteoporotic fractures in Finland was €110.8 per individual in the population, while in 2010 the average was €78.4 (after adjusting for inflation), representing an increase of 41% (€110.8 versus €78.4). The 2019 data rank Finland in 7^th^ place in terms of highest cost of osteoporotic fractures per capita in the EU27+2.

The cost of osteoporotic fractures in Finland accounted for approximately 2.9% of healthcare spending (i.e. €611 million out of €20.8 billion in 2019), lower than the EU27+2 average of 3.5% and placed Finland at 16^th^ place in the ranked order across the EU27+2 countries These data suggest some underinvestment of the healthcare budget in fragility fractures.

Using World Health Organization diagnostic criteria for osteoporosis based on the measurement of bone mineral density (BMD) [2], there were approximately 336,000 individuals with osteoporosis in Finland in 2019, of whom almost 80% were women. The prevalence of osteoporosis in the total Finnish population amounted to 5.7%, on par with the EU27+2 average (5.6%).

**Table 1** Key measures of burden of disease for Finland

**Table:** 

**Category**	**Measure**	**Estimate**	**Rank**
Burden of disease	Direct cost of incident fracture (€m)	406.60	
	Long-term disability cost (€m)	190.90	
	Intervention cost (€m)	13.62	
	Total cost (€m)	611.12	
	QALYs lost (€m)	1423	
	Cost per capita (€)	110.75	7
	Proportion of healthcare spending	2.9%	16
	Prevalence of osteoporosis	5.7%	6

There were estimated to be 45,000 new fragility fractures in Finland in 2019, equivalent to 124 fractures/day (or 5 per hour). This was a slight increase compared to 2010, equivalent to an increment of 2.1 fractures/1000 individuals, totalling 19.5 fractures/ 1000 individuals in 2019.

Some osteoporotic fractures are associated with premature mortality [3]. In Finland, the annual number of deaths associated with a fracture event was estimated to be 112 per 100,000 individuals of the population aged 50 years or more, compared to the EU27+2 average of 116/100,000. The number of fracture-related deaths is comparable to or exceeds that for some of the most common causes of death such as lung cancer, diabetes, chronic lower respiratory diseases.

The remaining lifetime probability of hip fracture (%) at the ages of 50 years in men and women was 5.8% and 12.4%, respectively, placing Finland in the mid tertile of risk for both men and women.

The population in men and women age 50 years or more is projected to increase by 6.9% between 2019 and 2034, somewhat lower than the EU27+2 average of 11.4%. The increases in men and women aged 75 years or more are more marked and amount to 66.7% and 47.8%, respectively. The annual number of osteoporotic fractures in Finland is expected to increase by 15,000 to 60,000 in 2034.

**Policy framework** (Table 2)

Documentation of the burden of disease is an essential prerequisite to determine the resources that should be allocated to the diagnosis and treatment of the disorder. High quality national data on hip fracture rates have been identified in 18 of 29 countries, of which Finland is one. Data are collected on a national basis and include more than only hip fracture data.

Given that osteoporosis and fragility fractures are common and that effective treatments are widely available, the vast majority of patients with osteoporosis are preferably managed at the primary health care level by general practitioners (GPs), with specialist referral reserved for difficult complex cases. Primary care was the principal provider of the medical care for osteoporosis in Finland, as for 13 of the 28 countries where data were available.

Osteoporosis and metabolic bone disease is not a recognised specialty in most countries including Finland. Specialty care of osteoporosis in Finland is managed via other specialties including endocrinology, internal medicine, geriatrics, orthopaedics and gynaecology. Osteoporosis is however recognized as a component of specialty training. Although it is possible that these specialties educate their trainees adequately, the wide variation may reflect inconsistencies in patient care, training of primary care physicians and a suboptimal voice to “defend” the interests of those who work within the field of osteoporosis.

**Table 2** Policy framework for osteoporosis in Finland

**Table:** 

**Category**	**Measure**	**Estimate**
Policy framework	National fracture data availability	Yes
	OP recognized as a specialty	No
	OP primarily managed in primary care	Yes
	Other specialties involved	Endocrinology, Internal medicine, Geriatrics, Orthopaedics, Gynaecology
	Advocacy areas covered by patient organisation	Policy, capacity, peer support, research and development

The role of national patient organisations is to improve the care of patients and increase awareness and prevention of osteoporosis and related fractures among the general public. Advocacy by patient organisations can fall into four categories: policy, capacity building and education, peer support, research and development. For Finland, all four of the advocacy areas were covered by a patient organisation, which was the case for only 10 out of the 26 countries with at least one patient organisation.

**Service provision** (Table 3)

A wide variety of approved drug treatments is available for the management of osteoporosis [4]. Potential limitations of their use in member states relate to reimbursement policies which may impair the delivery of health care. Twelve out of 27 countries offered full reimbursement, and Finland was reported to offer only partial reimbursement.

The assessment of bone mineral density forms a key component for the general management of osteoporosis, being used for diagnosis, risk prediction, selection of patients for treatment and monitoring of patients on treatment. In Finland, the number of DXA units expressed per million of the general population amounted to 11.6 which puts the country in 18^th^ place among the EU27+2.

The average waiting time for DXA ranged from 0 to 180 days across countries, and there was no clear relation between waiting times and the availability of DXA. In Finland, the estimated average waiting time for DXA amounted to 30 days. 17 countries reported shorter average waiting times.

**Table 3** Service provision for osteoporosis in Finland

**Table:** 

**Category**	**Measure**	**Estimate**	**Rank**
Service provision	Reimbursement of OP medications	40%	
	DXA units/million inhabitants	11.6	18
	DXA cost (€)	200	1
	FRAX risk assessment model available	Yes	
	Fracture liaison service density	25-50%	

Reimbursement for DXA scans varied between member states both in terms of the criteria required and level of reimbursement awarded. In Finland, the reimbursement was unconditional.

The effective targeting of treatment to those at highest risk of fracture requires an assessment of fracture risk. Risk assessment models for fractures, most usually based on FRAX, were available in 24 out of 29 countries, of which Finland was one. For Finland, guidance on the use of risk assessment within national guidelines was available, as in only 14 of the other countries.

Guidelines for the management of osteoporosis were available in Finland (as in 27 out of 29 countries). The guidelines in Finland included postmenopausal women specifically, as well as osteoporosis in men and secondary osteoporosis including glucocorticoid-induced osteoporosis.

Fracture liaison services (FLS), also known as osteoporosis coordinator programmes and care manager programmes, provide a system for the routine assessment and management of postmenopausal women and older men who have sustained a low trauma fracture. Fracture liaison services were reported for 25-50% of hospitals in Finland.

The use of indicators to systematically measure the quality of care provided to people with osteoporosis or associated fractures has expanded as a discipline within the past decade [5]. Finland stood out as one country of only a few with national quality indicators.

**Service uptake** (Table 4)

The web-based usage of FRAX showed considerable heterogeneity in uptake between the countries. The average uptake for the EU27+2 was 1,555 sessions/million/year of the general population with an enormous range of 49 to 41,874 sessions/million. The use of FRAX for Finland amounted to 4,343 sessions/million in 2019, with an increase of almost 900 percent increase since 2011.

Many studies have demonstrated that a significant proportion of men and women at high fracture risk do not receive therapy for osteoporosis (the treatment gap) [6]. In the EU27+2 the average treatment gap was 71% but ranged from 32% to 87%. For Finland, the treatment gap amongst women amounted to 80% or 154,000 out of 193,000 characterised at risk and it increased compared to 2010. The average treatment gap among EU27+2 increased from 55% in 2010 to 71% in 2019.

**Table 4** Service uptake for osteoporosis in Finland

**Table:** 

**Category**	**Measure**	**Estimate**	**Rank**
Service uptake	Number of FRAX sessions/million people/year	4343	5
	Treatment gap for women eligible for treatment (%)	80	21
	Proportion surgically managed hip fractures	>90%	

About 5% of people with a hip fracture die within 1 month of their fracture [7]. A determinant of peri-operative morbidity and mortality is the time a patient takes to get to surgery [8]. For Finland, the average waiting time for hip fracture surgery after hospital admission was reported to be 1-2 days. The proportion of surgically managed hip fractures was reported to be over 90%.

**Scores and scorecard**

Scores were developed for Burden of disease and the healthcare provision (Policy framework, Service provision and Service uptake) in the EU27+2 countries. Finland scores resulted in a 17th place regarding Burden of disease. The combined healthcare provision scorecard resulted in a 3rd place for Finland after only Sweden and Netherlands. Thus, Finland presents as one of the low-burden high-provision countries among the EU27+2.

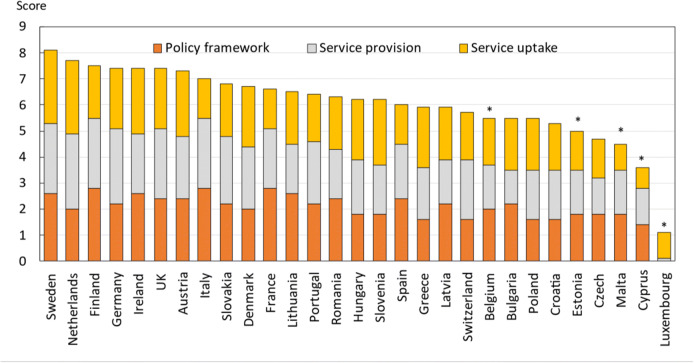



**Fig. 1** Scores by country for metrics related to policy framework, service provision and service uptake. The mean score for each of the 3 domains is given. An asterisk denotes that there was one or more missing metric which decreases the overall score

The first SCOPE was undertaken in 2010, almost 10 years previously. Fifteen of the 16 score card metrics on healthcare provision were used in the two surveys. Scores had improved or markedly improved in 15 countries, remained constant in 8 countries and worsened in 3 countries. For Finland the scores were much improved.

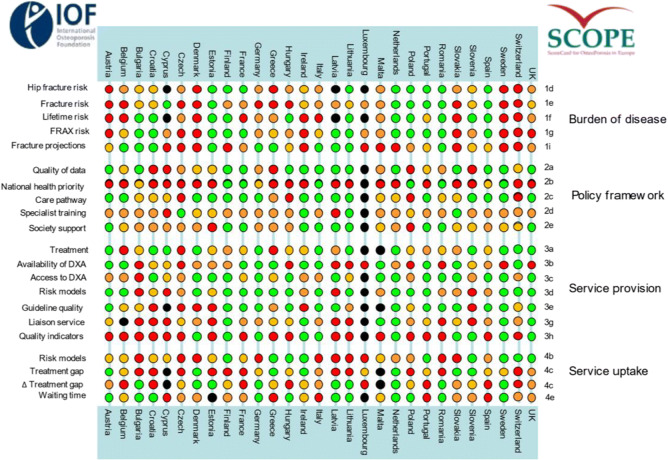



**Fig. 2** The scorecard for all the EU27+2 countries illustrating the scores across the four domains. The elements of each domain in each country were scored and coded using a traffic light system (red, orange, green). Black dots signify missing information

The second edition of the Scorecard for Osteoporosis in Europe (SCOPE 2021) allows health and policy professionals to assess key indicators on the healthcare provision for osteoporosis within countries and between countries within the EU 27+2. The scorecard is not intended as a prescriptive template. Thus, it does not set performance targets but may serve as a guide to the performance targets at which to aim in order to deliver the outcomes required.

**Acknowledgements**

SCOPE was supported by an unrestricted grant from Amgen to the International Osteoporosis Foundation (IOF). Amgen was neither involved in the design nor writing of the report. We are grateful to Anastasia Soulié Mlotek and Dominique Pierroz of the IOF for their help in the administration of SCOPE. The report has been reviewed by the members of the SCOPE Consultation Panel and the relevant IOF National societies, and we are grateful for their local insights on the management of osteoporosis in each country. The source document has been reviewed and endorsed by the Committee of Scientific Advisors of the IOF and benefitted from their feedback.

**References**

1. Kanis JA, Norton N, Harvey NC, Jacobson T, Johansson H, Lorentzon M, McCloskey EV, Willers C, Borgström F (2021) SCOPE 2021: a new scorecard for osteoporosis in Europe. Arch Osteoporos 16:82. 10.1007/s11657-020-00871-9

2. World Health Organisation (1994) Assessment of fracture risk and its application to screening for postmenopausal osteoporosis. Report of a WHO Study Group. World Health Organ Tech Rep Ser, 1994/01/01 edn, pp 1-129

3. Johnell O, Kanis JA, Oden A, Sernbo I, Redlund-Johnell I, Petterson C, De Laet C, Jonsson B (2004) Mortality after osteoporotic fractures. Osteoporos Int 15:38-42

4. Hernlund E, Svedbom A, Ivergard M, Compston J, Cooper C, Stenmark J, McCloskey EV, Jonsson B, Kanis JA (2013) Osteoporosis in the European Union: medical management, epidemiology and economic burden. A report prepared in collaboration with the International Osteoporosis Foundation (IOF) and the European Federation of Pharmaceutical Industry Associations (EFPIA). Arch Osteoporos 8:136

5. Allen P, Pilar M, Walsh-Bailey C, Hooley C, Mazzucca S, Lewis CC, Mettert KD, Dorsey CN, Purtle J, Kepper MM, Baumann AA, Brownson RC (2020) Quantitative measures of health policy implementation determinants and outcomes: a systematic review. Implement Sci 15:47

6. Borgstrom F, Karlsson L, Ortsater G, Norton N, Halbout P, Cooper C, Lorentzon M, McCloskey EV, Harvey NC, Javaid MK, Kanis JA (2020) Fragility fractures in Europe: burden, management and opportunities. Arch Osteoporos 15:59

7. Kanis JA, Oden A, Johnell O, De Laet C, Jonsson B, Oglesby AK (2003) The components of excess mortality after hip fracture. Bone 32:468-473

8. National Clinical Guideline Centre (2011) The Management of Hip Fracture in Adults. In Centre NCG (ed) London

## Epidemiology and economic burden of osteoporosis in France

F Alliot-Launois ∙ B Cortet ∙ L Grange ∙ T Thomas ∙ C Willers ∙ N Norton ∙ NC Harvey ∙ T Jacobson ∙ H Johansson ∙ M Lorentzon ∙ EV McCloskey ∙ F Borgström ∙ JA Kanis

**Introduction**

The scorecard summarises key indicators of the burden of osteoporosis and its management in the 27 member states of the European Union, as well as the UK and Switzerland (termed EU27+2) [1]. This country-specific report summarises the principal results for France.

**Methods**

The information obtained covers four domains: burden of osteoporosis and fractures; policy framework; service provision; and service uptake. Data were collected from numerous sources including previous research and IOF reports, and available registers which were used for additional analysis of resource utilization, costing and HRQoL data. Furthermore, country-specific information on osteoporosis management was obtained from each IOF member state via a questionnaire.

**Burden of disease**

The direct cost of incident fractures in France in 2019 was €5.05 billion. Added to this was the ongoing cost in 2019 from fractures that occurred before 2019, which amounted to €1.77 billion (long-term disability). The cost of pharmacological intervention (assessment and treatment) was €162 million. Thus, the total direct cost (excluding the value of QALYs lost) amounted to €6.98 billion in 2019. Key metrics are presented in Table 1.

In 2019, the average direct cost of osteoporotic fractures in France was €104.2 per individual in the population, while in 2010 the average was €85.0 (after adjusting for inflation), representing an increase of 23% (€104.2 versus €85.0). The 2019 data rank France in the 8th place in terms of highest cost of osteoporotic fractures per capita in the EU27+2.

The cost of osteoporotic fractures in France accounted for approximately 2.6% of healthcare spending (i.e. €6.98 billion out of €262 billion in 2019), below the EU27+2 average of 3.5%. These numbers indicate an underinvestment of the healthcare budget in osteoporosis.

Using World Health Organization diagnostic criteria for osteoporosis based on the measurement of bone mineral density (BMD) [2], there were approximately 4,000,000 individuals with osteoporosis in France in 2019, of whom approximately 80% were women. The prevalence of osteoporosis in the total French population amounted to 5.5%, on par with the EU27+2 average (5.6%).

**Table 1** Key measures of burden of disease for France

**Table:** 

**Category**	**Measure**	**Estimate**	**Rank**
Burden of disease	Direct cost of incident fracture (€m)	5047.97	
	Long-term disability cost (€m)	1769.89	
	Intervention cost (€m)	162.22	
	Total cost (€m)	6980.07	
	QALYs lost (€m)	12001	
	Cost per capita (€)	104.2	8
	Proportion of healthcare spending	2.6%	19
	Prevalence of osteoporosis	5.5%	14

There were estimated to be 484,000 new fragility fractures in France in 2019, equivalent to 1,325 fractures/day (or 55 per hour). This was a slight increase compared to 2010, equivalent to an increment of 1.9 fractures/1000 individuals, totalling 18.5 fractures/ 1000 individuals in 2019.

Some osteoporotic fractures are associated with premature mortality [3]. In France, the annual number of deaths associated with a fracture event was estimated to be 77 per 100,000 individuals of the population aged 50 years or more, compared to the EU27+2 average of 116/100,000. The number of fracture-related deaths is comparable to or exceeds some of the most common causes of death such as lung cancer, diabetes, chronic lower respiratory diseases, stroke and cardio-vascular diseases.

The remaining lifetime probability of hip fracture (%) at the ages of 50 years in men and women was 5.6% and 18.4%, respectively, placing France in the mid tertile of risk for men and the upper risk tertile for women.

The population in men and women age 50 years or more is projected to increase by 11.8% between 2019 and 2034, close to the EU27+2 average of 11.4%. The increases in men and women aged 75 years or more are even more marked and amount to 57.0% and 41.8%, respectively. The annual number of osteoporotic fractures in France is expected to increase by 126,000 to 610,000 in 2034.

**Policy framework** (Table 2)

Documentation of the burden of disease is an essential prerequisite to determine the resources that should be allocated to the diagnosis and treatment of the disorder. High quality national data on hip fracture rates have been identified in 18 of 29 countries, of which France is one. Data are collected on a national basis and include hip fracture data.

Given that osteoporosis and fragility fractures are common and that effective treatments are widely available, the vast majority of patients with osteoporosis are preferably managed at the primary health care level by general practitioners (GPs), with specialist referral reserved for difficult complex cases. Primary care was the principal provider of the medical care for osteoporosis in France, as for 13 of the 28 countries where data were available.

Osteoporosis and metabolic bone disease is not a recognised specialty in most countries including France. Specialty care of osteoporosis in France is via other specialties, mainly rheumatology but also geriatrics, endocrinology, gynaecology, orthopaedics and internal medicine. Osteoporosis is however recognized as a component of specialty training. Although it is possible that these specialties educate their trainees adequately, the wide variation may reflect inconsistencies in patient care, training of primary care physicians and a suboptimal voice to “defend” the interests of those who work within the field of osteoporosis.

**Table 2** Policy framework for osteoporosis in France

**Table:** 

**Category**	**Measure**	**Estimate**
Policy framework	National fracture data availability	Yes
	OP recognized as a specialty	No
	OP primarily managed in primary care	Yes
	Other specialties involved	Rheumatology, Geriatrics, Endocrinology, Gynaecology, Orthopaedics, Internal medicine
	Advocacy areas covered by patient organisation	Policy, capacity, peer support, research and development

The role of national patient organisations is to improve the care of patients and increase awareness and prevention of osteoporosis and related fractures among the general public. Advocacy by patient organisations can fall into four categories: policy, capacity building and education, peer support, research and development. For France, all four of the advocacy areas were covered by a patient organisation, which was the case for only 10 out of the 26 countries with at least one patient organisation.

**Service provision** (Table 3)

A wide variety of approved drug treatments is available for the management of osteoporosis [4]. Potential limitations of their use in member states relate to reimbursement policies which may impair the delivery of health care. 12 out of 27 countries offered full reimbursement of which France was not one.

The assessment of bone mineral density forms a key component for the general management of osteoporosis, being used for diagnosis, risk prediction, selection of patients for treatment and monitoring of patients on treatment. In France, the number of DXA units expressed per million of the general population amounted to 23.8 which puts the country in 8^th^ place among the EU27+2. Furthermore, the availability of TBS was amongst the highest in France.

The average waiting time for DXA ranged from 0 to 180 days across countries, and there was no clear relation between waiting times and the availability of DXA. In France, the estimated average waiting time for DXA amounted to 30 days. 17 countries reported shorter average waiting times.

**Table 3** Service provision for osteoporosis in France

**Table:** 

**Category**	**Measure**	**Estimate**	**Rank**
Service provision	Reimbursement of OP medications	65%	
	DXA units/million inhabitants	23.8	8
	DXA cost (€)	40	17
	FRAX risk assessment model available	Yes	
	Fracture liaison service density	10-25%	

Reimbursement for DXA scans varied between member states both in terms of the criteria required and level of reimbursement awarded. In France, the reimbursement was conditional and varied depending on the patient’s condition.

The effective targeting of treatment to those at highest risk of fracture requires an assessment of fracture risk. Risk assessment models for fractures, most usually based on FRAX, were available in 24 out of 29 countries, of which France was one. For France, guidance on the use of risk assessment within national guidelines was available, as in only 14 of the other countries.

Guidelines for the management of osteoporosis were available in France (as in 27 out of 29 countries). The guidelines in France included postmenopausal women specifically, as well as osteoporosis in men and secondary osteoporosis including glucocorticoid-induced osteoporosis.

Fracture liaison services (FLS), also known as osteoporosis coordinator programmes and care manager programmes, provide a system for the routine assessment and management of postmenopausal women and older men who have sustained a low trauma fracture. Fracture liaison services were reported for 10-25% of the hospitals in France but considered by others to be optimistic and closer to 2-3%.

The use of indicators to systematically measure the quality of care provided to people with osteoporosis or associated fractures has expanded as a discipline within the past decade [5]. No use of national quality indicators was reported for France.

**Service uptake** (Table 4)

The web-based usage of FRAX showed considerable heterogeneity in uptake between the countries. The average uptake for the EU27+2 was 1,555 sessions/million/year of the general population with an enormous range of 49 to 41,874 sessions/million. The use of FRAX in France amounted to 676 sessions/million in 2019, with a 115 percent increase since 2011.

Many studies have demonstrated that a significant proportion of men and women at high fracture risk do not receive therapy for osteoporosis (the treatment gap) [6]. In the EU27+2 the average treatment gap was 71% but ranged from 32% to 87%. For France, the treatment gap amongst women amounted to 79% or 2,019,000 out of 2,569,000 characterised at risk and it grew almost 35% compared to 2010. The average treatment gap among EU27+2 increased from 55% in 2010 to 71% in 2019.

**Table 4** Service uptake for osteoporosis in France

**Table:** 

**Category**	**Measure**	**Estimate**	**Rank**
Service uptake	Number of FRAX sessions/million people/year	676	16
	Treatment gap for women eligible for treatment (%)	79	20
	Proportion surgically managed hip fractures	>90%	

About 5% of people with a hip fracture die within 1 month of their fracture [7]. A determinant of peri-operative morbidity and mortality is the time a patient takes to get to surgery [8]. For France, the average waiting time for hip fracture surgery after hospital admission was reported to be 1–2 days. The proportion of surgically managed hip fractures was reported to be over 90%.

**Scores and scorecard**

Scores were developed for Burden of disease and the healthcare provision (Policy framework, Service provision and Service uptake) in the EU27+2 countries. France scores resulted in a 15th place regarding Burden of disease. The combined healthcare provision scorecard resulted in a 11th place for France. Thus, France presents as one of the low-burden high-provision countries among the EU27+2.

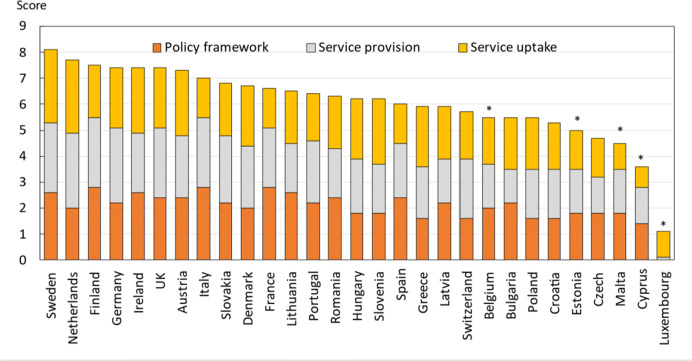



**Fig. 1** Scores by country for metrics related to policy framework, service provision and service uptake. The mean score for each of the 3 domains is given. An asterisk denotes that there was one or more missing metric which decreases the overall score

The first SCOPE was undertaken in 2010, almost 10 years previously. Fifteen of the 16 score card metrics on healthcare provision were used in the two surveys. Scores had improved or markedly improved in 15 countries, remained constant in 8 countries and worsened in 3 countries. For France, the scores were much improved.

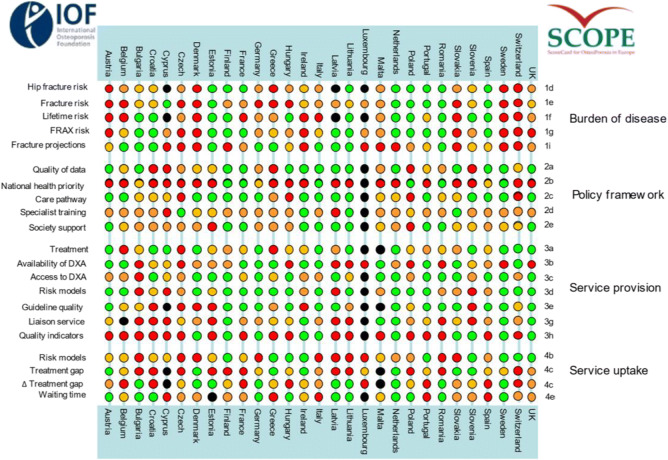



**Fig. 2** The scorecard for all the EU27+2 countries illustrating the scores across the four domains. The elements of each domain in each country were scored and coded using a traffic light system (red, orange, green). Black dots signify missing information

The second edition of the Scorecard for Osteoporosis in Europe (SCOPE 2021) allows health and policy professionals to assess key indicators on the healthcare provision for osteoporosis within countries and between countries within the EU 27+2. The scorecard is not intended as a prescriptive template. Thus, it does not set performance targets but may serve as a guide to the performance targets at which to aim in order to deliver the outcomes required.

**Acknowledgements**

SCOPE was supported by an unrestricted grant from Amgen to the International Osteoporosis Foundation (IOF). Amgen was neither involved in the design nor writing of the report. We are grateful to Anastasia Soulié Mlotek and Dominique Pierroz of the IOF for their help in the administration of SCOPE. The report has been reviewed by the members of the SCOPE Consultation Panel and the relevant IOF National societies, and we are grateful for their local insights on the management of osteoporosis in each country. The source document has been reviewed and endorsed by the Committee of Scientific Advisors of the IOF and benefitted from their feedback.

**References**

1. Kanis JA, Norton N, Harvey NC, Jacobson T, Johansson H, Lorentzon M, McCloskey EV, Willers C, Borgström F (2021) SCOPE 2021: a new scorecard for osteoporosis in Europe. Arch Osteoporos in press 16:82. 10.1007/s11657-020-00871-9

2. World Health Organisation (1994) Assessment of fracture risk and its application to screening for postmenopausal osteoporosis. Report of a WHO Study Group. World Health Organ Tech Rep Ser, 1994/01/01 edn, pp 1-129

3. Johnell O, Kanis JA, Oden A, Sernbo I, Redlund-Johnell I, Petterson C, De Laet C, Jonsson B (2004) Mortality after osteoporotic fractures. Osteoporos Int 15:38-42

4. Hernlund E, Svedbom A, Ivergard M, Compston J, Cooper C, Stenmark J, McCloskey EV, Jonsson B, Kanis JA (2013) Osteoporosis in the European Union: medical management, epidemiology and economic burden. A report prepared in collaboration with the International Osteoporosis Foundation (IOF) and the European Federation of Pharmaceutical Industry Associations (EFPIA). Arch Osteoporos 8:136

5. Allen P, Pilar M, Walsh-Bailey C, Hooley C, Mazzucca S, Lewis CC, Mettert KD, Dorsey CN, Purtle J, Kepper MM, Baumann AA, Brownson RC (2020) Quantitative measures of health policy implementation determinants and outcomes: a systematic review. Implement Sci 15:47

6. Borgstrom F, Karlsson L, Ortsater G, Norton N, Halbout P, Cooper C, Lorentzon M, McCloskey EV, Harvey NC, Javaid MK, Kanis JA (2020) Fragility fractures in Europe: burden, management and opportunities. Arch Osteoporos 15:59

7. Kanis JA, Oden A, Johnell O, De Laet C, Jonsson B, Oglesby AK (2003) The components of excess mortality after hip fracture. Bone 32:468-473

8. National Clinical Guideline Centre (2011) The Management of Hip Fracture in Adults. In Centre NCG (ed) London

## Epidemiology and economic burden of osteoporosis in Germany

AA. Kurth, ∙ S Scharla ∙ G Klatt ∙ C Willers ∙ N Norton ∙ NC Harvey ∙ T Jacobson ∙ H Johansson ∙ M Lorentzon ∙ EV McCloskey ∙ F Borgström ∙ JA Kanis

**Introduction**

The scorecard summarises key indicators of the burden of osteoporosis and its management in the 27 member states of the European Union, as well as the UK and Switzerland (termed EU27+2) [1]. This country-specific report summarises the principal results for Germany.

**Methods**

The information obtained covers four domains: burden of osteoporosis and fractures; policy framework; service provision; and service uptake. Data were collected from numerous sources including previous research and IOF reports, and available registers which were used for additional analysis of resource utilization, costing and HRQoL data. Furthermore, country-specific information on osteoporosis management was obtained from each IOF member state via a questionnaire.

**Burden of disease**

The direct cost of incident fractures in Germany in 2019 was €10.24 billion. Added to this was the ongoing cost in 2019 from fractures that occurred before 2019, which amounted to €3.35 billion (long-term disability). The cost of pharmacological intervention (assessment and treatment) was €249 million. Thus, the total direct cost (excluding the value of QALYs lost) amounted to €13.83 billion in 2019. Key metrics are presented in Table 1.

In 2019, the average direct cost of osteoporotic fractures in Germany was €166.8 per individual in the population, while in 2010 the average was €121.4 (after adjusting for inflation), representing an increase of 37% (€166.8 versus €121.4). The 2019 data rank Germany in 4^th^ place in terms of highest cost of osteoporotic fractures per capita in the EU27+2.

The cost of osteoporotic fractures in Germany accounted for approximately 3.7% of healthcare spending (i.e. €13.8 billion out of €371.4 billion in 2019), close to the EU27+2 average of 3.5%. These numbers indicate a substantial impact of fragility fractures on the healthcare budget.

Using World Health Organization diagnostic criteria for osteoporosis based on the measurement of bone mineral density (BMD) [2], there were approximately 5,659,000 individuals with osteoporosis in Germany in 2019, of whom almost 80% were women. The prevalence of osteoporosis in the total German population amounted to 6.1%, on par with the EU27+2 average (5.6%).

**Table 1** Key measures of burden of disease for Germany

**Table:** 

**Category**	**Measure**	**Estimate**	**Rank**
Burden of disease	Direct cost of incident fracture (€m)	10235.08	
	Long-term disability cost (€m)	3345.62	
	Intervention cost (€m)	249.36	
	Total cost (€m)	13830.06	
	QALYs lost (€m)	28 232	
	Cost per capita (€)	166.77	4
	Proportion of healthcare spending	3.7%	12
	Prevalence of osteoporosis	6.1%	2

There were estimated to be 831,000 new fragility fractures in Germany in 2019, equivalent to 2,300 fractures/day (or 95 per hour). This was a modest increase compared to 2010, equivalent to an increment of 0.2 fractures/1000 individuals, totalling 22.2 fractures/ 1000 individuals in 2019.

Some osteoporotic fractures are associated with premature mortality [3]. In Germany, the annual number of deaths associated with a fracture event was estimated to be 130 per 100,000 individuals of the population aged 50 years or more, compared to the EU27+2 average of 116/100,000. The number of fracture-related deaths is comparable to or exceeds that for some of the most common causes of death such as lung cancer, diabetes, chronic lower respiratory diseases.

The remaining lifetime probability of hip fracture (%) at the ages of 50 years in men and women was 5.3% and 14.2%, respectively, placing Germany in the middle tertile of risk for both men and women.

The population in men and women age 50 years or more is projected to increase by 3.5% between 2019 and 2034, significantly lower than the EU27+2 average of 11.4%. The increases in men and women aged 75 years or more are more marked and amount to 25.0% and 13.1%, respectively. The annual number of osteoporotic fractures in Germany is expected to increase by 136,000 to 967,000 in 2034.

**Policy framework** (Table 2)

Documentation of the burden of disease is an essential prerequisite to determine the resources that should be allocated to the diagnosis and treatment of the disorder. High quality national data on hip fracture rates have been identified in 18 of 29 countries, of which Germany is one. Data are collected on a national basis and include more than only hip fracture data.

Given that osteoporosis and fragility fractures are common and that effective treatments are widely available, the vast majority of patients with osteoporosis are preferably managed at the primary health care level by general practitioners (GPs), with specialist referral reserved for difficult complex cases. Primary care was the principal provider of the medical care for osteoporosis in Germany, as for 13 of the 28 countries where data were available.

Osteoporosis and metabolic bone disease is not a recognised specialty in most countries including Germany. Specialty care of osteoporosis in Germany is managed via other specialties including osteology, orthopaedics, gynaecology, rheumatology and endocrinology. Osteoporosis is however recognized as a component of specialty training. Although it is possible that these specialties educate their trainees adequately, the wide variation may reflect inconsistencies in patient care, training of primary care physicians and a suboptimal voice to “defend” the interests of those who work within the field of osteoporosis.

**Table 2** Policy framework for osteoporosis in Germany

**Table:** 

**Category**	**Measure**	**Estimate**
Policy framework	National fracture data availability	No
	OP recognized as a specialty	No
	OP primarily managed in primary care	Yes
	Other specialties involved	Osteology, Orthopaedics, Gynaecology, Rheumatology, Endocrinology
	Advocacy areas covered by patient organisation	Policy, capacity, peer support

The role of national patient organisations is to improve the care of patients and increase awareness and prevention of osteoporosis and related fractures among the general public. Advocacy by patient organisations can fall into four categories: policy, capacity building and education, peer support, research and development. For Germany, three of these advocacy areas were covered by a patient organisation. All advocacy areas were covered for only 10 out of the 26 countries with at least one patient organisation.

**Service provision** (Table 3)

A wide variety of approved drug treatments is available for the management of osteoporosis [4]. Potential limitations of their use in member states relate to reimbursement policies which may impair the delivery of health care. Germany is one of the 12 (out of 27) countries that offer full reimbursement.

The assessment of bone mineral density forms a key component for the general management of osteoporosis, being used for diagnosis, risk prediction, selection of patients for treatment and monitoring of patients on treatment. In Germany, the number of DXA units expressed per million of the general population amounted to 21.5 which puts the country in 10^th^ place among the EU27+2.

The average waiting time for DXA ranged from 0 to 180 days across countries, and there was no clear relation between waiting times and the availability of DXA. In Germany, the estimated average waiting time for DXA amounted to 0 days. Only one other country (Romania) reported such a short waiting times.

**Table 3** Service provision for osteoporosis in Germany

**Table:** 

**Category**	**Measure**	**Estimate**	**Rank**
Service provision	Reimbursement of OP medications	100%	
	DXA units/million inhabitants	21.5	10
	DXA cost (€)	45	16
	FRAX risk assessment model available	Yes	
	Fracture liaison service density	1-10%	

Reimbursement for DXA scans varied between member states both in terms of the criteria required and level of reimbursement awarded. In Germany, the reimbursement was unconditional.

The effective targeting of treatment to those at highest risk of fracture requires an assessment of fracture risk. Risk assessment models for fractures, most usually based on FRAX, were available in 24 out of 29 countries, of which Germany was one. An additional risk assessment model, DVO, was also used in Germany. For Germany, guidance on the use of risk assessment within national guidelines was available, as in only 14 of the other countries.

Guidelines for the management of osteoporosis were available in Germany (as in 27 out of 29 countries). The guidelines in Germany included postmenopausal women specifically, as well as osteoporosis in men and secondary osteoporosis including glucocorticoid-induced osteoporosis.

Fracture liaison services (FLS), also known as osteoporosis coordinator programmes and care manager programmes, provide a system for the routine assessment and management of postmenopausal women and older men who have sustained a low trauma fracture. Fracture liaison services were reported for 1–10% of hospitals in Germany.

The use of indicators to systematically measure the quality of care provided to people with osteoporosis or associated fractures has expanded as a discipline within the past decade [5]. Germany was one of only a few countries reporting national quality indicators.

**Service uptake** (Table 4)

The web-based usage of FRAX showed considerable heterogeneity in uptake between the countries. The average uptake for the EU27+2 was 1,555 sessions/million/year of the general population with an enormous range of 49 to 41,874 sessions/million. The use of FRAX in Germany amounted to 93 sessions/million in 2019, with an 11 percent decrease since 2011. It is notable, however, that Germany has its own assessment guidelines that are widely used [6].

Many studies have demonstrated that a significant proportion of men and women at high fracture risk do not receive therapy for osteoporosis (the treatment gap) [7]. In the EU27+2 the average treatment gap was 71% but ranged from 32% to 87%. For Germany, the treatment gap amongst women amounted to 76% or 2,477,000 out of 3,238,000 characterised at risk. The treatment gap in Germany treatment was similar to that for 2010, whilst the average treatment gap among EU27+2 increased from 55% in 2010 to 71% in 2019.

**Table 4** Service uptake for osteoporosis in Germany

**Table:** 

**Category**	**Measure**	**Estimate**	**Rank**
Service uptake	Number of FRAX sessions/million people/year	93	28
	Treatment gap for women eligible for treatment (%)	76	16
	Proportion surgically managed hip fractures	>90%	

About 5% of people with a hip fracture die within 1 month of their fracture [8]. A determinant of peri-operative morbidity and mortality is the time a patient takes to get to surgery [9]. For Germany, the average waiting time for hip fracture surgery after hospital admission was reported to be less than 24 h. The proportion of surgically managed hip fractures was reported to be over 90%.

**Scores and scorecard**

Scores were developed for Burden of disease and the healthcare provision (Policy framework, Service provision and Service uptake) in the EU27+2 countries. Germany scores resulted in a 12th place regarding Burden of disease. The combined healthcare provision scorecard resulted in a 4th place for Germany after only Sweden, Netherlands and Finland. Thus, Germany presents as one of the high-burden high-provision countries among the EU27+2.

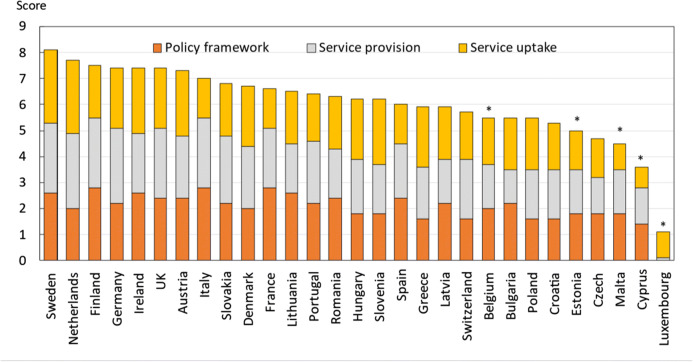



**Fig. 1** Scores by country for metrics related to policy framework, service provision and service uptake. The mean score for each of the 3 domains is given. An asterisk denotes that there was one or more missing metric which decreases the overall score

The first SCOPE was undertaken in 2010, almost 10 years previously. Fifteen of the 16 score card metrics on healthcare provision were used in the two surveys. Scores had improved or markedly improved in 15 countries, remained constant in 8 countries and worsened in 3 countries. For Germany the scores were much improved.

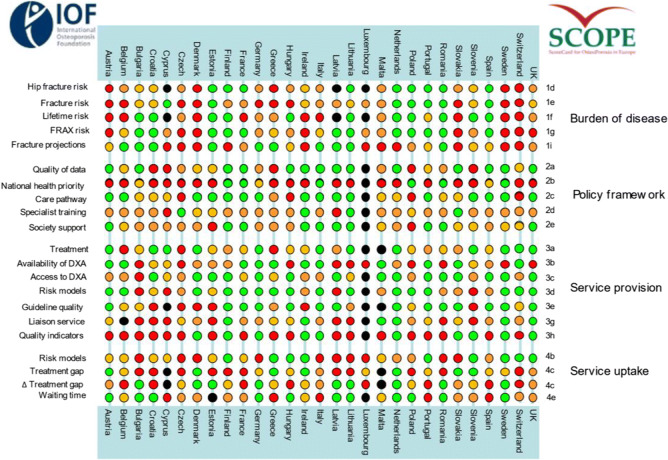



**Fig. 2** The scorecard for all the EU27+2 countries illustrating the scores across the four domains. The elements of each domain in each country were scored and coded using a traffic light system (red, orange, green). Black dots signify missing information

The second edition of the Scorecard for Osteoporosis in Europe (SCOPE 2021) allows health and policy professionals to assess key indicators on the healthcare provision for osteoporosis within countries and between countries within the EU 27+2. The scorecard is not intended as a prescriptive template. Thus, it does not set performance targets but may serve as a guide to the performance targets at which to aim in order to deliver the outcomes required.

**Acknowledgements**

SCOPE was supported by an unrestricted grant from Amgen to the International Osteoporosis Foundation (IOF). Amgen was neither involved in the design nor writing of the report. We are grateful to Anastasia Soulié Mlotek and Dominique Pierroz of the IOF for their help in the administration of SCOPE. The report has been reviewed by the members of the SCOPE Consultation Panel and the relevant IOF National societies, and we are grateful for their local insights on the management of osteoporosis in each country. The source document has been reviewed and endorsed by the Committee of Scientific Advisors of the IOF and benefitted from their feedback.

**References**

1. Kanis JA, Norton N, Harvey NC, Jacobson T, Johansson H, Lorentzon M, McCloskey EV, Willers C, Borgström F (2021) SCOPE 2021: a new scorecard for osteoporosis in Europe. Arch Osteoporos 16:82. 10.1007/s11657-020-00871-9

2. World Health Organisation (1994) Assessment of fracture risk and its application to screening for postmenopausal osteoporosis. Report of a WHO Study Group. World Health Organ Tech Rep Ser, 1994/01/01 edn, pp 1-129

3. Johnell O, Kanis JA, Oden A, Sernbo I, Redlund-Johnell I, Petterson C, De Laet C, Jonsson B (2004) Mortality after osteoporotic fractures. Osteoporos Int 15:38-42

4. Hernlund E, Svedbom A, Ivergard M, Compston J, Cooper C, Stenmark J, McCloskey EV, Jonsson B, Kanis JA (2013) Osteoporosis in the European Union: medical management, epidemiology and economic burden. A report prepared in collaboration with the International Osteoporosis Foundation (IOF) and the European Federation of Pharmaceutical Industry Associations (EFPIA). Arch Osteoporos 8:136

5. Allen P, Pilar M, Walsh-Bailey C, Hooley C, Mazzucca S, Lewis CC, Mettert KD, Dorsey CN, Purtle J, Kepper MM, Baumann AA, Brownson RC (2020) Quantitative measures of health policy implementation determinants and outcomes: a systematic review. Implement Sci 15:47

6. Pfeilschifter J, Kurth AA (2011) DVO Guideline 2009 for Prevention, Diagnosis and Therapy of Osteoporosis in Adults Full-Text Version. Osteologie/Osteology 20:55

7. Borgstrom F, Karlsson L, Ortsater G, Norton N, Halbout P, Cooper C, Lorentzon M, McCloskey EV, Harvey NC, Javaid MK, Kanis JA (2020) Fragility fractures in Europe: burden, management and opportunities. Arch Osteoporos 15:59

8. Kanis JA, Oden A, Johnell O, De Laet C, Jonsson B, Oglesby AK (2003) The components of excess mortality after hip fracture. Bone 32:468-473

9. National Clinical Guideline Centre (2011) The Management of Hip Fracture in Adults. In Centre NCG (ed) London

## Epidemiology and economic burden of osteoporosis in Greece

P Makras ∙ GP Lyritis ∙ S Rizou ∙ T Drakopoulou ∙ G Trovas ∙ C Willers ∙ N Norton ∙ NC Harvey ∙ T Jacobson ∙ H Johansson ∙ M Lorentzon ∙ EV McCloskey ∙ F Borgström ∙ JA Kanis

**Introduction**

The scorecard summarises key indicators of the burden of osteoporosis and its management in the 27 member states of the European Union, as well as the UK and Switzerland (termed EU27+2) [1]. This country-specific report summarises the principal results for Greece.

**Methods**

The information obtained covers four domains: burden of osteoporosis and fractures; policy framework; service provision; and service uptake. Data were collected from numerous sources including previous research and IOF reports, and available registers which were used for additional analysis of resource utilization, costing and HRQoL data. Furthermore, country-specific information on osteoporosis management was obtained from each IOF member state via a questionnaire.

**Burden of disease**

The direct cost of incident fractures in Greece in 2019 was €694.7 million. Added to this was the ongoing cost in 2019 from fractures that occurred before 2019, which amounted to €203.5 million (long-term disability). The cost of pharmacological intervention (assessment and treatment) was €80.5 million. Thus, the total direct cost (excluding the value of QALYs lost) amounted to €0.98 billion in 2019. Key metrics are presented in Table 1.

In 2019, the average direct cost of osteoporotic fractures in Greece was €91.2 per individual in the population, while in 2010 the average was €66.2 (after adjusting for inflation), representing an increase of 38% (€91.2 versus €66.2) and put Greece in 13th place in terms of highest cost of osteoporotic fractures per capita in the EU27+2.

The cost of osteoporotic fractures in Greece accounted for approximately 6.2% of healthcare spending (i.e. €0.98 billion out of €14.60 billion in 2019), which was significantly higher than the EU27+2 average of 3.5%. Indeed, Greece was ranked first across the EU27+2 countries. These numbers indicate a substantial impact of fragility fractures on the healthcare budget.

Using World Health Organization diagnostic criteria for osteoporosis based on the measurement of bone mineral density (BMD) [2], there were approximately 684,000 individuals with osteoporosis in Greece in 2019, of whom almost 80% were women. The prevalence of osteoporosis in the total Greek population amounted to 5.5%, on par with the EU27+2 average (5.6%).

**Table 1** Key measures of burden of disease for Greece

**Table:** 

**Category**	**Measure**	**Estimate**	**Rank**
Burden of disease	Direct cost of incident fracture (€m)	694.70	
	Long-term disability cost (€m)	203.51	
	Intervention cost (€m)	80.46	
	Total cost (€m)	978.68	
	QALYs lost (€m)	1 518	
	Cost per capita (€)	91.23	13
	Proportion of healthcare spending	6.2%	1
	Prevalence of osteoporosis	5.7%	7

There were estimated to be 99,000 new fragility fractures in Greece in 2019, equivalent to 272 fractures/day (or 11 per hour). This was a slight increase compared to 2010, equivalent to an increment of 1.8 fractures/1000 individuals, totalling 22.0 fractures/ 1000 individuals in 2019.

Some osteoporotic fractures are associated with premature mortality [3]. In Greece, the annual number of deaths associated with a fracture event was estimated to be 130 per 100,000 individuals of the population aged 50 years or more, compared to the EU27+2 average of 116/100,000. The number of fracture-related deaths is comparable to or exceeds that for some of the most common causes of death such as lung cancer, diabetes, chronic lower respiratory diseases.

The remaining lifetime probability of hip fracture (%) at the ages of 50 years in men and women was 8.0% and 15.8%, respectively, placing Greece in the upper tertile of risk for men and the mid tertile for women.

The population in men and women age 50 years or more is projected to increase by 11.9% between 2019 and 2034, close to the EU27+2 average of 11.4%. The increases in men and women aged 75 years or more are even more marked and amount to 23.7% and 21.0%, respectively. The annual number of osteoporotic fractures in Greece is expected to increase by 22,000 to 121,000 in 2034.

**Policy framework** (Table 2)

Documentation of the burden of disease is an essential prerequisite to determine the resources that should be allocated to the diagnosis and treatment of the disorder. High quality national data on hip fracture rates have been identified in 18 of 29 countries, of which Greece was not deemed as one. No data are collected on a national basis and the latest report dates from 2007 [4].

Given that osteoporosis and fragility fractures are common and that effective treatments are widely available, the vast majority of patients with osteoporosis are preferably managed at the primary health care level by general practitioners (GPs), with specialist referral reserved for difficult complex cases. Primary care was the principal provider of the medical care for osteoporosis in 13 of the 28 countries where data were available.

Osteoporosis and metabolic bone disease is not a recognised specialty in most countries including Greece. For Greece, orthopaedics was the lead specialty for osteoporosis management. Specialty care of osteoporosis in Greece is also managed via other specialties including endocrinology, and rheumatology. Osteoporosis is also recognized as a component of specialty training. Although it is possible that these specialties educate their trainees adequately, the wide variation may reflect inconsistencies in patient care, training of primary care physicians and a suboptimal voice to “defend” the interests of those who work within the field of osteoporosis.

**Table 2** Policy framework for osteoporosis in Greece

**Table:** 

**Category**	**Measure**	**Estimate**
Policy framework	National fracture data availability	No
	OP recognized as a specialty	No
	OP primarily managed in primary care	No
	Other specialties involved	Orthopaedics, Endocrinology, Rheumatology
	Advocacy areas covered by patient organisation	Policy, capacity, research and development

The role of national patient organisations is to improve the care of patients and increase awareness and prevention of osteoporosis and related fractures among the general public. Advocacy by patient organisations can fall into four categories: policy, capacity building and education, peer support, research and development. For Greece, three of these advocacy areas were covered by a patient organisation. All four advocacy areas were covered for only 10 out of the 26 countries with at least one patient organisation.

**Service provision** (Table 3)

A wide variety of approved drug treatments is available for the management of osteoporosis [5]. Potential limitations of their use in member states relate to reimbursement policies which may impair the delivery of health care. 12 out of 27 countries offered full reimbursement, of which Greece was not one.

The assessment of bone mineral density forms a key component for the general management of osteoporosis, being used for diagnosis, risk prediction, selection of patients for treatment and monitoring of patients on treatment. In Greece, the number of DXA units expressed per million of the general population amounted to 51.4 which puts the country in 1^st^ place among the EU27+2.

The average waiting time for DXA ranged from 0 to 180 days across countries, and there was no clear relation between waiting times and the availability of DXA. In Greece, the estimated average waiting time for DXA amounted to five days. Only two countries reported shorter average waiting times.

**Table 3** Service provision for osteoporosis in Greece

**Table:** 

**Category**	**Measure**	**Estimate**	**Rank**
Service provision	Reimbursement of OP medications	75%	
	DXA units/million inhabitants	51.4	1
	DXA cost (€)	55	11
	FRAX risk assessment model available	Yes	
	Fracture liaison service density	1-10%	

Reimbursement for DXA scans varied between member states both in terms of the criteria required and level of reimbursement awarded. In Greece, the reimbursement was conditional and varied depending on the patient’s condition.

The effective targeting of treatment to those at highest risk of fracture requires an assessment of fracture risk. Risk assessment models for fractures, most usually based on FRAX, were available in 24 out of 29 countries, of which Greece was one. For Greece, guidance on the use of risk assessment within national guidelines was available, as in only 14 of the other countries.

Guidelines for the management of osteoporosis were available in Greece (as in 27 out of 29 countries). The guidelines in Greece included postmenopausal women specifically, as well as osteoporosis in men.

Fracture liaison services (FLS), also known as osteoporosis coordinator programmes and care manager programmes, provide a system for the routine assessment and management of postmenopausal women and older men who have sustained a low trauma fracture. Fracture liaison services were reported for 1–10% of hospitals in Greece.

The use of indicators to systematically measure the quality of care provided to people with osteoporosis or associated fractures has expanded as a discipline within the past decade [6]. No use of national quality indicators was reported for Greece.

**Service uptake** (Table 4)

The web-based usage of FRAX showed considerable heterogeneity in uptake between the countries. The average uptake for the EU27+2 was 1,555 sessions/million/year of the general population with an enormous range of 49 to 41,874 sessions/million. The usage for Greece amounted to 4,566 sessions/million in 2019, with an eight-fold increase since 2011.

Many studies have demonstrated that a significant proportion of men and women at high fracture risk do not receive therapy for osteoporosis (the treatment gap) [7]. In the EU27+2 the average treatment gap was 71% but ranged from 32% to 87%. For Greece, the treatment gap amongst women amounted to 43% or 211,000 out of 485,000 characterised at risk and had increased compared to 2010. The average treatment gap among EU27+2 increased from 55% in 2010 to 71% in 2019.

**Table 4** Service uptake for osteoporosis in Greece

**Table:** 

**Category**	**Measure**	**Estimate**	**Rank**
Service uptake	Number of FRAX sessions/million people/year	4566	4
	Treatment gap for women eligible for treatment (%)	43	3
	Proportion surgically managed hip fractures	>90%	

About 5% of people with a hip fracture die within 1 month of their fracture [8]. A determinant of peri-operative morbidity and mortality is the time a patient takes to get to surgery [9]. For Greece, the average waiting time for hip fracture surgery after hospital admission was reported to be 2–3 days. The proportion of surgically managed hip fractures was reported to be over 90%.

**Scores and scorecard**

Scores were developed for Burden of disease and the healthcare provision (Policy framework, Service provision and Service uptake) in the EU27+2 countries. Greece scores resulted in a 9th place regarding Burden of disease. The combined healthcare provision scorecard resulted in a 18th place for Greece. Thus, Greece presents as one of the eight high-burden low-provision countries among the EU27+2.

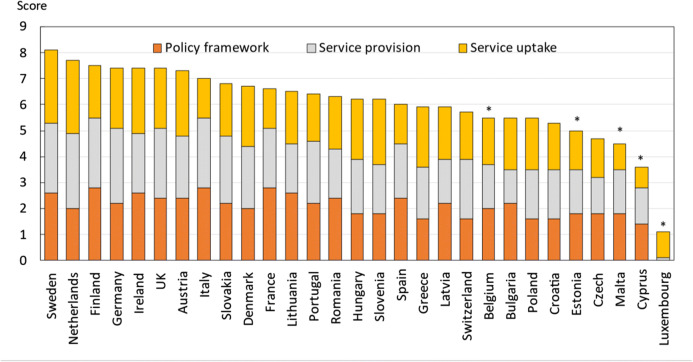



**Fig. 1** Scores by country for metrics related to policy framework, service provision and service uptake. The mean score for each of the 3 domains is given. An asterisk denotes that there was one or more missing metric which decreases the overall score

The first SCOPE was undertaken in 2010, almost 10 years previously. Fifteen of the 16 score card metrics on healthcare provision were used in the two surveys. Scores had improved or markedly improved in 15 countries, remained constant in 8 countries and worsened in 3 countries. For Greece the scores were unchanged.

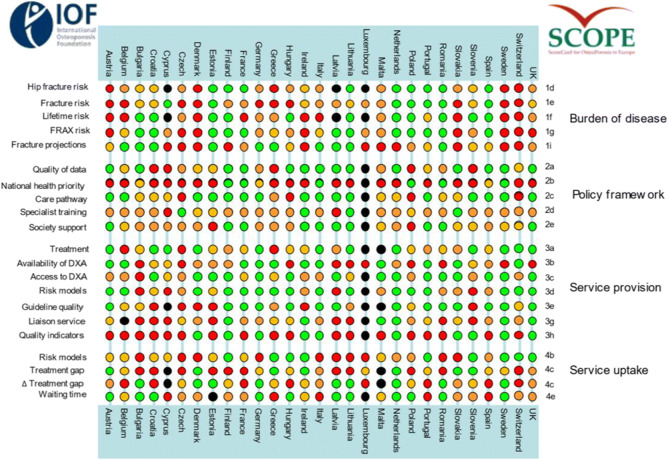



**Fig. 2** The scorecard for all the EU27+2 countries illustrating the scores across the four domains. The elements of each domain in each country were scored and coded using a traffic light system (red, orange, green). Black dots signify missing information

The second edition of the Scorecard for Osteoporosis in Europe (SCOPE 2021) allows health and policy professionals to assess key indicators on the healthcare provision for osteoporosis within countries and between countries within the EU 27+2. The scorecard is not intended as a prescriptive template. Thus, it does not set performance targets but may serve as a guide to the performance targets at which to aim in order to deliver the outcomes required.

**Acknowledgements**

SCOPE was supported by an unrestricted grant from Amgen to the International Osteoporosis Foundation (IOF). Amgen was neither involved in the design nor writing of the report. We are grateful to Anastasia Soulié Mlotek and Dominique Pierroz of the IOF for their help in the administration of SCOPE. We are grateful to the Butterfly Bone Health Society for their assistance. The report has been reviewed by the members of the SCOPE Consultation Panel and the relevant IOF National societies, and we are grateful for their local insights on the management of osteoporosis in each country. The source document has been reviewed and endorsed by the Committee of Scientific Advisors of the IOF and benefitted from their feedback.

**References**

1. Kanis JA, Norton N, Harvey NC, Jacobson T, Johansson H, Lorentzon M, McCloskey EV, Willers C, Borgström F (2021) SCOPE 2021: a new scorecard for osteoporosis in Europe. Arch Osteoporos 16:82. 10.1007/s11657-020-00871-9

2. World Health Organisation (1994) Assessment of fracture risk and its application to screening for postmenopausal osteoporosis. Report of a WHO Study Group. World Health Organ Tech Rep Ser, 1994/01/01 edn, pp 1-129

3. Johnell O, Kanis JA, Oden A, Sernbo I, Redlund-Johnell I, Petterson C, De Laet C, Jonsson B (2004) Mortality after osteoporotic fractures. Osteoporos Int 15:38-42

4. Lyritis GP, Rizou S, Galanos A, Makras P (2013) Incidence of hip fractures in Greece during a 30-year period: 1977-2007. Osteoporos Int 24: 1579-85.

5. Hernlund E, Svedbom A, Ivergard M, Compston J, Cooper C, Stenmark J, McCloskey EV, Jonsson B, Kanis JA (2013) Osteoporosis in the European Union: medical management, epidemiology and economic burden. A report prepared in collaboration with the International Osteoporosis Foundation (IOF) and the European Federation of Pharmaceutical Industry Associations (EFPIA). Arch Osteoporos 8:136

6. Allen P, Pilar M, Walsh-Bailey C, Hooley C, Mazzucca S, Lewis CC, Mettert KD, Dorsey CN, Purtle J, Kepper MM, Baumann AA, Brownson RC (2020) Quantitative measures of health policy implementation determinants and outcomes: a systematic review. Implement Sci 15:47

7. Borgstrom F, Karlsson L, Ortsater G, Norton N, Halbout P, Cooper C, Lorentzon M, McCloskey EV, Harvey NC, Javaid MK, Kanis JA (2020) Fragility fractures in Europe: burden, management and opportunities. Arch Osteoporos 15:59

8. Kanis JA, Oden A, Johnell O, De Laet C, Jonsson B, Oglesby AK (2003) The components of excess mortality after hip fracture. Bone 32:468-473

9. National Clinical Guideline Centre (2011) The Management of Hip Fracture in Adults. In Centre NCG (ed) London

## **Epidemiology and economic burden of osteoporosis in Hungary**

I Takacs ∙ J Donáth ∙ L Szekeres ∙ C Willers ∙ N Norton ∙ NC Harvey ∙ T Jacobson ∙ H Johansson ∙ M Lorentzon ∙ EV McCloskey ∙ F Borgström ∙ JA Kanis

**Introduction**

The scorecard summarises key indicators of the burden of osteoporosis and its management in the 27 member states of the European Union, as well as the UK and Switzerland (termed EU27+2) [1]. This country-specific report summarises the principal results for Hungary.

**Methods**

The information obtained covers four domains: burden of osteoporosis and fractures; policy framework; service provision; and service uptake. Data were collected from numerous sources including previous research and IOF reports, and available registers which were used for additional analysis of resource utilization, costing and HRQoL data. Furthermore, country-specific information on osteoporosis management was obtained from each IOF member state via a questionnaire.

**Burden of disease**

The direct cost of incident fractures in Hungary in 2019 was €348.9 million. Added to this was the ongoing cost in 2019 from fractures that occurred before 2019, which amounted to €79.7 million (long-term disability). The cost of pharmacological intervention (assessment and treatment) was €20.9 million. Thus, the total direct cost (excluding the value of QALYs lost) amounted to €449.4 million in 2019. Key metrics are presented in Table 1.

In 2019, the average direct cost of osteoporotic fractures in Hungary was €46.0 per individual in the population, while in 2010 the average was €22.1 (after adjusting for inflation), representing an increase of 108% (€46.0 versus €22.1). The 2019 data rank Hungary in 20th place in terms of highest cost of osteoporotic fractures per capita in the EU27+2.

The cost of osteoporotic fractures in Hungary accounted for approximately 5.0% of healthcare spending (i.e. €449.4 million out of €8.6 billion in 2019), which was higher than the EU27+2 average of 3.5% ranking 5th cross the EU27+2 countries. These data indicate a substantial impact of fragility fractures on the healthcare budget.

Using World Health Organization diagnostic criteria for osteoporosis based on the measurement of bone mineral density (BMD) [2], there were approximately 559,000 individuals with osteoporosis in Hungary in 2019, of whom approximately 82% were women. The prevalence of osteoporosis in the total Hungarian population amounted to 5.5%, on par with the EU27+2 average (5.6%).

**Table 1** Key measures of burden of disease for Hungary

**Table:** 

**Category**	**Measure**	**Estimate**	**Rank**
Burden of disease	Direct cost of incident fracture (€m)	348.93	
	Long-term disability cost (€m)	79.65	
	Intervention cost (€m)	20.85	
	Total cost (€m)	449.44	
	QALYs lost (€m)	890	
	Cost per capita (€)	46.01	20
	Proportion of healthcare spending	5.0%	5
	Prevalence of osteoporosis	5.5%	15

There were estimated to be 86,000 new fragility fractures in Hungary in 2019, equivalent to 236 fractures/day (or 10 per hour). This was a decrease compared to 2010, equivalent to a decrement 5.0 fractures per 1000 individuals, totalling 22.8 fractures/ 1000 individuals in 2019.

Some osteoporotic fractures are associated with premature mortality [3]. In Hungary, the annual number of deaths associated with a fracture event was estimated to be 209 per 100,000 individuals of the population aged 50 years or more, compared to the EU27+2 average of 116/100,000. The number of fracture-related deaths is comparable to, or exceeds, that for some of the most common causes of death such as lung cancer, diabetes, chronic lower respiratory diseases.

The remaining lifetime probability of hip fracture (%) at the ages of 50 years in men and women was 4.1% and 10.6%, respectively, placing Hungary in the lower tertile of risk for both men and women.

The population of men and women age 50 years or more is projected to increase by 9.8% in men and women between 2019 and 2034, close to the EU27+2 average of 11.4%. The increases in men and women aged 75 years or more are even more marked and amount to 49.3% and 32.1%, respectively. The annual number of osteoporotic fractures in Hungary is expected to increase by 22,000 to 108,000 in 2034.

**Policy framework** (Table 2)

Documentation of the burden of disease is an essential prerequisite to determine the resources that should be allocated to the diagnosis and treatment of the disorder. High quality national data on hip fracture rates have been identified in 18 of 29 countries, of which Hungary is one. Data are collected on a national basis and include more than only hip fracture data.

Given that osteoporosis and fragility fractures are common and that effective treatments are widely available, the vast majority of patients with osteoporosis are preferably managed at the primary health care level by general practitioners (GPs), with specialist referral reserved for difficult complex cases. Primary care was the principal provider of the medical care for osteoporosis in 13 of the 28 countries where data were available. In Hungary, osteoporosis care was managed within the rheumatology and endocrinology specialties. Osteoporosis and metabolic bone disease is not a recognised specialty in most countries including Hungary. Osteoporosis is however recognized as a component of specialty training. Although it is possible that these specialties educate their trainees adequately, the wide variation may reflect inconsistencies in patient care, training of primary care physicians and a suboptimal voice to “defend” the interests of those who work within the field of osteoporosis.

**Table 2** Policy framework for osteoporosis in Austria

**Table:** 

**Category**	**Measure**	**Estimate**
Policy framework	National fracture data availability	Yes
	OP recognized as a specialty	No
	OP primarily managed in primary care	No
	Other specialties involved	Rheumatology, Endocrinology
	Advocacy areas covered by patient organisation	None

The role of national patient organisations is to improve the care of patients and increase awareness and prevention of osteoporosis and related fractures among the general public. Advocacy by patient organisations can fall into four categories: policy, capacity building and education, peer support, research and development. For Hungary, none of the advocacy areas were covered by a patient organisation. All four of the advocacy areas were covered for only 10 out of the 26 countries with at least one patient organisation.

**Service provision** (Table 3)

A wide variety of approved drug treatments is available for the management of osteoporosis [4]. Potential limitations of their use in member states relate to reimbursement policies which may impair the delivery of health care. Twelve out of 27 countries offered full reimbursement, and Hungary was not one of these.

The assessment of bone mineral density forms a key component for the general management of osteoporosis, being used for diagnosis, risk prediction, selection of patients for treatment and monitoring of patients on treatment. In Hungary, the number of DXA units expressed per million of the general population amounted to 6.9 which puts the country in 26th place among the EU27+2.

The average waiting time for DXA ranged from 0 to 180 days across countries, and there was no clear relation between waiting times and the availability of DXA. In Hungary, the estimated average waiting time for DXA amounted to 14 days. Nine countries reported shorter average waiting times.

**Table 3** Service provision for osteoporosis in Hungary

**Table:** 

**Category**	**Measure**	**Estimate**	**Rank**
Service provision	Reimbursement of OP medications	70-90%	
	DXA units/million inhabitants	6.9	26
	DXA cost (€)	20	25
	FRAX risk assessment model available	Yes	
	Fracture liaison service density	1-10%	

Reimbursement for DXA scans varied between member states both in terms of the criteria required and level of reimbursement awarded. In Hungary, the reimbursement was unconditional.

The effective targeting of treatment to those at highest risk of fracture requires an assessment of fracture risk. Risk assessment models for fractures, most usually based on FRAX, were available in 24 out of 29 countries, of which Hungary was one. For Hungary, guidance on the use of risk assessment within national guidelines was available, as in only 14 of the other countries.

Guidelines for the management of osteoporosis were available in Hungary (as in a total of 27 out of 29 countries). The guidelines in Hungary included postmenopausal women specifically, as well as osteoporosis in men and secondary osteoporosis including glucocorticoid-induced osteoporosis.

Fracture liaison services (FLS), also known as osteoporosis coordinator programmes and care manager programmes, provide a system for the routine assessment and management of postmenopausal women and older men who have sustained a low trauma fracture. Fracture liaison services were reported for 1–10% of hospitals in Hungary.

The use of indicators to systematically measure the quality of care provided to people with osteoporosis or associated fractures has expanded as a discipline within the past decade [5]. No use of national quality indicators was reported for Hungary.

**Service uptake** (Table 4)

The web-based usage of FRAX showed considerable heterogeneity in uptake between the countries. The average uptake for the EU27+2 was 1,555 sessions/million/year of the general population with an enormous range of 49 to 41,874 sessions/million. The use of FRAX in Hungary amounted to 2,832 sessions/million in 2019, with a 135 percent increase since 2011.

Many studies have demonstrated that a significant proportion of men and women at high fracture risk do not receive therapy for osteoporosis (the treatment gap) [6]. In the EU27+2 the average treatment gap was 71% but ranged from 32 to 87%. For Hungary, the treatment gap amongst women amounted to 65% or 236,000 out of 361,000 characterised at risk and it grew significantly compared to 2010. The average treatment gap among EU27+2 increased from 55% in 2010 to 71% in 2019.

**Table 4** Service uptake for osteoporosis in Hungary

**Table:** 

**Category**	**Measure**	**Estimate**	**Rank**
Service uptake	Number of FRAX sessions/million people/year	2832	7
	Treatment gap for women eligible for treatment (%)	65	9
	Proportion surgically managed hip fractures	75-90%	

About 5% of people with a hip fracture die within 1 month of their fracture [7]. A determinant of peri-operative morbidity and mortality is the time a patient takes to get to surgery [8]. For Hungary, the average waiting time for hip fracture surgery after hospital admission was reported to be less than 24 h. The proportion of surgically managed hip fractures was reported to be between 75 and 90%.

**Scores and scorecard**

Scores were developed for Burden of disease and the healthcare provision (Policy framework, Service provision and Service uptake) in the EU27+2 countries. Hungary scores resulted in a 13th place regarding Burden of disease, and the combined healthcare provision scorecard resulted in a 15th place for Hungary.

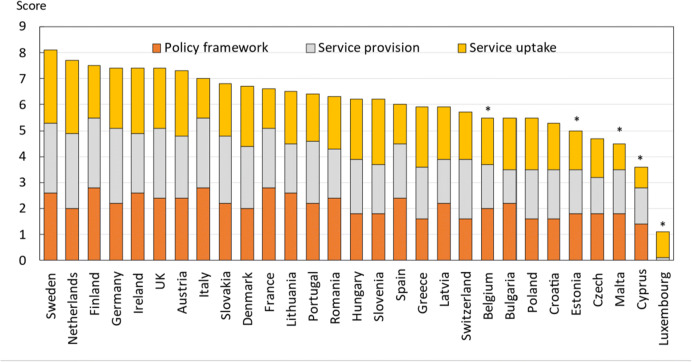



**Fig. 1** Scores by country for metrics related to policy framework, service provision and service uptake. The mean score for each of the 3 domains is given. An asterisk denotes that there was one or more missing metric which decreases the overall score

The first SCOPE was undertaken in 2010, almost 10 years previously. Fifteen of the 16 score card metrics on healthcare provision were used in the two surveys. Scores had improved or markedly improved in 15 countries, remained constant in 8 countries and worsened in 3 countries. For Hungary the scores were almost unchanged.

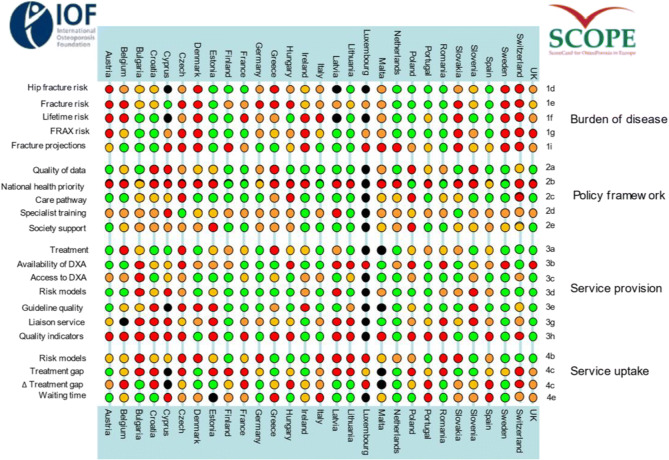



**Fig. 2** The scorecard for all the EU27+2 countries illustrating the scores across the four domains. The elements of each domain in each country were scored and coded using a traffic light system (red, orange, green). Black dots signify missing information

The second edition of the Scorecard for Osteoporosis in Europe (SCOPE 2021) allows health and policy professionals to assess key indicators on the healthcare provision for osteoporosis within countries and between countries within the EU 27+2. The scorecard is not intended as a prescriptive template. Thus, it does not set performance targets but may serve as a guide to the performance targets at which to aim in order to deliver the outcomes required.

**Acknowledgements**

SCOPE was supported by an unrestricted grant from Amgen to the International Osteoporosis Foundation (IOF). Amgen was neither involved in the design nor writing of the report. We are grateful to Anastasia Soulié Mlotek and Dominique Pierroz of the IOF for their help in the administration of SCOPE. The report has been reviewed by the members of the SCOPE Consultation Panel and the relevant IOF National societies, and we are grateful for their local insights on the management of osteoporosis in each country. The source document has been reviewed and endorsed by the Committee of Scientific Advisors of the IOF and benefitted from their feedback.

**References**

1. Kanis JA, Norton N, Harvey NC, Jacobson T, Johansson H, Lorentzon M, McCloskey EV, Willers C, Borgström F (2021) SCOPE 2021: a new scorecard for osteoporosis in Europe. Arch Osteoporos 16:82. 10.1007/s11657-020-00871-9

2. World Health Organisation (1994) Assessment of fracture risk and its application to screening for postmenopausal osteoporosis. Report of a WHO Study Group. World Health Organ Tech Rep Ser, 1994/01/01 edn, pp 1-129

3. Johnell O, Kanis JA, Oden A, Sernbo I, Redlund-Johnell I, Petterson C, De Laet C, Jonsson B (2004) Mortality after osteoporotic fractures. Osteoporos Int 15:38-42

4. Hernlund E, Svedbom A, Ivergard M, Compston J, Cooper C, Stenmark J, McCloskey EV, Jonsson B, Kanis JA (2013) Osteoporosis in the European Union: medical management, epidemiology and economic burden. A report prepared in collaboration with the International Osteoporosis Foundation (IOF) and the European Federation of Pharmaceutical Industry Associations (EFPIA). Arch Osteoporos 8:136

5. Allen P, Pilar M, Walsh-Bailey C, Hooley C, Mazzucca S, Lewis CC, Mettert KD, Dorsey CN, Purtle J, Kepper MM, Baumann AA, Brownson RC (2020) Quantitative measures of health policy implementation determinants and outcomes: a systematic review. Implement Sci 15:47

6. Borgstrom F, Karlsson L, Ortsater G, Norton N, Halbout P, Cooper C, Lorentzon M, McCloskey EV, Harvey NC, Javaid MK, Kanis JA (2020) Fragility fractures in Europe: burden, management and opportunities. Arch Osteoporos 15:59

7. Kanis JA, Oden A, Johnell O, De Laet C, Jonsson B, Oglesby AK (2003) The components of excess mortality after hip fracture. Bone 32:468-473

8. National Clinical Guideline Centre (2011) The Management of Hip Fracture in Adults. In Centre NCG (ed)London

## **Epidemiology and economic burden of osteoporosis in Ireland**

Moira O’Brien ∙ Michele O’Brien ∙ C Willers ∙ N Norton ∙ NC Harvey ∙ T Jacobson ∙ H Johansson ∙ M Lorentzon ∙ EV McCloskey ∙ F Borgström ∙ JA Kanis

**Introduction**

The scorecard summarises key indicators of the burden of osteoporosis and its management in the 27 member states of the European Union, as well as the UK and Switzerland (termed EU27+2) [1]. This country-specific report summarises the principal results for Ireland.

**Methods**

The information obtained covers four domains: burden of osteoporosis and fractures; policy framework; service provision; and service uptake. Data were collected from numerous sources including previous research and IOF reports, and available registers which were used for additional analysis of resource utilization, costing and HRQoL data. Furthermore, country-specific information on osteoporosis management was obtained from each IOF member state via a questionnaire.

**Burden of disease**

The direct cost of incident fractures in Ireland in 2019 was €290.8 million. Added to this was the ongoing cost in 2019 from fractures that occurred before 2019, which amounted to €135.7 million (long-term disability). The cost of pharmacological intervention (assessment and treatment) was €37.7 million. Thus, the total direct cost (excluding the value of QALYs lost) amounted to €464.3 million in 2019. Key metrics are presented in Table 1.

In 2019, the average direct cost of osteoporotic fractures in Ireland was €95.7 per individual in the population, while in 2010 the average was €55.2 (after adjusting for inflation) representing an increase of 73% (€95.7 versus €55.2). The 2019 data rank Ireland in 11th place in terms of highest cost of osteoporotic fractures per capita in the EU27+2.

The cost of osteoporotic fractures in Ireland accounted for approximately 2.0% of healthcare spending (i.e. €464 million out of €21.3 billion in 2019), much lower than the EU27+2 average of 3.5%. These data suggest an underinvestment of the healthcare budget in osteoporosis.

Using World Health Organization diagnostic criteria for osteoporosis based on the measurement of bone mineral density (BMD) [2], there were approximately 209,000 individuals with osteoporosis in Ireland in 2019, of whom almost 80% were women. The prevalence of osteoporosis in the total Irish population amounted to 3.7%, somewhat lower than the EU27+2 average (5.6%).

**Table 1** Key measures of burden of disease for Ireland

**Table:** 

**Category**	**Measure**	**Estimate**	**Rank**
Burden of disease	Direct cost of incident fracture (€m)	290.84	
	Long-term disability cost (€m)	135.72	
	Intervention cost (€m)	37.73	
	Total cost (€m)	464.29	
	QALYs lost (€m)	1456	
	Cost per capita (€)	95.66	11
	Proportion of healthcare spending	2.0%	27
	Prevalence of osteoporosis	3.7%	29

There were estimated to be 32,000 new fragility fractures in Ireland in 2019, equivalent to 89 fractures/day (or 3.7 per hour). This was an increase compared to 2010, equivalent to an increment of 6.1 fractures/1000 individuals, totalling 20.6 fractures/ 1000 individuals in 2019.

Some osteoporotic fractures are associated with premature mortality [3]. In Ireland, the annual number of deaths associated with a fracture event was estimated to be 115 per 100,000 individuals of the population aged 50 years or more, compared to the EU27+2 average of 116/100,000. The number of fracture-related deaths is comparable to, or exceeds, that for some of the most common causes of death such as lung cancer, diabetes, chronic lower respiratory diseases.

The remaining lifetime probability of hip fracture (%) at the ages of 50 years in men and women was 7.8% and 18.2%, respectively, placing Ireland in the upper tertile of risk for both men and women.

The population in men and women age 50 years or more is projected to increase by 38.0% between 2019 and 2034, significantly higher than the EU27+2 average of 11.4%. The increases in men and women aged 75 years or more are even more marked and amount to 78.9% and 69.0%, respectively. The annual number of osteoporotic fractures in Ireland is expected to increase by 19,000 to 51,000 in 2034.

**Policy framework** (Table 2)

Documentation of the burden of disease is an essential prerequisite to determine the resources that should be allocated to the diagnosis and treatment of the disorder. High quality national data on hip fracture rates have been identified in 18 of 29 countries, of which Ireland is one. Data are collected on a national basis and include hip fracture data.

Given that osteoporosis and fragility fractures are common and that effective treatments are widely available, the vast majority of patients with osteoporosis are preferably managed at the primary health care level by general practitioners (GPs), with specialist referral reserved for difficult complex cases. Primary care was the principal provider of the medical care for osteoporosis in Ireland, as for 13 of the 28 countries where data were available.

Osteoporosis and metabolic bone disease is not a recognised specialty in most countries, but this is the case in Ireland. Specialty care of osteoporosis in Ireland is however also managed via other specialties including geriatrics, rheumatology, orthopaedics and primary care. Osteoporosis is also recognized as a component of specialty training. Although it is possible that these other specialties educate their trainees adequately, the wide variation may reflect inconsistencies in patient care, training of primary care physicians and a suboptimal voice to “defend” the interests of those who work within the field of osteoporosis.

**Table 2** Policy framework for osteoporosis in Ireland

**Table:** 

**Category**	**Measure**	**Estimate**
Policy framework	National fracture data availability	Yes
	OP recognized as a specialty	Yes
	OP primarily managed in primary care	Yes
	Other specialties involved	Geriatrics, Rheumatology, Orthopaedics
	Advocacy areas covered by the national osteoporosis organisation	Policy, capacity, peer support, research and development

The role of national osteoporosis organisations is to improve the care of patients and increase awareness and prevention of osteoporosis and related fractures among the general public and health professionals. Advocacy by national organisations can fall into four categories: policy, capacity building and education, peer support, research and development. For Ireland, all four of the advocacy areas were covered by the national osteoporosis organisation, which was the case for only 10 out of the 26 countries with at least one patient organisation.

**Service provision** (Table 3)

A wide variety of approved drug treatments is available for the management of osteoporosis [4]. Potential limitations of their use in member states relate to reimbursement policies which may impair the delivery of health care. Ireland was not one of the 12 (out of 27) countries that offered full reimbursement.

The assessment of bone mineral density forms a key component for the general management of osteoporosis, being used for diagnosis, risk prediction, selection of patients for treatment and monitoring of patients on treatment. In Ireland, the number of DXA units expressed per million of the general population amounted to 20.5 which puts the country in 11th place among the EU27+2.

The average waiting time for DXA ranged from 0 to 180 days across countries, and there was no clear relation between waiting times and the availability of DXA. In Ireland, the estimated average waiting time for DXA amounted to seven days. Four countries reported shorter average waiting times.

**Table 3** Service provision for osteoporosis in Ireland

**Table:** 

**Category**	**Measure**	**Estimate**	**Rank**
Service provision	DXA units/million inhabitants	20.5	11
	DXA cost (€)	120	2
	FRAX risk assessment model available	Yes	
	Fracture liaison service density	25-50%	

Reimbursement for DXA scans varied between member states both in terms of the criteria required and level of reimbursement awarded. In Ireland, the reimbursement was conditional and varied depending on listing with caregiver.

The effective targeting of treatment to those at highest risk of fracture requires an assessment of fracture risk. Risk assessment models for fractures, most usually based on FRAX, were available in 24 out of 29 countries, of which Ireland was one. For Ireland, guidance on the use of risk assessment within national guidelines was available, as in only 14 of the other countries.

Guidelines for the management of osteoporosis were available in Ireland (as in a total of 27 out of 29 countries). The guidelines in Ireland included postmenopausal women specifically, as well as osteoporosis in men and secondary osteoporosis including glucocorticoid-induced osteoporosis.

Fracture liaison services (FLS), also known as osteoporosis coordinator programmes and care manager programmes, provide a system for the routine assessment and management of postmenopausal women and older men who have sustained a low trauma fracture. Fracture liaison services were reported for 25–50% of hospitals in Ireland.

The use of indicators to systematically measure the quality of care provided to people with osteoporosis or associated fractures has expanded as a discipline within the past decade [5]. Ireland was one of few countries with national quality indicators in place.

**Service uptake** (Table 4)

The web-based usage of FRAX showed considerable heterogeneity in uptake between the countries. The average uptake for the EU27+2 was 1,555 sessions/million/year of the general population with an enormous range of 49 to 41,874 sessions/million. The use of FRAX in Ireland amounted to 2,623 sessions/million in 2019, with a 60 percent increase since 2011.

Many studies have demonstrated that a significant proportion of men and women at high fracture risk do not receive therapy for osteoporosis (the treatment gap) [6]. In the EU27+2 the average treatment gap was 71% but ranged from 32 to 87%. For Ireland, the treatment gap amongst women amounted to 32% or 49,000 out of 153,000 characterised at risk. The Irish treatment gap did not change significantly compared to 2010, whilst the average treatment gap among EU27+2 increased from 55% in 2010 to 71% in 2019.

**Table 4** Service uptake for osteoporosis in Ireland

**Table:** 

**Category**	**Measure**	**Estimate**	**Rank**
Service uptake	Number of FRAX sessions/million people/year	2623	9
	Treatment gap for women eligible for treatment (%)	32	1
	Proportion surgically managed hip fractures	>90%	

About 5% of people with a hip fracture die within 1 month of their fracture [7]. A determinant of peri-operative morbidity and mortality is the time a patient takes to get to surgery [8]. For Ireland, the average waiting time for hip fracture surgery after hospital admission was reported to be 1–2 days, implying a reduction in waiting time compared to 2010 (waiting time of 2–3 days). The proportion of surgically managed hip fractures was reported to be over 90%.

**Scores and scorecard**

Scores were developed for Burden of disease and the healthcare provision (Policy framework, Service provision and Service uptake) in the EU27+2 countries. Ireland scores resulted in a 7th place regarding Burden of disease. The combined healthcare provision scorecard resulted in a 5th place for Ireland. Thus, Ireland presents as one of the high-burden high-provision countries among the EU27+2.

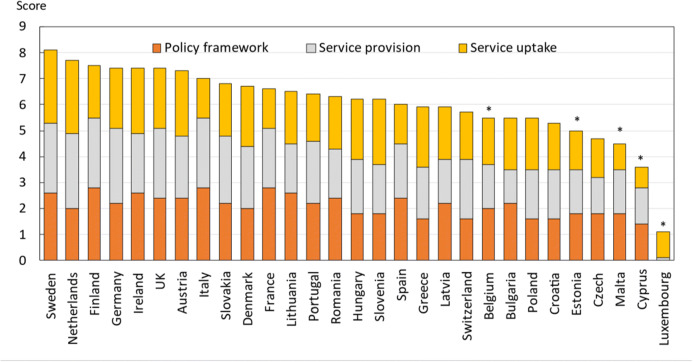



**Fig. 1** Scores by country for metrics related to policy framework, service provision and service uptake. The mean score for each of the 3 domains is given. An asterisk denotes that there was one or more missing metric which decreases the overall score

The first SCOPE was undertaken in 2010, almost 10 years previously. Fifteen of the 16 score card metrics on healthcare provision were used in the two surveys. Scores had improved or markedly improved in 15 countries, remained constant in 8 countries and worsened in 3 countries. For Ireland, the scores were markedly improved.

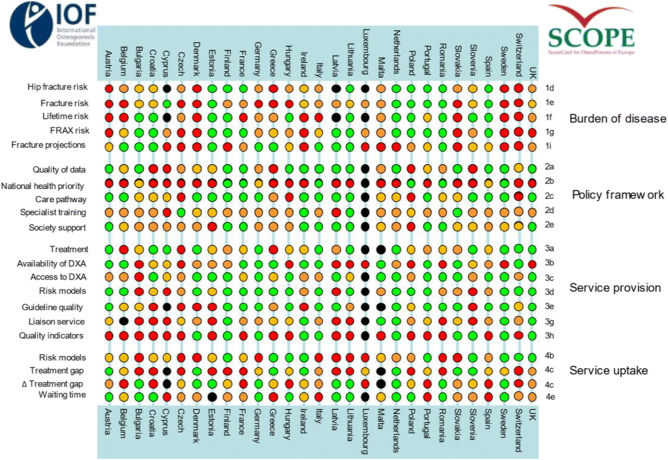



**Fig. 2** The scorecard for all the EU27+2 countries illustrating the scores across the four domains. The elements of each domain in each country were scored and coded using a traffic light system (red, orange, green). Black dots signify missing information

The second edition of the Scorecard for Osteoporosis in Europe (SCOPE 2021) allows health and policy professionals to assess key indicators on the healthcare provision for osteoporosis within countries and between countries within the EU 27+2. The scorecard is not intended as a prescriptive template. Thus, it does not set performance targets but may serve as a guide to the performance targets at which to aim in order to deliver the outcomes required.

**Acknowledgements**

SCOPE was supported by an unrestricted grant from Amgen to the International Osteoporosis Foundation (IOF). Amgen was neither involved in the design nor writing of the report. We are grateful to Anastasia Soulié Mlotek and Dominique Pierroz of the IOF for their help in the administration of SCOPE. The authors thank Louise Brent (National Office of Clinical Audit) for her assistance. The report has been reviewed by the members of the SCOPE Consultation Panel and the relevant IOF National societies, and we are grateful for their local insights on the management of osteoporosis in each country. The source document has been reviewed and endorsed by the Committee of Scientific Advisors of the IOF and benefitted from their feedback.

**References**

1. Kanis JA, Norton N, Harvey NC, Jacobson T, Johansson H, Lorentzon M, McCloskey EV, Willers C, Borgström F (2021) SCOPE 2021: a new scorecard for osteoporosis in Europe. Arch Osteoporos 16:82. 10.1007/s11657-020-00871-9

2. World Health Organisation (1994) Assessment of fracture risk and its application to screening for postmenopausal osteoporosis. Report of a WHO Study Group. World Health Organ Tech Rep Ser, 1994/01/01 edn, pp 1-129

3. Johnell O, Kanis JA, Oden A, Sernbo I, Redlund-Johnell I, Petterson C, De Laet C, Jonsson B (2004) Mortality after osteoporotic fractures. Osteoporos Int 15:38-42

4. Hernlund E, Svedbom A, Ivergard M, Compston J, Cooper C, Stenmark J, McCloskey EV, Jonsson B, Kanis JA (2013) Osteoporosis in the European Union: medical management, epidemiology and economic burden. A report prepared in collaboration with the International Osteoporosis Foundation (IOF) and the European Federation of Pharmaceutical Industry Associations (EFPIA). Arch Osteoporos 8:136

5. Allen P, Pilar M, Walsh-Bailey C, Hooley C, Mazzucca S, Lewis CC, Mettert KD, Dorsey CN, Purtle J, Kepper MM, Baumann AA, Brownson RC (2020) Quantitative measures of health policy implementation determinants and outcomes: a systematic review. Implement Sci 15:47

6. Borgstrom F, Karlsson L, Ortsater G, Norton N, Halbout P, Cooper C, Lorentzon M, McCloskey EV, Harvey NC, Javaid MK, Kanis JA (2020) Fragility fractures in Europe: burden, management and opportunities. Arch Osteoporos 15:59

7. Kanis JA, Oden A, Johnell O, De Laet C, Jonsson B, Oglesby AK (2003) The components of excess mortality after hip fracture. Bone 32:468-473

8. National Clinical Guideline Centre (2011) The Management of Hip Fracture in Adults. In Centre NCG (ed)London

## **Epidemiology and economic burden of osteoporosis in Italy**

ML Brandi ∙ F Silveri ∙ M Rossini ∙ C Willers ∙ N Norton ∙ NC Harvey ∙ T Jacobson ∙ H Johansson ∙ M Lorentzon ∙ EV McCloskey ∙ F Borgström ∙ JA Kanis

**Introduction**

The scorecard summarises key indicators of the burden of osteoporosis and its management in the 27 member states of the European Union, as well as the UK and Switzerland (termed EU27+2) [1]. This country-specific report summarises the principal results for Italy.

**Methods**

The information obtained covers four domains: burden of osteoporosis and fractures; policy framework; service provision; and service uptake. Data were collected from numerous sources including previous research and IOF reports, and available registers which were used for additional analysis of resource utilization, costing and HRQoL data. Furthermore, country-specific information on osteoporosis management was obtained from each IOF member state via a questionnaire.

**Burden of disease**

The direct cost of incident fractures in Italy in 2019 was €5.44 billion. Added to this was the ongoing cost in 2019 from fractures that occurred before 2019, which amounted to €3.75 million (long-term disability). The cost of pharmacological intervention (assessment and treatment) was €259 million. Thus, the total direct cost (excluding the value of QALYs lost) amounted to €9.45 billion in 2019. Key metrics are presented in Table 1.

In 2019, the average direct cost of osteoporotic fractures in Italy was €156.3 per individual in the population, while in 2010 the average was €129.1 (after adjusting for inflation) representing an increase of 21% (€156.3 versus €129.1). The 2019 data ranked Italy in 5th place in terms of highest cost of osteoporotic fractures per capita in the EU27+2.

The cost of osteoporotic fractures in Italy accounted for approximately 6.0% of healthcare spending (i.e. €9.45 billion out of €153.85 billion in 2019), significantly higher than the EU27+2 average of 3.5% and ranked Italy 2nd place in the EU27+2 countries These numbers indicate a substantial impact of fragility fractures on the healthcare budget.

Using World Health Organization diagnostic criteria for osteoporosis based on the measurement of bone mineral density (BMD) [2], there were approximately 4,359,000 individuals with osteoporosis in Italy in 2019, of whom approximately 80% were women. The prevalence of osteoporosis in the total Italian population amounted to 6.3%, somewhat higher than the EU27+2 average (5.6%).

**Table 1** Key measures of burden of disease for Italy

**Table:** 

**Category**	**Measure**	**Estimate**	**Rank**
Burden of disease	Direct cost of incident fracture (€m)	5438.79	
	Long-term disability cost (€m)	3749.16	
	Intervention cost (€m)	258.61	
	Total cost (€m)	9446.55	
	QALYs lost (€m)	14980	
	Cost per capita (€)	156.32	5
	Proportion of healthcare spending	6.0%	2
	Prevalence of osteoporosis	6.3%	1

There were estimated to be 568,000 new fragility fractures in Italy in 2019, equivalent to 1,560 fractures/day (or 60 per hour). This was a slight increase compared to 2010, equivalent to an increment of 1.0 fractures/1000 individuals, totalling 20.6 fractures/ 1000 individuals in 2019.

Some osteoporotic fractures are associated with premature mortality [3]. In Italy, the annual number of deaths associated with a fracture event was estimated to be 105 per 100,000 individuals of the population aged 50 years or more, compared to the EU27+2 average of 116/100,000. The number of fracture-related deaths is comparable to or exceeds that for some of the most common causes of death such as lung cancer, diabetes, chronic lower respiratory diseases.

The remaining lifetime probability of hip fracture (%) at the ages of 50 years in men and women was 7.7% and 19.2%, respectively, placing Italy in the upper tertile of risk for both men and women.

The population of men and women age 50 years or more is projected to increase by 10.1% in men and women between 2019 and 2034, close to the EU27+2 average of 11.4%. The increases in men and women aged 75 years or more are even more marked and amount to 31.8% and 20.3%, respectively. The annual number of osteoporotic fractures in Italy is expected to increase by 133,000 to 702,000 in 2034.

**Policy framework** (Table 2)

Documentation of the burden of disease is an essential prerequisite to determine the resources that should be allocated to the diagnosis and treatment of the disorder. High quality national data on hip fracture rates have been identified in 18 of 29 countries, of which Italy is one. Data are collected on a national basis and include more than only hip fracture data.

Given that osteoporosis and fragility fractures are common and that effective treatments are widely available, the vast majority of patients with osteoporosis are preferably managed at the primary health care level by general practitioners (GPs), with specialist referral reserved for difficult complex cases. Primary care was the principal provider of the medical care for osteoporosis in Italy, as for 13 of the 28 countries where data were available.

Osteoporosis and metabolic bone disease is not a recognised specialty in most countries including Italy. Specialty care of osteoporosis in Italy is managed via other specialties including rheumatology, endocrinology, internal medicine, rehabilitation medicine and orthopaedics. Osteoporosis is however recognized as a component of specialty training. Although it is possible that these specialties educate their trainees adequately, the wide variation may reflect inconsistencies in patient care, training of primary care physicians and a suboptimal voice to “defend” the interests of those who work within the field of osteoporosis.

**Table 2** Policy framework for osteoporosis in Italy

**Table:** 

**Category**	**Measure**	**Estimate**
Policy framework	National fracture data availability	Yes
	OP recognized as a specialty	No
	OP primarily managed in primary care	Yes
	Other specialties involved	Rheumatology, Endocrinology, Internal medicine, Rehabilitation medicine, Orthopaedics
	Advocacy areas covered by patient organisation	Policy, capacity, peer support, research and development

The role of national patient organisations is to improve the care of patients and increase awareness and prevention of osteoporosis and related fractures among the general public. Advocacy by patient organisations can fall into four categories: policy, capacity building and education, peer support, research and development. For Italy, all four of the advocacy areas were covered by a patient organisation, which was the case for only 10 out of the 26 countries with at least one patient organisation.

**Service provision** (Table 3)

A wide variety of approved drug treatments is available for the management of osteoporosis [4]. Potential limitations of their use in member states relate to reimbursement policies which may impair the delivery of health care. Italy is one of the 12 (out of 27) countries that offer full reimbursement.

The assessment of bone mineral density forms a key component for the general management of osteoporosis, being used for diagnosis, risk prediction, selection of patients for treatment and monitoring of patients on treatment. In Italy, the number of DXA units expressed per million of the general population amounted to 23.5 which puts the country in 9^th^ place among the EU27+2.

The average waiting time for DXA ranged from 0 to 180 days across countries, and there was no clear relation between waiting times and the availability of DXA. In Italy, the estimated average waiting time for DXA amounted to 90 days. 23 countries reported shorter average waiting times.

**Table 3** Service provision for osteoporosis in Italy

**Table:** 

**Category**	**Measure**	**Estimate**	**Rank**
Service provision	Reimbursement of OP medications	100%	
	DXA units/million inhabitants	23.5	9
	DXA cost (€)	90	7
	FRAX risk assessment model available	Yes	
	Fracture liaison service density	1-10%	

Reimbursement for DXA scans varied between member states both in terms of the criteria required and level of reimbursement awarded. In Italy, the reimbursement was conditional and varied depending on the patient’s condition.

The effective targeting of treatment to those at highest risk of fracture requires an assessment of fracture risk. Risk assessment models for fractures, most usually based on FRAX, were available in 24 out of 29 countries, of which Italy was one. An additional risk assessment model, DeFRA, was also used in Italy. For Italy, guidance on the use of risk assessment within national guidelines was available, as in only 14 of the other countries.

Guidelines for the management of osteoporosis were available in Italy (as in 27 out of 29 countries). The guidelines in Italy included postmenopausal women specifically, as well as osteoporosis in men and secondary osteoporosis including glucocorticoid-induced osteoporosis.

Fracture liaison services (FLS), also known as osteoporosis coordinator programmes and care manager programmes, provide a system for the routine assessment and management of postmenopausal women and older men who have sustained a low trauma fracture. Fracture liaison services were reported for 1–10% of hospitals in Italy.

The use of indicators to systematically measure the quality of care provided to people with osteoporosis or associated fractures has expanded as a discipline within the past decade [5]. Italy was one of only few countries with national quality indicators in place.

**Service uptake** (Table 4)

The web-based usage of FRAX showed considerable heterogeneity in uptake between the countries. The average uptake for the EU27+2 was 1,555 sessions/million/year of the general population with an enormous range of 49 to 41,874 sessions/million. The use of FRAX in Italy amounted to 414 sessions/million in 2019, with a 20 percent decrease since 2011. It is notable, however, that Italy has its own risk assessment tools that are widely used [6, 7].

Many studies have demonstrated that a significant proportion of men and women at high fracture risk do not receive therapy for osteoporosis (the treatment gap) [8]. In the EU27+2 the average treatment gap was 71% but ranged from 32% to 87%. For Italy, the treatment gap amongst women amounted to 71% or 2,055,000 out of 2,889,000 characterised at risk, and it increased significantly compared to 2010. The average treatment gap among EU27+2 increased from 55% in 2010 to 71% in 2019.

**Table 4** Service uptake for osteoporosis in Italy

**Table:** 

**Category**	**Measure**	**Estimate**	**Rank**
Service uptake	Number of FRAX sessions/million people/yer	414	23
	Treatment gap for women eligible for treatment (%)	71	13
	Proportion surgically managed hip fractures	>90%	

About 5% of people with a hip fracture die within 1 month of their fracture [9]. A determinant of peri-operative morbidity and mortality is the time a patient takes to get to surgery [10]. For Italy, the average waiting time for hip fracture surgery after hospital admission was reported to be 2–3 days. The proportion of surgically managed hip fractures was reported to be over 90%.

**Scores and scorecard**

Scores were developed for Burden of disease and the healthcare provision (Policy framework, Service provision and Service uptake) in the EU27+2 countries. Italy scores resulted in a 14th place regarding Burden of disease. The combined healthcare provision scorecard resulted in an 8^th^ place for Italy. Thus, Italy presents as one of the high-burden high-provision countries among the EU27+2.

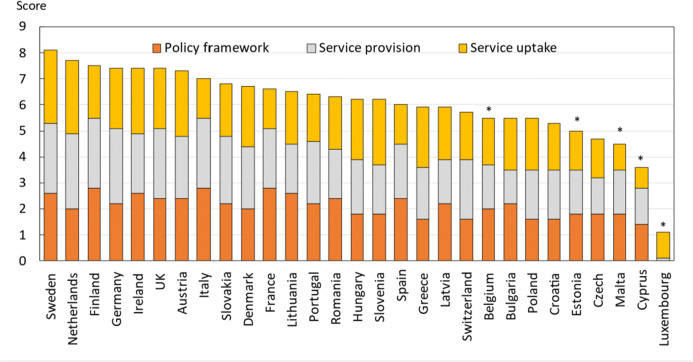



**Fig. 1** Scores by country for metrics related to policy framework, service provision and service uptake. The mean score for each of the 3 domains is given. An asterisk denotes that there was one or more missing metric which decreases the overall score

The first SCOPE was undertaken in 2010, almost 10 years previously. Fifteen of the 16 score card metrics on healthcare provision were used in the two surveys. Scores had improved or markedly improved in 15 countries, remained constant in 8 countries and worsened in 3 countries. For Italy, the scores were markedly improved.

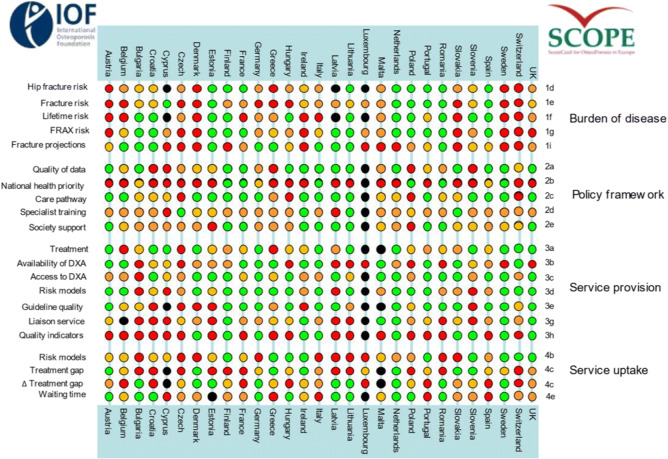



**Fig. 2** The scorecard for all the EU27+2 countries illustrating the scores across the four domains. The elements of each domain in each country were scored and coded using a traffic light system (red, orange, green). Black dots signify missing information

The second edition of the Scorecard for Osteoporosis in Europe (SCOPE 2021) allows health and policy professionals to assess key indicators on the healthcare provision for osteoporosis within countries and between countries within the EU 27+2. The scorecard is not intended as a prescriptive template. Thus, it does not set performance targets but may serve as a guide to the performance targets at which to aim in order to deliver the outcomes required.

**Acknowledgements**

SCOPE was supported by an unrestricted grant from Amgen to the International Osteoporosis Foundation (IOF). Amgen was neither involved in the design nor writing of the report. We are grateful to Anastasia Soulié Mlotek and Dominique Pierroz of the IOF for their help in the administration of SCOPE. We acknowledge the valuable assistance of the Italian Society for Osteoporosis, Mineral Metabolism and Bone Diseases (SIOMMMS). The report has been reviewed by the members of the SCOPE Consultation Panel and the relevant IOF National societies, and we are grateful for their local insights on the management of osteoporosis in each country. The source document has been reviewed and endorsed by the Committee of Scientific Advisors of the IOF and benefitted from their feedback.

**References**

1. Kanis JA, Norton N, Harvey NC, Jacobson T, Johansson H, Lorentzon M, McCloskey EV, Willers C, Borgström F (2021) SCOPE 2021: a new scorecard for osteoporosis in Europe. Arch Osteoporos 16:82. 10.1007/s11657-020-00871-9

2. World Health Organisation (1994) Assessment of fracture risk and its application to screening for postmenopausal osteoporosis. Report of a WHO Study Group. World Health Organ Tech Rep Ser, 1994/01/01 edn, pp 1-129

3. Johnell O, Kanis JA, Oden A, Sernbo I, Redlund-Johnell I, Petterson C, De Laet C, Jonsson B (2004) Mortality after osteoporotic fractures. Osteoporos Int 15:38-42

4. Hernlund E, Svedbom A, Ivergard M, Compston J, Cooper C, Stenmark J, McCloskey EV, Jonsson B, Kanis JA (2013) Osteoporosis in the European Union: medical management, epidemiology and economic burden. A report prepared in collaboration with the International Osteoporosis Foundation (IOF) and the European Federation of Pharmaceutical Industry Associations (EFPIA). Arch Osteoporos 8:136

5. Allen P, Pilar M, Walsh-Bailey C, Hooley C, Mazzucca S, Lewis CC, Mettert KD, Dorsey CN, Purtle J, Kepper MM, Baumann AA, Brownson RC (2020) Quantitative measures of health policy implementation determinants and outcomes: a systematic review. Implement Sci 15:47

6. Nuti R, Brandi ML, Checchia G, Di Munno O, Dominguez L, Falaschi P, Fiore CE, Iolascon G, Maggi S, Michieli R, Migliaccio S, Minisola S, Rossini M, Sessa G, Tarantino U, Toselli A, Isaia GC (2019) Guidelines for the management of osteoporosis and fragility fractures. Intern Emerg Med 14:85-102

7. Francesco L, Elisa B, Raffaella M, Alessandro P, Iacopo C, Giampiero M, Bruno F, Daniel PA, Luisa BM, Claudio C. Assessing Risk of Osteoporotic Fractures in Primary Care: Development and Validation of the FRA-HS Algorithm. Calcif Tissue Int. 2017 Jun;100(6):537-549. doi: 10.1007/s00223-016-0230-7. Epub 2017 Feb 3. Erratum in: Calcif Tissue Int. 2017 Jun;100(6):550

8. Borgstrom F, Karlsson L, Ortsater G, Norton N, Halbout P, Cooper C, Lorentzon M, McCloskey EV, Harvey NC, Javaid MK, Kanis JA (2020) Fragility fractures in Europe: burden, management and opportunities. Arch Osteoporos 15:59

9. Kanis JA, Oden A, Johnell O, De Laet C, Jonsson B, Oglesby AK (2003) The components of excess mortality after hip fracture. Bone 32:468-473

10. National Clinical Guideline Centre (2011) The Management of Hip Fracture in Adults. In Centre NCG (ed)London

## **Epidemiology and economic burden of osteoporosis in Latvia**

I Rasa ∙ C Willers ∙ N Norton ∙ NC Harvey ∙ T Jacobson ∙ H Johansson ∙ M Lorentzon ∙ EV McCloskey ∙ F Borgström ∙ JA Kanis

**Introduction**

The scorecard summarises key indicators of the burden of osteoporosis and its management in the 27 member states of the European Union, as well as the UK and Switzerland (termed EU27+2) [1]. This country-specific report summarises the principal results for Latvia.

**Methods**

The information obtained covers four domains: burden of osteoporosis and fractures; policy framework; service provision; and service uptake. Data were collected from numerous sources including previous research and IOF reports, and available registers which were used for additional analysis of resource utilization, costing and HRQoL data. Furthermore, country-specific information on osteoporosis management was obtained from each IOF member state via a questionnaire.

**Burden of disease**

The direct cost of incident fractures in Latvia in 2019 was €28.0 million. Added to this was the ongoing cost in 2019 from fractures that occurred before 2019, which amounted to €18.8 million (long-term disability). The cost of pharmacological intervention (assessment and treatment) was €1.8 million. Thus, the total direct cost (excluding the value of QALYs lost) amounted to €48.6 million in 2019. Key metrics are presented in Table 1.

In 2019, the average direct cost of osteoporotic fractures in Latvia was €25.2 per individual in the population, while in 2010 the average was €18.8 (after adjusting for inflation), representing an increase of 35% (€25.2 versus €18.8). The 2019 data rank Latvia in 26th place in terms of highest cost of osteoporotic fractures per capita in the EU27+2.

The cost of osteoporotic fractures in Latvia accounted for approximately 2.9% of healthcare spending (i.e. €49 million out of €1.6 billion in 2019), close to the EU27+2 average of 3.5%. These numbers indicate a substantial impact of fragility fractures on the healthcare budget.

Using World Health Organization diagnostic criteria for osteoporosis based on the measurement of bone mineral density (BMD) [2], there were approximately 124,800 individuals with osteoporosis in Latvia in 2019, of whom approximately 85% were women. The prevalence of osteoporosis in the total Latvian population amounted to 5.8%, on par with the EU27+2 average (5.6%).

**Table 1** Key measures of burden of disease for Latvia

**Table:** 

**Category**	**Measure**	**Estimate**	**Rank**
Burden of disease	Direct cost of incident fracture (€m)	28.04	
	Long-term disability cost (€m)	18.75	
	Intervention cost (€m)	1.84	
	Total cost (€m)	48.63	
	QALYs lost (€m)	170	
	Cost per capita (€)	25.24	26
	Proportion of healthcare spending	2.9%	17
	Prevalence of osteoporosis	5.8%	5

There is a paucity of fracture epidemiology in Latvia. There were estimated to be 15,800 new fragility fractures in Latvia in 2019, equivalent to 43 fractures/day (or 1.8 per hour). This was a slight increase compared to 2010, equivalent to an increment of 2.5 fractures/1000 individuals, totalling 20.1 fractures/ 1000 individuals in 2019.

Some osteoporotic fractures are associated with premature mortality [3]. In Latvia, the annual number of deaths associated with a fracture event was estimated to be 194 per 100,000 individuals of the population aged 50 years or more, compared to the EU27+2 average of 116/100,000. The number of fracture-related deaths is comparable to, or exceeds, that for some of the most common causes of death such as lung cancer, diabetes, chronic lower respiratory diseases.

The population in men and women age 50 years or more is projected to decrease by 3.1% between 2019 and 2034, compared to the EU27+2 average of increasing by 11.4%. The increases in men and women aged 75 years or more in Latvia are however more marked and amount to 18.9% and 6.1%, respectively. The annual number of osteoporotic fractures in Latvia is expected to increase by 1,300 to 17,100 in 2034.

**Policy framework** (Table 2)

Documentation of the burden of disease is an essential prerequisite to determine the resources that should be allocated to the diagnosis and treatment of the disorder. High quality national data on hip fracture rates have been identified in 18 of 29 countries, of which Latvia was not one.

Given that osteoporosis and fragility fractures are common and that effective treatments are widely available, the vast majority of patients with osteoporosis are preferably managed at the primary health care level by general practitioners (GPs), with specialist referral reserved for difficult complex cases. Primary care was the principal provider of the medical care for osteoporosis in Latvia, as for 13 of the 28 countries where data were available.

Osteoporosis and metabolic bone disease is not a recognised specialty in most countries including Latvia. In Latvia, osteoporosis is not recognized as a component of specialty training. This suggests the likelihood of inconsistencies in patient care, training of primary care physicians and a suboptimal voice to “defend” the interests of those who work within the field of osteoporosis. However, the Latvian Osteoporosis and Bone Metabolic Diseases Association (LOKMSA) organizes an Osteoporosis School to train physicians in various specialities.

**Table 2** Policy framework for osteoporosis in Latvia

**Table:** 

**Category**	**Measure**	**Estimate**
Policy framework	National fracture data availability	Yes
	OP recognized as a specialty	No
	OP primarily managed in primary care	Yes
	Other specialties involved	-
	Advocacy areas covered by patient organisation	Policy, capacity, peer support, research and development

The role of national patient organisations is to improve the care of patients and increase awareness and prevention of osteoporosis and related fractures among the general public. Advocacy by patient organisations can fall into four categories: policy, capacity building and education, peer support, research and development. For Latvia, all four of the advocacy areas were covered by a patient organisation, which was the case for only 10 out of the 26 countries with at least one patient organisation. The Latvian Osteoporosis and Bone Metabolic Diseases Association (LOKMSA) Patients’ Support Group organises various activities (www.osteoporozesasociacija.lv/) and regular publications (www.kauluveseliba.lv).

**Service provision** (Table 3)

A wide variety of approved drug treatments is available for the management of osteoporosis [4]. Potential limitations of their use in member states relate to reimbursement policies which may impair the delivery of health care. Twelve out of 27 countries offered full reimbursement, of which Latvia was not one.

The assessment of bone mineral density forms a key component for the general management of osteoporosis, being used for diagnosis, risk prediction, selection of patients for treatment and monitoring of patients on treatment. The Latvian Osteoporosis and Bone Metabolic Diseases Association indicate that there 24 DXA units available for clinical use of which 7 are state-owned and 17 are privately owned. This gives an availability of 13 DXA units/million of the general population. These data differ from the manufacturer estimates. Rather than ranking Latvia 27th as given in table 3, this uplifts the ranking to 12th.

The average waiting time for DXA ranged from 0 to 180 days across countries, and there was no clear relation between waiting times and the availability of DXA. In Latvia, the estimated average waiting time for DXA amounted to 17 days. 14 countries reported shorter average waiting times.

**Table 3** Service provision for osteoporosis in Latvia

**Table:** 

**Category**	**Measure**	**Estimate**	**Rank**
Service provision	Reimbursement of OP medications	50%	
	DXA units/million inhabitants	6.7	27
	DXA cost (€)	3.0	26
	FRAX risk assessment model available	No	
	Fracture liaison service density	No FLS	

Reimbursement for DXA scans varied between member states both in terms of the criteria required and level of reimbursement awarded. In Latvia, the reimbursement charge has remained unchanged in the last 5 years.

The effective targeting of treatment to those at highest risk of fracture requires an assessment of fracture risk. Risk assessment models for fractures, most usually based on FRAX, were available in 24 out of 29 countries, Latvia belonging to the remaining five countries without risk assessment model.

Guidelines for the management of osteoporosis were available in Latvia (as in a total of 27 out of 29 countries). The guidelines in Latvia included postmenopausal women specifically, as well as for osteoporosis in men and for secondary osteoporosis including glucocorticoid-induced osteoporosis. The latest iteration was in 2012 (https://www.osteoporozesasociacija.lv/uploads/LOKMSA-vadl%C4%ABnijas-2012.pdf).

Fracture liaison services (FLS), also known as osteoporosis coordinator programmes and care manager programmes, provide a system for the routine assessment and management of postmenopausal women and older men who have sustained a low trauma fracture. No fracture liaison services were reported from Latvia (together with 7 other countries).

The use of indicators to systematically measure the quality of care provided to people with osteoporosis or associated fractures has expanded as a discipline within the past decade [5]. No use of national quality indicators was reported for Latvia.

**Service uptake** (Table 4)

The web-based usage of FRAX showed considerable heterogeneity in uptake between the countries. The average uptake for the EU27+2 was 1,555 sessions/million/year of the general population with an enormous range of 49 to 41,874 sessions/million. The usage for Latvia amounted to 218 sessions/million in 2019, with an almost 300 percent increase since 2011.

Many studies have demonstrated that a significant proportion of men and women at high fracture risk do not receive therapy for osteoporosis (the treatment gap) [6]. In the EU27+2 the average treatment gap was 71% but ranged from 32 to 87%. For Latvia, the treatment gap amongst women amounted to 78% or 57,000 out of 74,000 characterised at risk and it had decreased somewhat compared to 2010. The average treatment gap among EU27+2 increased from 55% in 2010 to 71% in 2019.

**Table 4** Service uptake for osteoporosis in Latvia

**Table:** 

**Category**	**Measure**	**Estimate**	**Rank**
Service uptake	Number of FRAX sessions/million people/year	218	26
	Treatment gap for women eligible for treatment (%)	78	17
	Proportion surgically managed hip fractures	>90%	

About 5% of people with a hip fracture die within 1 month of their fracture [7]. A determinant of peri-operative morbidity and mortality is the time a patient takes to get to surgery [8]. For Latvia, the average waiting time for hip fracture surgery after hospital admission was reported to be less than 24 h. The proportion of surgically managed hip fractures was reported to be over 90%.

**Scores and scorecard**

Scores were developed for Burden of disease and the healthcare provision (Policy framework, Service provision and Service uptake) in the EU27+2 countries. Latvia scores resulted in a 27th place regarding Burden of disease. The combined healthcare provision scorecard resulted in a 19th place for Latvia. Thus, Latvia presents as one of the higher burden lower-provision countries among the EU27+2.

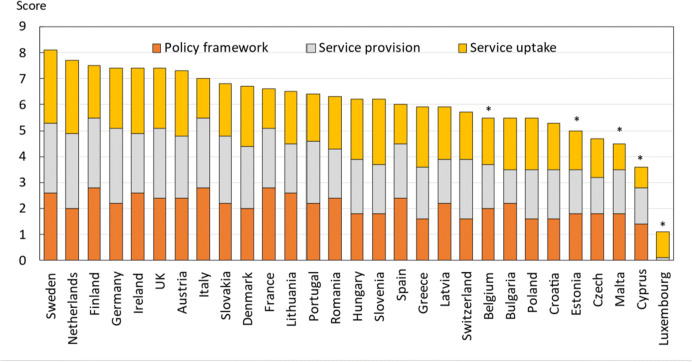



**Fig. 1** Scores by country for metrics related to policy framework, service provision and service uptake. The mean score for each of the 3 domains is given. An asterisk denotes that there was one or more missing metric which decreases the overall score

The first SCOPE was undertaken in 2010, almost 10 years previously. Fifteen of the 16 score card metrics on healthcare provision were used in the two surveys. Scores had improved or markedly improved in 15 countries, remained constant in 8 countries and worsened in 3 countries. For Latvia the scores were almost unchanged.

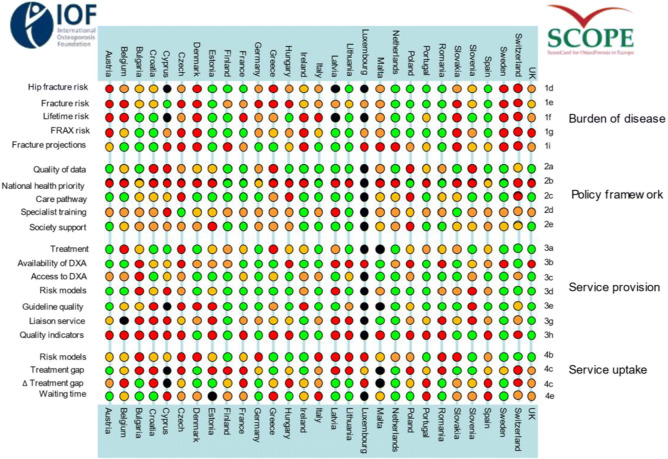



**Fig. 2** The scorecard for all the EU27+2 countries illustrating the scores across the four domains. The elements of each domain in each country were scored and coded using a traffic light system (red, orange, green). Black dots signify missing information

The second edition of the Scorecard for Osteoporosis in Europe (SCOPE 2021) allows health and policy professionals to assess key indicators on the healthcare provision for osteoporosis within countries and between countries within the EU 27+2. The scorecard is not intended as a prescriptive template. Thus, it does not set performance targets but may serve as a guide to the performance targets at which to aim in order to deliver the outcomes required.

**Acknowledgements**

SCOPE was supported by an unrestricted grant from Amgen to the International Osteoporosis Foundation (IOF). Amgen was neither involved in the design nor writing of the report. We are grateful to Anastasia Soulié Mlotek and Dominique Pierroz of the IOF for their help in the administration of SCOPE. The report has been reviewed by the members of the SCOPE Consultation Panel and the relevant IOF National societies, and we are grateful for their local insights on the management of osteoporosis in each country. The source document has been reviewed and endorsed by the Committee of Scientific Advisors of the IOF and benefitted from their feedback.

**References**

1. Kanis JA, Norton N, Harvey NC, Jacobson T, Johansson H, Lorentzon M, McCloskey EV, Willers C, Borgström F (2021) SCOPE 2021: a new scorecard for osteoporosis in Europe. Arch Osteoporos 16:82. 10.1007/s11657-020-00871-9

2. World Health Organisation (1994) Assessment of fracture risk and its application to screening for postmenopausal osteoporosis. Report of a WHO Study Group. World Health Organ Tech Rep Ser, 1994/01/01 edn, pp 1-129

3. Johnell O, Kanis JA, Oden A, Sernbo I, Redlund-Johnell I, Petterson C, De Laet C, Jonsson B (2004) Mortality after osteoporotic fractures. Osteoporos Int 15:38-42

4. Hernlund E, Svedbom A, Ivergard M, Compston J, Cooper C, Stenmark J, McCloskey EV, Jonsson B, Kanis JA (2013) Osteoporosis in the European Union: medical management, epidemiology and economic burden. A report prepared in collaboration with the International Osteoporosis Foundation (IOF) and the European Federation of Pharmaceutical Industry Associations (EFPIA). Arch Osteoporos 8:136

5. Allen P, Pilar M, Walsh-Bailey C, Hooley C, Mazzucca S, Lewis CC, Mettert KD, Dorsey CN, Purtle J, Kepper MM, Baumann AA, Brownson RC (2020) Quantitative measures of health policy implementation determinants and outcomes: a systematic review. Implement Sci 15:47

6. Borgstrom F, Karlsson L, Ortsater G, Norton N, Halbout P, Cooper C, Lorentzon M, McCloskey EV, Harvey NC, Javaid MK, Kanis JA (2020) Fragility fractures in Europe: burden, management and opportunities. Arch Osteoporos 15:59

7. Kanis JA, Oden A, Johnell O, De Laet C, Jonsson B, Oglesby AK (2003) The components of excess mortality after hip fracture. Bone 32:468-473

8. National Clinical Guideline Centre (2011) The Management of Hip Fracture in Adults. In Centre NCG (ed)London

## **Epidemiology and economic burden of osteoporosis in Lithuania**

A Vidmantas ∙ M Tamulaitiene ∙ C Willers ∙ N Norton ∙ NC Harvey ∙ T Jacobson ∙ H Johansson ∙ M Lorentzon ∙ EV McCloskey ∙ F Borgström ∙ JA Kanis

**Introduction**

The scorecard summarises key indicators of the burden of osteoporosis and its management in the 27 member states of the European Union, as well as the UK and Switzerland (termed EU27+2) [1]. This country-specific report summarises the principal results for Lithuania.

**Methods**

The information obtained covers four domains: burden of osteoporosis and fractures; policy framework; service provision; and service uptake. Data were collected from numerous sources including previous research and IOF reports, and available registers which were used for additional analysis of resource utilization, costing and HRQoL data. Furthermore, country-specific information on osteoporosis management was obtained from each IOF member state via a questionnaire.

**Burden of disease**

The direct cost of incident fractures in Lithuania in 2019 was €53.1 million. Added to this was the ongoing cost in 2019 from fractures that occurred before 2019, which amounted to €35.1 million (long-term disability). The cost of pharmacological intervention (assessment and treatment) was €2.8 million. Thus, the total direct cost (excluding the value of QALYs lost) amounted to €91.0 million in 2019. Key metrics are presented in Table 1.

In 2019, the average direct cost of osteoporotic fractures in Lithuania was €32.6 per individual in the population, while in 2010 the average was €15.5 (after adjusting for inflation), representing an increase of 111% (€32.6 versus €15.5). The 2019 data rank Lithuania in 23rd place in terms of highest cost of osteoporotic fractures per capita in the EU27+2.

The cost of osteoporotic fractures in Lithuania accounted for approximately 3.2% of healthcare spending (i.e. €91 million out of €2.75 billion in 2019), close to the EU27+2 average of 3.5%. These numbers indicate a substantial impact of fragility fractures on the healthcare budget.

Using World Health Organization diagnostic criteria for osteoporosis based on the measurement of bone mineral density (BMD) [2], there were approximately 181,000 individuals with osteoporosis in Lithuania in 2019, of whom approximately 84% were women. The prevalence of osteoporosis in the total Lithuanian population amounted to 5.3%, on par with the EU27+2 average (5.6%).

**Table 1** Key measures of burden of disease for Lithuania

**Table:** 

**Category**	**Measure**	**Estimate**	**Rank**
Burden of disease	Direct cost of incident fracture (€m)	53.14	
	Long-term disability cost (€m)	35.08	
	Intervention cost (€m)	2.79	
	Total cost (€m)	91.01	
	QALYs lost (€m)	258	
	Cost per capita (€)	32.63	23
	Proportion of healthcare spending	3.2%	14
	Prevalence of osteoporosis	5.3%	18

There were estimated to be 23,000 new fragility fractures in Lithuania in 2019, equivalent to 63 fractures/day (or 2.6 per hour). This was a significant increase compared to 2010, equivalent to an increment of 6.4 fractures/1000 individuals, totalling 19.8 fractures/ 1000 individuals in 2019.

Some osteoporotic fractures are associated with premature mortality [3]. In Lithuania, the annual number of deaths associated with a fracture event was estimated to be 172 per 100,000 individuals of the population aged 50 years or more, compared to the EU27+2 average of 116/100,000. The number of fracture-related deaths is comparable to, or exceeds, that for some of the most common causes of death such as lung cancer, diabetes, chronic lower respiratory diseases.

The remaining lifetime probability of hip fracture (%) at the ages of 50 years in men and women was 4.4% and 11.3%, respectively, placing Lithuania in the bottom tertile of risk for both men and women.

The population in men and women age 50 years or more is projected to decrease by 4.2% between 2019 and 2034, whilst they in the EU27+2 on average were projected to increase of 11.4%. The projected increases in men and women aged 75 years or more in Lithuania amount to 17.2% and 11.9%, respectively. The annual number of osteoporotic fractures in Lithuania is expected to increase by 3,800 to almost 27,000 in 2034.

**Policy framework** (Table 2)

Documentation of the burden of disease is an essential prerequisite to determine the resources that should be allocated to the diagnosis and treatment of the disorder. High quality national data on hip fracture rates have been identified in 18 of 29 countries, but Lithuania was not deemed one of these. Data are however collected on a national basis and include more than only hip fracture data.

Given that osteoporosis and fragility fractures are common and that effective treatments are widely available, the vast majority of patients with osteoporosis are preferably managed at the primary health care level by general practitioners (GPs), with specialist referral reserved for difficult complex cases. Primary care was the principal provider of the medical care for osteoporosis in Lithuania, as for 13 of the 28 countries where data were available.

Osteoporosis and metabolic bone disease is not a recognised specialty in most countries but this is however the case in Lithuania. Specialty care of osteoporosis in Lithuania is also managed via other specialties including internal medicine, geriatrics, endocrinology, rheumatology and orthopaedics and osteoporosis is recognized as a component of specialty training. Although it is possible that these specialties educate their trainees adequately, the wide variation may reflect inconsistencies in patient care, training of primary care physicians and a suboptimal voice to “defend” the interests of those who work within the field of osteoporosis.

**Table 2** Policy framework for osteoporosis in Lithuania

**Table:** 

**Category**	**Measure**	**Estimate**
Policy framework	National fracture data availability	Yes
	OP recognized as a specialty	Yes
	OP primarily managed in primary care	Yes
	Other specialties involved	Internal Medicine, Geriatrics, Endocrinology, Rheumatology, Orthopaedics
	Advocacy areas covered by patient organisation	Policy, capacity, peer support, research and development

The role of national patient organisations is to improve the care of patients and increase awareness and prevention of osteoporosis and related fractures among the general public. Advocacy by patient organisations can fall into four categories: policy, capacity building and education, peer support, research and development. For Lithuania, all four of the advocacy areas were covered by a patient organisation, which was the case for only 10 out of the 26 countries with at least one patient organisation.

**Service provision** (Table 3)

A wide variety of approved drug treatments is available for the management of osteoporosis [4]. Potential limitations of their use in member states relate to reimbursement policies which may impair the delivery of health care. Lithuania is one of the 12 (out of 27) countries that offer full reimbursement.

The assessment of bone mineral density forms a key component for the general management of osteoporosis, being used for diagnosis, risk prediction, selection of patients for treatment and monitoring of patients on treatment. In Lithuania, the number of DXA units expressed per million of the general population amounted to 8.0 which puts the country in 22^nd^ place among the EU27+2.

The average waiting time for DXA ranged from 0 to 180 days across countries, and there was no clear relation between waiting times and the availability of DXA. In Lithuania, the estimated average waiting time for DXA amounted to seven days. Only four countries reported shorter average waiting times.

**Table 3** Service provision for osteoporosis in Lithuania

**Table:** 

**Category**	**Measure**	**Estimate**	**Rank**
Service provision	Reimbursement of OP medications	100%	
	DXA units/million inhabitants	8.0	22
	DXA cost (€)	30	21
	FRAX risk assessment model available	Yes	
	Fracture liaison service density	No FLS	

Reimbursement for DXA scans varied between member states both in terms of the criteria required and level of reimbursement awarded. In Lithuania, the reimbursement was conditional and varied depending on the patient’s condition.

The effective targeting of treatment to those at highest risk of fracture requires an assessment of fracture risk. Risk assessment models for fractures, most usually based on FRAX, were available in 24 out of 29 countries, of which Lithuania was one. For Lithuania, guidance on the use of risk assessment within national guidelines was available, as in only 14 of the other countries. Guidelines for the management of osteoporosis were available in Lithuania (as in a total of 27 out of 29 countries). The guidelines in Lithuania included postmenopausal women specifically.

Fracture liaison services (FLS), also known as osteoporosis coordinator programmes and care manager programmes, provide a system for the routine assessment and management of postmenopausal women and older men who have sustained a low trauma fracture. No fracture liaison services were reported from Lithuania (together with 7 other countries).

The use of indicators to systematically measure the quality of care provided to people with osteoporosis or associated fractures has expanded as a discipline within the past decade [5]. No use of national quality indicators was reported for Lithuania.

**Service uptake** (Table 4)

The web-based usage of FRAX showed considerable heterogeneity in uptake between the countries. The average uptake for the EU27+2 was 1,555 sessions/million/year of the general population with an enormous range of 49 to 41,874 sessions/million. The use of FRAX in Lithuania amounted to 131 sessions/million in 2019, with a 360 percent increase since 2011.

Many studies have demonstrated that a significant proportion of men and women at high fracture risk do not receive therapy for osteoporosis (the treatment gap) [6]. In the EU27+2 the average treatment gap was 71% but ranged from 32 to 87%. For Lithuania, the treatment gap amongst women amounted to 82% or 88,000 out of 107,000 characterised at risk and it had decreased somewhat compared to 2010. The average treatment gap among EU27+2 increased from 55% in 2010 to 71% in 2019.

**Table 4** Service uptake for osteoporosis in Lithuania

**Table:** 

**Category**	**Measure**	**Estimate**	**Rank**
Service uptake	Number of FRAX sessions/million people/year	131	27
	Treatment gap for women eligible for treatment (%)	82	23
	Proportion surgically managed hip fractures	>90%	

About 5% of people with a hip fracture die within 1 month of their fracture [7]. A determinant of peri-operative morbidity and mortality is the time a patient takes to get to surgery [8]. For Lithuania, the average waiting time for hip fracture surgery after hospital admission was reported to be less than 24 h. The proportion of surgically managed hip fractures was reported to be over 90%.

**Scores and scorecard**

Scores were developed for Burden of disease and the healthcare provision (Policy framework, Service provision and Service uptake) in the EU27+2 countries. Lithuania scores resulted in a 24th place regarding Burden of disease. The combined healthcare provision scorecard resulted in a 12th place for Lithuania. Thus, Lithuania presents as one of the lower-burden higher-provision countries among the EU27+2.

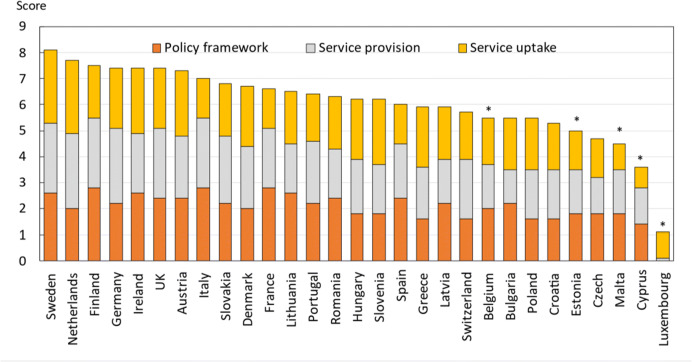



**Fig. 1** Scores by country for metrics related to policy framework, service provision and service uptake. The mean score for each of the 3 domains is given. An asterisk denotes that there was one or more missing metric which decreases the overall score

The first SCOPE was undertaken in 2010, almost 10 years previously. Fifteen of the 16 score card metrics on healthcare provision were used in the two surveys. Scores had improved or markedly improved in 15 countries, remained constant in 8 countries and worsened in 3 countries. For Lithuania the scores were markedly improved.

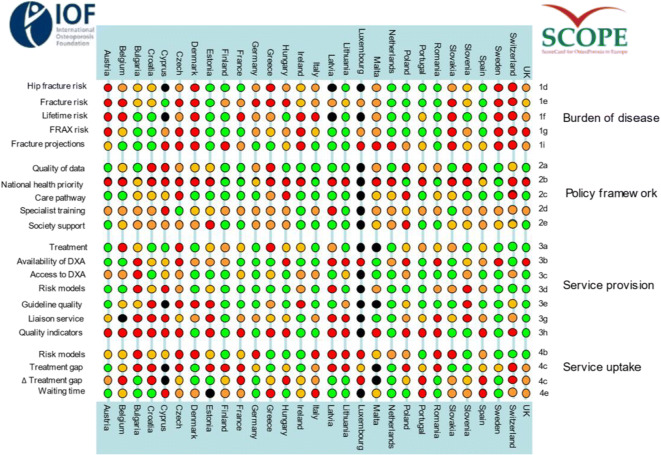



**Fig. 2** The scorecard for all the EU27+2 countries illustrating the scores across the four domains. The elements of each domain in each country were scored and coded using a traffic light system (red, orange, green). Black dots signify missing information

The second edition of the Scorecard for Osteoporosis in Europe (SCOPE 2021) allows health and policy professionals to assess key indicators on the healthcare provision for osteoporosis within countries and between countries within the EU 27+2. The scorecard is not intended as a prescriptive template. Thus, it does not set performance targets but may serve as a guide to the performance targets at which to aim in order to deliver the outcomes required.

**Acknowledgements**

SCOPE was supported by an unrestricted grant from Amgen to the International Osteoporosis Foundation (IOF). Amgen was neither involved in the design nor writing of the report. We are grateful to Anastasia Soulié Mlotek and Dominique Pierroz of the IOF for their help in the administration of SCOPE. The report has been reviewed by the members of the SCOPE Consultation Panel and the relevant IOF National societies, and we are grateful for their local insights on the management of osteoporosis in each country. The source document has been reviewed and endorsed by the Committee of Scientific Advisors of the IOF and benefitted from their feedback.

**References**

1. Kanis JA, Norton N, Harvey NC, Jacobson T, Johansson H, Lorentzon M, McCloskey EV, Willers C, Borgström F (2021) SCOPE 2021: a new scorecard for osteoporosis in Europe. Arch Osteoporos 16:82. 10.1007/s11657-020-00871-9

2. World Health Organisation (1994) Assessment of fracture risk and its application to screening for postmenopausal osteoporosis. Report of a WHO Study Group. World Health Organ Tech Rep Ser, 1994/01/01 edn, pp 1-129

3. Johnell O, Kanis JA, Oden A, Sernbo I, Redlund-Johnell I, Petterson C, De Laet C, Jonsson B (2004) Mortality after osteoporotic fractures. Osteoporos Int 15:38-42

4. Hernlund E, Svedbom A, Ivergard M, Compston J, Cooper C, Stenmark J, McCloskey EV, Jonsson B, Kanis JA (2013) Osteoporosis in the European Union: medical management, epidemiology and economic burden. A report prepared in collaboration with the International Osteoporosis Foundation (IOF) and the European Federation of Pharmaceutical Industry Associations (EFPIA). Arch Osteoporos 8:136

5. Allen P, Pilar M, Walsh-Bailey C, Hooley C, Mazzucca S, Lewis CC, Mettert KD, Dorsey CN, Purtle J, Kepper MM, Baumann AA, Brownson RC (2020) Quantitative measures of health policy implementation determinants and outcomes: a systematic review. Implement Sci 15:47

6. Borgstrom F, Karlsson L, Ortsater G, Norton N, Halbout P, Cooper C, Lorentzon M, McCloskey EV, Harvey NC, Javaid MK, Kanis JA (2020) Fragility fractures in Europe: burden, management and opportunities. Arch Osteoporos 15:59

7. Kanis JA, Oden A, Johnell O, De Laet C, Jonsson B, Oglesby AK (2003) The components of excess mortality after hip fracture. Bone 32:468-473

8. National Clinical Guideline Centre (2011) The Management of Hip Fracture in Adults. In Centre NCG (ed)London

## **Epidemiology and economic burden of osteoporosis in Luxembourg**

C Willers ∙ N Norton ∙ NC Harvey ∙ T Jacobson ∙ H Johansson ∙ M Lorentzon ∙ EV McCloskey ∙ F Borgström ∙ JA Kanis

**Introduction**

The scorecard summarises key indicators of the burden of osteoporosis and its management in the 27 member states of the European Union, as well as the UK and Switzerland (termed EU27+2) [1]. This country-specific report summarises the principal results for Luxembourg.

**Methods**

The information obtained covers four domains: burden of osteoporosis and fractures; policy framework; service provision; and service uptake. Data were collected from numerous sources including previous research and IOF reports, and available registers which were used for additional analysis of resource utilization, costing and HRQoL data. Furthermore, country-specific information on osteoporosis management was obtained from each IOF member state via a questionnaire.

**Burden of disease**

The direct cost of incident fractures in Luxembourg in 2019 was €28.3 million. Added to this was the ongoing cost in 2019 from fractures that occurred before 2019, which amounted to €10.8 million (long-term disability). The cost of pharmacological intervention (assessment and treatment) was €1.6 million. Thus, the total direct cost (excluding the value of QALYs lost) amounted to €40.7 million in 2019. Key metrics are presented in Table 1.

In 2019, the average direct cost of osteoporotic fractures in Luxembourg was €66.8 per individual in the population, while in 2010 the average was €47.5 (after adjusting for inflation), representing an increase of 41% (€66.8 versus €47.5). The 2019 numbers put Luxembourg in 17^th^ place in terms of highest cost of osteoporotic fractures per capita in the EU27+2.

The cost of osteoporotic fractures in Luxembourg accounted for approximately 1.3% of healthcare spending (i.e. €41 million out of €3.05 billion in 2019), significantly lower than the EU27+2 average of 3.5% and placed Luxembourg last in the rank order across the EU27+2 countries.

Using World Health Organization diagnostic criteria for osteoporosis based on the measurement of bone mineral density (BMD) [2], there were approximately 29,600 individuals with osteoporosis in Luxembourg in 2019, of whom almost 80% were women. The prevalence of osteoporosis in the total population amounted to 4.3%, somewhat lower than the EU27+2 average (5.6%).

**Table 1** Key measures of burden of disease for Luxembourg

**Table:** 

**Category**	**Measure**	**Estimate**	**Rank**
Burden of disease	Direct cost of incident fracture (€m)	28.26	
	Long-term disability cost (€m)	10.78	
	Intervention cost (€m)	1.58	
	Total cost (€m)	40.62	
	QALYs lost (€m)	317	
	Cost per capita (€)	66.84	17
	Proportion of healthcare spending	1.3%	29
	Prevalence of osteoporosis	4.3%	26

There is a paucity of fracture epidemiology in Luxembourg. There were estimated to be 4,000 new fragility fractures in Luxembourg in 2019, equivalent to 11 fractures/day (or almost 0.5 per hour). This was a slight increase compared to 2010, equivalent to an increment of 1.7 fractures/1000 individuals, totalling 18.8 fractures/ 1000 individuals in 2019.

Some osteoporotic fractures are associated with premature mortality [3]. In Luxembourg, the annual number of deaths associated with a fracture event was estimated to be 100 per 100,000 individuals of the population aged 50 years or more, compared to the EU27+2 average of 116/100,000. The number of fracture-related deaths is comparable to or exceeds that for some of the most common causes of death such as lung cancer, diabetes, chronic lower respiratory diseases.

The population in men and women age 50 years or more is projected to increase by 30.3% between 2019 and 2034, which is much more than the EU27+2 average of 11.4%. The increases in men and women aged 75 years or more are even more marked and amount to 81.2% and 45.0%, respectively. The annual number of osteoporotic fractures in Luxembourg is expected to increase by 1,800 to 5,800 in 2034.

**Policy framework**

Documentation of the burden of disease is an essential prerequisite to determine the resources that should be allocated to the diagnosis and treatment of the disorder. High quality national data on hip fracture rates have been identified in 18 of 29 countries. No information on fracture data was available for Luxembourg.

**Service provision** (Table 2)

A wide variety of approved drug treatments is available for the management of osteoporosis [4]. Potential limitations of their use in member states relate to reimbursement policies which may impair the delivery of health care. Twelve out of 27 countries offered full reimbursement. No information on reimbursement rates was available for Luxembourg.

The assessment of bone mineral density forms a cornerstone for the general management of osteoporosis, being used for diagnosis, risk prediction, selection of patients for treatment and monitoring of patients on treatment. In Luxembourg, the number of DXA units expressed per million of the general population amounted to 1.7 which puts the country in last place among the EU27+2. The availability of TBS was however relatively high in Luxembourg.

The average waiting time for DXA ranged from 0 to 180 days across countries, and there was no clear relation between waiting times and the availability of DXA. Reimbursement for DXA scans varied between member states both in terms of the criteria required and level of reimbursement awarded. There was no information available on waiting times and reimbursement in Luxembourg.

**Table 2** Service provision for osteoporosis in Luxembourg

**Table:** 

**Category**	**Measure**	**Estimate**	**Rank**
Service provision	Reimbursement of OP medications	N/A	
	DXA units/million inhabitants	1.7	29
	DXA cost (€)	N/A	
	FRAX risk assessment model available	No	
	Fracture liaison service density	N/A	

The effective targeting of treatment to those at highest risk of fracture requires an assessment of fracture risk. Risk assessment models for fractures, most usually based on FRAX, were available in 24 out of 29 countries, where Luxembourg belonged to the remaining five countries. Guidelines for the management of osteoporosis were available in Luxembourg (as in 27 out of 29 countries). The guidelines in Luxembourg were confined to postmenopausal women.

Fracture liaison services (FLS), also known as osteoporosis coordinator programmes and care manager programmes, provide a system for the routine assessment and management of postmenopausal women and older men who have sustained a low trauma fracture. There was no information available on fracture liaison services for Luxembourg.

The use of indicators to systematically measure the quality of care provided to people with osteoporosis or associated fractures has expanded as a discipline within the past decade [5]. No use of national quality indicators was reported for Luxembourg.

**Service uptake** (Table 3)

The web-based usage of FRAX showed considerable heterogeneity in uptake between the countries. The average uptake for the EU27+2 was 1,555 sessions/million/year of the general population with an enormous range of 49 to 41,874 sessions/million. The usage for Luxembourg amounted to 507 sessions/million in 2019, indicating a 78 percent decrease since 2011.

Many studies have demonstrated that a significant proportion of men and women at high fracture risk do not receive therapy for osteoporosis (the treatment gap) [6]. In the EU27+2 the average treatment gap was 71% but ranged from 32 to 87%. For Luxembourg, the treatment gap amongst women amounted to 71% or 14,000 out of 19,000 characterised at risk and increased significantly compared to 2010. The average treatment gap among EU27+2 increased from 55% in 2010 to 71% in 2019.

**Table 3** Service uptake for osteoporosis in Luxembourg

**Table:** 

**Category**	**Measure**	**Estimate**	**Rank**
Service uptake	Number of FRAX sessions/million people/year	507	20
	Treatment gap for women eligible for treatment (%)	74	14
	Proportion surgically managed hip fractures	N/A	

About 5% of people with a hip fracture die within 1 month of their fracture [7]. A determinant of peri-operative morbidity and mortality is the time a patient takes to get to surgery [8]. For Luxembourg, the average waiting time for hip fracture surgery after hospital admission was reported to be 1–2 days for 2010 whilst no new information was received for the present assessment.

**Scores and scorecard**

Scores were developed for Burden of disease and the healthcare provision (Policy framework, Service provision and Service uptake) in the EU27+2 countries. Luxembourg scores resulted in a 22nd place regarding Burden of disease. The combined healthcare provision scorecard resulted in a last (29th) place for Luxembourg. Thus, Luxembourg presents as one of the low-burden low-provision countries among the EU27+2.

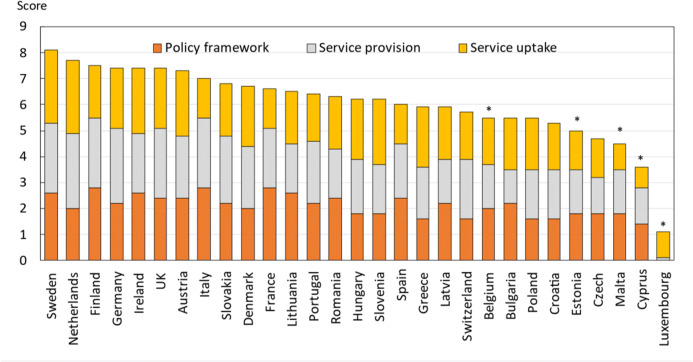



**Fig. 1** Scores by country for metrics related to policy framework, service provision and service uptake. The mean score for each of the 3 domains is given. An asterisk denotes that there was one or more missing metric which decreases the overall score

The first SCOPE was undertaken in 2010, almost 10 years previously. Fifteen of the 16 score card metrics on healthcare provision were used in the two surveys. Scores had improved or markedly improved in 15 countries, remained constant in 8 countries and worsened in 3 countries.

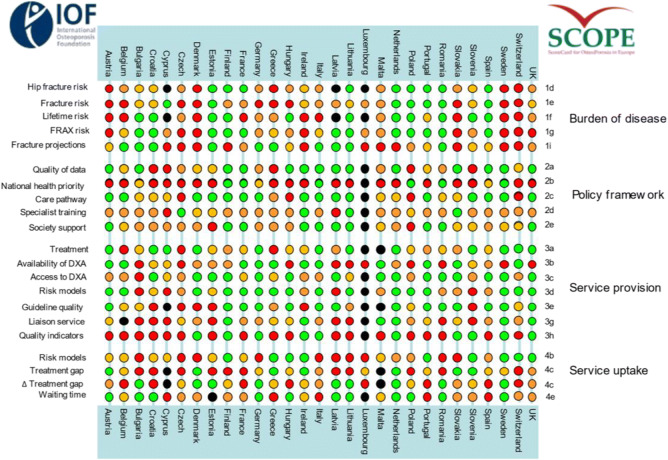



**Fig. 2** The scorecard for all the EU27+2 countries illustrating the scores across the four domains. The elements of each domain in each country were scored and coded using a traffic light system (red, orange, green). Black dots signify missing information

The second edition of the Scorecard for Osteoporosis in Europe (SCOPE 2021) allows health and policy professionals to assess key indicators on the healthcare provision for osteoporosis within countries and between countries within the EU 27+2. The scorecard is not intended as a prescriptive template. Thus, it does not set performance targets but may serve as a guide to the performance targets at which to aim in order to deliver the outcomes required.

**Acknowledgements**

SCOPE was supported by an unrestricted grant from Amgen to the International Osteoporosis Foundation (IOF). Amgen was neither involved in the design nor writing of the report. We are grateful to Anastasia Soulié Mlotek and Dominique Pierroz of the IOF for their help in the administration of SCOPE. The report has been reviewed by the members of the SCOPE Consultation Panel and the relevant IOF National societies, and we are grateful for their local insights on the management of osteoporosis in each country. The source document has been reviewed and endorsed by the Committee of Scientific Advisors of the IOF and benefitted from their feedback.

**References**

1. Kanis JA, Norton N, Harvey NC, Jacobson T, Johansson H, Lorentzon M, McCloskey EV, Willers C, Borgström F (2021) SCOPE 2021: a new scorecard for osteoporosis in Europe. Arch Osteoporos 16:82. 10.1007/s11657-020-00871-9

2. World Health Organisation (1994) Assessment of fracture risk and its application to screening for postmenopausal osteoporosis. Report of a WHO Study Group. World Health Organ Tech Rep Ser, 1994/01/01 edn, pp 1-129

3. Johnell O, Kanis JA, Oden A, Sernbo I, Redlund-Johnell I, Petterson C, De Laet C, Jonsson B (2004) Mortality after osteoporotic fractures. Osteoporos Int 15:38-42

4. Hernlund E, Svedbom A, Ivergard M, Compston J, Cooper C, Stenmark J, McCloskey EV, Jonsson B, Kanis JA (2013) Osteoporosis in the European Union: medical management, epidemiology and economic burden. A report prepared in collaboration with the International Osteoporosis Foundation (IOF) and the European Federation of Pharmaceutical Industry Associations (EFPIA). Arch Osteoporos 8:136

5. Allen P, Pilar M, Walsh-Bailey C, Hooley C, Mazzucca S, Lewis CC, Mettert KD, Dorsey CN, Purtle J, Kepper MM, Baumann AA, Brownson RC (2020) Quantitative measures of health policy implementation determinants and outcomes: a systematic review. Implement Sci 15:47

6. Borgstrom F, Karlsson L, Ortsater G, Norton N, Halbout P, Cooper C, Lorentzon M, McCloskey EV, Harvey NC, Javaid MK, Kanis JA (2020) Fragility fractures in Europe: burden, management and opportunities. Arch Osteoporos 15:59

7. Kanis JA, Oden A, Johnell O, De Laet C, Jonsson B, Oglesby AK (2003) The components of excess mortality after hip fracture. Bone 32:468-473

8. National Clinical Guideline Centre (2011) The Management of Hip Fracture in Adults. In Centre NCG (ed)London

## **Epidemiology and economic burden of osteoporosis in Malta**

R Galea ∙ Neville Calleja ∙ C Willers ∙ N Norton ∙ NC Harvey ∙ T Jacobson ∙ H Johansson ∙ M Lorentzon ∙ EV McCloskey ∙ F Borgström ∙ JA Kanis

**Introduction**

The scorecard summarises key indicators of the burden of osteoporosis and its management in the 27 member states of the European Union, as well as the UK and Switzerland (termed EU27+2) [1]. This country-specific report summarises the principal results for Malta.

**Methods**

The information obtained covers four domains: burden of osteoporosis and fractures; policy framework; service provision; and service uptake. Data were collected from numerous sources including previous research and IOF reports, and available registers which were used for additional analysis of resource utilization, costing and HRQoL data. Furthermore, country-specific information on osteoporosis management was obtained from each IOF member state via a questionnaire.

**Burden of disease**

The direct cost of incident fractures in Malta in 2019 was €18.6 million. Added to this was the ongoing cost in 2019 from fractures that occurred before 2019, which amounted to €8.4 million (long-term disability). The cost of pharmacological intervention (assessment and treatment) was €2.1 million. Thus, the total direct cost (excluding the value of QALYs lost) amounted to €29.1 million in 2019. Key metrics are presented in Table 1.

In 2019, the average direct cost of osteoporotic fractures in Malta was €60.1 per individual in the population, while in 2010 the average was €45.3 (after adjusting for inflation), representing an increase of 33% (€151.8 versus €104.8). The 2019 data ranked Malta in 18th place in terms of highest cost of osteoporotic fractures per capita in the EU27+2.

The cost of osteoporotic fractures in Malta accounted for approximately 2.5% of healthcare spending (i.e. €29 million out of €1.06 billion in 2019), which is lower than the EU27+2 average of 3.5%.

Using World Health Organization diagnostic criteria for osteoporosis based on the measurement of bone mineral density (BMD) [2], there were approximately 23,000 individuals with osteoporosis in Malta in 2019, of whom almost 80% were women. The prevalence of osteoporosis in the total Maltese population amounted to 4.9%, on a par with the EU27+2 average (5.6%).

**Table 1** Key measures of burden of disease for Malta

**Table:** 

**Category**	**Measure**	**Estimate**	**Rank**
Burden of disease	Direct cost of incident fracture (€m)	18.59	
	Long-term disability cost (€m)	8.41	
	Intervention cost (€m)	2.07	
	Total cost (€m)	29.06	
	QALYs lost (€m)	65	
	Cost per capita (€)	60.1	18
	Proportion of healthcare spending	2.5%	20
	Prevalence of osteoporosis	4.9%	22

There were estimated to be 3,200 new fragility fractures in Malta in 2019, equivalent to 9 fractures/day (or 0.4 per hour). This was a slight increase compared to 2010, equivalent to an increment of 0.9 fractures/1000 individuals, totalling 18.3 fractures/ 1000 individuals in 2019.

Some osteoporotic fractures are associated with premature mortality [3]. In Malta, the annual number of deaths associated with a fracture event was estimated to be 84 per 100,000 individuals of the population aged 50 years or more, compared to the EU27+2 average of 116/100,000. The number of fracture-related deaths is comparable to, or exceeds, that for some of the most common causes of death such as lung cancer, diabetes, chronic lower respiratory diseases.

The remaining lifetime probability of hip fracture (%) at the ages of 50 years in men and women was 5.8% and 14.2%, respectively, placing Malta in the mid tertile of risk for both men and women.

The population of men and women age 50 years or more is projected to increase by 14.7% between 2019 and 2034, close to the EU27+2 average of 11.4%. The increases in men and women aged 75 years or more are even more marked and amount to 76.2% and 57.3%, respectively. The annual number of osteoporotic fractures in Malta is expected to increase by 1,500 to 4,700 in 2034.

**Policy framework** (Table 2)

Documentation of the burden of disease is an essential prerequisite to determine the resources that should be allocated to the diagnosis and treatment of the disorder. High quality national data on hip fracture rates have been identified in 18 of 29 countries, of which Malta is one. Data are collected on a national basis and include more than only hip fracture data.

Given that osteoporosis and fragility fractures are common and that effective treatments are widely available, the vast majority of patients with osteoporosis are preferably managed at the primary health care level by general practitioners (GPs), with specialist referral reserved for difficult complex cases. Primary care was the principal provider of the medical care for osteoporosis for 13 of the 28 countries where data were available although this was not the case for Malta, where osteoporosis was primarily devolved to rheumatological care. Osteoporosis and metabolic bone disease is not a recognised specialty in most countries including Malta. Specialty care of osteoporosis in Malta is managed via rheumatology but also via other specialties including orthopaedics, gynaecology, rehabilitation medicine, endocrinology and geriatrics. Osteoporosis is recognized as a component of specialty training. Although it is possible that these specialties educate their trainees adequately, the wide variation may reflect inconsistencies in patient care, training of primary care physicians and a suboptimal voice to “defend” the interests of those who work within the field of osteoporosis.

**Table 2** Policy framework for osteoporosis in Malta

**Table:** 

**Category**	**Measure**	**Estimate**
Policy framework	National fracture data availability	No
	OP recognized as a specialty	No
	OP primarily managed in primary care	No
	Other specialties involved	Rheumatology, Orthopaedics, Gynaecology, Rehabilitation Medicine, Endocrinology, Geriatrics
	Advocacy areas covered by patient organisation	Policy, capacity, peer support

The role of national patient organisations is to improve the care of patients and increase awareness and prevention of osteoporosis and related fractures among the general public. Advocacy by patient organisations can fall into four categories: policy, capacity building and education, peer support, research and development. For Malta, three of these advocacy areas were covered by a patient organisation. All four advocacy areas were covered for only 10 out of the 26 countries with at least one patient organisation.

**Service provision** (Table 3)

A wide variety of approved drug treatments is available for the management of osteoporosis [4]. Potential limitations of their use in member states relate to reimbursement policies which may impair the delivery of health care. Twelve out of 27 countries offered full reimbursement. For Malta no information on reimbursement was available. Since publication of the SCOPE report [1], we are advised that treatment is reimbursed for steroid induced osteoporosis.

The assessment of bone mineral density forms a key component for the general management of osteoporosis, being used for diagnosis, risk prediction, selection of patients for treatment and monitoring of patients on treatment. In Malta, the number of DXA units expressed per million of the general population amounted to 24.6 which puts the country in 7th place among the EU27+2. DXA is provided at no cost to the patient. Assessment of trabecular bone score was not available in Malta.

The average waiting time for DXA ranged from 0 to 180 days across countries, and there was no clear relation between waiting times and the availability of DXA. In Malta, the estimated average waiting time for DXA amounted to 30 days. Seventeen countries reported shorter average waiting times.

**Table 3** Service provision for osteoporosis in Malta

**Table:** 

**Category**	**Measure**	**Estimate**	**Rank**
Service provision	Reimbursement of OP medications	N/A	
	DXA units/million inhabitants	24.6	7
	DXA cost (€)	0	27
	FRAX risk assessment model available	Yes	
	Fracture liaison service density	>50%	

Reimbursement for DXA scans varied between member states both in terms of the criteria required and level of reimbursement awarded. In Malta, the reimbursement was unconditional.

The effective targeting of treatment to those at highest risk of fracture requires an assessment of fracture risk. Risk assessment models for fractures, most usually based on FRAX, were available in 24 out of 29 countries, of which Malta was one. For Malta, no guidance on the use of risk assessment within national guidelines was available. Guidelines for the management of osteoporosis were not available in Malta (as in totally only two countries).

Fracture liaison services (FLS), also known as osteoporosis coordinator programmes and care manager programmes, provide a system for the routine assessment and management of postmenopausal women and older men who have sustained a low trauma fracture. Fracture liaison services were reported for more than 50% of hospitals for Malta.

The use of indicators to systematically measure the quality of care provided to people with osteoporosis or associated fractures has expanded as a discipline within the past decade [5]. No use of national quality indicators was reported for Malta.

**Service uptake** (Table 4)

The web-based usage of FRAX showed considerable heterogeneity in uptake between the countries. The average uptake for the EU27+2 was 1,555 sessions/million/year of the general population with an enormous range of 49 to 41,874 sessions/million. The use of FRAX in Malta amounted to 1,541 sessions/million in 2019, suggesting a 91 percent decrease since 2011.

Many studies have demonstrated that a significant proportion of men and women at high fracture risk do not receive therapy for osteoporosis (the treatment gap) [6]. In the EU27+2 the average treatment gap was 71% but ranged from 32% to 87%. The average treatment gap among EU27+2 increased from 55% in 2010 to 71% in 2019. For Malta, there was no information available on the treatment gap.

**Table 4** Service uptake for osteoporosis in Malta

**Table:** 

**Category**	**Measure**	**Estimate**	**Rank**
Service uptake	Number of FRAX sessions/million people/year	1541	12
	Treatment gap for women eligible for treatment (%)	N/A	
	Proportion surgically managed hip fractures	>90%	

About 5% of people with a hip fracture die within 1 month of their fracture [7]. A determinant of peri-operative morbidity and mortality is the time a patient takes to get to surgery [8]. For Malta, the average waiting time for hip fracture surgery after hospital admission was reported to be 1-2 days. The proportion of surgically managed hip fractures was reported to be over 90%.

**Scores and scorecard**

Scores were developed for Burden of disease and the healthcare provision (Policy framework, Service provision and Service uptake) in the EU27+2 countries. Malta scores resulted in a 10th place regarding Burden of disease. The combined healthcare provision scorecard resulted in a 27th place for Malta. Thus, Malta presents as one of the eight higher-burden lower-provision countries among the EU27+2.

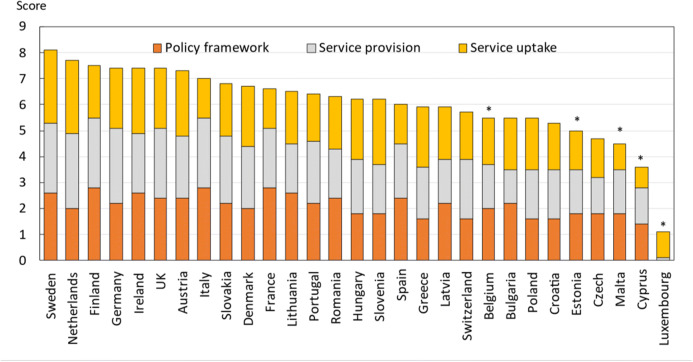



**Fig. 1** Scores by country for metrics related to policy framework, service provision and service uptake. The mean score for each of the 3 domains is given. An asterisk denotes that there was one or more missing metric which decreases the overall score

The first SCOPE was undertaken in 2010, almost 10 years previously. Fifteen of the 16 score card metrics on healthcare provision were used in the two surveys. Scores had improved or markedly improved in 15 countries, remained constant in 8 countries and worsened in 3 countries. For Malta the scores were much improved.

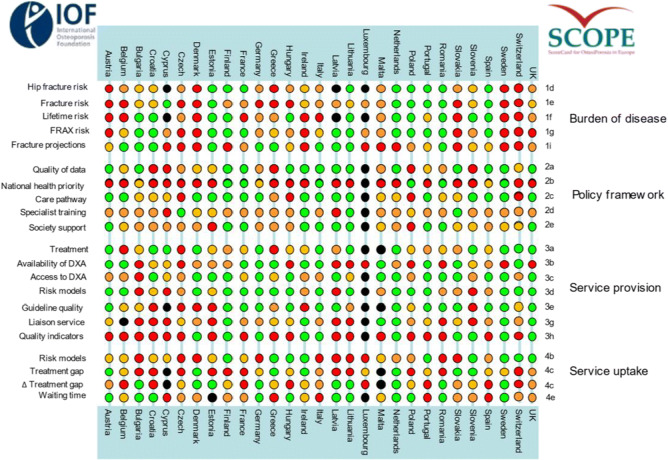



**Fig. 2** The scorecard for all the EU27+2 countries illustrating the scores across the four domains. The elements of each domain in each country were scored and coded using a traffic light system (red, orange, green). Black dots signify missing information

The second edition of the Scorecard for Osteoporosis in Europe (SCOPE 2021) allows health and policy professionals to assess key indicators on the healthcare provision for osteoporosis within countries and between countries within the EU 27+2. The scorecard is not intended as a prescriptive template. Thus, it does not set performance targets but may serve as a guide to the performance targets at which to aim in order to deliver the outcomes required.

**Acknowledgements**

SCOPE was supported by an unrestricted grant from Amgen to the International Osteoporosis Foundation (IOF). Amgen was neither involved in the design nor writing of the report. We are grateful to Anastasia Soulié Mlotek and Dominique Pierroz of the IOF for their help in the administration of SCOPE. The report has been reviewed by the members of the SCOPE Consultation Panel and the relevant IOF National societies, and we are grateful for their local insights on the management of osteoporosis in each country. The source document has been reviewed and endorsed by the Committee of Scientific Advisors of the IOF and benefitted from their feedback.

**References**

1. Kanis JA, Norton N, Harvey NC, Jacobson T, Johansson H, Lorentzon M, McCloskey EV, Willers C, Borgström F (2021) SCOPE 2021: a new scorecard for osteoporosis in Europe. Arch Osteoporos 16:82. 10.1007/s11657-020-00871-9

2. World Health Organisation (1994) Assessment of fracture risk and its application to screening for postmenopausal osteoporosis. Report of a WHO Study Group. World Health Organ Tech Rep Ser, 1994/01/01 edn, pp 1-129

3. Johnell O, Kanis JA, Oden A, Sernbo I, Redlund-Johnell I, Petterson C, De Laet C, Jonsson B (2004) Mortality after osteoporotic fractures. Osteoporos Int 15:38-42

4. Hernlund E, Svedbom A, Ivergard M, Compston J, Cooper C, Stenmark J, McCloskey EV, Jonsson B, Kanis JA (2013) Osteoporosis in the European Union: medical management, epidemiology and economic burden. A report prepared in collaboration with the International Osteoporosis Foundation (IOF) and the European Federation of Pharmaceutical Industry Associations (EFPIA). Arch Osteoporos 8:136

5. Allen P, Pilar M, Walsh-Bailey C, Hooley C, Mazzucca S, Lewis CC, Mettert KD, Dorsey CN, Purtle J, Kepper MM, Baumann AA, Brownson RC (2020) Quantitative measures of health policy implementation determinants and outcomes: a systematic review. Implement Sci 15:47

6. Borgstrom F, Karlsson L, Ortsater G, Norton N, Halbout P, Cooper C, Lorentzon M, McCloskey EV, Harvey NC, Javaid MK, Kanis JA (2020) Fragility fractures in Europe: burden, management and opportunities. Arch Osteoporos 15:59

7. Kanis JA, Oden A, Johnell O, De Laet C, Jonsson B, Oglesby AK (2003) The components of excess mortality after hip fracture. Bone 32:468-473

8. National Clinical Guideline Centre (2011) The Management of Hip Fracture in Adults. In Centre NCG (ed)London

## **Epidemiology and economic burden of osteoporosis in the Netherlands**

GEMP Willemsen-de Mey ∙ H van den Broek ∙ H Witte ∙ C Willers ∙ N Norton ∙ NC Harvey ∙ T Jacobson ∙ H Johansson ∙ M Lorentzon ∙ EV McCloskey ∙ F Borgström ∙ JA Kanis

**Introduction**

The scorecard summarises key indicators of the burden of osteoporosis and its management in the 27 member states of the European Union, as well as the UK and Switzerland (termed EU27+2) [1]. This country-specific report summarises the principal results for the Netherlands.

**Methods**

The information obtained covers four domains: burden of osteoporosis and fractures; policy framework; service provision; and service uptake. Data were collected from numerous sources including previous research and IOF reports, and available registers which were used for additional analysis of resource utilization, costing and HRQoL data. Furthermore, country-specific information on osteoporosis management was obtained from each IOF member state via a questionnaire.

**Burden of disease**

The direct cost of incident fractures in the Netherlands in 2019 was €652.7 million. Added to this was the ongoing cost in 2019 from fractures that occurred before 2019, which amounted to €708.4 million (long-term disability). The cost of pharmacological intervention (assessment and treatment) was €42.8 million. Thus, the total direct cost (excluding the value of QALYs lost) amounted to €1.4 billion in 2019. Key metrics are presented in Table 1.

In 2019, the average direct cost of osteoporotic fractures in the Netherlands was €81.5 per individual in the population, while in 2010 the average was €55.2 (after adjusting for inflation), giving an increase of 48% (€81.5 versus €55.2). The 2019 numbersdata rank the Netherlands in 15^th^ place in terms of highest cost of osteoporotic fractures per capita in the EU27+2.

The cost of osteoporotic fractures in the Netherlands accounted for approximately 1.8% of healthcare spending (i.e. €1.4 billion out of €75.0 billion in 2019), which is significantly lower than the EU27+2 average of 3.5%.

Using World Health Organization diagnostic criteria for osteoporosis based on the measurement of bone mineral density (BMD) [2], there were approximately 976,000 individuals with osteoporosis in the Netherlands in 2019, of whom almost 80% were women. The prevalence of osteoporosis in the total population amounted to 4.9%, somewhat lower than the EU27+2 average (5.6%).

**Table 1** Key measures of burden of disease for the Netherlands

**Table:** 

**Category**	**Measure**	**Estimate**	**Rank**
Burden of disease	Direct cost of incident fracture (€m)	652.72	
	Long-term disability cost (€m)	708.35	
	Intervention cost (€m)	42.82	
	Total cost (€m)	1403.88	
	QALYs lost (€m)	3735	
	Cost per capita (€)	81.47	15
	Proportion of healthcare spending	1.8%	28
	Prevalence of osteoporosis	4.9%	23

There were estimated to be 99,600 new fragility fractures in the Netherlands in 2019, equivalent to 273 fractures/day (or 11 per hour). This was a slight increase compared to 2010, equivalent to an increment of 1.2 fractures/1000 individuals, totalling 14.1 fractures/ 1000 individuals in 2019.

Some osteoporotic fractures are associated with premature mortality [3]. In the Netherlands, the annual number of deaths associated with a fracture event was estimated to be 82 per 100,000 individuals of the population aged 50 years or more, compared to the EU27+2 average of 116/100,000. The number of fracture-related deaths is comparable to or exceeds that for some of the most common causes of death such as lung cancer, diabetes, chronic lower respiratory diseases.

The remaining lifetime probability of hip fracture (%) at the ages of 50 years in men and women was 5.4% and 12.5%, respectively, placing the Netherlands in the mid tertile of risk for both men and women.

The population of men and women age 50 years or more is projected to increase by 9.5% between 2019 and 2034, close to the EU27+2 average of 11.4%. The increases in men and women aged 75 years or more are even more marked and amount to 67.0% and 49.1%, respectively. The annual number of osteoporotic fractures in the Netherlands is expected to increase by 37,000 to almost 137,000 in 2034.

**Policy framework** (Table 2)

Documentation of the burden of disease is an essential prerequisite to determine the resources that should be allocated to the diagnosis and treatment of the disorder. High quality national data on hip fracture rates have been identified in 18 of 29 countries, of which the Netherlands is one. Data are collected on a national basis and include hip fracture data.

Given that osteoporosis and fragility fractures are common and that effective treatments are widely available, the vast majority of patients with osteoporosis are preferably managed at the primary health care level by general practitioners (GPs), with specialist referral reserved for difficult complex cases. Primary care was the principal provider of the medical care for osteoporosis for 13 of the 28 countries where data were available but that was not the case for the Netherlands.

Osteoporosis and metabolic bone disease is not a recognised specialty in most countries including the Netherlands. Specialty care of osteoporosis is managed via other specialties including endocrinology, internal medicine, orthopaedics, gynaecology, rheumatology and traumatology. Osteoporosis is however recognized as a component of specialty training. Although it is possible that these specialties educate their trainees adequately, the wide variation may reflect inconsistencies in patient care, training of primary care physicians and a suboptimal voice to “defend” the interests of those who work within the field of osteoporosis.

**Table 2** Policy framework for osteoporosis in the Netherlands

**Table:** 

**Category**	**Measure**	**Estimate**
Policy framework	National fracture data availability	Yes
	OP recognized as a specialty	No
	OP primarily managed in primary care	No
	Other specialties involved	Endocrinology, Internal medicine, Orthopaedics, Gynaecology, Rheumatology, Traumatology
	Advocacy areas covered by patient organisation	Policy, capacity, peer support

The role of national patient organisations is to improve the care of patients and increase awareness and prevention of osteoporosis and related fractures among the general public. Advocacy by patient organisations can fall into four categories: policy, capacity building and education, peer support, research and development. For the Netherlands, three of these were covered by a patient organisation. All four advocacy areas were covered for only 10 out of the 26 countries with at least one patient organisation.

**Service provision** (Table 3)

A wide variety of approved drug treatments is available for the management of osteoporosis [4]. Potential limitations of their use in member states relate to reimbursement policies which may impair the delivery of health care. The Netherlands is one of the 12 (out of 27) countries that offer full reimbursement.

The assessment of bone mineral density forms a key component for the general management of osteoporosis, being used for diagnosis, risk prediction, selection of patients for treatment and monitoring of patients on treatment. In the Netherlands, the number of DXA units expressed per million of the general population amounted to 12.3 which puts the country in 17th place among the EU27+2.

The average waiting time for DXA ranged from 0 to 180 days across countries, and there was no clear relation between waiting times and the availability of DXA. In the Netherlands, the estimated average waiting time for DXA amounted to 14 days. Nine countries reported shorter average waiting times.

**Table 3** Service provision for osteoporosis in the Netherlands

**Table:** 

**Category**	**Measure**	**Estimate**	**Rank**
Service provision	Reimbursement of OP medications	100%	
	DXA units/million inhabitants	12.3	17
	DXA cost (€)	100	4
	FRAX risk assessment model available	Yes	
	Fracture liaison service density	>50%	

Reimbursement for DXA scans varied between member states both in terms of the criteria required and level of reimbursement awarded. In the Netherlands, the reimbursement was unconditional.

The effective targeting of treatment to those at highest risk of fracture requires an assessment of fracture risk. Risk assessment models for fractures, most usually based on FRAX, were available in 24 out of 29 countries, of which the Netherlands was one. An additional risk assessment model, CBO, was also used in the Netherlands. Guidance on the use of risk assessment within national guidelines was available, as in only 14 of the other countries.

Guidelines for the management of osteoporosis were available in the Netherlands (as in 27 out of 29 countries). The guidelines in the Netherlands included postmenopausal women specifically, as well as osteoporosis in men and secondary osteoporosis including glucocorticoid-induced osteoporosis.

Fracture liaison services (FLS), also known as osteoporosis coordinator programmes and care manager programmes, provide a system for the routine assessment and management of postmenopausal women and older men who have sustained a low trauma fracture. Fracture liaison services were reported for more than 50% of hospitals for the Netherlands.

The use of indicators to systematically measure the quality of care provided to people with osteoporosis or associated fractures has expanded as a discipline within the past decade [5]. The Netherlands was one of few countries with national quality indicators in place.

**Service uptake** (Table 4)

The web-based usage of FRAX showed considerable heterogeneity in uptake between the countries. The average uptake for the EU27+2 was 1,555 sessions/million/year of the general population with an enormous range of 49 to 41,874 sessions/million. The use of FRAX for the Netherlands amounted to 609 sessions/million in 2019, with a 16 percent increase since 2011.

Many studies have demonstrated that a significant proportion of men and women at high fracture risk do not receive therapy for osteoporosis (the treatment gap) [6]. In the EU27+2 the average treatment gap was 71% but ranged from 32 to 87%. For the Netherlands, the treatment gap amongst women amounted to 56% or 388,000 out of 696,000 characterised at risk. The treatment gap did not change significantly compared to 2010, whilst the average treatment gap among EU27+2 increased from 55% in 2010 to 71% in 2019.

**Table 4** Service uptake for osteoporosis in the Netherlands

**Table:** 

**Category**	**Measure**	**Estimate**	**Rank**
Service uptake	Number of FRAX sessions/million people/year	609	18
	Treatment gap for women eligible for treatment (%)	56	6
	Proportion surgically managed hip fractures	>90%	

About 5% of people with a hip fracture die within 1 month of their fracture [7]. A determinant of peri-operative morbidity and mortality is the time a patient takes to get to surgery [8]. For the Netherlands, the average waiting time for hip fracture surgery after hospital admission was reported to be less than 24 h, implying a reduction in waiting time compared to 2010 (waiting time of 1–2 days). The proportion of surgically managed hip fractures was reported to be over 90%.

**Scores and scorecard**

Scores were developed for Burden of disease and the healthcare provision (Policy framework, Service provision and Service uptake) in the EU27+2 countries. The Netherlands scores resulted in a 20th place regarding Burden of disease. The combined healthcare provision scorecard resulted in a 2nd place for the Netherlands after only Sweden. Thus, the Netherlands presents as one of the low-burden high-provision countries among the EU27+2.

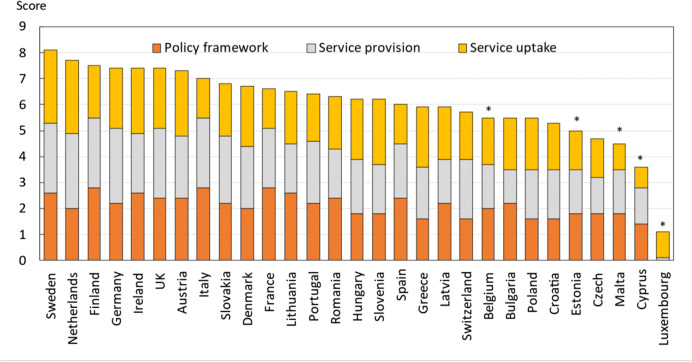



**Fig. 1** Scores by country for metrics related to policy framework, service provision and service uptake. The mean score for each of the 3 domains is given. An asterisk denotes that there was one or more missing metric which decreases the overall score

The first SCOPE was undertaken in 2010, almost 10 years previously. Fifteen of the 16 score card metrics on healthcare provision were used in the two surveys. Scores had improved or markedly improved in 15 countries, remained constant in 8 countries and worsened in 3 countries. For the Netherlands the scores were almost unchanged.

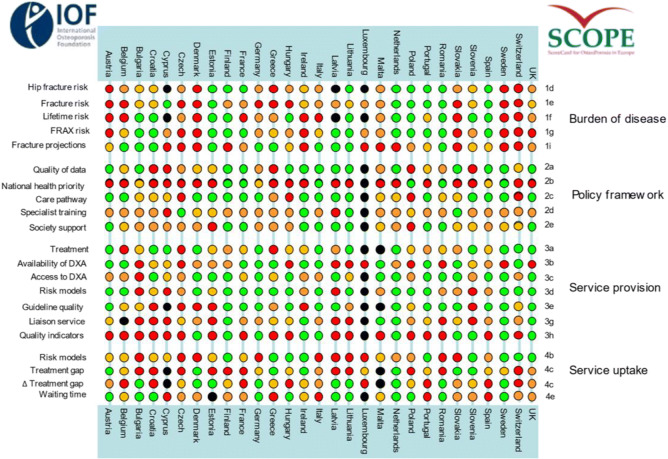



**Fig. 2** The scorecard for all the EU27+2 countries illustrating the scores across the four domains. The elements of each domain in each country were scored and coded using a traffic light system (red, orange, green). Black dots signify missing information

The second edition of the Scorecard for Osteoporosis in Europe (SCOPE 2021) allows health and policy professionals to assess key indicators on the healthcare provision for osteoporosis within countries and between countries within the EU 27+2. The scorecard is not intended as a prescriptive template. Thus, it does not set performance targets but may serve as a guide to the performance targets at which to aim in order to deliver the outcomes required.

**Acknowledgements**

SCOPE was supported by an unrestricted grant from Amgen to the International Osteoporosis Foundation (IOF). Amgen was neither involved in the design nor writing of the report. We are grateful to Anastasia Soulié Mlotek and Dominique Pierroz of the IOF for their help in the administration of SCOPE. We thank the two Dutch organisations Nederlandse Vereniging voor Reumatologie and Nederlandse Orthopaedische Vereniging for their assistance. The report has been reviewed by the members of the SCOPE Consultation Panel and the relevant IOF National societies, and we are grateful for their local insights on the management of osteoporosis in each country. The source document has been reviewed and endorsed by the Committee of Scientific Advisors of the IOF and benefitted from their feedback.

**References**

1. Kanis JA, Norton N, Harvey NC, Jacobson T, Johansson H, Lorentzon M, McCloskey EV, Willers C, Borgström F (2021) SCOPE 2021: a new scorecard for osteoporosis in Europe. Arch Osteoporos 16:82. 10.1007/s11657-020-00871-9

2. World Health Organisation (1994) Assessment of fracture risk and its application to screening for postmenopausal osteoporosis. Report of a WHO Study Group. World Health Organ Tech Rep Ser, 1994/01/01 edn, pp 1-129

3. Johnell O, Kanis JA, Oden A, Sernbo I, Redlund-Johnell I, Petterson C, De Laet C, Jonsson B (2004) Mortality after osteoporotic fractures. Osteoporos Int 15:38-42

4. Hernlund E, Svedbom A, Ivergard M, Compston J, Cooper C, Stenmark J, McCloskey EV, Jonsson B, Kanis JA (2013) Osteoporosis in the European Union: medical management, epidemiology and economic burden. A report prepared in collaboration with the International Osteoporosis Foundation (IOF) and the European Federation of Pharmaceutical Industry Associations (EFPIA). Arch Osteoporos 8:136

5. Allen P, Pilar M, Walsh-Bailey C, Hooley C, Mazzucca S, Lewis CC, Mettert KD, Dorsey CN, Purtle J, Kepper MM, Baumann AA, Brownson RC (2020) Quantitative measures of health policy implementation determinants and outcomes: a systematic review. Implement Sci 15:47

6. Borgstrom F, Karlsson L, Ortsater G, Norton N, Halbout P, Cooper C, Lorentzon M, McCloskey EV, Harvey NC, Javaid MK, Kanis JA (2020) Fragility fractures in Europe: burden, management and opportunities. Arch Osteoporos 15:59

7. Kanis JA, Oden A, Johnell O, De Laet C, Jonsson B, Oglesby AK (2003) The components of excess mortality after hip fracture. Bone 32:468-473

8. National Clinical Guideline Centre (2011) The Management of Hip Fracture in Adults. In Centre NCG (ed)London

## **Epidemiology and economic burden of osteoporosis in Poland**

E Czerwinski ∙ JE Badurski ∙ C Willers ∙ N Norton ∙ NC Harvey ∙ T Jacobson ∙ H Johansson ∙ M Lorentzon ∙ EV McCloskey ∙ F Borgström ∙ JA Kanis

**Introduction**

The scorecard summarises key indicators of the burden of osteoporosis and its management in the 27 member states of the European Union, as well as the UK and Switzerland (termed EU27+2) [1]. This country-specific report summarises the principal results for Poland.

**Methods**

The information obtained covers four domains: burden of osteoporosis and fractures; policy framework; service provision; and service uptake. Data were collected from numerous sources including previous research and IOF reports, and available registers which were used for additional analysis of resource utilization, costing and HRQoL data. Furthermore, country-specific information on osteoporosis management was obtained from each IOF member state via a questionnaire.

**Burden of disease**

The direct cost of incident fractures in Poland in 2019 was €332.9 million. Added to this was the ongoing cost in 2019 from fractures that occurred before 2019, which amounted to €347.3 million (long-term disability). The cost of pharmacological intervention (assessment and treatment) was €13.5 million. Thus, the total direct cost (excluding the value of QALYs lost) amounted to €693.7 million in 2019. Key metrics are presented in Table 1.

In 2019, the average direct cost of osteoporotic fractures in Poland was €18.3 per individual in the population, while in 2010 the average was €17.7 (after adjusting for inflation), giving an increase of 3% (€18.3 versus €17.7). The 2019 data rank Poland in 28^th^ place in terms of highest cost of osteoporotic fractures per capita in the EU27+2.

The cost of osteoporotic fractures in Poland accounted for approximately 2.2% of healthcare spending (i.e. €694 million out of €30.8 billion in 2019), less than the EU27+2 average of 3.5%.

Using World Health Organization diagnostic criteria for osteoporosis based on the measurement of bone mineral density (BMD) [2], there were approximately 1,985,000 individuals with osteoporosis in Poland in 2019, of whom more than 80% were women. The prevalence of osteoporosis in the total Polish population amounted to 4.8%, somewhat lower than the EU27+2 average (5.6%).

**Table 1** Key measures of burden of disease for Poland

**Table:** 

**Category**	**Measure**	**Estimate**	**Rank**
Burden of disease	Direct cost of incident fracture (€m)	332.89	
	Long-term disability cost (€m)	347.32	
	Intervention cost (€m)	13.52	
	Total cost (€m)	693.73	
	QALYs lost (€m)	2172	
	Cost per capita (€)	18.27	28
	Proportion of healthcare spending	2.2%	25
	Prevalence of osteoporosis	4.8%	24

There were estimated to be 206,000 new fragility fractures in Poland in 2019, equivalent to 563 fractures/day (or 23 per hour). This was a slight increase compared to 2010, equivalent to an increment of 1.7 fractures/1000 individuals, totalling 14.3 fractures/ 1000 individuals in 2019.

Some osteoporotic fractures are associated with premature mortality [3]. In Poland, the annual number of deaths associated with a fracture event was estimated to be 113 per 100,000 individuals of the population aged 50 years or more, compared to the EU27+2 average of 116/100,000. The number of fracture-related deaths is comparable to or exceeds that of some of the most common causes of death such as lung cancer, diabetes, chronic lower respiratory diseases.

The remaining lifetime probability of hip fracture (%) at the ages of 50 years in men and women was 4.0% and 9.7%, respectively, Poland in the bottom tertile of risk for both men and women.

The population of men and women age 50 years or more is projected to increase by 16.6% between 2019 and 2034, somewhat higher than the EU27+2 average of 11.4%. The increases in men and women aged 75 years or more are even more marked and amount to 92.4% and 60.8%, respectively. The annual number of osteoporotic fractures in Poland is expected to increase by 61,000 to 267,000 in 2034.

**Policy framework** (Table 2)

Documentation of the burden of disease is an essential prerequisite to determine the resources that should be allocated to the diagnosis and treatment of the disorder. High quality national data on hip fracture rates have been identified in 18 of 29 countries, of which Poland belongs to the remaining 11 countries.

Given that osteoporosis and fragility fractures are common and that effective treatments are widely available, the vast majority of patients with osteoporosis are preferably managed at the primary health care level by general practitioners (GPs), with specialist referral reserved for difficult complex cases. Primary care was the principal provider of the medical care for osteoporosis in 13 of the 28 countries where data were available, and that was not the case for Poland.

Osteoporosis and metabolic bone disease is not a recognised specialty in most countries including Poland. Specialty care of osteoporosis in Poland is managed via other specialties including rheumatology, endocrinology, geriatrics, gynaecology, internal medicine and orthopaedics. Osteoporosis is however recognized as a component of specialty training. Although it is possible that these specialties educate their trainees adequately, the wide variation may reflect inconsistencies in patient care, training of primary care physicians and a suboptimal voice to “defend” the interests of those who work within the field of osteoporosis.

**Table 2** Policy framework for osteoporosis in Poland

**Table:** 

**Category**	**Measure**	**Estimate**
Policy framework	National fracture data availability	No
	OP recognized as a specialty	No
	OP primarily managed in primary care	No
	Other specialties involved	Rheumatology, Endocrinology, Geriatrics, Gynaecology, Internal medicine, Orthopaedics
	Advocacy areas covered by patient organisation	None

The role of national patient organisations is to improve the care of patients and increase awareness and prevention of osteoporosis and related fractures among the general public. Advocacy by patient organisations can fall into four categories: policy, capacity building and education, peer support, research and development. For Poland, none of the advocacy areas were covered. All four advocacy areas were covered for only 10 out of the 26 countries with at least one patient organisation.

**Service provision** (Table 3)

A wide variety of approved drug treatments is available for the management of osteoporosis [4]. Potential limitations of their use in member states relate to reimbursement policies which may impair the delivery of health care. Twelve out of 27 countries offered full reimbursement, which was not the case for Poland.

The assessment of bone mineral density forms a key component for the general management of osteoporosis, being used for diagnosis, risk prediction, selection of patients for treatment and monitoring of patients on treatment. In Poland, the number of DXA units expressed per million of the general population amounted to 7.1 which puts the country in 25^th^ place among the EU27+2.

The average waiting time for DXA ranged from 0 to 180 days across countries, and there was no clear relation between waiting times and the availability of DXA. In Poland, the estimated average waiting time for DXA amounted to 42 days. Twenty-one countries reported shorter average waiting times.

**Table 3** Service provision for osteoporosis in Poland

**Table:** 

**Category**	**Measure**	**Estimate**	**Rank**
Service provision	Reimbursement of OP medications	30%	
	DXA units/million inhabitants	7.1	25
	DXA cost (€)	22	24
	FRAX risk assessment model available	Yes	
	Fracture liaison service density	1-10%	

Reimbursement for DXA scans varied between member states both in terms of the criteria required and level of reimbursement awarded. In Poland, the reimbursement was conditional.

The effective targeting of treatment to those at highest risk of fracture requires an assessment of fracture risk. Risk assessment models for fractures, most usually based on FRAX, were available in 24 out of 29 countries, of which Poland was one. For Poland, guidance on the use of risk assessment within national guidelines was available, as in only 14 of the other countries.

Guidelines for the management of osteoporosis were available in Poland (as in 27 out of 29 countries). The guidelines in Poland included postmenopausal women specifically, as well as osteoporosis in men and secondary osteoporosis including glucocorticoid-induced osteoporosis.

Fracture liaison services (FLS), also known as osteoporosis coordinator programmes and care manager programmes, provide a system for the routine assessment and management of postmenopausal women and older men who have sustained a low trauma fracture. Fracture liaison services were reported for 1–10% of hospitals in Poland.

The use of indicators to systematically measure the quality of care provided to people with osteoporosis or associated fractures has expanded as a discipline within the past decade [5]. No use of national quality indicators was reported for Poland.

**Service uptake** (Table 4)

The web-based usage of FRAX showed considerable heterogeneity in uptake between the countries. The average uptake for the EU27+2 was 1,555 sessions/million/year of the general population with an enormous range of 49 to 41,874 sessions/million. The use of FRAX in Poland amounted to 513 sessions/million in 2019, with a 52 percent increase since 2011.

Many studies have demonstrated that a significant proportion of men and women at high fracture risk do not receive therapy for osteoporosis (the treatment gap) [6]. In the EU27+2 the average treatment gap was 71% but ranged from 32 to 87%. For Poland, the treatment gap amongst women amounted to 83% or 1,031,000 out of 1,236,000 characterised at risk. The Polish treatment gap did not change significantly compared to 2010, whilst the average treatment gap among EU27+2 increased from 55% in 2010 to 71% in 2019.

**Table 4** Service uptake for osteoporosis in Poland

**Table:** 

**Category**	**Measure**	**Estimate**	**Rank**
Service uptake	Number of FRAX sessions/million people/year	513	19
	Treatment gap for women eligible for treatment (%)	83	24
	Proportion surgically managed hip fractures	>90%	

About 5% of people with a hip fracture die within 1 month of their fracture [7]. A determinant of peri-operative morbidity and mortality is the time a patient takes to get to surgery [8]. For Poland, the average waiting time for hip fracture surgery after hospital admission was reported to be less than 24 h, implying a reduction in waiting time compared to 2010 (waiting time of 1–2 days). The proportion of surgically managed hip fractures was reported to be over 90%.

**Scores and scorecard**

Scores were developed for Burden of disease and the healthcare provision (Policy framework, Service provision and Service uptake) in the EU27+2 countries. Poland scores resulted in a 25th place regarding Burden of disease. The combined healthcare provision scorecard resulted in a 23rd place for Poland. Thus, Poland presents as one of the five low-burden low-provision countries among the EU27+2.

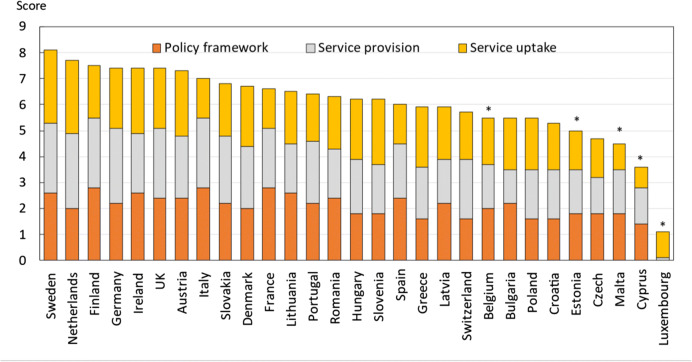



**Fig. 1** Scores by country for metrics related to policy framework, service provision and service uptake. The mean score for each of the 3 domains is given. An asterisk denotes that there was one or more missing metric which decreases the overall score

The first SCOPE was undertaken in 2010, almost 10 years previously. Fifteen of the 16 score card metrics on healthcare provision were used in the two surveys. Scores had improved or markedly improved in 15 countries, remained constant in 8 countries and worsened in 3 countries. For Poland, the scores were much improved.

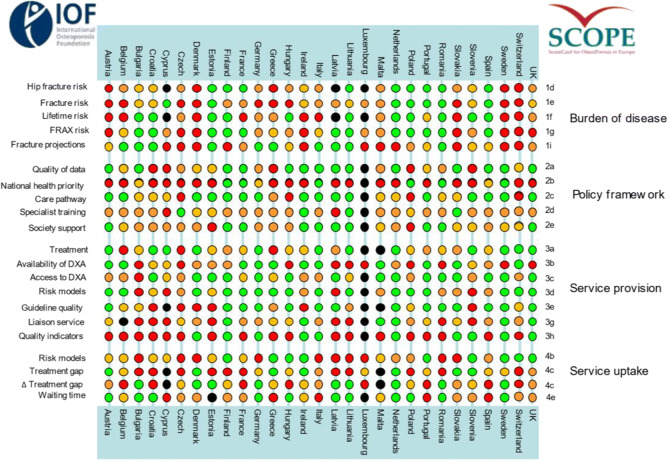



**Fig. 2** The scorecard for all the EU27+2 countries illustrating the scores across the four domains. The elements of each domain in each country were scored and coded using a traffic light system (red, orange, green). Black dots signify missing information

The second edition of the Scorecard for Osteoporosis in Europe (SCOPE 2021) allows health and policy professionals to assess key indicators on the healthcare provision for osteoporosis within countries and between countries within the EU 27+2. The scorecard is not intended as a prescriptive template. Thus, it does not set performance targets but may serve as a guide to the performance targets at which to aim in order to deliver the outcomes required.

**Acknowledgements**

SCOPE was supported by an unrestricted grant from Amgen to the International Osteoporosis Foundation (IOF). Amgen was neither involved in the design nor writing of the report. We are grateful to Anastasia Soulié Mlotek and Dominique Pierroz of the IOF for their help in the administration of SCOPE. The report has been reviewed by the members of the SCOPE Consultation Panel and the relevant IOF National societies, and we are grateful for their local insights on the management of osteoporosis in each country. The source document has been reviewed and endorsed by the Committee of Scientific Advisors of the IOF and benefitted from their feedback.

**References**

1. Kanis JA, Norton N, Harvey NC, Jacobson T, Johansson H, Lorentzon M, McCloskey EV, Willers C, Borgström F (2021) SCOPE 2021: a new scorecard for osteoporosis in Europe. Arch Osteoporos 16:82. 10.1007/s11657-020-00871-9

2. World Health Organisation (1994) Assessment of fracture risk and its application to screening for postmenopausal osteoporosis. Report of a WHO Study Group. World Health Organ Tech Rep Ser, 1994/01/01 edn, pp 1-129

3. Johnell O, Kanis JA, Oden A, Sernbo I, Redlund-Johnell I, Petterson C, De Laet C, Jonsson B (2004) Mortality after osteoporotic fractures. Osteoporos Int 15:38-42

4. Hernlund E, Svedbom A, Ivergard M, Compston J, Cooper C, Stenmark J, McCloskey EV, Jonsson B, Kanis JA (2013) Osteoporosis in the European Union: medical management, epidemiology and economic burden. A report prepared in collaboration with the International Osteoporosis Foundation (IOF) and the European Federation of Pharmaceutical Industry Associations (EFPIA). Arch Osteoporos 8:136

5. Allen P, Pilar M, Walsh-Bailey C, Hooley C, Mazzucca S, Lewis CC, Mettert KD, Dorsey CN, Purtle J, Kepper MM, Baumann AA, Brownson RC (2020) Quantitative measures of health policy implementation determinants and outcomes: a systematic review. Implement Sci 15:47

6. Borgstrom F, Karlsson L, Ortsater G, Norton N, Halbout P, Cooper C, Lorentzon M, McCloskey EV, Harvey NC, Javaid MK, Kanis JA (2020) Fragility fractures in Europe: burden, management and opportunities. Arch Osteoporos 15:59

7. Kanis JA, Oden A, Johnell O, De Laet C, Jonsson B, Oglesby AK (2003) The components of excess mortality after hip fracture. Bone 32:468-473

8. National Clinical Guideline Centre (2011) The Management of Hip Fracture in Adults. In Centre NCG (ed)London

## **Epidemiology and economic burden of osteoporosis in Portugal**

JAP Da Silva ∙ A Tirado ∙ AP Barbosa ∙ A Rodrigues ∙ A Gonçalves ∙ C Willers ∙ N Norton ∙ NC Harvey ∙ T Jacobson ∙ H Johansson ∙ M Lorentzon ∙ EV McCloskey ∙ F Borgström ∙ JA Kanis

**Introduction**

The scorecard summarises key indicators of the burden of osteoporosis and its management in the 27 member states of the European Union, as well as the UK and Switzerland (termed EU27+2) [1]. This country-specific report summarises the principal results for Portugal.

**Methods**

The information obtained covers four domains: burden of osteoporosis and fractures; policy framework; service provision; and service uptake. Data were collected from numerous sources including previous research and IOF reports, and available registers which were used for additional analysis of resource utilization, costing and HRQoL data. Furthermore, country-specific information on osteoporosis management was obtained from each IOF member state via a questionnaire.

**Burden of disease**

The direct cost of incident fractures in Portugal in 2019 was €523.9 million. Added to this was the ongoing cost in 2019 from fractures that occurred before 2019, which amounted to €464.8 million (long-term disability). The cost of pharmacological intervention (assessment and treatment) was €14.8 million. Thus, the total direct cost (excluding the value of QALYs lost) amounted to €1.0 billion in 2019. Key metrics are presented in Table 1.

In 2019, the average direct cost of osteoporotic fractures in Portugal was €97.6 per individual in the population, while in 2010 the average was €59.6 (after adjusting for inflation), representing an increase of 64% (€97.6 versus €59.6). The 2019 data rank Portugal in 10^th^ place in terms of highest cost of osteoporotic fractures per capita in the EU27+2.

The cost of osteoporotic fractures in Portugal accounted for approximately 5.6% of healthcare spending (i.e. €1.0 billion out of €17.6 billion in 2019), significantly higher than the EU27+2 average of 3.5% and ranked Portugal 4^th^ across the EU27+2 countries. These data indicate a substantial impact of fragility fractures on the healthcare budget.

Using World Health Organization diagnostic criteria for osteoporosis based on the measurement of bone mineral density (BMD) [2], there were approximately 681,000 individuals with osteoporosis in Portugal in 2019, of whom approximately 80% were women. The prevalence of osteoporosis in the total population amounted to 5.6%, on par with the EU27+2 average (5.6%).

**Table 1** Key measures of burden of disease for Portugal

**Table:** 

**Category**	**Measure**	**Estimate**	**Rank**
Burden of disease	Direct cost of incident fracture (€m)	523.86	
	Long-term disability cost (€m)	464.82	
	Intervention cost (€m)	14.82	
	Total cost (€m)	1003.51	
	QALYs lost (€m)	720	
	Cost per capita (€)	97.6	10
	Proportion of healthcare spending	5.6%	4
	Prevalence of osteoporosis	5.6%	10

There were estimated to be 70,700 new fragility fractures in Portugal in 2019, equivalent to 194 fractures/day (or 8 per hour). This was a slight increase compared to 2010, equivalent to an increment of 2.6 fractures/1000 individuals, totalling 15.8 fractures/ 1000 individuals in 2019.

Some osteoporotic fractures are associated with premature mortality [3]. In Portugal, the annual number of deaths associated with a fracture event was estimated to be 89 per 100,000 individuals of the population aged 50 years or more, compared to the EU27+2 average of 116/100,000. The number of fracture-related deaths is comparable to or exceeds that of some of the most common causes of death such as lung cancer, diabetes, chronic lower respiratory diseases.

The remaining lifetime probability of hip fracture (%) at the ages of 50 years in men and women was 4.8% and 14.4%, respectively, placing Portugal in the bottom tertile of risk for men and the mid tertile of risk for women.

The population of men and women age 50 years or more is projected to increase by 11.3% between 2019 and 2034, close to the EU27+2 average of 11.4%. The increases in men and women aged 75 years or more are even more marked and amount to 33.9% and 26.1%, respectively. The annual number of osteoporotic fractures in Portugal is expected to increase by 20,500 to 91,200 in 2034.

**Policy framework** (Table 2)

Documentation of the burden of disease is an essential prerequisite to determine the resources that should be allocated to the diagnosis and treatment of the disorder. High quality national data on hip fracture rates have been identified in 18 of 29 countries, of which Portugal is one. Data are collected on a national basis and include more than only hip fracture data.

Given that osteoporosis and fragility fractures are common and that effective treatments are widely available, the vast majority of patients with osteoporosis are preferably managed at the primary health care level by general practitioners (GPs), with specialist referral reserved for difficult complex cases. Primary care was the principal provider of the medical care for osteoporosis in Portugal, as for 13 of the 28 countries where data were available.

Osteoporosis and metabolic bone disease is not a recognised specialty in most countries including Portugal. Specialty care of osteoporosis in Portugal is managed via other specialties including general practice, rheumatology internal medicine, orthopaedics, gynaecology, endocrinology, and rehabilitation medicine. Osteoporosis is however recognized as a component of specialty training. Although it is possible that these specialties educate their trainees adequately, the wide variation may reflect inconsistencies in patient care, training of primary care physicians and a suboptimal voice to “defend” the interests of those who work within the field of osteoporosis.

**Table 2** Policy framework for osteoporosis in Portugal

**Table:** 

**Category**	**Measure**	**Estimate**
Policy framework	National fracture data availability	No
	OP recognized as a specialty	No
	OP primarily managed in primary care	Yes
	Other specialties involved	Rheumatology, Internal medicine, Orthopaedics, Gynaecology, Endocrinology, Rehabilitation medicine,
	Advocacy areas covered by patient organisation	Policy, capacity, peer support, research and development

The role of national patient organisations is to improve the care of patients and increase awareness and prevention of osteoporosis and related fractures among the general public. Advocacy by patient organisations can fall into four categories: policy, capacity building and education, peer support, research and development. For Portugal, all four of the advocacy areas were covered by a patient organisation, which was the case for only 10 out of the 26 countries with at least one patient organisation.

**Service provision** (Table 3)

A wide variety of approved drug treatments is available for the management of osteoporosis [4]. Potential limitations of their use in member states relate to reimbursement policies which may impair the delivery of health care. Twelve out of 27 countries offered full reimbursement, and that was not the case for Portugal.

The assessment of bone mineral density forms a key component for the general management of osteoporosis, being used for diagnosis, risk prediction, selection of patients for treatment and monitoring of patients on treatment. In Portugal, the number of DXA units expressed per million of the general population amounted to 25.4 which puts the country in 6th place among the EU27+2.

The average waiting time for DXA ranged from 0 to 180 days across countries, and there was no clear relation between waiting times and the availability of DXA. In Portugal, the estimated average waiting time for DXA amounted to 7 days. Four countries reported shorter average waiting times.

**Table 3** Service provision for osteoporosis in Portugal

**Table:** 

**Category**	**Measure**	**Estimate**	**Rank**
Service provision	Reimbursement of OP medications	69%	
	DXA units/million inhabitants	25.4	6
	DXA cost (€)	35	18
	FRAX risk assessment model available	Yes	
	Fracture liaison service density	1-10%	

Reimbursement for DXA scans varied between member states both in terms of the criteria required and level of reimbursement awarded. In Portugal, the reimbursement was unconditional.

The effective targeting of treatment to those at highest risk of fracture requires an assessment of fracture risk. Risk assessment models for fractures, most usually based on FRAX, were available in 24 out of 29 countries, of which Portugal was one. For Portugal, guidance on the use of risk assessment within national guidelines was available, as in only 14 of the other countries.

Guidelines for the management of osteoporosis were available in Portugal (as in 27 out of 29 countries). The guidelines in Portugal included postmenopausal women specifically, as well as osteoporosis in men and secondary osteoporosis including glucocorticoid-induced osteoporosis.

Fracture liaison services (FLS), also known as osteoporosis coordinator programmes and care manager programmes, provide a system for the routine assessment and management of postmenopausal women and older men who have sustained a low trauma fracture. Fracture liaison services were reported for 1–10% of hospitals in Portugal.

The use of indicators to systematically measure the quality of care provided to people with osteoporosis or associated fractures has expanded as a discipline within the past decade [5]. No use of national quality indicators was reported for Portugal.

**Service uptake** (Table 4)

The web-based usage of FRAX showed considerable heterogeneity in uptake between the countries. The average uptake for the EU27+2 was 1,555 sessions/million/year of the general population with an enormous range of 49 to 41,874 sessions/million. The use of FRAX in Portugal amounted to 2,662 sessions/million in 2019, with a 156 percent increase since 2011.

Many studies have demonstrated that a significant proportion of men and women at high fracture risk do not receive therapy for osteoporosis (the treatment gap) [6]. In the EU27+2 the average treatment gap was 71% but ranged from 32 to 87%. For Portugal, the treatment gap amongst women amounted to 75% or 356,000 out of 474,000 characterised at risk and it increased significantly compared to 2010. The average treatment gap among EU27+2 increased from 55% in 2010 to 71% in 2019.

**Table 4** Service uptake for osteoporosis in Portugal

**Table:** 

**Category**	**Measure**	**Estimate**	**Rank**
Service uptake	Number of FRAX sessions/million people/year	2662	8
	Treatment gap for women eligible for treatment (%)	75	15
	Proportion surgically managed hip fractures	>90%	

About 5% of people with a hip fracture die within 1 month of their fracture [7]. A determinant of peri-operative morbidity and mortality is the time a patient takes to get to surgery [8]. For Portugal, the average waiting time for hip fracture surgery after hospital admission was reported to be more than 3 days, implying an increase in waiting time compared to 2010 (waiting time of 2–3 days). The proportion of surgically managed hip fractures was reported to be over 90%.

**Scores and scorecard**

Scores were developed for Burden of disease and the healthcare provision (Policy framework, Service provision and Service uptake) in the EU27+2 countries. Portugal scores resulted in a 21st place regarding Burden of disease. The combined healthcare provision scorecard resulted in a 13th place for Portugal. Thus, Portugal presents as one of the low-burden high-provision countries among the EU27+2.

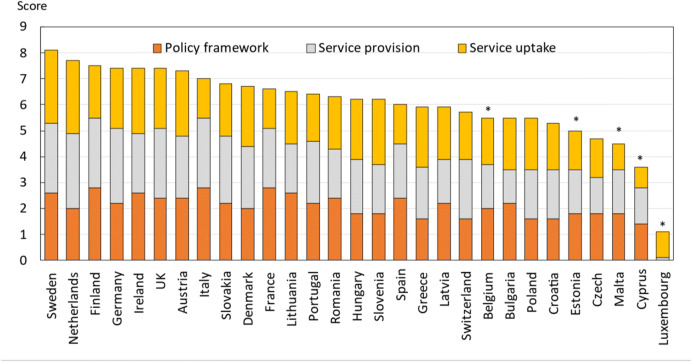



**Fig. 1** Scores by country for metrics related to policy framework, service provision and service uptake. The mean score for each of the 3 domains is given. An asterisk denotes that there was one or more missing metric which decreases the overall score

The first SCOPE was undertaken in 2010, almost 10 years previously. Fifteen of the 16 score card metrics on healthcare provision were used in the two surveys. Scores had improved or markedly improved in 15 countries, remained constant in 8 countries and worsened in 3 countries. For Portugal the scores were almost unchanged.

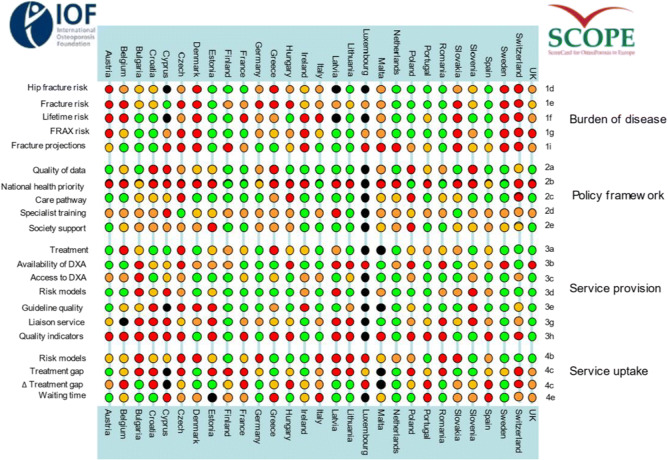



**Fig. 2** The scorecard for all the EU27+2 countries illustrating the scores across the four domains. The elements of each domain in each country were scored and coded using a traffic light system (red, orange, green). Black dots signify missing information

The second edition of the Scorecard for Osteoporosis in Europe (SCOPE 2021) allows health and policy professionals to assess key indicators on the healthcare provision for osteoporosis within countries and between countries within the EU 27+2. The scorecard is not intended as a prescriptive template. Thus, it does not set performance targets but may serve as a guide to the performance targets at which to aim in order to deliver the outcomes required.

**Acknowledgements**

SCOPE was supported by an unrestricted grant from Amgen to the International Osteoporosis Foundation (IOF). Amgen was neither involved in the design nor writing of the report. We are grateful to Anastasia Soulié Mlotek and Dominique Pierroz of the IOF for their help in the administration of SCOPE. The report has been reviewed by the members of the SCOPE Consultation Panel and the relevant IOF National societies, and we are grateful for their local insights on the management of osteoporosis in each country. The source document has been reviewed and endorsed by the Committee of Scientific Advisors of the IOF and benefitted from their feedback.

**References**

1. Kanis JA, Norton N, Harvey NC, Jacobson T, Johansson H, Lorentzon M, McCloskey EV, Willers C, Borgström F (2021) SCOPE 2021: a new scorecard for osteoporosis in Europe. Arch Osteoporos 16:82. 10.1007/s11657-020-00871-9

2. World Health Organisation (1994) Assessment of fracture risk and its application to screening for postmenopausal osteoporosis. Report of a WHO Study Group. World Health Organ Tech Rep Ser, 1994/01/01 edn, pp 1-129

3. Johnell O, Kanis JA, Oden A, Sernbo I, Redlund-Johnell I, Petterson C, De Laet C, Jonsson B (2004) Mortality after osteoporotic fractures. Osteoporos Int 15:38-42

4. Hernlund E, Svedbom A, Ivergard M, Compston J, Cooper C, Stenmark J, McCloskey EV, Jonsson B, Kanis JA (2013) Osteoporosis in the European Union: medical management, epidemiology and economic burden. A report prepared in collaboration with the International Osteoporosis Foundation (IOF) and the European Federation of Pharmaceutical Industry Associations (EFPIA). Arch Osteoporos 8:136

5. Allen P, Pilar M, Walsh-Bailey C, Hooley C, Mazzucca S, Lewis CC, Mettert KD, Dorsey CN, Purtle J, Kepper MM, Baumann AA, Brownson RC (2020) Quantitative measures of health policy implementation determinants and outcomes: a systematic review. Implement Sci 15:47

6. Borgstrom F, Karlsson L, Ortsater G, Norton N, Halbout P, Cooper C, Lorentzon M, McCloskey EV, Harvey NC, Javaid MK, Kanis JA (2020) Fragility fractures in Europe: burden, management and opportunities. Arch Osteoporos 15:59

7. Kanis JA, Oden A, Johnell O, De Laet C, Jonsson B, Oglesby AK (2003) The components of excess mortality after hip fracture. Bone 32:468-473

8. National Clinical Guideline Centre (2011) The Management of Hip Fracture in Adults. In Centre NCG (ed)London

## **Epidemiology and economic burden of osteoporosis in Romania**

D Grigorie ∙ AI Gasparik ∙ I Pascanu ∙ D Grigorie ∙ C Willers ∙ N Norton ∙ NC Harvey ∙ T Jacobson ∙ H Johansson ∙ M Lorentzon ∙ EV McCloskey ∙ F Borgström ∙ JA Kanis

**Introduction**

The scorecard summarises key indicators of the burden of osteoporosis and its management in the 27 member states of the European Union, as well as the UK and Switzerland (termed EU27+2) [1]. This country-specific report summarises the principal results for Romania.

**Methods**

The information obtained covers four domains: burden of osteoporosis and fractures; policy framework; service provision; and service uptake. Data were collected from numerous sources including previous research and IOF reports, and available registers which were used for additional analysis of resource utilization, costing and HRQoL data. Furthermore, country-specific information on osteoporosis management was obtained from each IOF member state via a questionnaire.

**Burden of disease**

The direct cost of incident fractures in Romania in 2019 was €91.0 million. Added to this was the ongoing cost in 2019 from fractures that occurred before 2019, which amounted to €150.1 million (long-term disability). The cost of pharmacological intervention (assessment and treatment) was €16.2 million. Thus, the total direct cost (excluding the value of QALYs lost) amounted to €257.3 million in 2019. Key metrics are presented in Table 1.

In 2019, the average direct cost of osteoporotic fractures in Romania was €13.2 per individual in the population, while in 2010 the average was €6.6 (after adjusting for inflation), representing an increase of 100% (€13.2 versus €6.6). The 2019 data place Romania lowest (29th) in terms of the cost of osteoporotic fractures per capita in the EU27+2.

The cost of osteoporotic fractures in Romania accounted for approximately 2.5% of healthcare spending (i.e. €257 million out of €9.7 billion in 2019), which is lower than the EU27+2 average of 3.5%.

Using World Health Organization diagnostic criteria for osteoporosis based on the measurement of bone mineral density (BMD) [2], there were approximately 1,071,000 individuals with osteoporosis in Romania in 2019, of whom approximately 81% were women. The prevalence of osteoporosis in the total Romanian population amounted to 4.8%, somewhat lower than the EU27+2 average (5.6%).

**Table 1** Key measures of burden of disease for Romania

**Table:** 

**Category**	**Measure**	**Estimate**	**Rank**
Burden of disease	Direct cost of incident fracture (€m)	91.02	
	Long-term disability cost (€m)	150.13	
	Intervention cost (€m)	16.17	
	Total cost (€m)	257.32	
	QALYs lost (€m)	1035	
	Cost per capita (€)	13.21	29
	Proportion of healthcare spending	2.5%	22
	Prevalence of osteoporosis	4.8%	25

There were estimated to be 103,000 new fragility fractures in Romania in 2019, equivalent to 282 fractures/day (or almost 12 per hour). This was a slight increase compared to 2010, equivalent to an increment of 0.7 fractures/1000 individuals, totalling 13.6 fractures/ 1000 individuals in 2019.

Some osteoporotic fractures are associated with premature mortality [3]. In Romania, the annual number of deaths associated with a fracture event was estimated to be 148 per 100,000 individuals of the population aged 50 years or more, compared to the EU27+2 average of 116/100,000. The number of fracture-related deaths is comparable to or exceeds that for some of the most common causes of death such as lung cancer, diabetes, chronic lower respiratory diseases.

The remaining lifetime probability of hip fracture (%) at the ages of 50 years in men and women was 3.8% and 7.0%, respectively, placing Romania in the bottom tertile of risk for both men and women.

The population in men and women age 50 years or more is projected to increase by 6.4% between 2019 and 2034, which is lower than the EU27+2 average of 11.4%. The increases in men and women aged 75 years or more are more marked and amount to 38.5% and 29.6%, respectively. The annual number of osteoporotic fractures in Romania is expected to increase by 15,000 to 118,000 in 2034.

**Policy framework** (Table 2)

Documentation of the burden of disease is an essential prerequisite to determine the resources that should be allocated to the diagnosis and treatment of the disorder. High quality national data on hip fracture rates have been identified in 18 of 29 countries, of which Romania is one. Data are collected on a national basis and include more than only hip fracture data [4].

Given that osteoporosis and fragility fractures are common and that effective treatments are widely available, the vast majority of patients with osteoporosis are preferably managed at the primary health care level by general practitioners (GPs), with specialist referral reserved for difficult complex cases. Primary care was the principal provider of the medical care for osteoporosis in 13 of the 28 countries where data were available, but this was not the case in Romania. Here, osteoporosis care was primarily devolved to endocrinologists. Specialty care of osteoporosis in Romania is managed also via other specialties including rheumatology and rehabilitation medicine.

Osteoporosis and metabolic bone disease is not a recognised specialty in most countries including Romania. Osteoporosis is however recognized as a component of specialty training. Although it is possible that these specialties educate their trainees adequately, the wide variation may reflect inconsistencies in patient care, training of primary care physicians and a suboptimal voice to “defend” the interests of those who work within the field of osteoporosis.

**Table 2** Policy framework for osteoporosis in Romania

**Table:** 

**Category**	**Measure**	**Estimate**
Policy framework	National fracture data availability	Yes
	OP recognized as a specialty	No
	OP primarily managed in primary care	No
	Other specialties involved	Endocrinology, Rheumatology, Rehabilitation medicine
	Advocacy areas covered by patient organisation	Policy, capacity, peer support, research and development

The role of national patient organisations is to improve the care of patients and increase awareness and prevention of osteoporosis and related fractures among the general public. Advocacy by patient organisations can fall into four categories: policy, capacity building and education, peer support, research and development. The Romanian Society of Osteoporosis and Musculoskeletal Diseases (SROBMS) covers all four of the advocacy areas, which was the case for only 10 out of the 26 countries with at least one patient organisation.

**Service provision** (Table 3)

A wide variety of approved drug treatments is available for the management of osteoporosis [5]. Potential limitations of their use in member states relate to reimbursement policies which may impair the delivery of health care. Twelve out of 27 countries offered full reimbursement, but this was not the case for Romania.

The assessment of bone mineral density forms a key component for the general management of osteoporosis, being used for diagnosis, risk prediction, selection of patients for treatment and monitoring of patients on treatment. In Romania, the number of DXA units expressed per million of the general population amounted to 9.9 which puts the country in 20th place among the EU27+2.

The average waiting time for DXA ranged from 0 to 180 days across countries, and there was no clear relation between waiting times and the availability of DXA. In Romania, the waiting time for DXA was 0 days if the test was paid by the patient and 2–4 weeks when using the reimbursement budget.

**Table 3** Service provision for osteoporosis in Romania

**Table:** 

**Category**	**Measure**	**Estimate**	**Rank**
Service provision	Reimbursement of OP medications	50-100%	
	DXA units/million inhabitants	9.9	20
	DXA cost (€)	10-50	
	FRAX risk assessment model available	Yes	
	Fracture liaison service density	No FLS	

Reimbursement for DXA scans varied between member states both in terms of the criteria required and level of reimbursement awarded. In Romania, the reimbursement was unconditional.

The effective targeting of treatment to those at highest risk of fracture requires an assessment of fracture risk. Risk assessment models for fractures, most usually based on FRAX, were available in 24 out of 29 countries, of which Romania was one [6]. For Romania, guidance on the use of risk assessment within national guidelines was available, as in only 14 of the other countries.

Guidelines for the management of osteoporosis were available in Romania (as in 27 out of 29 countries). The guidelines in Romania included postmenopausal women specifically, as well as osteoporosis in men and secondary osteoporosis including glucocorticoid-induced osteoporosis but has not been fully updated since 2010.

Fracture liaison services (FLS), also known as osteoporosis coordinator programmes and care manager programmes, provide a system for the routine assessment and management of postmenopausal women and older men who have sustained a low trauma fracture. No fracture liaison services were reported from Romania (together with 7 other countries).

The use of indicators to systematically measure the quality of care provided to people with osteoporosis or associated fractures has expanded as a discipline within the past decade [7]. No use of national quality indicators was reported for Romania.

**Service uptake** (Table 4)

The web-based usage of FRAX showed considerable heterogeneity in uptake between the countries. The average uptake for the EU27+2 2019 was 1,555 sessions/million of the general population with an enormous range of 49 to 41,874 sessions/million. The usage for Romania amounted to 463 sessions/million in 2019, with a 101 percent increase since 2011.

Many studies have demonstrated that a significant proportion of men and women at high fracture risk do not receive therapy for osteoporosis (the treatment gap) [8]. In the EU27+2 the average treatment gap was 71% but ranged from 32 to 87%. For Romania, the treatment gap amongst women amounted to 78% or 469,000 out of 599,000 characterised at risk. The Romanian treatment gap did not change significantly compared to 2010, whilst the average treatment gap among EU27+2 increased from 55% in 2010 to 71% in 2019.

**Table 4** Service uptake for osteoporosis in Romania

**Table:** 

**Category**	**Measure**	**Estimate**	**Rank**
Service uptake	Number of FRAX sessions/million people/year	463	22
	Treatment gap for women eligible for treatment (%)	78	18
	Proportion surgically managed hip fractures	N/A	

About 5% of people with a hip fracture die within 1 month of their fracture [9]. A determinant of peri-operative morbidity and mortality is the time a patient takes to get to surgery [10]. For Romania, the average waiting time for hip fracture surgery after hospital admission was reported to be less than 24 h, implying a reduction in waiting time compared to 2010 (waiting time of 1–2 days). No information was available regarding the proportion of surgically managed hip fractures. Subsequent information indicates that 81% of hip fractures are surgically managed [4].

**Scores and scorecard**

Scores were developed for Burden of disease and the healthcare provision (Policy framework, Service provision and Service uptake) in the EU27+2 countries. Romania scores resulted in a last (29th) place regarding Burden of disease. The combined healthcare provision scorecard resulted in a 14th place for Romania. Thus, Romania presents as one of the low-burden high-provision countries among the EU27+2.

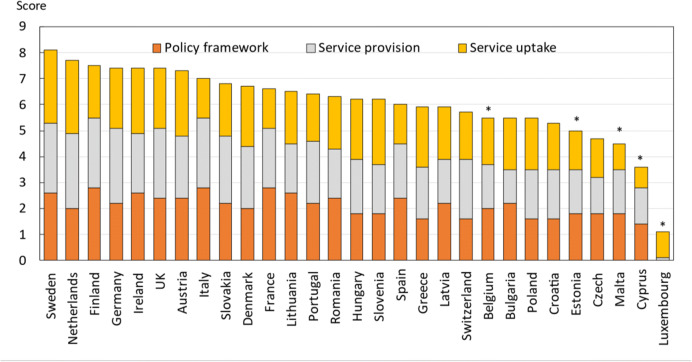



**Fig. 1** Scores by country for metrics related to policy framework, service provision and service uptake. The mean score for each of the 3 domains is given. An asterisk denotes that there was one or more missing metric which decreases the overall score

The first SCOPE was undertaken in 2010, almost 10 years previously. Fifteen of the 16 score card metrics on healthcare provision were used in the two surveys. Scores had improved or markedly improved in 15 countries, remained constant in 8 countries and worsened in 3 countries. For Romania, the scores were much improved.

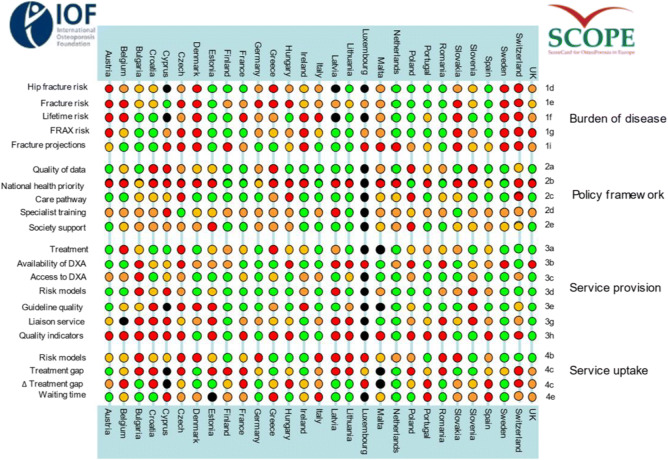



**Fig. 2** The scorecard for all the EU27+2 countries illustrating the scores across the four domains. The elements of each domain in each country were scored and coded using a traffic light system (red, orange, green). Black dots signify missing information

The second edition of the Scorecard for Osteoporosis in Europe (SCOPE 2021) allows health and policy professionals to assess key indicators on the healthcare provision for osteoporosis within countries and between countries within the EU 27+2. The scorecard is not intended as a prescriptive template. Thus, it does not set performance targets but may serve as a guide to the performance targets at which to aim in order to deliver the outcomes required.

**Acknowledgements**

SCOPE was supported by an unrestricted grant from Amgen to the International Osteoporosis Foundation (IOF). Amgen was neither involved in the design nor writing of the report. We are grateful to Anastasia Soulié Mlotek and Dominique Pierroz of the IOF for their help in the administration of SCOPE. The report has been reviewed by the members of the SCOPE Consultation Panel and the relevant IOF National societies, and we are grateful for their local insights on the management of osteoporosis in each country. The source document has been reviewed and endorsed by the Committee of Scientific Advisors of the IOF and benefitted from their feedback.

**References**

1. Kanis JA, Norton N, Harvey NC, Jacobson T, Johansson H, Lorentzon M, McCloskey EV, Willers C, Borgström F (2021) SCOPE 2021: a new scorecard for osteoporosis in Europe. Arch Osteoporos 16:82. 10.1007/s11657-020-00871-9

2. World Health Organisation (1994) Assessment of fracture risk and its application to screening for postmenopausal osteoporosis. Report of a WHO Study Group. World Health Organ Tech Rep Ser, 1994/01/01 edn, pp 1-129

3. Johnell O, Kanis JA, Oden A, Sernbo I, Redlund-Johnell I, Petterson C, De Laet C, Jonsson B (2004) Mortality after osteoporotic fractures. Osteoporos Int 15:38-42

4. Grigorie D, Sucaliuc A, Ciutan M, Vladescu C (2019) incidence and time trend of hip fractures in Romania: a nationwide study from 2008 to 2018. Acta Endocrinol (Buchar) - 15: 505-512.

5. Hernlund E, Svedbom A, Ivergard M, Compston J, Cooper C, Stenmark J, McCloskey EV, Jonsson B, Kanis JA (2013) Osteoporosis in the European Union: medical management, epidemiology and economic burden. A report prepared in collaboration with the International Osteoporosis Foundation (IOF) and the European Federation of Pharmaceutical Industry Associations (EFPIA). Arch Osteoporos 8:136

6. Grigorie D, Sucaliuc A, Johansson H, Kanis JA, McCloskey E (2013) FRAX-based intervention and assessment thresholds for osteoporosis in Romania. Arch Osteoporos 8:164. doi: 10.1007/s11657-013-0164-x.

7. Allen P, Pilar M, Walsh-Bailey C, Hooley C, Mazzucca S, Lewis CC, Mettert KD, Dorsey CN, Purtle J, Kepper MM, Baumann AA, Brownson RC (2020) Quantitative measures of health policy implementation determinants and outcomes: a systematic review. Implement Sci 15:47

8. Borgstrom F, Karlsson L, Ortsater G, Norton N, Halbout P, Cooper C, Lorentzon M, McCloskey EV, Harvey NC, Javaid MK, Kanis JA (2020) Fragility fractures in Europe: burden, management and opportunities. Arch Osteoporos 15:59

9. Kanis JA, Oden A, Johnell O, De Laet C, Jonsson B, Oglesby AK (2003) The components of excess mortality after hip fracture. Bone 32:468-473

10. National Clinical Guideline Centre (2011) The Management of Hip Fracture in Adults. In Centre NCG (ed)London

## **Epidemiology and economic burden of osteoporosis in Slovakia**

J Payer ∙ P Masaryk ∙ P Jackuliak ∙ C Willers ∙ N Norton ∙ NC Harvey ∙ T Jacobson ∙ H Johansson ∙ M Lorentzon ∙ EV McCloskey ∙ F Borgström ∙ JA Kanis

**Introduction**

The scorecard summarises key indicators of the burden of osteoporosis and its management in the 27 member states of the European Union, as well as the UK and Switzerland (termed EU27+2) [1]. This country-specific report summarises the principal results for Slovakia.

**Methods**

The information obtained covers four domains: burden of osteoporosis and fractures; policy framework; service provision; and service uptake. Data were collected from numerous sources including previous research and IOF reports, and available registers which were used for additional analysis of resource utilization, costing and HRQoL data. Furthermore, country-specific information on osteoporosis management was obtained from each IOF member state via a questionnaire.

**Burden of disease**

The direct cost of incident fractures in Slovakia in 2019 was €135.2 million. Added to this was the ongoing cost in 2019 from fractures that occurred before 2019, which amounted to €41.7 million (long-term disability). The cost of pharmacological intervention (assessment and treatment) was €16.7 million. Thus, the total direct cost (excluding the value of QALYs lost) amounted to €193.7 million in 2019. Key metrics are presented in Table 1.

In 2019, the average direct cost of osteoporotic fractures in Slovakia was €35.6 per individual in the population, while in 2010 the average was €22.1 (after adjusting for inflation), representing an increase of 61% (€35.6 versus €22.1). The 2019 data rank Slovakia in 22^nd^ place in terms of highest cost of osteoporotic fractures per capita in the EU27+2.

The cost of osteoporotic fractures in Slovakia accounted for approximately 3.1% of healthcare spending (i.e. €194 million out of €5.8 billion in 2019), close to the EU27+2 average of 3.5%. These numbers indicate a substantial impact of fragility fractures on the healthcare budget.

Using World Health Organization diagnostic criteria for osteoporosis based on the measurement of bone mineral density (BMD) [2], there were approximately 264,000 individuals with osteoporosis in Slovakia in 2019, of whom approximately 81% were women. The prevalence of osteoporosis in the total Slovakian population amounted to 4.2%, somewhat lower than the EU27+2 average (5.6%).

**Table 1** Key measures of burden of disease for Slovakia

**Table:** 

**Category**	**Measure**	**Estimate**	**Rank**
Burden of disease	Direct cost of incident fracture (€m)	135.24	
	Long-term disability cost (€m)	41.73	
	Intervention cost (€m)	16.68	
	Total cost (€m)	193.66	
	QALYs lost (€m)	724	
	Cost per capita (€)	35.55	22
	Proportion of healthcare spending	3.1%	15
	Prevalence of osteoporosis	4.2%	27

There were estimated to be 76,000 new fragility fractures in Slovakia in 2019, equivalent to 207 fractures/day (or almost 9 per hour). This was a significant increase compared to 2010, equivalent to an increased population risk of incrementally 15.9 fractures/1000 individuals, totalling 38.2 fractures/ 1000 individuals in 2019.

Some osteoporotic fractures are associated with premature mortality [3]. In Slovakia, the annual number of deaths associated with a fracture event was high (216 per 100,000 individuals of the population aged 50 years or more). The number of fracture-related deaths is comparable to or exceeds that for some of the most common causes of death such as lung cancer, diabetes, chronic lower respiratory diseases.

The remaining lifetime probability of hip fracture (%) at the ages of 50 years in men and women was 9.5% and 20.3%, respectively, placing Slovakia in the upper tertile of risk for both men and women.

The population of men and women age 50 years or more is projected to increase by 20.2% between 2019 and 2034, significantly above the EU27+2 average of 11.4%. The increases in men and women aged 75 years or more are even more marked and amount to 88.8% and 58.8%, respectively. The annual number of osteoporotic fractures in Slovakia is expected to increase by 25,000 to 101,000 in 2034.

**Policy framework** (Table 2)

Documentation of the burden of disease is an essential prerequisite to determine the resources that should be allocated to the diagnosis and treatment of the disorder. High quality national data on hip fracture rates have been identified in 18 of 29 countries, of which Slovakia is one. Data are collected on a national basis and include hip fracture data.

Given that osteoporosis and fragility fractures are common and that effective treatments are widely available, the vast majority of patients with osteoporosis are preferably managed at the primary health care level by general practitioners (GPs), with specialist referral reserved for difficult complex cases. Primary care was the principal provider of the medical care for osteoporosis in 13 of the 28 countries where data were available, but this was not the case for Slovakia.

Osteoporosis and metabolic bone disease is not a recognised specialty in most countries but this is the case in Slovakia. Specialty care of osteoporosis in Slovakia is managed also via other specialties including rheumatology, orthopaedics and endocrinology, and osteoporosis is recognized as a component of specialty training. In addition, there is a faculty-based training course in place for formal certification in clinical osteology. Although it is possible that these specialties educate their trainees adequately, the wide variation may reflect inconsistencies in patient care, training of primary care physicians and a suboptimal voice to “defend” the interests of those who work within the field of osteoporosis.

**Table 2** Policy framework for osteoporosis in Slovakia

**Table:** 

**Category**	**Measure**	**Estimate**
Policy framework	National fracture data availability	Yes
	OP recognized as a specialty	Yes
	OP primarily managed in primary care	No
	Other specialties involved	Rheumatology, Orthopaedics, Endocrinology
	Advocacy areas covered by patient organisation	Policy, capacity, peer support

The role of national patient organisations is to improve the care of patients and increase awareness and prevention of osteoporosis and related fractures among the general public. Advocacy by patient organisations can fall into four categories: policy, capacity building and education, peer support, research and development. For Slovakia, three of these were covered by a patient organisation. All four advocacy areas were covered for only 10 out of the 26 countries with at least one patient organisation.

**Service provision** (Table 3)

A wide variety of approved drug treatments is available for the management of osteoporosis [4]. Potential limitations of their use in member states relate to reimbursement policies which may impair the delivery of health care. Twelve out of 27 countries offered full reimbursement, but Slovakia was not one, though reimbursement is more than 90%.

The assessment of bone mineral density forms a key component for the general management of osteoporosis, being used for diagnosis, risk prediction, selection of patients for treatment and monitoring of patients on treatment. In Slovakia, the number of DXA units expressed per million of the general population amounted to 30.2 which puts the country in 2nd place among the EU27+2.

The average waiting time for DXA ranged from 0 to 180 days across countries, and there was no clear relation between waiting times and the availability of DXA. In Slovakia, the estimated average waiting time for DXA amounted to 18 days. Fifteen countries reported shorter average waiting times.

**Table 3** Service provision for osteoporosis in Slovakia

**Table:** 

**Category**	**Measure**	**Estimate**	**Rank**
Service provision	Reimbursement of OP medications	90%	
	DXA units/million inhabitants	30.2	2
	DXA cost (€)	32	19
	FRAX risk assessment model available	Yes	
	Fracture liaison service density	1-10%	

Reimbursement for DXA scans varied between member states both in terms of the criteria required and level of reimbursement awarded. In Slovakia, the reimbursement was unconditional.

The effective targeting of treatment to those at highest risk of fracture requires an assessment of fracture risk. Risk assessment models for fractures, most usually based on FRAX, were available in 24 out of 29 countries, of which Slovakia was one. For Slovakia, guidance on the use of risk assessment within national guidelines was available, as in only 14 of the other countries.

Guidelines for the management of osteoporosis were available in Slovakia (as in 27 out of 29 countries). The guidelines in Slovakia included postmenopausal women specifically, as well as osteoporosis in men and secondary osteoporosis including glucocorticoid-induced osteoporosis.

Fracture liaison services (FLS), also known as osteoporosis coordinator programmes and care manager programmes, provide a system for the routine assessment and management of postmenopausal women and older men who have sustained a low trauma fracture. Fracture liaison services were reported for 1-10% of hospitals in Slovakia.

The use of indicators to systematically measure the quality of care provided to people with osteoporosis or associated fractures has expanded as a discipline within the past decade [5]. Slovakia was one of few countries with national quality indicators in place.

**Service uptake** (Table 4)

The web-based usage of FRAX showed considerable heterogeneity in uptake between the countries. The average uptake for the EU27+2 in 2019 was 1,555 sessions/million of the general population with an enormous range of 49 to 41,874 sessions/million. The use of FRAX in Slovakia amounted to 504 sessions/million in 2019, with a 35% increase since 2011.

Many studies have demonstrated that a significant proportion of men and women at high fracture risk do not receive therapy for osteoporosis (the treatment gap) [6]. In the EU27+2 the average treatment gap was 71% but ranged from 32 to 87%. For Slovakia, the treatment gap amongst women amounted to 54% or 90,000 out of 165,000 characterised at risk. The Slovakian treatment gap did not change significantly compared to 2010, whilst the average treatment gap among EU27+2 increased from 55% in 2010 to 71% in 2019.

**Table 4** Service uptake for osteoporosis in Slovakia

**Table:** 

**Category**	**Measure**	**Estimate**	**Rank**
Service uptake	Number of FRAX sessions/million people/year	504	21
	Treatment gap for women eligible for treatment (%)	54	5
	Proportion surgically managed hip fractures	>90%	

About 5% of people with a hip fracture die within 1 month of their fracture [7]. A determinant of peri-operative morbidity and mortality is the time a patient takes to get to surgery [8]. For Slovakia, the average waiting time for hip fracture surgery after hospital admission was reported to be 1–2 days. The proportion of surgically managed hip fractures was reported to be over 90%.

**Scores and scorecard**

Scores were developed for Burden of disease and the healthcare provision (Policy framework, Service provision and Service uptake) in the EU27+2 countries. Slovakia scores resulted in a 5th place regarding Burden of disease after only Denmark, Sweden, Switzerland and Austria. The combined healthcare provision scorecard resulted in a 9th place for Slovakia. Thus, Slovakia presents as one of the high-burden high-provision countries among the EU27+2.

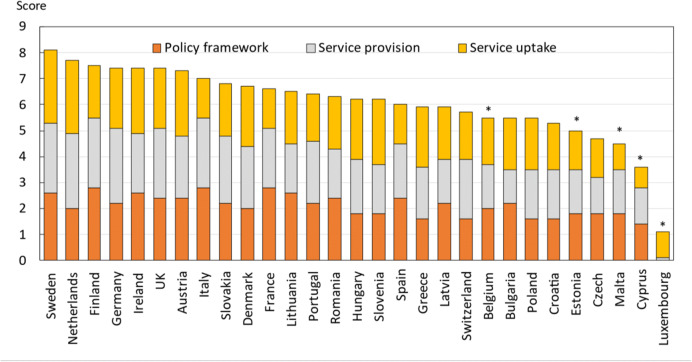



**Fig. 1** Scores by country for metrics related to policy framework, service provision and service uptake. The mean score for each of the 3 domains is given. An asterisk denotes that there was one or more missing metric which decreases the overall score

The first SCOPE was undertaken in 2010, almost 10 years previously. Fifteen of the 16 score card metrics on healthcare provision were used in the two surveys. Scores had improved or markedly improved in 15 countries, remained constant in 8 countries and worsened in 3 countries. For Slovakia, the scores were markedly improved.

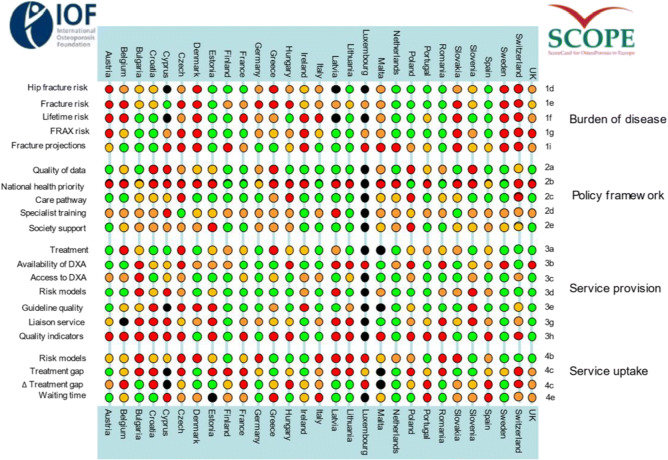



**Fig. 2** The scorecard for all the EU27+2 countries illustrating the scores across the four domains. The elements of each domain in each country were scored and coded using a traffic light system (red, orange, green). Black dots signify missing information

The second edition of the Scorecard for Osteoporosis in Europe (SCOPE 2021) allows health and policy professionals to assess key indicators on the healthcare provision for osteoporosis within countries and between countries within the EU 27+2. The scorecard is not intended as a prescriptive template. Thus, it does not set performance targets but may serve as a guide to the performance targets at which to aim in order to deliver the outcomes required.

**Acknowledgements**

SCOPE was supported by an unrestricted grant from Amgen to the International Osteoporosis Foundation (IOF). Amgen was neither involved in the design nor writing of the report. We are grateful to Anastasia Soulié Mlotek and Dominique Pierroz of the IOF for their help in the administration of SCOPE. The report has been reviewed by the members of the SCOPE Consultation Panel and the relevant IOF National societies, and we are grateful for their local insights on the management of osteoporosis in each country. The source document has been reviewed and endorsed by the Committee of Scientific Advisors of the IOF and benefitted from their feedback.

**References**

1. Kanis JA, Norton N, Harvey NC, Jacobson T, Johansson H, Lorentzon M, McCloskey EV, Willers C, Borgström F (2021) SCOPE 2021: a new scorecard for osteoporosis in Europe. Arch Osteoporos 16:82. 10.1007/s11657-020-00871-9

2. World Health Organisation (1994) Assessment of fracture risk and its application to screening for postmenopausal osteoporosis. Report of a WHO Study Group. World Health Organ Tech Rep Ser, 1994/01/01 edn, pp 1-129

3. Johnell O, Kanis JA, Oden A, Sernbo I, Redlund-Johnell I, Petterson C, De Laet C, Jonsson B (2004) Mortality after osteoporotic fractures. Osteoporos Int 15:38-42

4. Hernlund E, Svedbom A, Ivergard M, Compston J, Cooper C, Stenmark J, McCloskey EV, Jonsson B, Kanis JA (2013) Osteoporosis in the European Union: medical management, epidemiology and economic burden. A report prepared in collaboration with the International Osteoporosis Foundation (IOF) and the European Federation of Pharmaceutical Industry Associations (EFPIA). Arch Osteoporos 8:136

5. Allen P, Pilar M, Walsh-Bailey C, Hooley C, Mazzucca S, Lewis CC, Mettert KD, Dorsey CN, Purtle J, Kepper MM, Baumann AA, Brownson RC (2020) Quantitative measures of health policy implementation determinants and outcomes: a systematic review. Implement Sci 15:47

6. Borgstrom F, Karlsson L, Ortsater G, Norton N, Halbout P, Cooper C, Lorentzon M, McCloskey EV, Harvey NC, Javaid MK, Kanis JA (2020) Fragility fractures in Europe: burden, management and opportunities. Arch Osteoporos 15:59

7. Kanis JA, Oden A, Johnell O, De Laet C, Jonsson B, Oglesby AK (2003) The components of excess mortality after hip fracture. Bone 32:468-473

8. National Clinical Guideline Centre (2011) The Management of Hip Fracture in Adults. In Centre NCG (ed)London

## **Epidemiology and economic burden of osteoporosis in Slovenia**

T Kocjan ∙ C Willers ∙ N Norton ∙ NC Harvey ∙ T Jacobson ∙ H Johansson ∙ M Lorentzon ∙ EV McCloskey ∙ F Borgström ∙ JA Kanis

**Introduction**

The scorecard summarises key indicators of the burden of osteoporosis and its management in the 27 member states of the European Union, as well as the UK and Switzerland (termed EU27+2) [1]. This country-specific report summarises the principal results for Slovenia.

**Methods**

The information obtained covers four domains: burden of osteoporosis and fractures; policy framework; service provision; and service uptake. Data were collected from numerous sources including previous research and IOF reports, and available registers which were used for additional analysis of resource utilization, costing and HRQoL data. Furthermore, country-specific information on osteoporosis management was obtained from each IOF member state via a questionnaire.

**Burden of disease**

The direct cost of incident fractures in Slovenia in 2019 was €60.8 million. Added to this was the ongoing cost in 2019 from fractures that occurred before 2019, which amounted to €26.7 million (long-term disability). The cost of pharmacological intervention (assessment and treatment) was €8.2 million. Thus, the total direct cost (excluding the value of QALYs lost) amounted to €95.7 million in 2019. Key metrics are presented in Table 1.

In 2019, the average direct cost of osteoporotic fractures in Slovenia was €46.3 per individual in the population, while in 2010 the average was €30.9 (after adjusting for inflation), representing an increase of 50% (€46.3 versus €30.9). The 2019 data rank Slovenia in 19^th^ place in terms of highest cost of osteoporotic fractures per capita in the EU27+2.

The cost of osteoporotic fractures in Slovenia accounted for approximately 2.5% of healthcare spending (i.e. €96 million out of €3.5 billion in 2019), which is lower than the EU27+2 average of 3.5%.

Using World Health Organization diagnostic criteria for osteoporosis based on the measurement of bone mineral density (BMD) [2], there were approximately 125,000 individuals with osteoporosis in Slovenia in 2019, of whom approximately 80% were women. The prevalence of osteoporosis in the total Slovenian population amounted to 5.4%, on par with the EU27+2 average (5.6%).

**Table 1** Key measures of burden of disease for Slovenia

**Table:** 

**Category**	**Measure**	**Estimate**	**Rank**
Burden of disease	Direct cost of incident fracture (€m)	60.81	
	Long-term disability cost (€m)	26.74	
	Intervention cost (€m)	8.15	
	Total cost (€m)	95.69	
	QALYs lost (€m)	302	
	Cost per capita (€)	46.29	19
	Proportion of healthcare spending	2.5%	22
	Prevalence of osteoporosis	5.4%	17

There were estimated to be 16,600 new fragility fractures in Slovenia in 2019, equivalent to 46 fractures/day (or 2 per hour). This was a slight decrease compared to 2010, equivalent to a decrement of 1.5 fractures less per 1000 individuals, totalling 18.9 fractures/ 1000 individuals in 2019.

Some osteoporotic fractures are associated with premature mortality [3]. In Slovenia, the annual number of deaths associated with a fracture event was estimated to be 114 per 100,000 individuals of the population aged 50 years or more, compared to the EU27+2 average of 116/100,000. The number of fracture-related deaths is comparable to or exceeds that for some of the most common causes of death such as lung cancer, diabetes, chronic lower respiratory diseases.

The population of men and women age 50 years or more is projected to increase by 13.1% between 2019 and 2034, close to the EU27+2 average of 11.4%. The increases in men and women aged 75 years or more are even more marked and amount to 64.9% and 33.3%, respectively. The annual number of osteoporotic fractures in Slovenia is expected to increase by 5,000 to 21,600 in 2034.

**Policy framework** (Table 2)

Documentation of the burden of disease is an essential prerequisite to determine the resources that should be allocated to the diagnosis and treatment of the disorder. High quality national data on hip fracture rates have been identified in 18 of 29 countries, and Slovenia belonged to the remaining 11 countries.

Given that osteoporosis and fragility fractures are common and that effective treatments are widely available, the vast majority of patients with osteoporosis are preferably managed at the primary health care level by general practitioners (GPs), with specialist referral reserved for difficult complex cases. Primary care was the principal provider of the medical care for osteoporosis in Slovenia, as for 13 of the 28 countries where data were available.

Osteoporosis and metabolic bone disease is not a recognised specialty in most countries including Slovenia. Specialty care of osteoporosis in Slovenia is managed via other specialties including endocrinology, general internal medicine, gynaecology and orthopaedics, and endocrinology. Osteoporosis is however recognized as a component of specialty training. Although it is possible that these specialties educate their trainees adequately, the wide variation may reflect inconsistencies in patient care, training of primary care physicians and a suboptimal voice to “defend” the interests of those who work within the field of osteoporosis.

**Table 2** Policy framework for osteoporosis in Slovenia

**Table:** 

**Category**	**Measure**	**Estimate**
Policy framework	National fracture data availability	No
	OP recognized as a specialty	No
	OP primarily managed in primary care	Yes
	Other specialties involved	Endocrinology, General internal medicine, Rheumatology, Gynaecology, Orthopaedics
	Advocacy areas covered by patient organisation	Policy, capacity, peer support

The role of national patient organisations is to improve the care of patients and increase awareness and prevention of osteoporosis and related fractures among the general public. Advocacy by patient organisations can fall into four categories: policy, capacity building and education, peer support, research and development. For Slovenia, three of these were covered. All four advocacy areas were covered for only 10 out of the 26 countries with at least one patient organisation.

**Service provision** (Table 3)

A wide variety of approved drug treatments is available for the management of osteoporosis [4]. Potential limitations of their use in member states relate to reimbursement policies which may impair the delivery of health care. Slovenia is one of the 12 (out of 27) countries that offer full reimbursement.

The assessment of bone mineral density forms a key component for the general management of osteoporosis, being used for diagnosis, risk prediction, selection of patients for treatment and monitoring of patients on treatment. In Slovenia, the number of DXA units expressed per million of the general population amounted to 18.0 which puts the country in 13th place among the EU27+2.

The average waiting time for DXA ranged from 0 to 180 days across countries, and there was no clear relation between waiting times and the availability of DXA. In Slovenia, the estimated average waiting time for DXA amounted to 7 days. Only four countries reported shorter average waiting times.

**Table 3** Service provision for osteoporosis in Slovenia

**Table:** 

**Category**	**Measure**	**Estimate**	**Rank**
Service provision	Reimbursement of OP medications	100%	
	DXA units/million inhabitants	18	13
	DXA cost (€)	50	13
	FRAX risk assessment model available	No	
	Fracture liaison service density	No FLS	

Reimbursement for DXA scans varied between member states both in terms of the criteria required and level of reimbursement awarded. In Slovenia, the reimbursement was conditional.

The effective targeting of treatment to those at highest risk of fracture requires an assessment of fracture risk. Risk assessment models for fractures, most usually based on FRAX, were available in 24 out of 29 countries, but not in Slovenia. However, the UK FRAX model for assessment of fracture risk was introduced to Slovenia in 2013 and it is now widely used by physicians, nurses and patients.

Guidelines for the management of osteoporosis were available in Slovenia (as in 27 out of 29 countries). The guidelines in Slovenia included postmenopausal women specifically, as well as osteoporosis in men and secondary osteoporosis including glucocorticoid-induced osteoporosis.

Fracture liaison services (FLS), also known as osteoporosis coordinator programmes and care manager programmes, provide a system for the routine assessment and management of postmenopausal women and older men who have sustained a low trauma fracture. No fracture liaison services were reported from Slovenia (together with 7 other countries).

The use of indicators to systematically measure the quality of care provided to people with osteoporosis or associated fractures has expanded as a discipline within the past decade [5]. Slovenia was one of few countries with national quality indicators in place.

**Service uptake** (Table 4)

The web-based usage of FRAX showed considerable heterogeneity in uptake between the countries. The average uptake for the EU27+2 in 2019 was 1,555 sessions/million of the general population with an enormous range of 49 to 41,874 sessions/million. The use of FRAX in Slovenia amounted to 41,874 sessions/million in 2019, with a 31-fold increase since 2011.

Many studies have demonstrated that a significant proportion of men and women at high fracture risk do not receive therapy for osteoporosis (the treatment gap) [6]. In the EU27+2 the average treatment gap was 71% but ranged from 32 to 87%. For Slovenia, the treatment gap amongst women amounted to 57% or 42,000 out of 74,000 characterised at risk and did increase compared to 2010. The average treatment gap among EU27+2 increased from 55% in 2010 to 71% in 2019.

**Table 4** Service uptake for osteoporosis in Slovenia

**Table:** 

**Category**	**Measure**	**Estimate**	**Rank**
Service uptake	Number of FRAX sessions/million people/year	41874	1
	Treatment gap for women eligible for treatment (%)	57	7
	Proportion surgically managed hip fractures	>90%	

About 5% of people with a hip fracture die within 1 month of their fracture [7]. A determinant of peri-operative morbidity and mortality is the time a patient takes to get to surgery [8]. For Slovenia, the average waiting time for hip fracture surgery after hospital admission was reported to be 1–2 days. The proportion of surgically managed hip fractures was reported to be over 90%.

**Scores and scorecard**

Scores were developed for Burden of disease and the healthcare provision (Policy framework, Service provision and Service uptake) in the EU27+2 countries. Slovenia scores resulted in a 16th place regarding Burden of disease. The combined healthcare provision scorecard resulted in a 16th place for Slovenia. Thus, Slovenia presents as one of the five low-burden low-provision countries among the EU27+2.

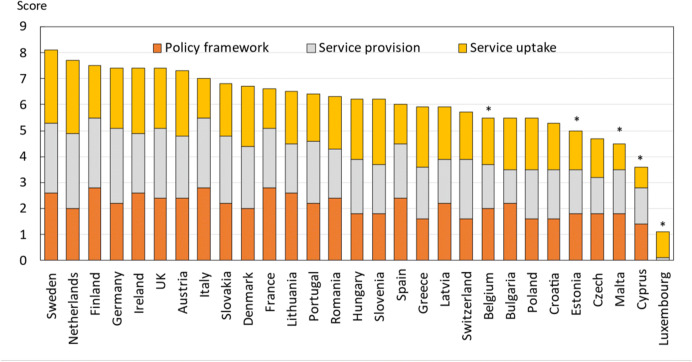



**Fig. 1** Scores by country for metrics related to policy framework, service provision and service uptake. The mean score for each of the 3 domains is given. An asterisk denotes that there was one or more missing metric which decreases the overall score

The first SCOPE was undertaken in 2010, almost 10 years previously. Fifteen of the 16 score card metrics on healthcare provision were used in the two surveys. Scores had improved or markedly improved in 15 countries, remained constant in 8 countries and worsened in 3 countries. For Slovenia the scores were worsened.

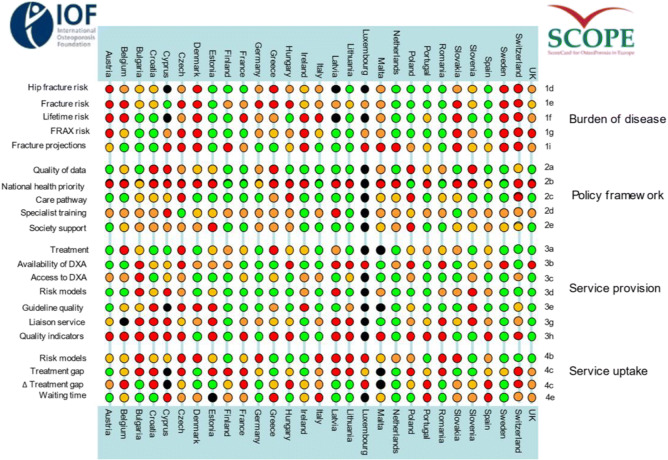



**Fig. 2** The scorecard for all the EU27+2 countries illustrating the scores across the four domains. The elements of each domain in each country were scored and coded using a traffic light system (red, orange, green). Black dots signify missing information

The second edition of the Scorecard for Osteoporosis in Europe (SCOPE 2021) allows health and policy professionals to assess key indicators on the healthcare provision for osteoporosis within countries and between countries within the EU 27+2. The scorecard is not intended as a prescriptive template. Thus, it does not set performance targets but may serve as a guide to the performance targets at which to aim in order to deliver the outcomes required.

**Acknowledgements**

SCOPE was supported by an unrestricted grant from Amgen to the International Osteoporosis Foundation (IOF). Amgen was neither involved in the design nor writing of the report. We are grateful to Anastasia Soulié Mlotek and Dominique Pierroz of the IOF for their help in the administration of SCOPE. The report has been reviewed by the members of the SCOPE Consultation Panel and the relevant IOF National societies, and we are grateful for their local insights on the management of osteoporosis in each country. The source document has been reviewed and endorsed by the Committee of Scientific Advisors of the IOF and benefitted from their feedback.

**References**

1. Kanis JA, Norton N, Harvey NC, Jacobson T, Johansson H, Lorentzon M, McCloskey EV, Willers C, Borgström F (2021) SCOPE 2021: a new scorecard for osteoporosis in Europe. Arch Osteoporos 16:82. 10.1007/s11657-020-00871-9

2. World Health Organisation (1994) Assessment of fracture risk and its application to screening for postmenopausal osteoporosis. Report of a WHO Study Group. World Health Organ Tech Rep Ser, 1994/01/01 edn, pp 1-129

3. Johnell O, Kanis JA, Oden A, Sernbo I, Redlund-Johnell I, Petterson C, De Laet C, Jonsson B (2004) Mortality after osteoporotic fractures. Osteoporos Int 15:38-42

4. Hernlund E, Svedbom A, Ivergard M, Compston J, Cooper C, Stenmark J, McCloskey EV, Jonsson B, Kanis JA (2013) Osteoporosis in the European Union: medical management, epidemiology and economic burden. A report prepared in collaboration with the International Osteoporosis Foundation (IOF) and the European Federation of Pharmaceutical Industry Associations (EFPIA). Arch Osteoporos 8:136

5. Allen P, Pilar M, Walsh-Bailey C, Hooley C, Mazzucca S, Lewis CC, Mettert KD, Dorsey CN, Purtle J, Kepper MM, Baumann AA, Brownson RC (2020) Quantitative measures of health policy implementation determinants and outcomes: a systematic review. Implement Sci 15:47

6. Borgstrom F, Karlsson L, Ortsater G, Norton N, Halbout P, Cooper C, Lorentzon M, McCloskey EV, Harvey NC, Javaid MK, Kanis JA (2020) Fragility fractures in Europe: burden, management and opportunities. Arch Osteoporos 15:59

7. Kanis JA, Oden A, Johnell O, De Laet C, Jonsson B, Oglesby AK (2003) The components of excess mortality after hip fracture. Bone 32:468-473

8. National Clinical Guideline Centre (2011) The Management of Hip Fracture in Adults. In Centre NCG (ed)London

## **Epidemiology and economic burden of osteoporosis in Spain**

A Diez-Perez ∙ M Naves-Díaz ∙ S Palacios ∙ C Willers ∙ N Norton ∙ NC Harvey ∙ T Jacobson ∙ H Johansson ∙ M Lorentzon ∙ EV McCloskey ∙ F Borgström ∙ JA Kanis

**Introduction**

The scorecard summarises key indicators of the burden of osteoporosis and its management in the 27 member states of the European Union, as well as the UK and Switzerland (termed EU27+2) [1]. This country-specific report summarises the principal results for Spain.

**Methods**

The information obtained covers four domains: burden of osteoporosis and fractures; policy framework; service provision; and service uptake. Data were collected from numerous sources including previous research and IOF reports, and available registers which were used for additional analysis of resource utilization, costing and HRQoL data. Furthermore, country-specific information on osteoporosis management was obtained from each IOF member state via a questionnaire.

**Burden of disease**

The direct cost of incident fractures in Spain in 2019 was €1,813 million. Added to this was the ongoing cost in 2019 from fractures that occurred before 2019, which amounted to €2,198 million (long-term disability). The cost of pharmacological intervention (assessment and treatment) was €303 million. Thus, the total direct cost (excluding the value of QALYs lost) amounted to €4.3 billion in 2019. Key metrics are presented in Table 1.

In 2019, the average direct cost of osteoporotic fractures in Spain was €92.3 per individual in the population, while in 2010 the average was €69.5 (after adjusting for inflation) representing an increase of 33% (€92.3 versus €69.5). The 2019 data rank Spain in 12th place in terms of highest cost of osteoporotic fractures per capita in the EU27+2.

The cost of osteoporotic fractures in Spain accounted for approximately 3.8% of healthcare spending (i.e. €4.3 billion out of €104.3 billion in 2019), somewhat more than the EU27+2 average of 3.5% and ranked Spain 11th amongst the EU27+2 countries. These numbers indicate a substantial impact of fragility fractures on the healthcare budget.

Using World Health Organization diagnostic criteria for osteoporosis based on the measurement of bone mineral density (BMD) [2], there were approximately 2,945,000 individuals with osteoporosis in Spain in 2019, of whom almost 80% were women. The prevalence of osteoporosis in the total Spanish population amounted to 5.4%, on par with the EU27+2 average (5.6%).

**Table 1** Key measures of burden of disease for Spain

**Table:** 

**Category**	**Measure**	**Estimate**	**Rank**
Burden of disease	Direct cost of incident fracture (€m)	1813.37	
	Long-term disability cost (€m)	2197.98	
	Intervention cost (€m)	302.95	
	Total cost (€m)	4314.30	
	QALYs lost (€m)	6224	
	Cost per capita (€)	92.34	12
	Proportion of healthcare spending	3.8%	11
	Prevalence of osteoporosis	5.4%	17

There were estimated to be 285,000 new fragility fractures in Spain in 2019, equivalent to 782 fractures/day (or 33 per hour). This was a slight increase compared to 2010, equivalent to an increment of 2.0 fractures/1000 individuals, totalling 14.8 fractures/ 1000 individuals in 2019.

Some osteoporotic fractures are associated with premature mortality [3]. In Spain, the annual number of deaths associated with a fracture event was estimated to be 74 per 100,000 individuals of the population aged 50 years or more, compared to the EU27+2 average of 116/100,000. The number of fracture-related deaths is comparable to or exceeds that for some of the most common causes of death such as lung cancer, diabetes, chronic lower respiratory diseases.

The remaining lifetime probability of hip fracture (%) at the ages of 50 years in men and women was 4.0% and 12.1%, respectively, placing Spain in the bottom tertile of risk for both men and women.

The population of men and women age 50 years or more isprojected to increase by 22.3% between 2019 and 2034, significantly higher than the EU27+2 average of 11.4%. The increases in men and women aged 75 years or more are even more marked and amount to 37.7% and 28.6%, respectively. The annual number of osteoporotic fractures in Spain is expected to increase by 84,000 to 370,000 in 2034.

**Policy framework** (Table 2)

Documentation of the burden of disease is an essential prerequisite to determine the resources that should be allocated to the diagnosis and treatment of the disorder. High quality national data on hip fracture rates have been identified in 18 of 29 countries, of which Spain is one. Data are collected on a national basis and include more than only hip fracture data.

Given that osteoporosis and fragility fractures are common and that effective treatments are widely available, the vast majority of patients with osteoporosis are preferably managed at the primary health care level by general practitioners (GPs), with specialist referral reserved for difficult complex cases. Primary care was the principal provider of the medical care for osteoporosis in Spain, as for 13 of the 28 countries where data were available.

Osteoporosis and metabolic bone disease is not a recognised specialty in most countries including Spain. Specialty care of osteoporosis in Spain is managed via other specialties including internal medicine, orthopaedics, gynaecology, endocrinology, and rheumatology. Osteoporosis is however recognized as a component of specialty training. Although it is possible that these specialties educate their trainees adequately, the wide variation may reflect inconsistencies in patient care, training of primary care physicians and a suboptimal voice to “defend” the interests of those who work within the field of osteoporosis.

**Table 2** Policy framework for osteoporosis in Spain

**Table:** 

**Category**	**Measure**	**Estimate**
Policy framework	National fracture data availability	Yes
	OP recognized as a specialty	No
	OP primarily managed in primary care	Yes
	Other specialties involved	Internal medicine, Orthopaedics, Gynaecology, Endocrinology, Rheumatology, Geriatrics
	Advocacy areas covered by patient organisation	Policy, capacity, research and development

The role of national patient organisations is to improve the care of patients and increase awareness and prevention of osteoporosis and related fractures among the general public. Advocacy by patient organisations can fall into four categories: policy, capacity building and education, peer support, research and development. For Spain, three of these were covered. All four advocacy areas were covered for only 10 out of the 26 countries with at least one patient organisation.

**Service provision** (Table 3)

A wide variety of approved drug treatments is available for the management of osteoporosis [4]. Potential limitations of their use in member states relate to reimbursement policies which may impair the delivery of health care. Twelve out of 27 countries offered full reimbursement, but this was not the case for Spain.

The assessment of bone mineral density forms a key component for the general management of osteoporosis, being used for diagnosis, risk prediction, selection of patients for treatment and monitoring of patients on treatment. In Spain, the number of DXA units expressed per million of the general population amounted to 15.5 which puts the country in 15th place among the EU27+2.

The average waiting time for DXA ranged from 0 to 180 days across countries, and there was no clear relation between waiting times and the availability of DXA. In Spain, the estimated average waiting time for DXA amounted to 180 days.

**Table 3** Service provision for osteoporosis in Spain

**Table:** 

**Category**	**Measure**	**Estimate**	**Rank**
Service provision	Reimbursement of OP medications	90%	
	DXA units/million inhabitants	15.5	15
	DXA cost (€)	90	6
	FRAX risk assessment model available	Yes	
	Fracture liaison service density	1-10%	

Reimbursement for DXA scans varied between member states both in terms of the criteria required and level of reimbursement awarded. In Spain, the reimbursement was unconditional.

The effective targeting of treatment to those at highest risk of fracture requires an assessment of fracture risk. Risk assessment models for fractures, most usually based on FRAX, were available in 24 out of 29 countries, of which Spain was one. For Spain, guidance on the use of risk assessment within national guidelines was available, as in only 14 of the other countries.

Guidelines for the management of osteoporosis were available in Spain (as in 27 out of 29 countries). The guidelines in Spain included postmenopausal women specifically, as well as osteoporosis in men and secondary osteoporosis including glucocorticoid-induced osteoporosis.

Fracture liaison services (FLS), also known as osteoporosis coordinator programmes and care manager programmes, provide a system for the routine assessment and management of postmenopausal women and older men who have sustained a low trauma fracture. Fracture liaison services were reported for 1-10% of hospitals in Spain.

The use of indicators to systematically measure the quality of care provided to people with osteoporosis or associated fractures has expanded as a discipline within the past decade [5]. No use of national quality indicators was reported for Spain.

**Service uptake** (Table 4)

The web-based usage of FRAX showed considerable heterogeneity in uptake between the countries. The average uptake for the EU27+2 in 2019 was 1,555 sessions/million of the general population with an enormous range of 49 to 41,874 sessions/million. The use of FRAX in Spain amounted to 1,527 sessions/million in 2019, with a 37 percent increase since 2011.

Many studies have demonstrated that a significant proportion of men and women at high fracture risk do not receive therapy for osteoporosis (the treatment gap) [6]. In the EU27+2 the average treatment gap was 71% but ranged from 32 to 87%. For Spain, the treatment gap amongst women amounted to 64% or 1,171,000 out of 1,827,000 characterised at risk and it increased by as much as almost 40% compared to 2010. The average treatment gap among EU27+2 increased from 55% in 2010 to 71% in 2019.

**Table 4** Service uptake for osteoporosis in Spain

**Table:** 

**Category**	**Measure**	**Estimate**	**Rank**
Service uptake	Number of FRAX sessions/million people/year	1527	13
	Treatment gap for women eligible for treatment (%)	64	8
	Proportion surgically managed hip fractures	75-90%	

About 5% of people with a hip fracture die within 1 month of their fracture [7]. A determinant of peri-operative morbidity and mortality is the time a patient takes to get to surgery [8]. For Spain, the average waiting time for hip fracture surgery after hospital admission was reported to be more than three days, implying a reduction in waiting time compared to 2010 (waiting time of 2–3 days). The proportion of surgically managed hip fractures was reported to be lie between 75 and 90%.

**Scores and scorecard**

Scores were developed for Burden of disease and the healthcare provision (Policy framework, Service provision and Service uptake) in the EU27+2 countries. Spain scores resulted in a 26th place regarding Burden of disease. The combined healthcare provision scorecard resulted in a 17th place for Spain. Thus, Spain presents as one of the low-burden low-provision countries among the EU27+2.

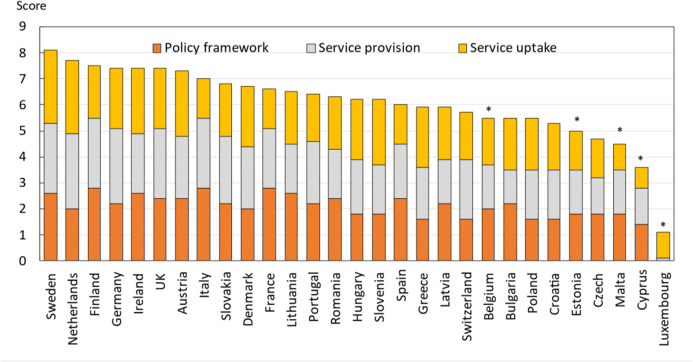



**Fig. 1** Scores by country for metrics related to policy framework, service provision and service uptake. The mean score for each of the 3 domains is given. An asterisk denotes that there was one or more missing metric which decreases the overall score

The first SCOPE was undertaken in 2010, almost 10 years previously. Fifteen of the 16 score card metrics on healthcare provision were used in the two surveys. Scores had improved or markedly improved in 15 countries, remained constant in 8 countries and worsened in 3 countries. For Spain the scores were much improved.

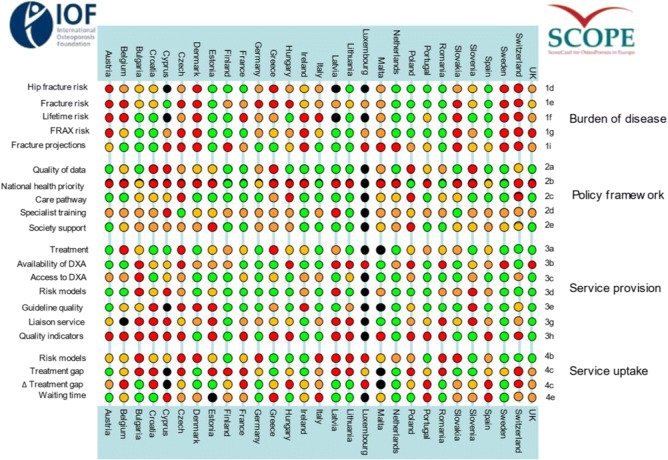



**Fig. 2** The scorecard for all the EU27+2 countries illustrating the scores across the four domains. The elements of each domain in each country were scored and coded using a traffic light system (red, orange, green). Black dots signify missing information

The second edition of the Scorecard for Osteoporosis in Europe (SCOPE 2021) allows health and policy professionals to assess key indicators on the healthcare provision for osteoporosis within countries and between countries within the EU 27+2. The scorecard is not intended as a prescriptive template. Thus, it does not set performance targets but may serve as a guide to the performance targets at which to aim in order to deliver the outcomes required.

**Acknowledgements**

SCOPE was supported by an unrestricted grant from Amgen to the International Osteoporosis Foundation (IOF). Amgen was neither involved in the design nor writing of the report. We are grateful to Anastasia Soulié Mlotek and Dominique Pierroz of the IOF for their help in the administration of SCOPE. The report has been reviewed by the members of the SCOPE Consultation Panel and the relevant IOF National societies, and we are grateful for their local insights on the management of osteoporosis in each country. We are grateful to the Sociedad Española de Investigaciones Óseas y Metabolismo Mineral (SEIOMM) for their review of the Spanish component of this report.The source document has been reviewed and endorsed by the Committee of Scientific Advisors of the IOF and benefitted from their feedback.

**References**

1. Kanis JA, Norton N, Harvey NC, Jacobson T, Johansson H, Lorentzon M, McCloskey EV, Willers C, Borgström F (2021) SCOPE 2021: a new scorecard for osteoporosis in Europe. Arch Osteoporos 16:82. 10.1007/s11657-020-00871-9

2. World Health Organisation (1994) Assessment of fracture risk and its application to screening for postmenopausal osteoporosis. Report of a WHO Study Group. World Health Organ Tech Rep Ser, 1994/01/01 edn, pp 1-129

3. Johnell O, Kanis JA, Oden A, Sernbo I, Redlund-Johnell I, Petterson C, De Laet C, Jonsson B (2004) Mortality after osteoporotic fractures. Osteoporos Int 15:38-42

4. Hernlund E, Svedbom A, Ivergard M, Compston J, Cooper C, Stenmark J, McCloskey EV, Jonsson B, Kanis JA (2013) Osteoporosis in the European Union: medical management, epidemiology and economic burden. A report prepared in collaboration with the International Osteoporosis Foundation (IOF) and the European Federation of Pharmaceutical Industry Associations (EFPIA). Arch Osteoporos 8:136

5. Allen P, Pilar M, Walsh-Bailey C, Hooley C, Mazzucca S, Lewis CC, Mettert KD, Dorsey CN, Purtle J, Kepper MM, Baumann AA, Brownson RC (2020) Quantitative measures of health policy implementation determinants and outcomes: a systematic review. Implement Sci 15:47

6. Borgstrom F, Karlsson L, Ortsater G, Norton N, Halbout P, Cooper C, Lorentzon M, McCloskey EV, Harvey NC, Javaid MK, Kanis JA (2020) Fragility fractures in Europe: burden, management and opportunities. Arch Osteoporos 15:59

7. Kanis JA, Oden A, Johnell O, De Laet C, Jonsson B, Oglesby AK (2003) The components of excess mortality after hip fracture. Bone 32:468-473

8. National Clinical Guideline Centre (2011) The Management of Hip Fracture in Adults. In Centre NCG (ed) London

## **Epidemiology and economic burden of osteoporosis in Sweden**

KE Åkesson ∙ B Freyschuss ∙ C Willers ∙ N Norton ∙ NC Harvey ∙ T Jacobson ∙ H Johansson ∙ M Lorentzon ∙ EV McCloskey ∙ F Borgström ∙ JA Kanis

**Introduction**

The scorecard summarises key indicators of the burden of osteoporosis and its management in the 27 member states of the European Union, as well as the UK and Switzerland (termed EU27+2) [1]. This country-specific report summarises the principal results for Sweden.

**Methods**

The information obtained covers four domains: burden of osteoporosis and fractures; policy framework; service provision; and service uptake. Data were collected from numerous sources including previous research and IOF reports, and available registers which were used for additional analysis of resource utilization, costing and HRQoL data. Furthermore, country-specific information on osteoporosis management was obtained from each IOF member state via a questionnaire.

**Burden of disease**

The direct cost of incident fractures in Sweden in 2019 was €1,440 million. Added to this was the ongoing cost in 2019 from fractures that occurred before 2019, which amounted to €848 million (long-term disability). The cost of pharmacological intervention (assessment and treatment) was €45 million. Thus, the total direct cost (excluding the value of QALYs lost) amounted to €2.3 billion in 2019. Key metrics are presented in Table 1.

In 2019, the average direct cost of osteoporotic fractures in Sweden was €229.1 per individual in the population, while in 2010 the average was €176.6 (after adjusting for inflation), representing an increase of 30% (€229.1 versus €176.6). The 2019 data rank Sweden in 3rd place in terms of highest cost of osteoporotic fractures per capita in the EU27+2.

The cost of osteoporotic fractures in Sweden accounted for approximately 4.3% of healthcare spending (i.e. €2.3 billion out of €52.8 billion in 2019), which is substantially higher than the EU27+2 average of 3.5% and rank Sweden 8th amongst the EU27+2 countries. These numbers indicate a substantial impact of fragility fractures on the healthcare budget.

Using World Health Organization diagnostic criteria for osteoporosis based on the measurement of bone mineral density (BMD) [2], there were approximately 583,000 individuals with osteoporosis in Sweden in 2019, of whom almost 80% were women. The prevalence of osteoporosis in the total Swedish population amounted to 5.6%, on par with the EU27+2 average (5.6%).

**Table 1** Key measures of burden of disease for Sweden

**Table:** 

**Category**	**Measure**	**Estimate**	**Rank**
Burden of disease	Direct cost of incident fracture (€m)	1440.28	
	Long-term disability cost (€m)	848.47	
	Intervention cost (€m)	44.63	
	Total cost (€m)	2333.37	
	QALYs lost (€m)	4457	
	Cost per capita (€)	229.14	3
	Proportion of healthcare spending	4.3%	8
	Prevalence of osteoporosis	5.6%	11

There were estimated to be 124,000 new fragility fractures in Sweden in 2019, equivalent to 338 fractures/day (or 14 per hour). This was a slight increase compared to 2010, equivalent to an increment of 0.9 fractures/1000 individuals, totalling 31.6 fractures/ 1000 individuals in 2019. These estimates differ somewhat from data available from Swedish register because of differences in the methodology of data acquisition [3].

Some osteoporotic fractures are associated with premature mortality [4]. In Sweden, the annual number of deaths associated with a fracture event was estimated to be 168 per 100,000 individuals of the population aged 50 years or more, compared to the EU27+2 average of 116/100,000. The number of fracture-related deaths is comparable to or exceeds that for some of the most common causes of death such as lung cancer, diabetes, chronic lower respiratory diseases.

The remaining lifetime probability of hip fracture (%) at the ages of 50 years in men and women was 10.9% and 25.1%, respectively, placing Sweden in the upper tertile of risk for both men and women.

The population of men and women age 50 years or more is projected to increase by 12.0% between 2019 and 2034, close to the EU27+2 average of 11.4%. The increases in men and women aged 75 years or more are even more marked and amount to 43.0% and 32.0%, respectively. The annual number of osteoporotic fractures in Sweden is expected to increase by 37,000 to 161,000 in 2034.

**Policy framework** (Table 2)

Documentation of the burden of disease is an essential prerequisite to determine the resources that should be allocated to the diagnosis and treatment of the disorder. High quality national data on hip fracture rates have been identified in 18 of 29 countries, of which Sweden is one. Data are collected on a national basis and include more than only hip fracture data.

Given that osteoporosis and fragility fractures are common and that effective treatments are widely available, the vast majority of patients with osteoporosis are preferably managed at the primary health care level by general practitioners (GPs), with specialist referral reserved for difficult complex cases. Primary care was the principal provider of the medical care for osteoporosis in Sweden, as for 13 of the 28 countries where data were available.

Osteoporosis and metabolic bone disease is not a recognised specialty in most countries including Sweden. Specialty care of osteoporosis in Sweden is managed via other specialties including orthopaedics, endocrinology and geriatrics. Osteoporosis is however recognized as a component of specialty training. Although it is possible that these specialties educate their trainees adequately, the wide variation may reflect inconsistencies in patient care, training of primary care physicians and a suboptimal voice to “defend” the interests of those who work within the field of osteoporosis.

**Table 2** Policy framework for osteoporosis in Sweden

**Table:** 

**Category**	**Measure**	**Estimate**
Policy framework	National fracture data availability	Yes
	OP recognized as a specialty	No
	OP primarily managed in primary care	Yes
	Other specialties involved	Orthopaedics, Endocrinology, Geriatrics
	Advocacy areas covered by patient organisation	Policy, capacity, peer support

The role of national patient organisations is to improve the care of patients and increase awareness and prevention of osteoporosis and related fractures among the general public. Advocacy by patient organisations can fall into four categories: policy, capacity building and education, peer support, research and development. For Sweden, three of these were covered. All four advocacy areas were covered for only 10 out of the 26 countries with at least one patient organisation.

**Service provision** (Table 3)

A wide variety of approved drug treatments is available for the management of osteoporosis [5]. Potential limitations of their use in member states relate to reimbursement policies which may impair the delivery of health care. Sweden is one of the 12 (out of 27) countries that offer full reimbursement.

The assessment of bone mineral density forms a key component for the general management of osteoporosis, being used for diagnosis, risk prediction, selection of patients for treatment and monitoring of patients on treatment. In Sweden, the number of DXA units expressed per million of the general population amounted to 7.4 which puts the country in 24th place among the EU27+2. Furthermore, the relative availability of TBS was high in Sweden.

The average waiting time for DXA ranged from 0 to 180 days across countries, and there was no clear relation between waiting times and the availability of DXA. In Sweden, the estimated average waiting time for DXA amounted to 90 days. Twenty-three countries reported shorter average waiting times.

**Table 3** Service provision for osteoporosis in Sweden

**Table:** 

**Category**	**Measure**	**Estimate**	**Rank**
Service provision	Reimbursement of OP medications	100%	
	DXA units/million inhabitants	7.4	24
	DXA cost (€)	85	8
	FRAX risk assessment model available	Yes	
	Fracture liaison service density	25-50%	

Reimbursement for DXA scans varied between member states both in terms of the criteria required and level of reimbursement awarded. In Sweden, the reimbursement was unconditional.

The effective targeting of treatment to those at highest risk of fracture requires an assessment of fracture risk. Risk assessment models for fractures, most usually based on FRAX, were available in 24 out of 29 countries, of which Sweden was one. For Sweden, guidance on the use of risk assessment within national guidelines was available, as in only 14 of the other countries.

Guidelines for the management of osteoporosis were available in Sweden (as in 27 out of 29 countries). The guidelines in Sweden included postmenopausal women specifically, as well as osteoporosis in men and secondary osteoporosis including glucocorticoid-induced osteoporosis.

Fracture liaison services (FLS), also known as osteoporosis coordinator programmes and care manager programmes, provide a system for the routine assessment and management of postmenopausal women and older men who have sustained a low trauma fracture. Fracture liaison services were reported for 25–50% of hospitals in Sweden.

The use of indicators to systematically measure the quality of care provided to people with osteoporosis or associated fractures has expanded as a discipline within the past decade [6]. Sweden was one of few countries with national quality indicators in place.

**Service uptake** (Table 4)

The web-based usage of FRAX showed considerable heterogeneity in uptake between the countries. The average uptake for the EU27+2 was 1,555 sessions/million/year of the general population with an enormous range of 49 to 41,874 sessions/million. The use of FRAX in Sweden amounted to 5,306 sessions/million in 2019, with a 178 percent increase since 2011.

Many studies have demonstrated that a significant proportion of men and women at high fracture risk do not receive therapy for osteoporosis (the treatment gap) [7]. In the EU27+2 the average treatment gap was 71% but ranged from 32 to 87%. For Sweden, the treatment gap amongst women amounted to 67% or 261,000 out of 389,000 characterised at risk. The Swedish treatment gap did not change significantly compared to 2010, whilst the average treatment gap among EU27+2 increased from 55% in 2010 to 71% in 2019.

**Table 4** Service uptake for osteoporosis in Sweden

**Table:** 

**Category**	**Measure**	**Estimate**	**Rank**
Service uptake	Number of FRAX sessions/million people/year	5306	3
	Treatment gap for women eligible for treatment (%)	67	12
	Proportion surgically managed hip fractures	>90%	

About 5% of people with a hip fracture die within 1 month of their fracture [8]. A determinant of peri-operative morbidity and mortality is the time a patient takes to get to surgery [9]. For Sweden, the average waiting time for hip fracture surgery after hospital admission was reported to be less than 24 hours. The proportion of surgically managed hip fractures was reported to be over 90%.

**Scores and scorecard**

Scores were developed for Burden of disease and the healthcare provision (Policy framework, Service provision and Service uptake) in the EU27+2 countries. Sweden scores resulted in a 2nd place regarding Burden of disease after only Denmark. The combined healthcare provision scorecard resulted in a 1st place for Sweden. Thus, Sweden presents as one of the high-burden high-provision countries among the EU27+2.

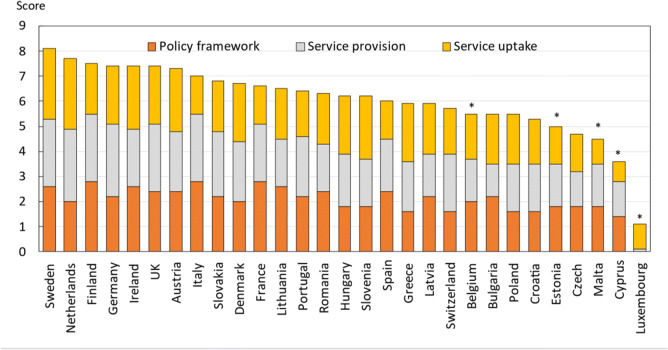



**Fig. 1** Scores by country for metrics related to policy framework, service provision and service uptake. The mean score for each of the 3 domains is given. An asterisk denotes that there was one or more missing metric which decreases the overall score

The first SCOPE was undertaken in 2010, almost 10 years previously. Fifteen of the 16 score card metrics on healthcare provision were used in the two surveys. Scores had improved or markedly improved in 15 countries, remained constant in 8 countries and worsened in 3 countries. For Sweden the scores were almost unchanged.

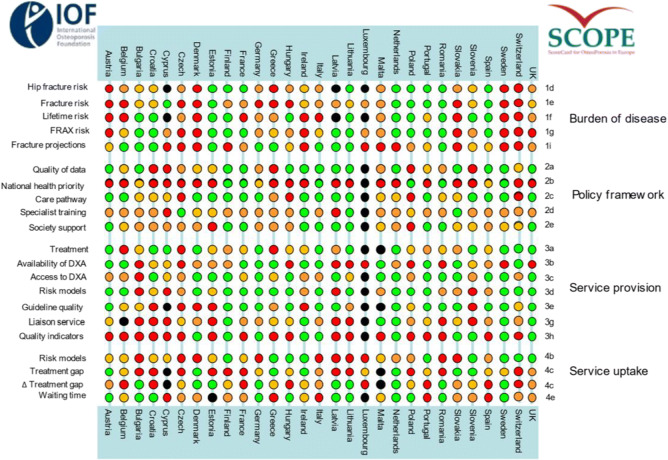



**Fig. 2** The scorecard for all the EU27+2 countries illustrating the scores across the four domains. The elements of each domain in each country were scored and coded using a traffic light system (red, orange, green). Black dots signify missing information

The second edition of the Scorecard for Osteoporosis in Europe (SCOPE 2021) allows health and policy professionals to assess key indicators on the healthcare provision for osteoporosis within countries and between countries within the EU 27+2. The scorecard is not intended as a prescriptive template. Thus, it does not set performance targets but may serve as a guide to the performance targets at which to aim in order to deliver the outcomes required.

**Acknowledgements**

SCOPE was supported by an unrestricted grant from Amgen to the International Osteoporosis Foundation (IOF). Amgen was neither involved in the design nor writing of the report. We are grateful to Anastasia Soulié Mlotek and Dominique Pierroz of the IOF for their help in the administration of SCOPE. We acknowledge the assistance of the Swediish Osteoporosis Society. The report has been reviewed by the members of the SCOPE Consultation Panel and the relevant IOF National societies, and we are grateful for their local insights on the management of osteoporosis in each country. The source document has been reviewed and endorsed by the Committee of Scientific Advisors of the IOF and benefitted from their feedback.

**References**

1. Kanis JA, Norton N, Harvey NC, Jacobson T, Johansson H, Lorentzon M, McCloskey EV, Willers C, Borgström F (2021) SCOPE 2021: a new scorecard for osteoporosis in Europe. Arch Osteoporos 16:82. 10.1007/s11657-020-00871-9

2. World Health Organisation (1994) Assessment of fracture risk and its application to screening for postmenopausal osteoporosis. Report of a WHO Study Group. World Health Organ Tech Rep Ser, 1994/01/01 edn, pp 1-129

3. National Board of Health and Welfare, 2021-1-17137; https://www.socialstyrelsen.se/regler-och-riktlinjer/nationella-riktlinjer/riktlinjer-och-utvarderingar/rorelseorganens-sjukdomar/

4. Johnell O, Kanis JA, Oden A, Sernbo I, Redlund-Johnell I, Petterson C, De Laet C, Jonsson B (2004) Mortality after osteoporotic fractures. Osteoporos Int 15:38-42

5. Hernlund E, Svedbom A, Ivergard M, Compston J, Cooper C, Stenmark J, McCloskey EV, Jonsson B, Kanis JA (2013) Osteoporosis in the European Union: medical management, epidemiology and economic burden. A report prepared in collaboration with the International Osteoporosis Foundation (IOF) and the European Federation of Pharmaceutical Industry Associations (EFPIA). Arch Osteoporos 8:136

6. Allen P, Pilar M, Walsh-Bailey C, Hooley C, Mazzucca S, Lewis CC, Mettert KD, Dorsey CN, Purtle J, Kepper MM, Baumann AA, Brownson RC (2020) Quantitative measures of health policy implementation determinants and outcomes: a systematic review. Implement Sci 15:47

7. Borgstrom F, Karlsson L, Ortsater G, Norton N, Halbout P, Cooper C, Lorentzon M, McCloskey EV, Harvey NC, Javaid MK, Kanis JA (2020) Fragility fractures in Europe: burden, management and opportunities. Arch Osteoporos 15:59

8. Kanis JA, Oden A, Johnell O, De Laet C, Jonsson B, Oglesby AK (2003) The components of excess mortality after hip fracture. Bone 32:468-473

9. National Clinical Guideline Centre (2011) The Management of Hip Fracture in Adults. In Centre NCG

## **Epidemiology and economic burden of osteoporosis in Switzerland**

S Ferrari ∙ R Rizzoli ∙ C Willers ∙ N Norton ∙ NC Harvey ∙ T Jacobson ∙ H Johansson ∙ M Lorentzon ∙ EV McCloskey ∙ F Borgström ∙ JA Kanis

**Introduction**

The scorecard summarises key indicators of the burden of osteoporosis and its management in the 27 member states of the European Union, as well as the UK and Switzerland (termed EU27+2) [1]. This country-specific report summarises the principal results for Switzerland.

**Methods**

The information obtained covers four domains: burden of osteoporosis and fractures; policy framework; service provision; and service uptake. Data were collected from numerous sources including previous research and IOF reports, and available registers which were used for additional analysis of resource utilization, costing and HRQoL data. Furthermore, country-specific information on osteoporosis management was obtained from each IOF member state via a questionnaire. Switzerland was not formally included in SCOPE in 2010 but some comparative data were available and included where possible [2].

**Burden of disease**

The direct cost of incident fractures in Switzerland in 2019 was €2.62 billion. Added to this was the ongoing cost in 2019 from fractures that occurred before 2019, which amounted to €746 million (long-term disability). The cost of pharmacological intervention (assessment and treatment) was €60 million. Thus, the total direct cost (excluding the value of QALYs lost) amounted to €3.43 billion in 2019. Key metrics are presented in Table 1.

In 2019, the average direct cost of osteoporotic fractures in Switzerland was €402.8 per individual in the population, while in 2010 the average was €190.2 (after adjusting for inflation) representing an increase of 112% (€402.8 versus €190.2). The 2019 estimate places Switzerland in 1^st^ place in terms of highest cost of osteoporotic fractures per capita in the EU27+2.

The cost of osteoporotic fractures in Switzerland accounted for approximately 4.5% of healthcare spending (i.e. €3.4 billion out of €74.9 billion in 2019), which is higher than the EU27+2 average of 3.5% and placed Switzerland at 7th place in the ranking across the EU27+2 countries. These data indicate a substantial impact of fragility fractures on the healthcare budget.

Using World Health Organization diagnostic criteria for osteoporosis based on the measurement of bone mineral density (BMD) [3], there were approximately 524,000 individuals with osteoporosis in Switzerland in 2019, of whom almost 80% were women. The prevalence of osteoporosis in the total Swiss population amounted to 6.1%, on a par with the EU27+2 average (5.6%).

**Table 1** Key measures of burden of disease for Switzerland

**Table:** 

**Category**	**Measure**	**Estimate**	**Rank**
Burden of disease	Direct cost of incident fracture (€m)	2624.76	
	Long-term disability cost (€m)	745.65	
	Intervention cost (€m)	59.91	
	Total cost (€m)	3430.32	
	QALYs lost (€m)	5166	
	Cost per capita (€)	402.78	1
	Proportion of healthcare spending	4.5%	7
	Prevalence of osteoporosis	6.1%	3

There were estimated to be 82,000 new fragility fractures in Switzerland in 2019, equivalent to 226 fractures/day (or 9.4 per hour). This was a slight decrease compared to 2010, equivalent to a decrement of 0.9 fractures per 1000 individuals, totalling 23.5 fractures/ 1000 individuals in 2019.

Some osteoporotic fractures are associated with premature mortality [4]. In Switzerland, the annual number of deaths associated with a fracture event was estimated to be 107 per 100,000 individuals of the population aged 50 years or more, compared to the EU27+2 average of 116/100,000. The number of fracture-related deaths is comparable to or exceeds that for some of the most common causes of death such as lung cancer, diabetes, chronic lower respiratory diseases.

The remaining lifetime probability of hip fracture (%) at the ages of 50 years in men and women was 7.1% and 22.5%, respectively, placing Switzerland in the upper tertile of risk for both men and women.

The population in men and women age 50 years or more is projected to increase by 18.7% between 2019 and 2034, significantly above the EU27+2 average of 11.4%. The increases in men and women aged 75 years or more are even more marked and amount to 57.0% and 39.1%, respectively. The annual number of osteoporotic fractures in Switzerland is expected to increase by 31,000 to 113,000 in 2034.

**Policy framework** (Table 2)

Documentation of the burden of disease is an essential prerequisite to determine the resources that should be allocated to the diagnosis and treatment of the disorder. High quality national data on hip fracture rates have been identified in 18 of 29 countries, of which Switzerland is one. The administrative and medical statistics database of the Swiss Federal Statistical Office (SFSO) provides data on a national basis for hospital admissions [5].

Given that osteoporosis and fragility fractures are common and that effective treatments are widely available, the vast majority of patients with osteoporosis are preferably managed at the primary health care level by general practitioners (GPs), with specialist referral reserved for difficult complex cases. Primary care was the principal provider of the medical care for osteoporosis in 13 of the 28 countries where data were available, but this is not the case for Switzerland.

Osteoporosis and metabolic bone disease is not a recognised specialty in most countries including Switzerland. Specialty care of osteoporosis in Switzerland is managed via other specialties including endocrinology, rheumatology, gynaecology, geriatrics and internal medicine. Osteoporosis is however recognized as a component of specialty training. Although it is possible that these specialties educate their trainees adequately, the wide variation may reflect inconsistencies in patient care, training of primary care physicians and a suboptimal voice to “defend” the interests of those who work within the field of osteoporosis.

**Table 2** Policy framework for osteoporosis in Switzerland

**Table:** 

**Category**	**Measure**	**Estimate**
Policy framework	National fracture data availability	No
	OP recognized as a specialty	No
	OP primarily managed in primary care	No
	Other specialties involved	Endocrinology, Rheumatology, Gynaecology, Geriatrics, Internal Medicine
	Advocacy areas covered by osteoporosis organisation	Peer support

The role of national osteoporosis organisations is to improve the care of patients and increase awareness and prevention of osteoporosis and related fractures among the general public. Advocacy by t organisations can fall into four categories: policy, capacity building and education, peer support, research and development. For Switzerland, only one of the advocacy areas were covered. All four advocacy areas were covered for only 10 out of the 26 countries with at least one patient organisation.

**Service provision** (Table 3)

A wide variety of approved drug treatments is available for the management of osteoporosis [6]. Potential limitations of their use in member states relate to reimbursement policies which may impair the delivery of health care. Switzerland is one of the 12 (out of 27) countries that offer full reimbursement.

The assessment of bone mineral density forms a key component for the general management of osteoporosis, being used for diagnosis, risk prediction, selection of patients for treatment and monitoring of patients on treatment. In Switzerland, the number of DXA units expressed per million of the general population amounted to 26.9 which puts the country in 5th place among the EU27+2. Furthermore, the availability of TBS was second highest in Switzerland comparing all EU27+2 countries.

The average waiting time for DXA ranged from 0 to 180 days across countries, and there was no clear relation between waiting times and the availability of DXA. In Switzerland, the estimated average waiting time for DXA amounted to 14 days. Nine countries reported shorter average waiting times.

**Table 3** Service provision for osteoporosis in Switzerland

**Table:** 

**Category**	**Measure**	**Estimate**	**Rank**
Service provision	Reimbursement of OP medications	100%	
	DXA units/million inhabitants	26.9	5
	DXA cost (€)	70	10
	FRAX risk assessment model available	Yes	
	Fracture liaison service density	1-10%	

Reimbursement for DXA scans varied between member states both in terms of the criteria required and level of reimbursement awarded. In Switzerland, the reimbursement was conditional.

The effective targeting of treatment to those at highest risk of fracture requires an assessment of fracture risk. Risk assessment models for fractures, most usually based on FRAX, were available in 24 out of 29 countries, of which Switzerland was one. An additional risk assessment model, Tool Osteoporose-Plattform TOP, was also used in Switzerland. For Switzerland, guidance on the use of risk assessment within national guidelines was available, as in only 14 of the other countries.

Guidelines for the management of osteoporosis were available in Switzerland (as in 27 out of 29 countries). The guidelines in Switzerland included postmenopausal women specifically, as well as osteoporosis in men and secondary osteoporosis including glucocorticoid-induced osteoporosis.

Fracture liaison services (FLS), also known as osteoporosis coordinator programmes and care manager programmes, provide a system for the routine assessment and management of postmenopausal women and older men who have sustained a low trauma fracture. Fracture liaison services were reported for 1–10% of hospitals in Switzerland.

The use of indicators to systematically measure the quality of care provided to people with osteoporosis or associated fractures has expanded as a discipline within the past decade [7]. No use of national quality indicators was reported for Switzerland.

**Service uptake** (Table 4)

The web-based usage of FRAX showed considerable heterogeneity in uptake between the countries. The average uptake for the EU27+2 in 2019 was 1,555 sessions/million of the general population with an enormous range of 49 to 41,874 sessions/million. The usage for Switzerland amounted to 3,702 sessions/million in 2019. No data were available for 2010.

Many studies have demonstrated that a significant proportion of men and women at high fracture risk do not receive therapy for osteoporosis (the treatment gap) [8]. In the EU27+2 the average treatment gap was 71% but ranged from 32 to 87%. For Switzerland, the treatment gap amongst women amounted to 83% or 684,000 out of 827,000 characterised at risk andhad increased significantly from 56% in 2010. The average treatment gap among EU27+2 increased from 55% in 2010 to 71% in 2019.

**Table 4** Service uptake for osteoporosis in Switzerland

**Table:** 

**Category**	**Measure**	**Estimate**	**Rank**
Service uptake	Number of FRAX sessions/million people/year	3702	6
	Treatment gap for women eligible for treatment (%)	83	25
	Proportion surgically managed hip fractures	>90%	

About 5% of people with a hip fracture die within 1 month of their fracture [9]. A determinant of peri-operative morbidity and mortality is the time a patient takes to get to surgery [10]. For Switzerland, the average waiting time for hip fracture surgery after hospital admission was reported to be 1–2 days. The proportion of surgically managed hip fractures was reported to be over 90%.

**Scores and scorecard**

Scores were developed for Burden of disease and the healthcare provision (Policy framework, Service provision and Service uptake) in the EU27+2 countries. Switzerland scores resulted in a 3rd place regarding Burden of disease after only Denmark and Sweden. The combined healthcare provision scorecard resulted in a 20th place for Switzerland. Thus, Switzerland presents as one of the high-burden low-provision countries among the EU27+2.

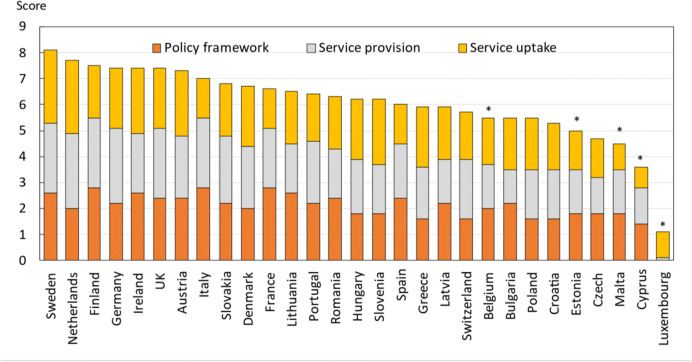



**Fig. 1** Scores by country for metrics related to policy framework, service provision and service uptake. The mean score for each of the 3 domains is given. An asterisk denotes that there was one or more missing metric which decreases the overall score

The first SCOPE was undertaken in 2010, almost 10 years previously. Fifteen of the 16 score card metrics on healthcare provision were used in the two surveys. Scores had improved or markedly improved in 15 countries, remained constant in 8 countries and worsened in 3 countries. Comparative data for Switzerland were not available since Switzerland was not included in the 2010 scorecard.

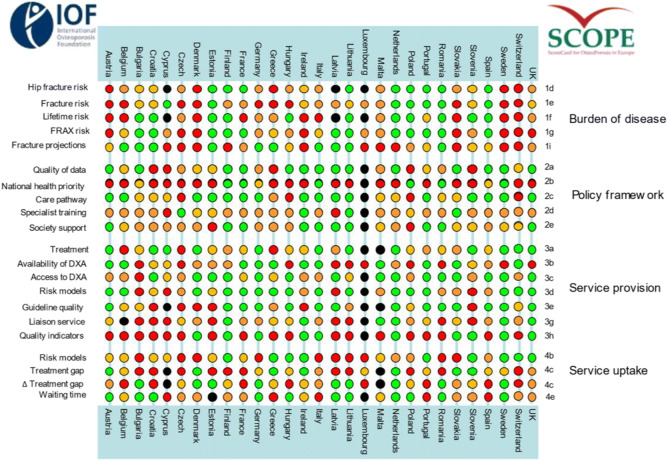



**Fig. 2** The scorecard for all the EU27+2 countries illustrating the scores across the four domains. The elements of each domain in each country were scored and coded using a traffic light system (red, orange, green). Black dots signify missing information

The second edition of the Scorecard for Osteoporosis in Europe (SCOPE 2021) allows health and policy professionals to assess key indicators on the healthcare provision for osteoporosis within countries and between countries within the EU 27+2. The scorecard is not intended as a prescriptive template. Thus, it does not set performance targets but may serve as a guide to the performance targets at which to aim in order to deliver the outcomes required.

**Acknowledgements**

SCOPE was supported by an unrestricted grant from Amgen to the International Osteoporosis Foundation (IOF). Amgen was neither involved in the design nor writing of the report. We are grateful to Anastasia Soulié Mlotek and Dominique Pierroz of the IOF for their help in the administration of SCOPE. The report has been reviewed by the members of the SCOPE Consultation Panel and the relevant IOF National societies, and we are grateful for their local insights on the management of osteoporosis in each country. The source document has been reviewed and endorsed by the Committee of Scientific Advisors of the IOF and benefitted from their feedback.

**References**

1. Kanis JA, Norton N, Harvey NC, Jacobson T, Johansson H, Lorentzon M, McCloskey EV, Willers C, Borgström F (2021) SCOPE 2021: a new scorecard for osteoporosis in Europe. Arch Osteoporos 16:82. 10.1007/s11657-020-00871-9

2. Svedbom A, Ivergård M, Hernlund E, Rizzoli R, Kanis JA (2014) Epidemiology and economic burden of osteoporosis in Switzerland. Archives of Osteoporosis 9(1): 187. doi: 10.1007/s11657-014-0187-y

3. World Health Organisation (1994) Assessment of fracture risk and its application to screening for postmenopausal osteoporosis. Report of a WHO Study Group. World Health Organ Tech Rep Ser, 1994/01/01 edn, pp 1-129

4. Johnell O, Kanis JA, Oden A, Sernbo I, Redlund-Johnell I, Petterson C, De Laet C, Jonsson B (2004) Mortality after osteoporotic fractures. Osteoporos Int 15:38-42

5. Lippuner K, Grifone S, Schwenkglenks M, Schwab P, Popp AW, Senn C, Perrelet R. Comparative trends in hospitalizations for osteoporotic fractures and other frequent diseases between 2000 and 2008. Osteoporos Int. 2012 Mar;23(3):829-39

6. Hernlund E, Svedbom A, Ivergard M, Compston J, Cooper C, Stenmark J, McCloskey EV, Jonsson B, Kanis JA (2013) Osteoporosis in the European Union: medical management, epidemiology and economic burden. A report prepared in collaboration with the International Osteoporosis Foundation (IOF) and the European Federation of Pharmaceutical Industry Associations (EFPIA). Arch Osteoporos 8:136

7. Allen P, Pilar M, Walsh-Bailey C, Hooley C, Mazzucca S, Lewis CC, Mettert KD, Dorsey CN, Purtle J, Kepper MM, Baumann AA, Brownson RC (2020) Quantitative measures of health policy implementation determinants and outcomes: a systematic review. Implement Sci 15:47

8. Borgstrom F, Karlsson L, Ortsater G, Norton N, Halbout P, Cooper C, Lorentzon M, McCloskey EV, Harvey NC, Javaid MK, Kanis JA (2020) Fragility fractures in Europe: burden, management and opportunities. Arch Osteoporos 15:59

9. Kanis JA, Oden A, Johnell O, De Laet C, Jonsson B, Oglesby AK (2003) The components of excess mortality after hip fracture. Bone 32:468-473

10. National Clinical Guideline Centre (2011) The Management of Hip Fracture in Adults. In Centre NCG (ed) London

## **Epidemiology and economic burden of osteoporosis in the United Kingdom**

MK Javaid ∙ C Jones ∙ C Cooper ∙ C Willers ∙ N Norton ∙ NC Harvey ∙ T Jacobson ∙ H Johansson ∙ M Lorentzon ∙ EV McCloskey ∙ F Borgström ∙ JA Kanis

**Introduction**

The scorecard summarises key indicators of the burden of osteoporosis and its management in the 27 member states of the European Union, as well as the UK and Switzerland (termed EU27+2) [1]. This country-specific report summarises the principal results for the UK.

**Methods**

The information obtained covers four domains: burden of osteoporosis and fractures; policy framework; service provision; and service uptake. Data were collected from numerous sources including previous research and IOF reports, and available registers which were used for additional analysis of resource utilization, costing and HRQoL data. Furthermore, country-specific information on osteoporosis management was obtained from each IOF member state via a questionnaire.

**Burden of disease**

The direct cost of incident fractures in the UK in 2019 was €3.0 billion. Added to this was the ongoing cost in 2019 from fractures that occurred before 2019, which amounted to €2.3 billion (long-term disability). The cost of pharmacological intervention (assessment and treatment) was €111 million. Thus, the total direct cost (excluding the value of QALYs lost) amounted to €5.5 billion in 2019. Key metrics are presented in Table 1.

In 2019, the average direct cost of osteoporotic fractures in the UK was €82.5 per individual in the population, while in 2010 the average was €96.0 (after adjusting for inflation) amounting to a decrease of 14% (€82.5 versus €96.0). The 2019 data rank the UK in 14th place in terms of highest cost of osteoporotic fractures per capita in the EU27+2.

The cost of osteoporotic fractures in the UK accounted for approximately 2.4% of healthcare spending (i.e. €5.5 billion out of €227.2 billion in 2019), which is lower than the EU27+2 average of 3.5%.

Using World Health Organization diagnostic criteria for osteoporosis based on the measurement of bone mineral density (BMD) [2], there were approximately 3,775,000 individuals with osteoporosis in the UK in 2019, of whom almost 80% were women. The prevalence of osteoporosis in the total population amounted to 5.2%, on par with the EU27+2 average (5.6%).

**Table 1** Key measures of burden of disease for the UK

**Table:** 

**Category**	**Measure**	**Estimate**	**Rank**
Burden of disease	Direct cost of incident fracture (€m)	3031.07	
	Long-term disability cost (€m)	2339.81	
	Intervention cost (€m)	111.21	
	Total cost (€m)	5482.09	
	QALYs lost (€m)	14465	
	Cost per capita (€)	82.45	14
	Proportion of healthcare spending	2.4%	24
	Prevalence of osteoporosis	5.2%	19

There were estimated to be 527,000 new fragility fractures in the UK in 2019, equivalent to 1,444 fractures/day (or 60 per hour). This was a slight decrease compared to 2010, equivalent to a decreased population risk 0.2 fractures per 1000 individuals, totalling 20.5 fractures/ 1000 individuals in 2019.

Some osteoporotic fractures are associated with premature mortality [3]. In the UK, the annual number of deaths associated with a fracture event was estimated to be 114 per 100,000 individuals of the population aged 50 years or more, compared to the EU27+2 average of 116/100,000. The number of fracture-related deaths is comparable to or exceeds that for some of the most common causes of death such as lung cancer, diabetes, chronic lower respiratory diseases.

The remaining lifetime probability of hip fracture (%) at the ages of 50 years in men and women was 4.8% and 13.8%, respectively, placing the UK in the bottom tertile of risk for men and the mid tertile of risk for women.

The population of men and women age 50 years or more is projected to increase by 13.2% between 2019 and 2034, close to the EU27+2 average of 11.4%. The increases in men and women aged 75 years or more are even more marked and amount to 42.2% and 31.0%, respectively. The annual number of osteoporotic fractures in the UK is expected to increase by 138,000 to 665,000 in 2034.

**Policy framework** (Table 2)

Documentation of the burden of disease is an essential prerequisite to determine the resources that should be allocated to the diagnosis and treatment of the disorder. High quality national data on hip fracture rates have been identified in 18 of 29 countries, of which the UK is one. Data are collected on a national basis and include data on all fragility fractures as well as hip fractures.

Given that osteoporosis and fragility fractures are common and that effective treatments are widely available, the vast majority of patients with osteoporosis are preferably managed at the primary health care level by general practitioners (GPs), with specialist referral reserved for difficult complex cases. Primary care was the principal provider of the medical care for osteoporosis in the UK, as for 13 of the 28 countries where data were available.

Osteoporosis and metabolic bone disease is not a recognised specialty in most countries including the UK. Specialty care of osteoporosis in the UK is managed via other specialties including rheumatology, orthogeriatrics, metabolic medicine and endocrinology. Osteoporosis is however recognized as a component of specialty training. It is possible that these specialties educate their trainees differently, giving rise to inconsistencies in patient care. However, the Royal Osteoporosis Society has developed a competency framework for fracture prevention practitioners at the foundation and advanced level (https://theros.org.uk/healthcare-professionals/courses-and-cpd/fracture-prevention-practitioner-training/).

**Table 2** Policy framework for osteoporosis in the UK

**Table:** 

**Category**	**Measure**	**Estimate**
Policy framework	National fracture data availability	Yes
	OP recognized as a specialty	No
	OP primarily managed in primary care	Yes
	Other specialties involved	Rheumatology, Endocrinology Orthogeriatrics Metabolic medicine
	Advocacy areas covered by patient organisation	Policy, capacity, peer support, research and development

The role of national patient organisations is to improve the care of patients and increase awareness and prevention of osteoporosis and related fractures among the general public. Advocacy by patient organisations can fall into four categories: policy, capacity building and education, peer support, research and development. For the UK, all four of the advocacy areas were covered by a patient organisation, which was the case for only 10 out of the 26 countries with at least one patient organisation.

**Service provision** (Table 3)

A wide variety of approved drug treatments is available for the management of osteoporosis [4]. Potential limitations of their use in member states relate to reimbursement policies which may impair the delivery of health care. The UK is one of the 12 (out of 27) countries that offer full reimbursement bar a small prescription cost for those under the age of 60 years.

The assessment of bone mineral density forms a key component for the general management of osteoporosis, being used for diagnosis, risk prediction, selection of patients for treatment and monitoring of patients on treatment. In the UK, the number of DXA units expressed per million of the general population amounted to 7.5 which puts the country in 23^rd^ place among the EU27+2.

The average waiting time for DXA ranged from 0 to 180 days across countries, and there was no clear relation between waiting times and the availability of DXA. In the UK, the estimated average waiting time for DXA amounted to 42 days. Twenty-one countries reported shorter average waiting times.

**Table 3** Service provision for osteoporosis in the UK

**Table:** 

**Category**	**Measure**	**Estimate**	**Rank**
Service provision	Reimbursement of OP medications	100%	
	DXA units/million inhabitants	7.5	23
	DXA cost (€)	45	15
	FRAX risk assessment model available	Yes	
	Fracture liaison service density	>50%	

Reimbursement for DXA scans varied between member states both in terms of the criteria required and level of reimbursement awarded. In the UK, the reimbursement was unconditional.

The effective targeting of treatment to those at highest risk of fracture requires an assessment of fracture risk. Risk assessment models for fractures, most usually based on FRAX, were available in 24 out of 29 countries, of which the UK was one. An additional risk assessment model, QFracture, was also used in UK. For the UK, guidance on the use of risk assessment within national guidelines was available, as in only 14 of the other countries.

Guidelines for the management of osteoporosis were available in the UK (as in 27 out of 29 countries). The guidelines in the UK included postmenopausal women specifically, as well as osteoporosis in men and secondary osteoporosis including glucocorticoid-induced osteoporosis.

Fracture liaison services (FLS), also known as osteoporosis coordinator programmes and care manager programmes, provide a system for the routine assessment and management of postmenopausal women and older men who have sustained a low trauma fracture. Fracture liaison services were reported for more than 50% of hospitals in the UK.

The use of indicators to systematically measure the quality of care provided to people with osteoporosis or associated fractures has expanded as a discipline within the past decade [5]. The UK was one of few countries with national quality indicators in place.

**Service uptake** (Table 4)

The web-based usage of FRAX showed considerable heterogeneity in uptake between the countries. The average uptake for the EU27+2 was 1,555 sessions/million/year of the general population with an enormous range of 49 to 41,874 sessions/million. The use of FRAX in the UK amounted to 5,443 sessions/million in 2019, with a 137 percent increase since 2011.

Many studies have demonstrated that a significant proportion of men and women at high fracture risk do not receive therapy for osteoporosis (the treatment gap) [6]. In the EU27+2 the average treatment gap was 71% but ranged from 32 to 87%. For the UK, the treatment gap amongst women amounted to 66% or 1,761,000 out of 2,679,000 characterised at risk and increased somewhat compared to 2010. The average treatment gap among EU27+2 increased from 55% in 2010 to 71% in 2019.

**Table 4** Service uptake for osteoporosis in the UK

**Table:** 

**Category**	**Measure**	**Estimate**	**Rank**
Service uptake	Number of FRAX sessions/million people/year	5443	2
	Treatment gap for women eligible for treatment (%)	66	11
	Proportion surgically managed hip fractures	>90%	

About 5% of people with a hip fracture die within 1 month of their fracture [7] (5.8% in England and Wales [8]). A determinant of peri-operative morbidity and mortality is the time a patient takes to get to surgery [9]. For the UK, the average waiting time for hip fracture surgery after hospital admission was reported to be 1–2 days. The proportion of surgically managed hip fractures was reported to be over 90%.

**Scores and scorecard**

Scores were developed for Burden of disease and the healthcare provision (Policy framework, Service provision and Service uptake) in the EU27+2 countries. UK scores resulted in a 11th place regarding Burden of disease. The combined healthcare provision scorecard resulted in a 6th place for the UK. Thus, the UK presents as one of the high-burden high-provision countries among the EU27+2.

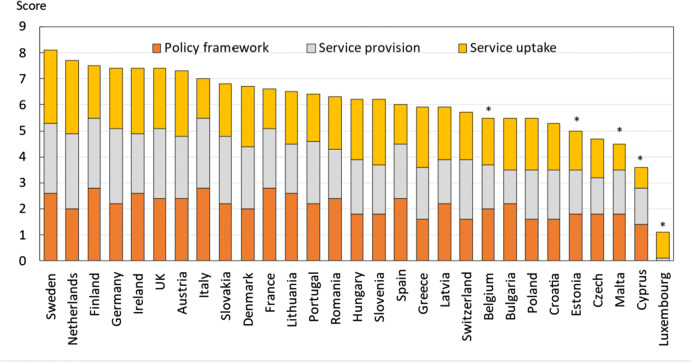



**Fig. 1** Scores by country for metrics related to policy framework, service provision and service uptake. The mean score for each of the 3 domains is given. An asterisk denotes that there was one or more missing metric which decreases the overall score

The first SCOPE was undertaken in 2010, almost 10 years previously. Fifteen of the 16 score card metrics on healthcare provision were used in the two surveys. Scores had improved or markedly improved in 15 countries, remained constant in 8 countries and worsened in 3 countries. For the UK the scores were unchanged.

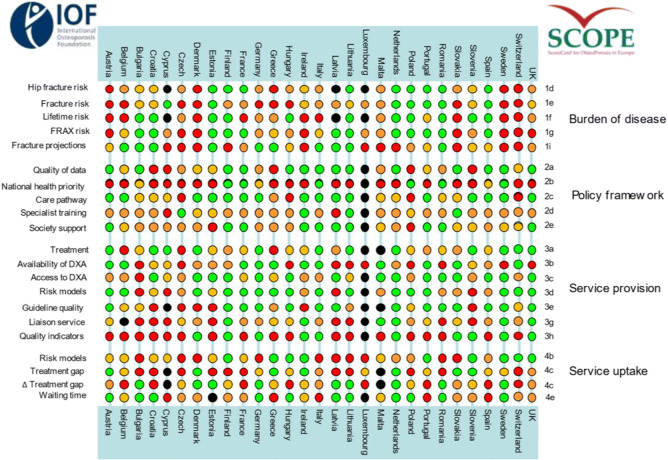



**Fig. 2** The scorecard for all the EU27+2 countries illustrating the scores across the four domains. The elements of each domain in each country were scored and coded using a traffic light system (red, orange, green). Black dots signify missing information

The second edition of the Scorecard for Osteoporosis in Europe (SCOPE 2021) allows health and policy professionals to assess key indicators on the healthcare provision for osteoporosis within countries and between countries within the EU 27+2. The scorecard is not intended as a prescriptive template. Thus, it does not set performance targets but may serve as a guide to the performance targets at which to aim in order to deliver the outcomes required.

**Acknowledgements**

SCOPE was supported by an unrestricted grant from Amgen to the International Osteoporosis Foundation (IOF). Amgen was neither involved in the design nor writing of the report. We are grateful to Anastasia Soulié Mlotek and Dominique Pierroz of the IOF for their help in the administration of SCOPE. We are most grateful to the Royal Osteoporosis Society of the UK for their valuable input. The report has been reviewed by the members of the SCOPE Consultation Panel and the relevant IOF National societies, and we are grateful for their local insights on the management of osteoporosis in each country. The source document has been reviewed and endorsed by the Committee of Scientific Advisors of the IOF and benefitted from their feedback.

**References**

1. Kanis JA, Norton N, Harvey NC, Jacobson T, Johansson H, Lorentzon M, McCloskey EV, Willers C, Borgström F (2021) SCOPE 2021: a new scorecard for osteoporosis in Europe. Arch Osteoporos 16:82. 10.1007/s11657-020-00871-9

2. World Health Organisation (1994) Assessment of fracture risk and its application to screening for postmenopausal osteoporosis. Report of a WHO Study Group. World Health Organ Tech Rep Ser, 1994/01/01 edn, pp 1-129

3. Johnell O, Kanis JA, Oden A, Sernbo I, Redlund-Johnell I, Petterson C, De Laet C, Jonsson B (2004) Mortality after osteoporotic fractures. Osteoporos Int 15:38-42

4. Hernlund E, Svedbom A, Ivergard M, Compston J, Cooper C, Stenmark J, McCloskey EV, Jonsson B, Kanis JA (2013) Osteoporosis in the European Union: medical management, epidemiology and economic burden. A report prepared in collaboration with the International Osteoporosis Foundation (IOF) and the European Federation of Pharmaceutical Industry Associations (EFPIA). Arch Osteoporos 8:136

5. Allen P, Pilar M, Walsh-Bailey C, Hooley C, Mazzucca S, Lewis CC, Mettert KD, Dorsey CN, Purtle J, Kepper MM, Baumann AA, Brownson RC (2020) Quantitative measures of health policy implementation determinants and outcomes: a systematic review. Implement Sci 15:47

6. Borgstrom F, Karlsson L, Ortsater G, Norton N, Halbout P, Cooper C, Lorentzon M, McCloskey EV, Harvey NC, Javaid MK, Kanis JA (2020) Fragility fractures in Europe: burden, management and opportunities. Arch Osteoporos 15:59

7. Kanis JA, Oden A, Johnell O, De Laet C, Jonsson B, Oglesby AK (2003) The components of excess mortality after hip fracture. Bone 32:468-473

8. Royal College of Physicians (2019) The Falls and Fragility Fracture Audit Programme (FFFAP). https://www.nhfd.co.uk/20/NHFDcharts.nsf/vwCharts/Mortality?open&org= , accessed 12 March 2021

9. National Clinical Guideline Centre (2011) The Management of Hip Fracture in Adults. In Centre NCG (ed) London

## Abbreviations

BMD: Bone mineral density
DXA: Dual-energy X-ray absorptiometry
EU27+2: Refers to the 27 countries of the European Union plus Switzerland and the UK
FLS: Fracture liaison service
FRAX: Fracture risk assessment tool
HRQoL: Health-related quality of life
IOF: International Osteoporosis Foundation
OP: Osteoporosis
QALY: Quality-adjusted life year
SCOPE: Scorecard for osteoporosis in Europe
TBS: Trabecular bone score

